# Modelling and implementation of soft bio-mimetic turtle using echo state network and soft pneumatic actuators

**DOI:** 10.1038/s41598-021-91136-z

**Published:** 2021-06-08

**Authors:** MennaAllah Soliman, Mostafa A. Mousa, Mahmood A. Saleh, Mahmoud Elsamanty, Ahmed G. Radwan

**Affiliations:** 1 Mechanical Engineering Program, School of Engineering and Applied Sciences, Nile University, Sheikh Zayed City, 12588 Egypt; 2 Nanoelectronics Integrated Systems Center (NISC), Nile University, Sheikh Zayed City, 12588 Egypt; 3 Smart Engineering Systems Research Center (SESC), Nile University, Sheikh Zayed City, 12588 Egypt; 4 Mechanical Department, Faculty of Engineering at Shoubra, Benha University, Cairo, 11672 Egypt; 5 Department of Engineering Mathematics and Physics, Cairo University, Giza, 12613 Egypt; 6 School of Engineering and Applied Sciences, Nile University, Sheikh Zayed City, 12588 Egypt

**Keywords:** Electrical and electronic engineering, Mechanical engineering

## Abstract

Advances of soft robotics enabled better mimicking of biological creatures and closer realization of animals’ motion in the robotics field. The biological creature’s movement has morphology and flexibility that is problematic deportation to a bio-inspired robot. This paper aims to study the ability to mimic turtle motion using a soft pneumatic actuator (SPA) as a turtle flipper limb. SPA’s behavior is simulated using finite element analysis to design turtle flipper at 22 different geometrical configurations, and the simulations are conducted on a large pressure range (0.11–0.4 Mpa). The simulation results are validated using vision feedback with respect to varying the air pillow orientation angle. Consequently, four SPAs with different inclination angles are selected to build a bio-mimetic turtle, which is tested at two different driving configurations. The nonlinear dynamics of soft actuators, which is challenging to model the motion using traditional modeling techniques affect the turtle’s motion. Conclusively, according to kinematics behavior, the turtle motion path is modeled using the Echo State Network (ESN) method, one of the reservoir computing techniques. The ESN models the turtle path with respect to the actuators’ rotation motion angle with maximum root-mean-square error of $1.04 \times 10^{-11}$. The turtle is designed to enhance the robot interaction with living creatures by mimicking their limbs’ flexibility and the way of their motion.

## Introduction

Robots are machines developed to perform tasks previously carried by humans in multiple fields. Robots utilization in industry led to revolutionary progression in industrial automation. Robots could replace labor in repetitive tasks and proved to be more accurate for a significantly longer periods of time, and also replaced humans in hazardous environments. The efficiency provided by robots extended their usage in other tasks in shared environment with humans. However, the rigidity of the robots was an obstacle to realize the Human–Robot Interaction (HRI) in an appropriate manner^1^.

Towards a shared environment where humans and robots can work collaboratively, researchers began the development of robots inspired by biological organisms. Living organisms originally possessed high degree of rationality in structure, function execution, environment adaptation, and autonomous learning^1^. One of the important physical attributes of living organisms is their body flexibility which is realized in robots that can provide a shared working environment between robots and humans.

Advances in biomimicry of robots led to the rise of soft robotics domain in robotics science^2^ which are fabricated by soft materials^3^. This physical nature enabled it to perform tasks that were not possible or that required extensive computations and complex sensorial system like handling fragile objects^4^. For example, it enables the manufacturing of bio-mimicked animal soft robot^5^. Soft actuators exploited higher degrees of freedom than rigid ones^6,7^ based on its structural parameters optimization^8^. However, unlike hard robots that had well-defined models and constant kinematics, soft robots are challenging to model, and it is difficult to capture its deflections and surface irregularities^9^ which could be simulated using finite element analysis (FEA)^10^.

The inspirations of bio-mimicked robots come from observing the behaviour of living creatures from the animal kingdom even though the plants^11^. Multiple works were presented that reproduced animal motion through robotics^12–16^. Some robots even conformed using biological tissues within its structure^17,18^. Cephalopods were a source of inspiration to develop propulsor unit of a biomimetic soft robotic siphon^12^. This was achieved through developing circumferential siphon actuation muscles and a new central flow-regulative duct. The presented work^12^ enables better water-jetting for underwater robots and hence an improved propulsion.

Caterpillars crawling locomotion was investigated using soft materials and rigid magnetic parts to build a modular crawling robot “MagWorm”^13^. This robot is unique for being developed on a centimeter scale, where the relation between the robot’s number of segments used and the speed of the driving magnet were discussed. It was found that on increasing its speed, increased number of segments enhances the robot’s performance.

Recently, multiple works were presented to discuss the turtle motion principle and its bio-mechanism^19–24^.The proposed robots in these works are depend on motors or rigid actuators for locomotion and modelled by traditional kinematics and dynamics hard robot analysis. One instance is the design and fabrication of a morphing limb that exploits amphibious locomotion. A variable stiffness composite was used to realize the transition of the actuator from the leg state to the flipper state^21,22^. This was achieved by varying the electrical current of the heaters bounded to the thermal responsive composite. In addition, the flipping motion of the turtle leg was developed using a soft pneumatic actuator that bends in the 3D space. Moreover, helical soft actuators were developed to enhance the gripping feature of the irregularly shaped bodies^25,26^. This work defer from the other work as the robot actuator is soft actuator. Also, the 3D-bending actuator showed the effect of the air chamber inclination angle on the final tip position of the actuator has been achieved^26^. Similar to the previous work, the fabrication process adopted is additive manufacturing where a Fused Deposition Modeling (FDM) printer is used to directly print the actuator. However, the work is depend on morphing the robot actuator and analysis its motion and it does not mention the analysis study of the robot motion with the proposed actuator. Electronics free turtle which use oscillating valves to allow pressurized air in and out in a predetermined order is proposed^27^. The robot is tested by experiments depends on the motion gait analysis for the robot not the dynamics model analysis.

In this paper, a turtle robot is developed using SPAs with different oriented air chambers. The actuator motion is simulated by Finite Element Analysis (FEA) simulation tool at different angles of orientation of the air chambers. The FEA is used as a study for design optimization like-wise the study in^28^. The SPAs are tested over a large range of pressure starting from 0.1 MPa to 0.4 MPa with a step 0.01. After comparing the behavior of the fabricated actuators to the simulation results, two actuators are selected acordingly to be used as the turtle morphing limbs. The kinematics of the fabricated turtle is modelled using an Echo state network^29–32^. The robot is using soft actuator and the motion of the robot is modelled with consideration of the soft actuator chaotic dynamic behaviour which differs this work from the previous ones.

Echo state networks is one form to realize reservoir computing. Reservoir computing is adopted due to the severe difficulty to capture the nonlinear dynamics of the fabricated actuators^33^. Echo state network is one form of reservoir computing methods which is commonly used in robotics^34^. Its basic architecture consists of an input layer, reservoir layer, and an output layer. The reservoir layer is a Recurrent Neural Network (RNN) that has some sort of sparsity. This sparsity contributes to the elimination of the noise present at the input side. The weights of this RNN are completely random and are not trained, while the weights at the input and output layers are trained.

## Methods

### Design criteria

#### SPA: turtle geometrical presentation

**Figure 1:**
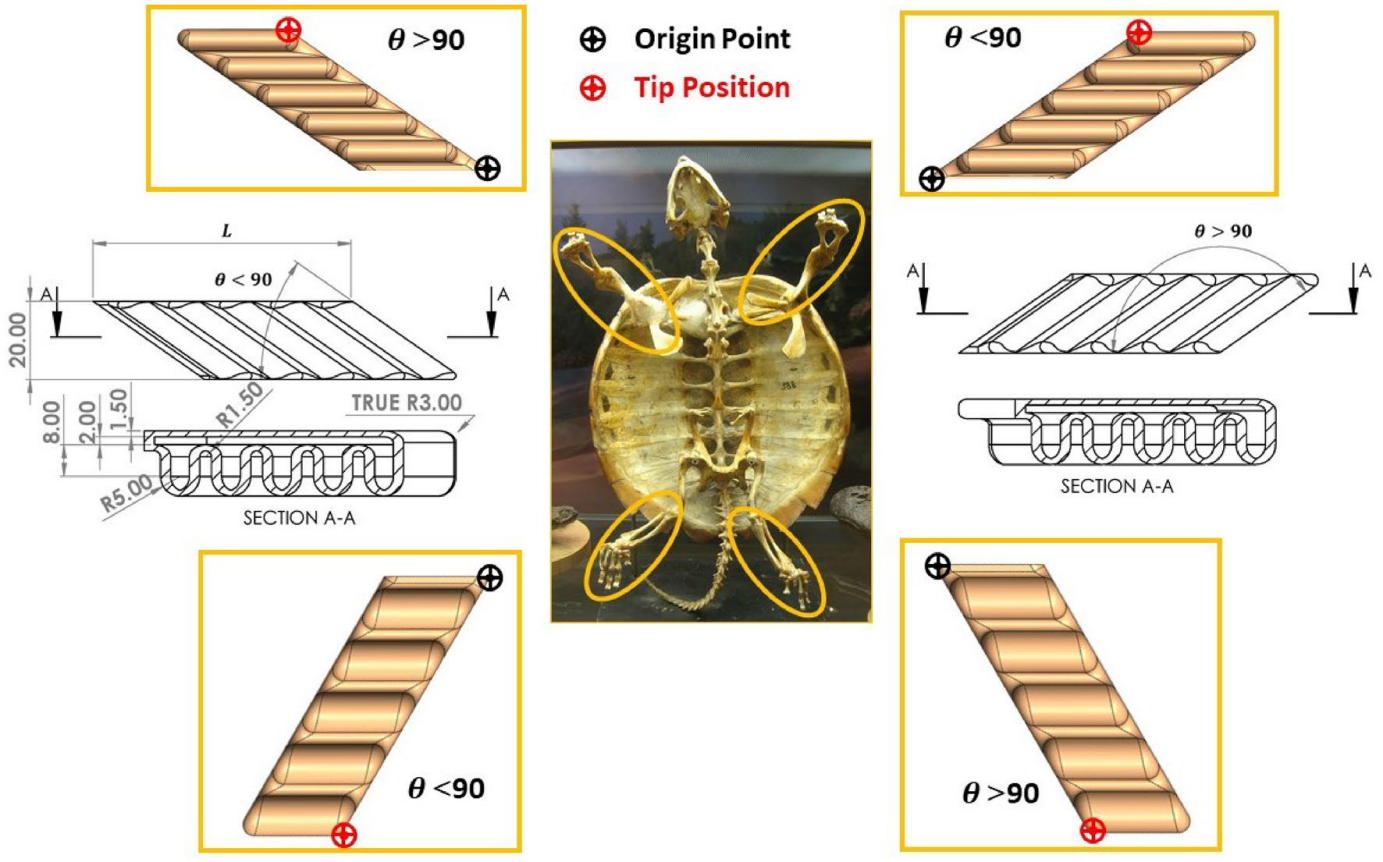
The similarities between the turtle flipper’s anatomy and the soft oriented angle pneumatic actuator with the CAD designs of SPA. The figure shows that the front flippers have wider rotation range than the back ones. The designed actuators motion is from morphology of the real turtle. Every flippers has its origin point and tip position point that the analysis is implemented based on it.

The turtle skeleton has four flippers with specific rotation ranges in 3D. The front flippers provide wider rotation ranges than the back ones^23^. In order to mimic the turtle’s movement according to its anatomy, each pair of front and back flippers should have the same rotation range in 3D but in opposite directions to each other. To achieve this, an SPA is designed and studied with variable oriented air pillow angle $\theta$. The SPA $\theta$ angle will provide the needed rotation range for each flipper. To achieve this design scheme, the $\theta$ angles for the two front and back flippers should be supplementary to each other as illustrated in Fig. 1. The proposed mimicked SPA, which will be studied at different $\theta$ angle from $35^{\circ }$ to 1$45^{\circ }$ with $5^{\circ }$ step angle (22 SPAs). The SPAs are selected to be in cylindrical embedded design shape due to its more durability, high resistance for layers delamination under high applied pressure and the ease of manufacturing as stated in^35^. For simplification, the actuators will be named as follows; actuators designed at $\theta = 35^{\circ }$ will be mentioned as “SPA35” and likewise for the rest of the actuators. The SPA is designed using CAD software (Dassault System SOLIDWORKS$^{\mathrm{TM}}$ Corp. MA).

#### Simulated model using FEA

The effect of change in $\theta$ angle on the SPA rotation range in 3D is studied and simulated using FEA. This study is conducted to simulate the behavior of every SPA rotation range in 3D coordinate (X, Y, Z) with respect to different inlet pressure (minimum absolute pressure 0.11 MPa, maximum absolute pressure 0.4 MPa at incremental step of 0.01 MPa) using Ansys Multi-Physics 2019 R2$^{\mathrm{TM}}$. FEA analysis is conducted for 22 different $\theta$ angles. The $\theta$ SPAs are fabricated with (Thermoplastic Polyurethane) TPU material using (Fused Deposition Modeling) FDM 3D Printer. TPU material is one type of soft material that is suitable for 3D printing. However, the material is geometrically nonlinear with hyperelastic behavior, but most of its characteristics are needed to be identified. So, uniaxial tests have been performed to a printed TPU material on a standard tension specimen ISO 37. Uniaxial test data is fitted to the Mooney-Rilivin five parameters material hyperelastic model like the same procedure mentioned in^28^. The material model parameters are:

1 $$\begin{aligned} \begin{aligned} C_{10}&= -\,1.2554 \frac{\mathrm{kJ}}{\mathrm{mm}^3} (\mathrm{MPa}),\quad C_{01} = 5.316 \frac{\mathrm{kJ}}{\mathrm{mm}^3} (\mathrm{MPa}), \quad C_{20} = -\,0.009697 \frac{\mathrm{kJ}}{\mathrm{mm}^3} (\mathrm{MPa}), \\ C_{11}&= 0.13421 \frac{\mathrm{kJ}}{\mathrm{mm}^3} (\mathrm{MPa}), \quad C_{02} = 0.15704 \frac{\mathrm{kJ}}{\mathrm{mm}^3} (\mathrm{MPa}), \end{aligned} \end{aligned}$$

**Figure 2:**
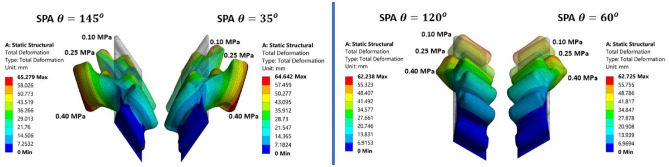
FEA results of accumulated deformation images on top of each other to show the effect of applied pressure on variable $\theta$, SPA60, SPA120, SPA35, and SPA145 work envelope. FEA simulated work envelope for variable $\theta$ SPAs.

**Figure 3:**
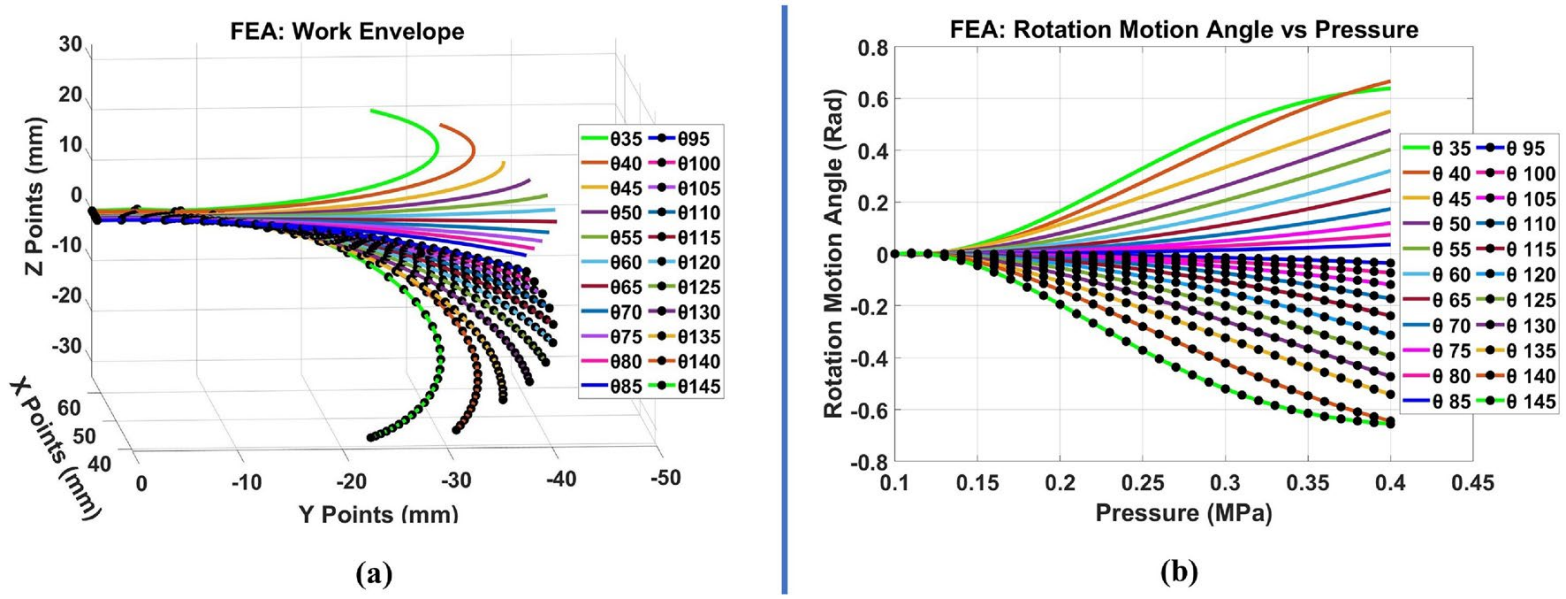
(**a**) FEA (X, Y, Z) work envelope in 3D, (**b**) FEA rotation motion angle.

The 3D nonlinear mechanical physical preference finite element models are built for mimicked SPA using the previous hyperelastic model with large deflection mode activation to consider the nonlinearities in the material. All the geometrical solids of the SPAs are modeled using solid tetrahedral quadratic mesh elements due to the hyperelastic material behavior and to smoothly capture the large curvatures during the simulation. The mesh element size is obtained and converged between 1.2 and 2.0 mm. Fixed support boundary condition is applied at facial face of every $\theta$ SPA where the SPA origin is located at its lower tip. Atmospheric pressure 1 bar/0.1 MPa is applied for the outer faces of SPAs and pressure range of 0.11:0.01:0.4 MPa is applied on inner faces of every SPAs. The work envelope of every simulated SPA is measured at the end tip with respect to the origin point at the facial face of SPA. The 3D rotation of SPAs ($\theta =35^{\circ },\,60^{\circ },\,120^{\circ } \,and \, 145^{\circ }$) under 0.1,0.25 and 0.4 MPa inner faces air pressurization obtained from FEA are illustrated in Fig. 2. The whole 3D work envelopes as (*X*, *Y*, *Z*) tip position with respect to origin point of the 22 simulated SPAs are illustrated in Fig. 3a. SPAs FEA motion rotation angles between tip Z and X position (Z/X angle) are illustrated in Fig. 3b. Every two SPAs with supplementary $\theta$ angles have approximately identical 2D rotation (X,Y) range in magnitude and direction and opposite in Z direction with approximate magnitude as shown in Supplementary Fig. S8.

### Experimental validation

#### Experimental setup

To assure the behaviour of the SPAs, four samples are chosen from the 22 simulated SPAs to be tested and printed using 3D Fused deposition modelling (FDM) printer.

**Figure 4:**
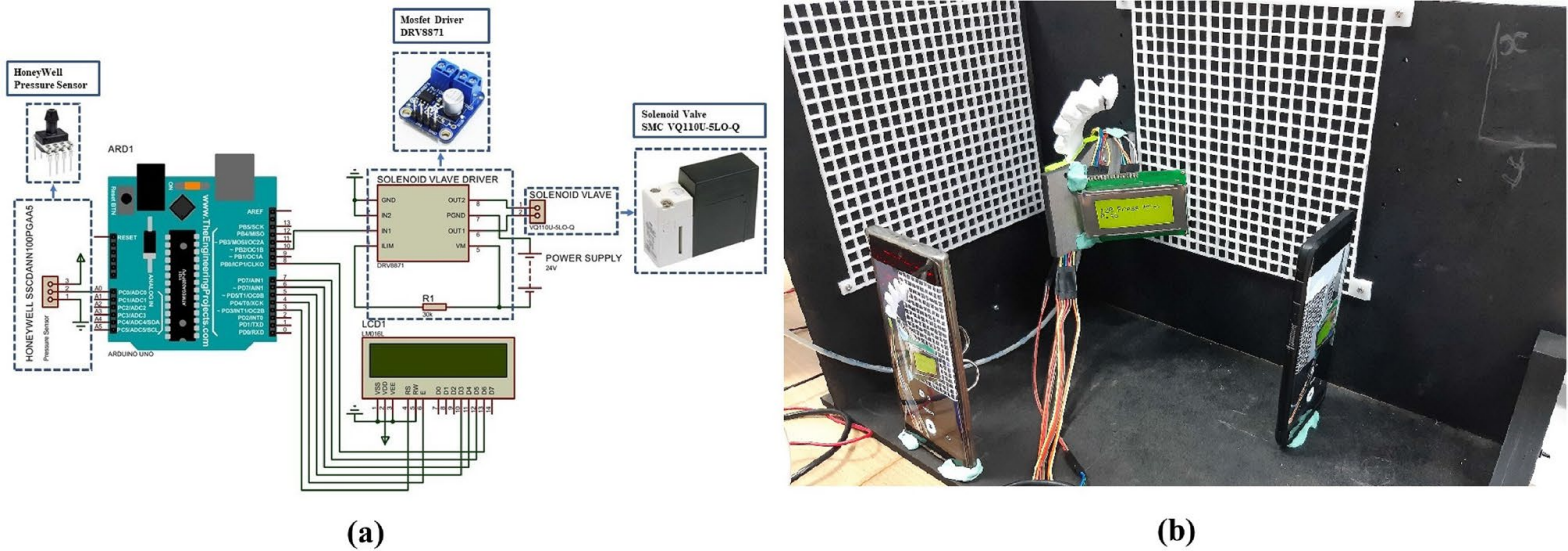
Experiment setup of testing SPA motion. (**a**) The pneumatic circuit used to control the applied pressure on SPA which changes with the PWM. (**b**) The SPA two frames captured with video for vision feedback to extract the coordinates of the SPA tip from each plane to be compared with FEA simulation.

The four SPAs are SPA60, SPA120, SPA35, and SPA145, the first two are chosen as they show the most significant 3D motion in simulation. The last two are the maximum inclination angle that can be printed. The four SPAs are powered by pneumatic pressure controlled by a pneumatic circuit shown in Fig. 4a. The pneumatic circuit consists of Pulse Width Modulation (PWM) with 100Hz to control the high-speed valve that changes the pressure from 0.1 to 0.4 MPa continuously. The position of the end tip of the SPA is recorded by two videos, one for the XY plane and one for the XZ plane to be able to extract X, Y, and Z points of 3D motion of the SPA. The two recorders are analysed using tracker software to capture the coordinates of the tip. Tracker software is an analysis software for video recordings built on the Open-Source Physics (OSP) Java framework^36^ [30]. The software has a feature of auto-tracking the position of a selected pixel in the video and gets its coordinate over time. The auto-tracking feature is used to track the 3D coordinates of the SPA tip from two planes (XZ and XY). The experiment is repeated for 5 to 7 trials, depending on the reliability of SPA to high pressure. The experimental setup and the two planes are shown in Fig. 4b.

#### Actuator behaviour

**Figure 5:**
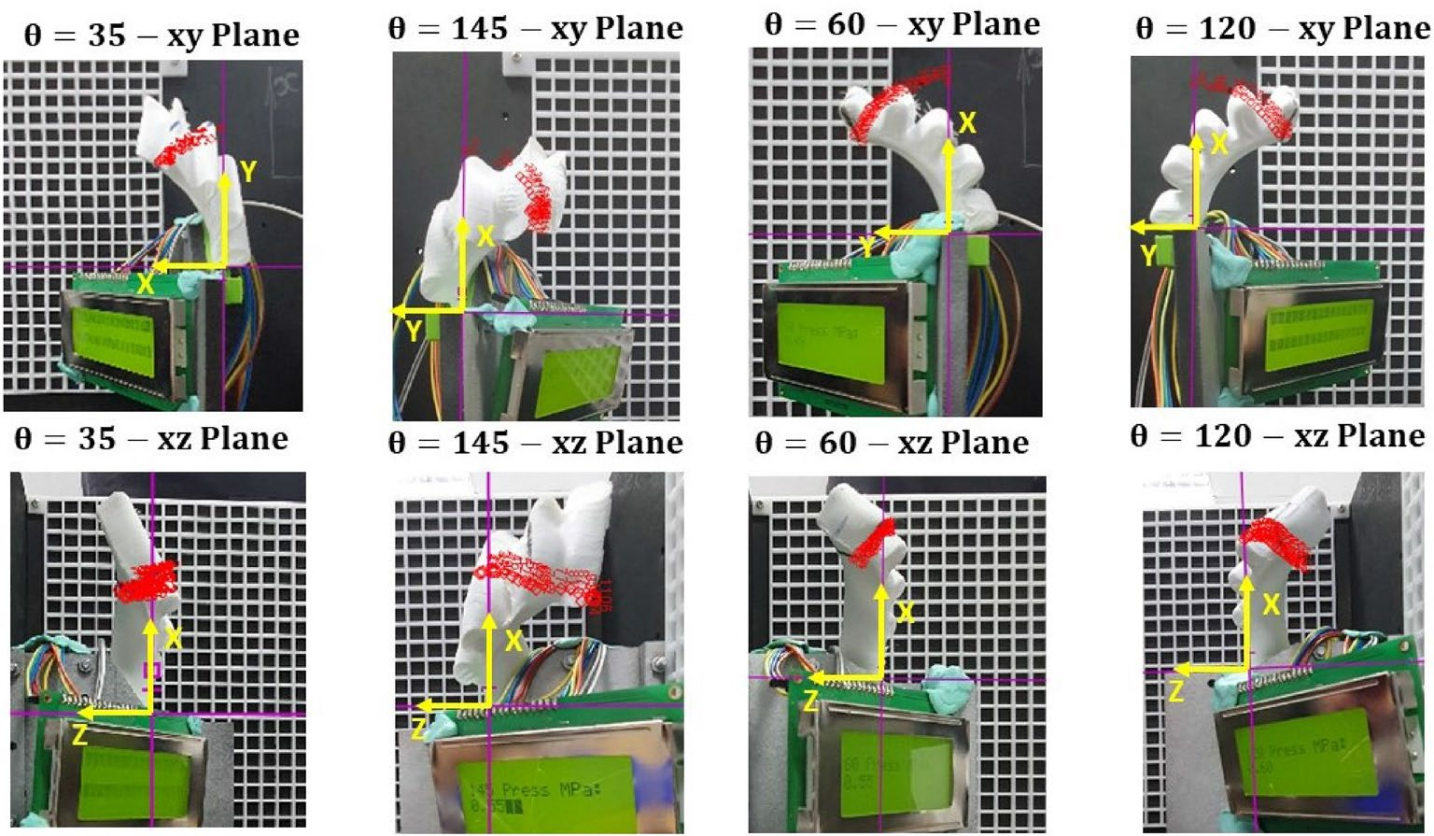
The two frames (XY and XZ) for 4 manufactured SPAs, the figures are snap-shots from tracker software where the origin axis in purple and the tip position throughout the trial is indicated in red circles.

**Figure 6:**
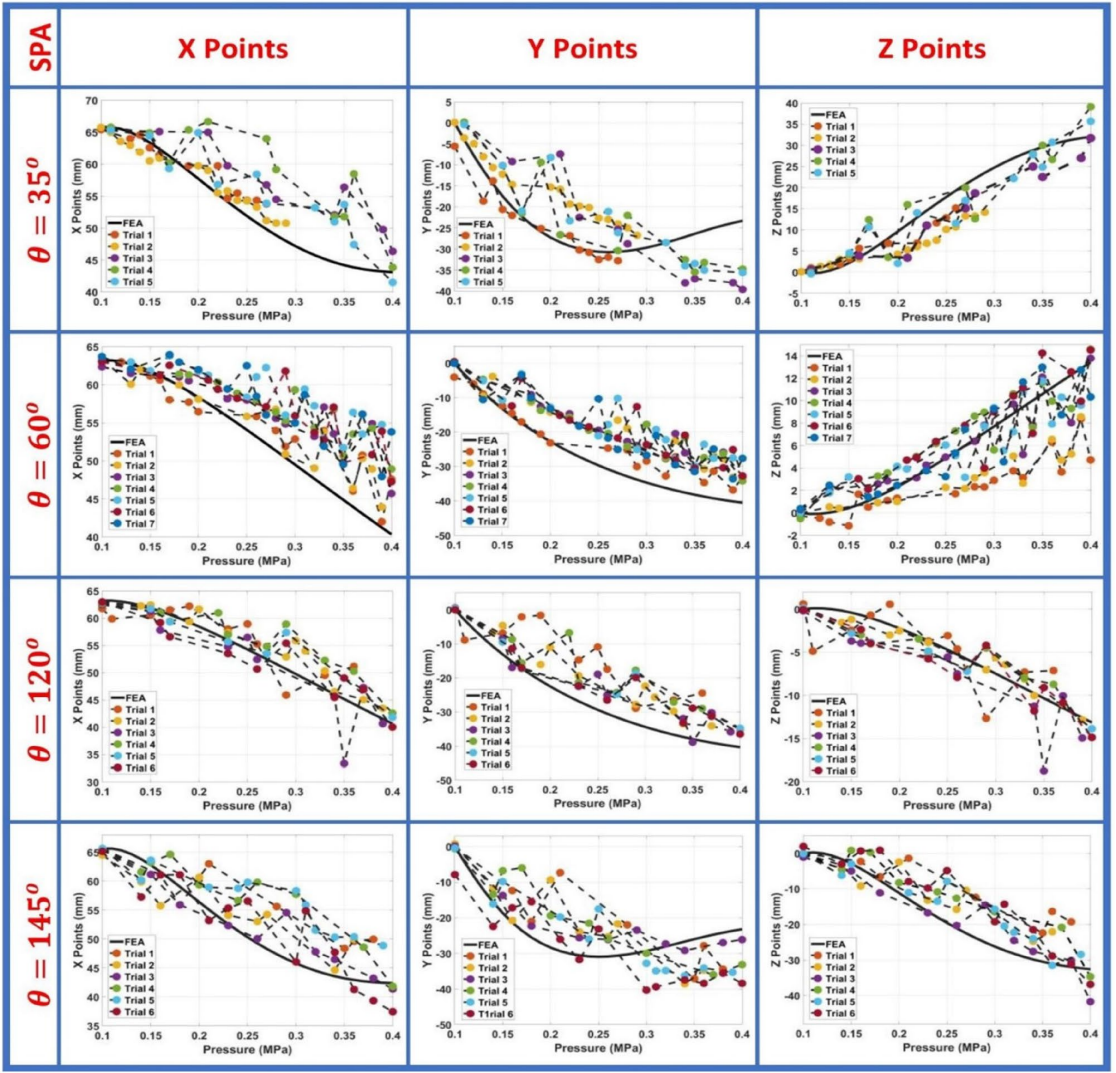
Experimental versus FEA for SPA35, SPA145, SPA60, and SPA120 for X, Y and Z coordinates points.

The experiment is repeated from 4 to 7 times for each SPA due to air leakage and the uncertainty of the point tracking. The experimental data is extracted from tracker software for X, Y, Z points as shown in Fig. 5 and is compared to the finite element simulated curve for each axis, as shown in Fig. 6 and the difference between calculated rotation angle (Z/X) of FEA and experiments is shown in Supplementary Fig. S7 which is similar to Z coordinates curves. The experimental data curves behaviour is similar to FEA data. However, there is an error ranging from (2-10 mm) for all SPA due to the unavailability of axial and shear data tests in the FEA material model. The error notably appears at high pressure above 0.4 MPa as the compressed air starts to leak from the SPA and motion in 3D increases. The motion of the SPA and it supplementary are the same in X and Y curves and opposite in Z curve with a slight difference in values, which will help the turtle’s motion and affect its path. Taking the SPA with $\theta =120^{\circ }$ as an example to describe the behaviour of motion for using SPA as an actuator, the error between simulation and real data coordinates increases as the pressure exceeds 0.4 MPa. The error between real and simulated data increased from trial 1 to trial 6. There are two observations: first, the air leakage happens at high pressures as the 3D printed layers are detached from each other. The second observation is that the deformation gradient increases on increasing the number of trials for a specific SPA. This is the justification for the increasing error at every trial.

This specifies the reliability of SPA during trials to sustain the same motion behaviour before its failure. Overall, the SPAs SPA120, and SPA145 have less error than SPA35, and SPA60, which have increased air leakage. SPA120, and SPA145 3D printing fabrication is in direction of the pole of the 3D printer, in contrast SPA35, and SPA60, which results in better adhesion of layers in SPA120 and SPA145.

### Turtle prototype

After simulating the SPAs with FEA, SPA35 and SPA145 are selected as the turtle’s front legs due to their wide rotation range in 3D and SPA60, and SPA120 are selected as the back legs due to their medium rotation range based on the turtle morphology. The selection of the front leg actuators is inspired from anatomy of turtle flippers. The front flippers of the turtle have large deflection angle than back flippers as they are responsible for dragging the turtle body on the land while the back flippers support. SPA35 and SPA145 show large rotation angle than the other SPAs which allow them to be used as the turtle robot front legs The points each SPA can reach in 3D are presented in Table 1.

**Table 1:** Comparing XYZ coordinates and Z/X rotation motion angle for front and back SPAs at 0.2 and 0.4 MPa.

SPA	Pressure (MPa)	X (mm)	Y (mm)	Z (mm)	ZX rotation motion angle (Rad)
SPA35	0.2	57.22	− 27	9.62	0.165
	0.4	43.11	− 23.26	31.03	0.639
SPA 145	0.2	56.38	− 28.37	− 11.14	− 0.195
	0.4	42.38	− 23.21	− 32.59	− 0.655
SPA 60	0.2	58.35	− 22.75	2.31	0.039
	0.4	40.35	− 40.51	13.42	0.321
SPA 120	0.2	48.46	− 22.46	− 2.23	− 0.038
	0.4	40.63	− 40.31	− 13.14	− 0.312

Finally, a 3D printer is used to fabricate the 4 selected SPAs using the FDM Felix Tec 4$^{\mathrm{TM}}$ 3D printer. The CAD design is sliced using the software CURA$^{\mathrm{TM}}$. The printing parameters are 0.1 mm layer height, 0.4 mm line width, and $220\,^{\circ }\hbox {C}$ printing temperature with enabling ironing feature and 100% infill density. To solve the SPA layer adhesion issue especially between the intersect layers between side wall and top layers, inner fillet is added between those two faces to increase the contact area. Also, by using these fillets as shown in Supplementary Fig. S3 and set the SPA orientation on 3D printing bed in the pull direction of extrusion nozzle as shown in Supplementary Fig. S4, support material for the SPA top face during the 3D printing is avoided. For the turtle final prototype, base form 3 mm thickness acrylic material is manufactured using CO_2_ laser cutter machine. The acrylic base is used alongside with 4 SPAs to complete the final soft turtle robot morphology as shown in Supplementary Figs. S5 and S6.

## Results

### Turtle motion modelling

#### Turtle paths

**Figure 7:**
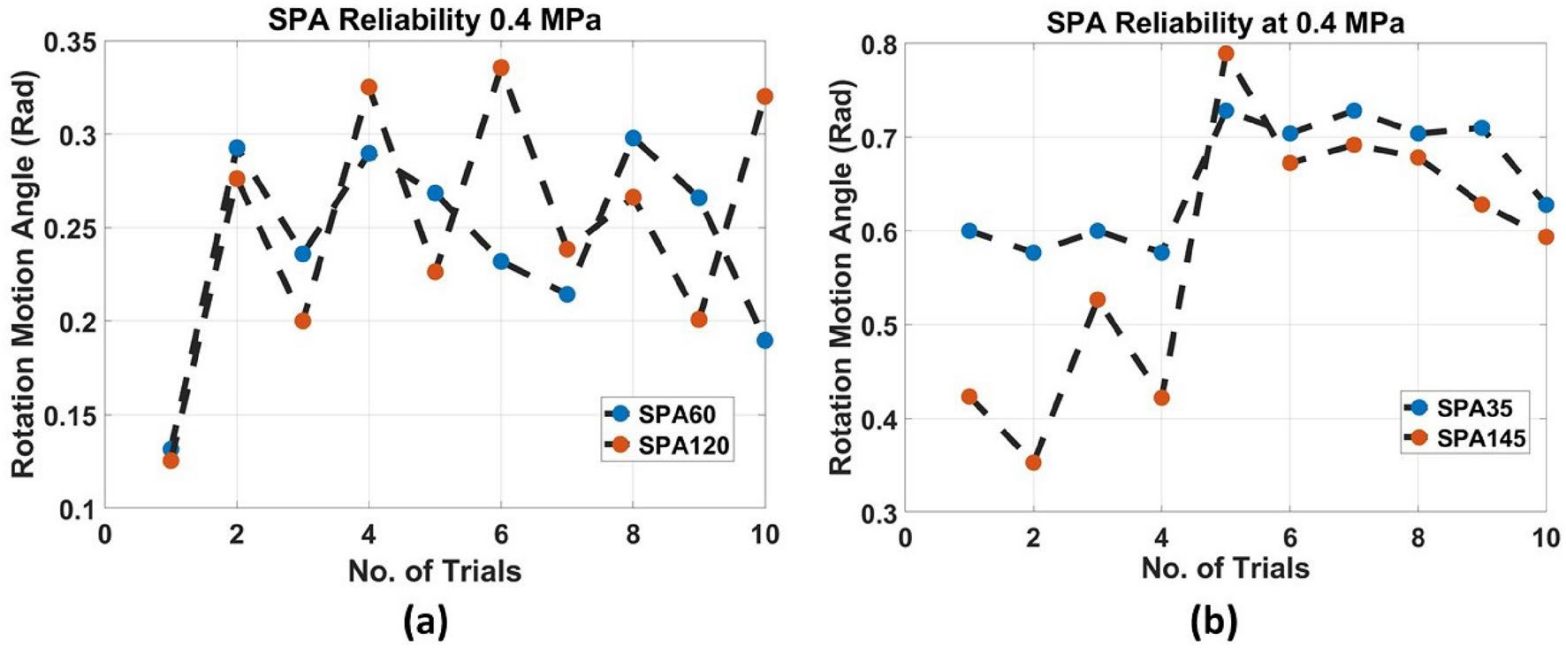
Reliability performance for SPAs against different trials at applied pressure of 0.4 MPa, (**a**) reliability performance for SP60 and SPA120, (**b**) reliability performance for SPA35 and SPA145.

For testing the turtle prototype ability of motion; the turtle is actuated using front-drive SPAs; SPA60, and SPA120, or back-drive SPAs; SPA35m and SPA145 in real-time. In each trial, the pressure is sustained at 0.4 Mpa as it is observed from the experiments of the SPAs that this is the yielding point of SPA performance. Above 0.4 Mpa pressure, the SPA coordinates start to deviate from simulation results, the air leakage increases, and the reliability of the SPA decreases. This happened due to the increase of the strain energy of hyperelastic material, which increases the deformation gradient of the SPA. So, by choosing 0.4 MPa pressure; The SPA maintains the highest 3D performance and the longest lifetime. For each actuator, the pressure is applied in a sequence of on/off with a time interval of 0.33 s to allow the SPA to perform its cycle and reach the maximum rotation angle. The applied pressure causes the turtle flippers to rotate at a specific angular velocity, analogous to the mobile robots’ wheels. For the turtle’s front-drive SPAs SPA60, and SPA120, the turtle moves 916 steps in 32.3 s with 8.5 mm/s velocity. The distance covered by the robot in the X-axis is almost linear, which is 275.91 mm, and inclined in Y-axis ranging from − 176 to − 182 mm. The robot is supposed to move in a straight line, however, the robot slides toward SPA120 after 10 s from the beginning of the trial as the SPA60 has air leakage and its reliability to sustain the same rotation motion angle is small as shown in Fig. 7a. For the back-drive SPAs; SPA35, and SPA145, the turtle takes 45.6 s to perform 1312 steps with 4.3 mm/s velocity. The path in X-axis is linear but with small ripples and it covered 704.37 mm. It has a pyramid shape in Y-axis ranging from 135 to 155 mm. The inclination and ripples in the path due to difference in the rotation motion angle of SPA145, and SPA35 during the experiment is largely different as shown in Fig. 7b. The results is prove that using SPA35 and SPA145 as front legs of the turtle is better in dragging robot body as it mention in turtle prototype section.

#### Echo state modelling using reservoir computing

**Figure 8:**
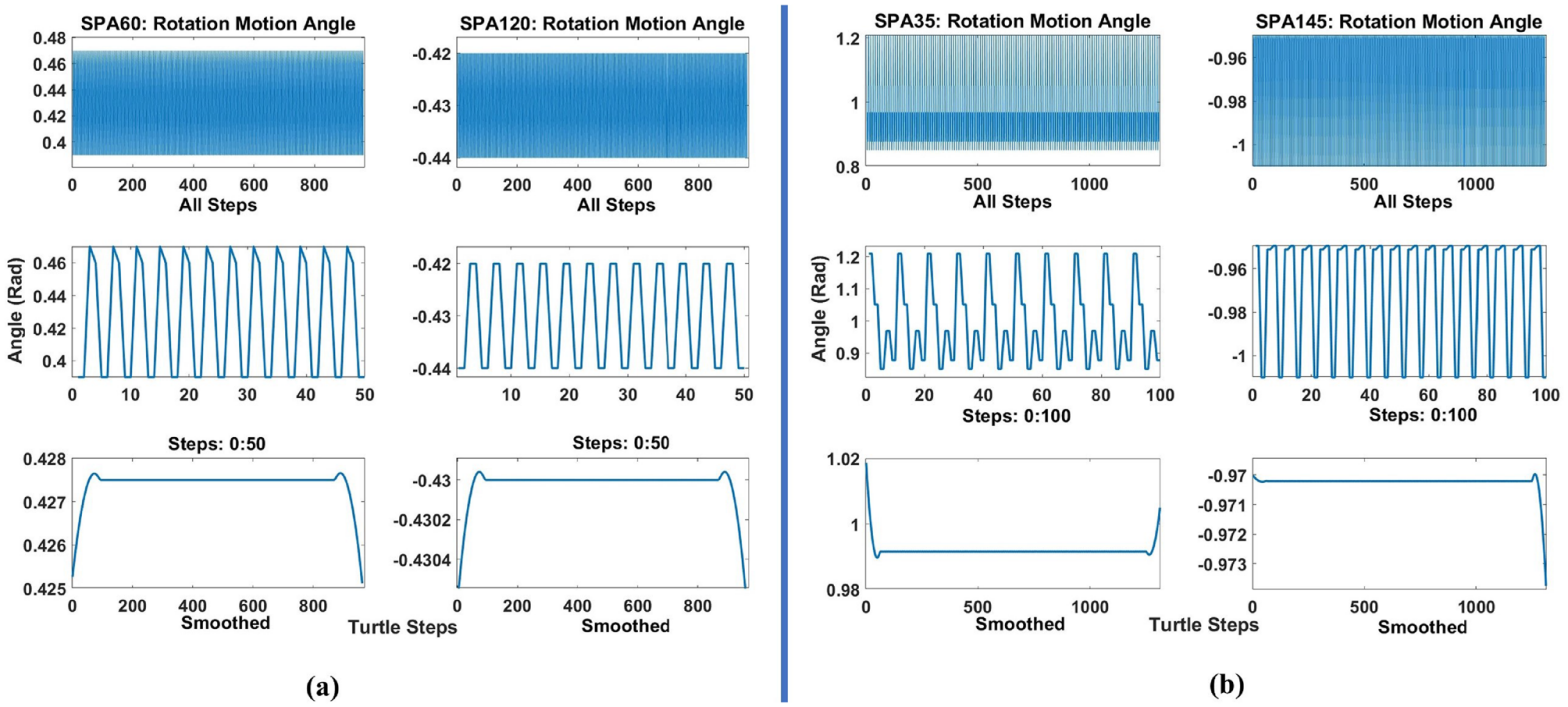
(**a**) SPA35, and SPA145 rotation motion angle hysteresis input for turtle movement, (**b**) SPA60, and SPA120 rotation motion angle hysteresis input for turtle movement.

**Figure 9:**
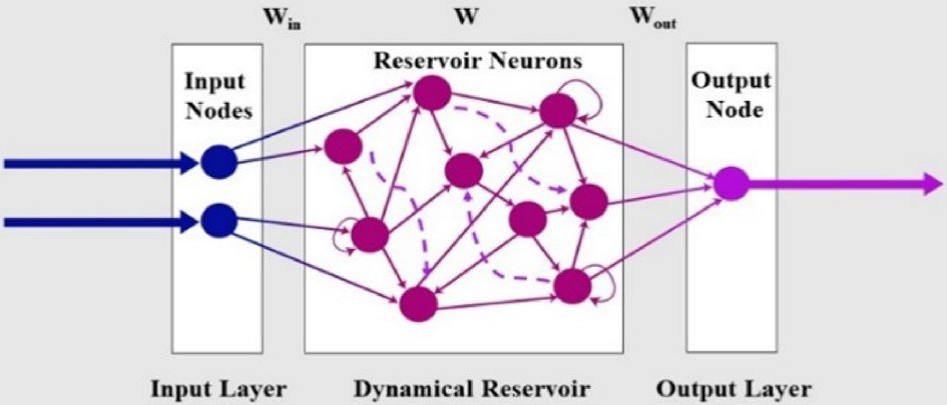
Echo state neural network architecture.

The uncertainty conditions that define the path of the turtle robot, which is predictable for a certain time, changes its motion randomly. This behaviour shows that the scenario of motion makes the turtle robot system defined as a chaotic system. The behaviour is obvious from what is described in the previous section when the robot seemed to have an inclined path based on the behaviour of the SPA actuators that changes through time and based on the sustaining of the pressure values. Figure 8a,b shows the input angle (defined by angle made by SPA (Z/X) cycle of each actuator to turtle robot; that responsible to push the robot forward; proves the instability of the system which shows high hysteresis. The hysteresis cycles appearing in SPA35, and SPA145 are bigger than SPA60 and SPA120, and by comparing the output paths of both it shows that the increasing hysteresis in SPA35 and SPA145 causes more ripples and slipping in the path of the turtle. Modelling the motion of turtle needs to consider the chaotic spatiotemporal behaviour of the actuator in response to the input pressure, to be able to predict the turtle motion. Regarding the modelling technique, the standard feedforward Neural Network (NN) techniques are investigated which demonstrate 80% accuracy which implies large error (3 cm mean absolute error). Then, the model-free one has been chosen which demonstrates the effect of actuator angles on the robot’s motion with correlation factor R = 0.9 (best results). The fact that the body is taking part in controlling the robot’s motion is called morphology, which makes it hard to map the input to the output of the high nonlinear dynamic system. Reservoir computing technique is one of recurrent neural network (RNN) used to model complex dynamic system and “Echo State Network” is one method of these techniques that are usually used in robotics. The Echo State method is chosen as it considers the change of SPA behaviour over time as it is one form of RNN networks, and it can take varying time step. The architecture of the echo state network is shown in Fig. 9. Echo State Network (ESN) consists of a non-trainable input layer ($W_in$) connected to a random dynamic system called reservoir (W) that creating higher representation (embedding). The embedding is connected to the desired output through a trainable unit ($W_out$) that produce temporal patterns for the system. Back projection nodes ($W_back$) are stored to connect the projection back from the output to the internal unit. The activation scheme for the internal units is:

2 $$\begin{aligned} x(n+1)=f(W_{in} u(n+1)+Wx(n)+ W_{back} , \end{aligned}$$

where $f=(f_1,\ldots ,f_N)$ are output functions of internal nodes (dynamic reservoir), *x*(*n*) is activation state of random nodes which is a function of input history $(u(n),u(n-1),\ldots )$ entered the network. Then, the output is calculated as:

3 $$\begin{aligned} y(n+1)=f_{out} (W_{out} (u(n+1)),x(n+1),y(n))) , \end{aligned}$$

where $f_out=(f_(out_1),\ldots ,f_(out_L))$ are output functions of output nodes and (L) is the number of output nodes.

For the used ESN architecture in modelling the system, the turtle system has two inputs whenever it works by front-drive or by back drive which are the rotation angles of the actuator’s rotation motion angle and two outputs that are the X and Y coordination of turtle path.

**Figure 10:**
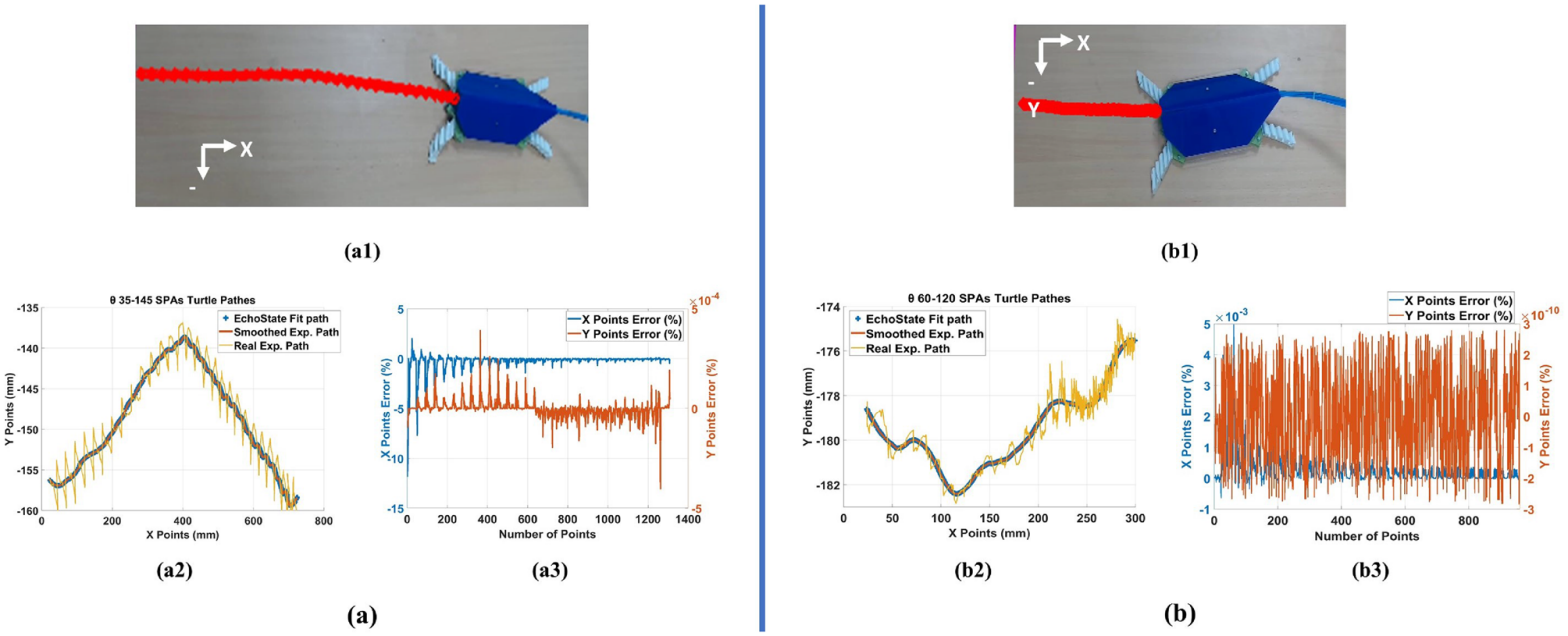
(**a**) Turtle movement actuated by front drive SPA145 and SPA35 modeling by ESN, (a1) Turtle path from tracker software, (a2) XY coordinates of turtle path, smoothed path, and result path from ESN. (a3) Percentage of error in X and Y points. (**b**) Turtle movement actuated by front drive SPA60 and SPA120 modelling by ESN, (b1) Turtle path from tracker software, (b2) XY coordinates of turtle path, smoothed path, and result path from ESN, (b3) Percentage of error in X and Y points.

Consequently, the ESN has two input nodes and two output nodes, the internal dynamic reservoir has 10000 nodes with 0.25 spectral radius and 0.95 sparsity. These parameters are chosen by trial to get the minimum error for the fitting problem. The output layer is trained using “tanh” activation function. The ESN trained by 1312 sets of data for back drive SPAs trial and 916 sets of data for front-drive SPAs. The training input data is smoothed using Lowess smoothing method with 10% of the total number of data points shown in Figs. 7 and 8. The output and input are normalized by rescaling the data to the third decimal. The root means square error of the ESN model for (35–145) back drive SPAs is $1.04 \times 10^{-11}$ and for (60–120) front-drive SPAs is $1.63 \times 10^{-12}$. The compared paths and percentage error in X and Y coordinates are shown in Fig. 10a,b for front and back drive SPAs.

## Discussion

This study is divided into two main parts; the first part is choosing a suitable actuator that can mimic the turtle flippers; the second is to test and model a designed turtle’s motion. From the observation of the soft pneumatic actuator (SPA) motion in 3D. The SPA motion is affected by changing its shape’s geometrical parameters as the air pillow’s height, the width of the air pillow, or the air pillow orientation angle $\theta$, making the SPA move in 3D. How much rotation angle is needed to move the turtle and study the geometrical parameter influences the SPA’s motion in 3D. Twenty-two different SPAs with different $\theta$ angles ($35^{\circ }:5^{\circ }:145^{\circ }$) is designed and analyzed to optimize the turtle actuator’s design. SPA $\theta$ angle was chosen as the $90^0$ is the standard angle of SPA to move in 2D, and then the angle increment and decrement by $5^{\circ }$ reaching $35^{\circ }$ and $145^{\circ }$ as this was the maximum incline that the angle could reach concerning the length of SPA. The designed SPAs are simulated at a wide range of pressure from 0.11 to 0.4 Mpa using finite element analysis to ponder their motion in 3D by analyzing the actuator’s tip coordinates. The first twisting angle in Z coordinates begins at $\theta =60^{\circ }$, and it is complementary $120^{\circ }$, while the maximum rotation occurs at angles $35^{\circ }$ and $145^{\circ }$. To validate the simulation, the 4 SPAs ($\theta =60^{\circ },\, 120^{\circ },\, 35^{\circ } and 145^{\circ }$) had manufactured using the 3D printing technique (FDM) and tested by frame their motion in 3D coordination using tracker software. Comparing both simulation and real data, the two have the same curve behaviour with an error that indicates that the simulation result is comprehending the real one. Still, it was needed more material tests for the FEA model to overcome the gap between coordinates curves. The other reason for this error is the manufacturing method of 3D printing that depends on the layer building and strain energy theory of hyperelastic material. After trials, the layers of SPA were disintegrated due to air pressure, which changes the motion coordinate of the tip of the SPA that can reach.

The second part of the study is to test the designed turtle’s motion and model it. The turtle robot tests were two tests: operating the front-drive SPA60 and SPA120 and the other test used back-drive SPA35 and SPA145. Although the tests show the SPA’s ability to move the turtle forward, each drive type changes the robot’s dynamics. From the turtle’s paths observation, the motion dynamics mean that the turtle’s position depends on SPAs rotation angle that causes the slipping of the robot that happened during the motion. The path of back-drive SPAs is faster with ripples, while front-drive is smoother but slower due to the reliability difference between two complementary angles of the drive SPAs. The dynamics motion was modelled using one of the reservoir computing methods, which is the echo state neural network. The non-linearity that emphases in turtle motion was hard to be modelled with the standard modelling method, so the black box model as the neural network. Neural network performance was insufficient to predict the turtle’s path as the turtle’s motion is complex in its dynamics. The echo state’s preference on the neural network can adapt with chaotic time series and deal with bifurcation that neural networks can agitate. The bifurcation here is the change of the rotation motion angle that the SPA can do during the operating time that made the qualitative changes in robot dynamics. The error reduced in the echo state model overcame the vanishing gradient problem as the dynamic reservoir in its architecture learned how to cope with the oscillators of inputs. The dynamic reservoir converts the state of the oscillator of the input (rotation angle) and predicts the output (robot path coordinate) by the state of these oscillators. The normalization operation is performed on the SPA’s rotation angle by changing angles from degree to rad, which allows the network to drive the loosely coupled oscillator, saving the previous information of past operations. This function is done by the sparse connectivity of a dynamic reservoir. The difference between the proposed turtle and other bio-inspired turtle is shown in Table 2 which shows that using Echo State method in modelling the robot motion allow to consider the dynamics change of soft actuator and it is affect on robot path.On contrast the other work that used rigid actuators,even if the turtle robot with soft actuator focused on actuator model and not the whole robot path and motion.

## Conclusion

This paper aims to utilize the echo state network in modelling the complex dynamic motion of bio-mimic robots as turtle robot with soft flippers. The other focus is to study the ability to use an oriented air pillow SPA to act as a flipper for a turtle robot to mimic its motion on land. Twenty-two SPAs were simulated on FEA with tabular pressure from 0.11Mpa to 0.4Mpa to study their behaviour in 3D. 4 SPAs were chosen for manufacturing: $\theta =60^{\circ },\, 120^{\circ },\, 35^{\circ }$, and $145^{\circ }$ as maximum and minimum rotation angles in 3D. These SPAs are manufactured using FDM printer and tested using vision feedback using tracker software. Comparing the simulation data with real data tested shows the same curve behaviour, however, there was an error margin from (10-18 mm) due to lack of shear and biaxial test on material from the simulation’s beginning and layer adhesive of FDM manufacturing. The turtle designed with four actuators tested its path by using SPA60 and SPA120 as front-drive, and back drive with SPA35 and SPA145. The paths were inclined and had ripples, but it proves the ability to use SPAs to move turtle robot. The front SPAs were steadier in slipping; however, it was slow with velocity 8.5 mm/sec, and the rear-drive was faster with velocity 15.3  mm/s, but there was slipping in turtle path. The robot’s two behaviour is modelled using ESN, which shows small error compared to traditional methods and model the turtle robot with all the uncertainty included in the system. The ESN model square mean error for front-drive was $1.63 \times 10^{-12}$ and $1.04 \times 10^{-11}$ for back-drive. This study’s continuity is to try to use other manufacturing techniques such as stereolithography or selective laser sintering 3d printers. It solves FDM’s layer problem and trains the ESN model to perform the robot’s path planning, including its velocity and acceleration to develop motion and tasks that the robot can do. In order to realize the tortoise robot, the research followed a paradigm. This paradigm can be to create another soft robot inspired by biology. The 3D SPA proposed in this article is an actuator that can be used for crawling mechanisms inspired by snakes, lizards, and crocodiles, and crawling mechanisms may make other designs flourish. By simply changing the necessary motion and motion data, the workflow of modeling the behavior of the actuator and using the echo state method to build a dynamic model of the robot can be used in the same way as the turtle.

**Table 2:** Summary of the Soft actuated turtle robots mentioned in this work.

Ref	Actuator	Actuation force	Motion analysis	Application
^21^	Variable-stiffness morphing limb	Temperature–pressure	–	Underwater and on land robot
^22^	Variable-stiffness morphing limb	Temperature–pressure	Actuator analysis (does not study the whole robot motion)	Underwater and on land robot
^27^	Soft pneumatic actuator (SPA)	Pneumatic	Experiment analysis	Underwater robot
This work	3D Soft pneumatic actuator (SPA)	Pneumatic	Modelling dynamics of robot using echo state method	On land robot

## Supplementary Information

Supplementary Information.
