# Supplementary material for: Modelling and implementation of soft bio-mimetic turtle using echo state network and soft pneumatic actuators
Source: Sci Rep. 2021 Jun 8;11:12076. doi: 10.1038/s41598-021-91136-z (PMC8187634; doi:10.1038/s41598-021-91136-z)
Supplement: Supplementary file 1 — Supplementary Information. [file 41598_2021_91136_MOESM1_ESM.pdf]

Modelling and Implementation of Soft Bio-Mimetic Turtle Using Echo State Network and Soft Pneumatic Actuators (Supplementary material)

MennaAllah Soliman[1,*], Mostafa A. Mousa[2], Mahmood A. Saleh[1], Mahmoud Elsamanty[3,4], Ahmed G. Radwan[5,6]

[1]Mechanical Engineering Program, School of Engineering and Applied Sciences, Nile University,Giza,12588, Egypt.
[2]Nanoelectronics Integrated Systems Center (NISC), Nile University, Giza, 12588, Egypt.
[3]Smart Engineering Systems Research Center (SESC), Nile University, Giza,12588, Egypt.
[4]Benha University, Faculty of Engineering at Shoubra, Mechanical Department, Egypt.
[5]Department of Engineering Mathematics and Physics, Cairo University, Giza 12613, Egypt.
[6]School of Engineering and Applied Sciences, Nile University, Giza, 12588, Egypt.
*msoliman@nu.edu.eg

Table of Contents

Movie S1: 3

Table S1: FEA Results of SPA35 3

Table S2: Experimental Results of SPA35 X Points 4

Table S3: Experimental Results SPA35 Y Points 5

Table S4: Experimental Results SPA35 Z Points 6

Table S5: FEA Results of SPA60 7

Table S6: Experimental Results SPA60 X Points 8

Table S7: Experimental Results SPA60 Y Points 9

Table S8: Experimental Results SPA60 Z Points 10

Table S9: FEA Results of SPA120 11

Table S10: Experimental Results SPA120 X Points 12

Table S11: Experimental Results SPA120 Y Points 13

Table S12: Experimental Results SPA120 Z Points 14

Table S13: FEA Results of SPA145 15

Table S14: Experimental Results SPA145 X Points 16

Table S15: Experimental Results SPA145 Y Points 17

Table S16: Experimental Results SPA145 Z Points 18

Table S17: Turtle movement actuated by front drive SPA60 and SPA120. 19

Table S18: Turtle movement actuated by front drive SPA35 and SPA145. 41

Figure S1: Difference between SPA 35 and SPA145 rotation motion angle distribution for different paths (extension for figure 7 of reliability of SPA during path) 69

Figure S2: Difference between SPA60 and SPA120 Rotation motion angle distribution for different paths (extension for figure 7 of reliability of SPA during path) 70

Figure S3, S4, S5, and S6 Details of robot design and fabrication Figures: - 71

Figure S7: Experimental vs FEA for (a) SPA35, (b) SPA145, (c) SPA60, and (d) SPA120 for Rotation angle 73

Figure S8: FEA for (a) FEA (X, Y, Z) work envelope in 3D, (b) FEA (X, Y) work envelope vs applied pressure, and (c) FEA Z points (rotation in 3D) work envelope vs applied pressure 74

Movie S1:

https://nileuniversity-my.sharepoint.com/:v:/g/personal/mabdullah_nu_edu_eg/EYpY9v8ZAQ5EskRX-uZPw3MBBKPeWpdBoojLJ_WAO9wRAQ?e=QNJWL0

Table S1: FEA Results of SPA35

P MPa	Coordinates			Calculated Angles			
	x points mm	y points mm	z points mm	y/x radian	y/x degree	z/x radian	z/x degree
**0.1**	65.8	0	0	0	0	0	0
**0.11**	65.7847231	-3.990225077	-0.253120214	-0.060581578	-3.471068739	-0.003847686	-0.220456174
**0.12**	65.53443398	-7.77897501	-0.104111604	-0.118147751	-6.769367497	-0.001588654	-0.091023148
**0.13**	65.00518333	-11.29911613	0.399775326	-0.172099234	-9.860559792	0.006149822	0.352358828
**0.14**	64.27519183	-14.51625729	1.203568697	-0.222118973	-12.72647972	0.018723057	1.072752124
**0.15**	63.38753276	-17.41535187	2.253102541	-0.268128305	-15.36262025	0.035529926	2.035714829
**0.16**	62.38039117	-19.99176216	3.497507811	-0.31013967	-17.76969414	0.056008784	3.209066923
**0.17**	61.2841156	-22.25452995	4.894305706	-0.34832989	-19.95783258	0.079693408	4.566095914
**0.18**	60.12770109	-24.209795	6.402171612	-0.382779842	-21.93166945	0.106076576	6.077740111
**0.19**	58.93442945	-25.87143326	7.986913204	-0.41365765	-23.70083748	0.134701374	7.717820197
**0.2**	57.72415428	-27.25520515	9.618947983	-0.441130989	-25.27494387	0.165119274	9.460637544
**0.21**	56.51170139	-28.38013077	11.27552795	-0.465405414	-26.665766	0.19693931	11.28379131
**0.22**	55.3119257	-29.26247978	12.93431282	-0.486612462	-27.88084032	0.229715336	13.16171922
**0.23**	54.13530712	-29.92070389	14.57903957	-0.504915477	-28.92952583	0.263066195	15.07258271
**0.24**	52.99083881	-30.3721981	16.19576836	-0.52044993	-29.81958445	0.296616972	16.99490064
**0.25**	51.88600807	-30.63363647	17.77285957	-0.533332734	-30.55771476	0.330010548	18.90821162
**0.26**	50.8272646	-30.72099686	19.3003521	-0.543662898	-31.14958956	0.362906155	20.79299105
**0.27**	49.82037811	-30.64985085	20.76950073	-0.551526224	-31.60012492	0.394979405	22.6306529
**0.28**	48.87076454	-30.43578911	22.17241287	-0.557002119	-31.91387062	0.425922292	24.40354972
**0.29**	47.98364716	-30.09483147	23.50189018	-0.560172115	-32.09549802	0.455445956	26.09513106
**0.3**	47.1637085	-29.64356232	24.75172615	-0.561130017	-32.15038173	0.483293726	27.69069076
**0.31**	46.41546707	-29.09909058	25.91568565	-0.55998353	-32.08469288	0.509224936	29.17643968
**0.32**	45.74254875	-28.47907257	26.98794937	-0.556867857	-31.90617794	0.533031606	30.5404614
**0.33**	45.14792709	-27.8026638	27.96249771	-0.551965373	-31.6252863	0.554528139	31.77212201
**0.34**	44.6335495	-27.09106064	28.83427238	-0.545526045	-31.25633998	0.573573927	32.86336523
**0.35**	44.1998661	-26.36736298	29.60063934	-0.537877754	-30.81812517	0.590099493	33.81021044
**0.36**	43.84535103	-25.65494919	30.2627182	-0.529409143	-30.33290953	0.604128606	34.61401941
**0.37**	43.56580811	-24.97461128	30.82632065	-0.520526956	-29.82399768	0.615795517	35.28248419
**0.38**	43.3552597	-24.3426857	31.30031967	-0.511606824	-29.31291176	0.62530596	35.8273924
**0.39**	43.20520096	-23.76865005	31.69681168	-0.502946085	-28.81668798	0.632944437	36.26504489
**0.4**	43.10690956	-23.2564888	32.02761459	-0.494751656	-28.34718177	0.638993791	36.61164735

Table S2: Experimental Results of SPA35 X Points

P(MPa)	T1	T2	T3	T4	T5	T6	T7	T8	T9	T10
0.10	**65.41**	**65.58**	**65.80**	**65.80**	**65.76**	**65.75**	**65.80**	**65.80**	**65.80**	**65.80**
0.11	**65.34**	**65.38**	**65.34**	**65.38**	**64.98**	**64.73**	**65.78**	**66.06**	**65.31**	**65.84**
0.12	**64.66**	**65.31**	**65.29**	**65.38**	**63.56**	**62.44**	**65.57**	**65.47**	**65.09**	**65.78**
0.13	**63.99**	**65.24**	**65.24**	**65.38**	**62.96**	**60.43**	**65.35**	**64.88**	**64.87**	**65.73**
0.14	**64.56**	**64.31**	**65.19**	**65.38**	**61.90**	**60.14**	**65.13**	**64.28**	**64.66**	**65.67**
0.15	**62.57**	**63.96**	**65.15**	**65.38**	**60.51**	**60.58**	**64.91**	**63.69**	**64.44**	**65.61**
0.16	**61.44**	**64.20**	**65.10**	**65.38**	**60.94**	**60.59**	**62.56**	**62.53**	**61.88**	**63.92**
0.17	**60.87**	**63.36**	**65.08**	**65.38**	**60.64**	**60.55**	**60.21**	**61.38**	**59.33**	**62.23**
0.18	**60.30**	**62.53**	**65.06**	**65.38**	**60.33**	**60.51**	**62.79**	**62.97**	**61.20**	**63.58**
0.19	**59.74**	**61.69**	**65.04**	**65.38**	**60.03**	**60.47**	**65.36**	**64.56**	**63.07**	**64.94**
0.20	**59.74**	**62.31**	**65.02**	**65.38**	**59.73**	**60.43**	**66.01**	**61.23**	**64.94**	**66.29**
0.21	**59.74**	**62.93**	**64.99**	**65.38**	**59.02**	**61.74**	**66.66**	**57.90**	**60.87**	**63.36**
0.22	**59.74**	**63.55**	**62.37**	**63.92**	**55.52**	**59.64**	**66.21**	**57.72**	**56.81**	**60.43**
0.23	**54.63**	**62.92**	**59.75**	**62.46**	**55.81**	**60.23**	**65.77**	**57.55**	**57.21**	**60.77**
0.24	**55.48**	**60.17**	**59.00**	**62.12**	**54.33**	**58.86**	**65.32**	**57.37**	**57.61**	**61.11**
0.25	**54.35**	**60.92**	**58.25**	**61.79**	**54.26**	**57.75**	**64.88**	**57.20**	**58.02**	**61.44**
0.26	**54.35**	**59.68**	**57.50**	**61.45**	**53.28**	**56.98**	**64.44**	**57.02**	**58.42**	**61.78**
0.27	**53.78**	**62.36**	**56.75**	**61.11**	**51.18**	**56.49**	**63.99**	**56.85**	**53.88**	**57.95**
0.28	**54.50**	**57.97**	**54.50**	**57.97**	**50.79**	**56.34**	**59.17**	**60.50**	**53.74**	**57.46**
0.29	**54.09**	**57.41**	**54.09**	**57.41**	**50.78**	**55.41**	**50.78**	**55.41**	**53.60**	**56.96**
0.30	**53.69**	**56.84**	**53.69**	**56.84**	**50.99**	**54.44**	**50.99**	**54.44**	**53.47**	**56.46**
0.31	**53.28**	**56.28**	**53.28**	**56.28**	**51.19**	**53.47**	**51.19**	**53.47**	**53.33**	**55.97**
0.32	**52.88**	**55.72**	**52.88**	**55.72**	**51.40**	**52.50**	**51.40**	**52.50**	**53.19**	**55.47**
0.33	**52.47**	**55.16**	**52.47**	**55.16**	**51.61**	**51.53**	**51.61**	**51.53**	**52.09**	**53.44**
0.34	**52.06**	**54.60**	**52.06**	**54.60**	**51.82**	**50.56**	**51.82**	**50.56**	**50.99**	**51.42**
0.35	**56.40**	**55.95**	**56.40**	**55.95**	**51.80**	**49.04**	**51.80**	**49.04**	**53.70**	**52.99**
0.36	**54.74**	**55.16**	**54.74**	**55.16**	**58.48**	**52.75**	**58.48**	**52.75**	**47.42**	**48.94**
0.37	**53.09**	**54.37**	**53.09**	**54.37**	**54.82**	**51.08**	**54.82**	**51.08**	**45.95**	**48.99**
0.38	**51.43**	**53.59**	**51.43**	**53.59**	**51.17**	**49.42**	**51.17**	**49.42**	**44.47**	**49.05**
0.39	**49.78**	**52.80**	**49.78**	**52.80**	**47.51**	**47.75**	**47.51**	**47.75**	**42.99**	**49.11**
0.40	**46.36**	**48.76**	**46.36**	**48.76**	**43.85**	**46.08**	**43.85**	**46.08**	**41.52**	**49.16**

Table S3: Experimental Results SPA35 Y Points

P(MPa)	T1	T2	T3	T4	T5	T6	T7	T8	T9	T10
0.10	**-5.55**	**-5.55**	**0.09**	**0.09**	**0.09**	**0.09**	**0.09**	**0.09**	**0.09**	**0.09**
0.11	**-9.90**	**-9.90**	**0.09**	**0.09**	**-3.56**	**-3.56**	**0.19**	**0.19**	**-0.40**	**-0.40**
0.12	**-14.24**	**-14.24**	**-1.76**	**-1.76**	**-4.94**	**-4.94**	**-2.40**	**-2.40**	**-2.83**	**-2.83**
0.13	**-18.59**	**-18.59**	**-3.62**	**-3.62**	**-8.02**	**-8.02**	**-4.98**	**-4.98**	**-5.25**	**-5.25**
0.14	**-13.77**	**-13.77**	**-5.47**	**-5.47**	**-10.60**	**-10.60**	**-7.56**	**-7.56**	**-7.68**	**-7.68**
0.15	**-20.58**	**-20.58**	**-7.33**	**-7.33**	**-12.19**	**-12.19**	**-10.15**	**-10.15**	**-10.11**	**-10.11**
0.16	**-21.99**	**-21.99**	**-9.18**	**-9.18**	**-14.66**	**-14.66**	**-15.82**	**-15.82**	**-15.53**	**-15.53**
0.17	**-23.03**	**-23.03**	**-8.83**	**-8.83**	**-14.81**	**-14.81**	**-21.49**	**-21.49**	**-20.95**	**-20.95**
0.18	**-24.07**	**-24.07**	**-8.48**	**-8.48**	**-14.95**	**-14.95**	**-15.43**	**-15.43**	**-16.71**	**-16.71**
0.19	**-25.11**	**-25.11**	**-8.12**	**-8.12**	**-15.10**	**-15.10**	**-9.36**	**-9.36**	**-12.46**	**-12.46**
0.20	**-25.68**	**-25.68**	**-7.77**	**-7.77**	**-15.25**	**-15.25**	**-17.97**	**-17.97**	**-8.22**	**-8.22**
0.21	**-26.25**	**-26.25**	**-7.42**	**-7.42**	**-15.80**	**-15.80**	**-26.58**	**-26.58**	**-15.74**	**-15.74**
0.22	**-26.81**	**-26.81**	**-14.93**	**-14.93**	**-19.29**	**-19.29**	**-27.21**	**-27.21**	**-23.27**	**-23.27**
0.23	**-30.21**	**-30.21**	**-22.45**	**-22.45**	**-19.77**	**-19.77**	**-27.84**	**-27.84**	**-22.71**	**-22.71**
0.24	**-30.78**	**-30.78**	**-23.15**	**-23.15**	**-20.12**	**-20.12**	**-28.47**	**-28.47**	**-22.15**	**-22.15**
0.25	**-32.48**	**-32.48**	**-23.86**	**-23.86**	**-22.63**	**-22.63**	**-29.10**	**-29.10**	**-21.60**	**-21.60**
0.26	**-31.91**	**-31.91**	**-24.56**	**-24.56**	**-22.95**	**-22.95**	**-29.73**	**-29.73**	**-21.04**	**-21.04**
0.27	**-32.77**	**-32.77**	**-25.26**	**-25.26**	**-24.01**	**-24.01**	**-30.36**	**-30.36**	**-26.09**	**-26.09**
0.28	**-28.74**	**-28.74**	**-28.74**	**-28.74**	**-24.95**	**-24.95**	**-21.97**	**-21.97**	**-26.56**	**-26.56**
0.29	**-30.28**	**-30.28**	**-30.28**	**-30.28**	**-26.74**	**-26.74**	**-26.74**	**-26.74**	**-27.03**	**-27.03**
0.30	**-31.83**	**-31.83**	**-31.83**	**-31.83**	**-27.89**	**-27.89**	**-27.89**	**-27.89**	**-27.50**	**-27.50**
0.31	**-33.38**	**-33.38**	**-33.38**	**-33.38**	**-29.05**	**-29.05**	**-29.05**	**-29.05**	**-27.98**	**-27.98**
0.32	**-34.92**	**-34.92**	**-34.92**	**-34.92**	**-30.21**	**-30.21**	**-30.21**	**-30.21**	**-28.45**	**-28.45**
0.33	**-36.47**	**-36.47**	**-36.47**	**-36.47**	**-31.37**	**-31.37**	**-31.37**	**-31.37**	**-31.20**	**-31.20**
0.34	**-38.02**	**-38.02**	**-38.02**	**-38.02**	**-32.53**	**-32.53**	**-32.53**	**-32.53**	**-33.95**	**-33.95**
0.35	**-37.06**	**-37.06**	**-37.06**	**-37.06**	**-35.45**	**-35.45**	**-35.45**	**-35.45**	**-33.45**	**-33.45**
0.36	**-37.29**	**-37.29**	**-37.29**	**-37.29**	**-33.20**	**-33.20**	**-33.20**	**-33.20**	**-35.00**	**-35.00**
0.37	**-37.52**	**-37.52**	**-37.52**	**-37.52**	**-33.59**	**-33.59**	**-33.59**	**-33.59**	**-35.14**	**-35.14**
0.38	**-37.75**	**-37.75**	**-37.75**	**-37.75**	**-33.97**	**-33.97**	**-33.97**	**-33.97**	**-35.29**	**-35.29**
0.39	**-37.98**	**-37.98**	**-37.98**	**-37.98**	**-34.36**	**-34.36**	**-34.36**	**-34.36**	**-35.44**	**-35.44**
0.40	**-39.59**	**-39.59**	**-39.59**	**-39.59**	**-34.74**	**-34.74**	**-34.74**	**-34.74**	**-35.59**	**-35.59**

Table S4: Experimental Results SPA35 Z Points

P(MPa)	T1	T2	T3	T4	T5	T6	T7	T8	T9	T10
0.10	**0.12**	**0.12**	**0.12**	**0.12**	**0.11**	**0.11**	**0.11**	**0.11**	**0.11**	**0.11**
0.11	**0.64**	**0.64**	**0.73**	**0.73**	**1.13**	**1.13**	**-0.03**	**-0.03**	**-0.38**	**-0.38**
0.12	**1.15**	**1.15**	**1.36**	**1.36**	**1.40**	**1.40**	**1.03**	**1.03**	**0.86**	**0.86**
0.13	**1.66**	**1.66**	**1.98**	**1.98**	**1.83**	**1.83**	**2.08**	**2.08**	**2.10**	**2.10**
0.14	**1.95**	**1.95**	**2.61**	**2.61**	**3.03**	**3.03**	**3.14**	**3.14**	**3.34**	**3.34**
0.15	**3.17**	**3.17**	**3.24**	**3.24**	**3.95**	**3.95**	**4.20**	**4.20**	**4.58**	**4.58**
0.16	**5.65**	**5.65**	**3.87**	**3.87**	**3.19**	**3.19**	**8.30**	**8.30**	**7.62**	**7.62**
0.17	**6.04**	**6.04**	**3.78**	**3.78**	**3.45**	**3.45**	**12.40**	**12.40**	**10.66**	**10.66**
0.18	**6.43**	**6.43**	**3.69**	**3.69**	**3.72**	**3.72**	**7.96**	**7.96**	**7.81**	**7.81**
0.19	**6.82**	**6.82**	**3.60**	**3.60**	**3.98**	**3.98**	**3.52**	**3.52**	**4.95**	**4.95**
0.20	**6.84**	**6.84**	**3.51**	**3.51**	**4.25**	**4.25**	**9.75**	**9.75**	**2.10**	**2.10**
0.21	**6.85**	**6.85**	**3.42**	**3.42**	**5.26**	**5.26**	**15.98**	**15.98**	**8.07**	**8.07**
0.22	**6.87**	**6.87**	**7.24**	**7.24**	**6.06**	**6.06**	**16.65**	**16.65**	**14.04**	**14.04**
0.23	**10.97**	**10.97**	**11.06**	**11.06**	**6.83**	**6.83**	**17.31**	**17.31**	**13.42**	**13.42**
0.24	**11.67**	**11.67**	**12.07**	**12.07**	**7.56**	**7.56**	**17.98**	**17.98**	**12.80**	**12.80**
0.25	**12.86**	**12.86**	**13.08**	**13.08**	**10.12**	**10.12**	**18.64**	**18.64**	**12.18**	**12.18**
0.26	**15.17**	**15.17**	**14.09**	**14.09**	**10.72**	**10.72**	**19.31**	**19.31**	**11.56**	**11.56**
0.27	**16.80**	**16.80**	**15.10**	**15.10**	**13.29**	**13.29**	**19.97**	**19.97**	**16.97**	**16.97**
0.28	**18.69**	**18.69**	**18.69**	**18.69**	**13.29**	**13.29**	**12.63**	**12.63**	**18.01**	**18.01**
0.29	**19.74**	**19.74**	**19.74**	**19.74**	**14.18**	**14.18**	**14.18**	**14.18**	**19.05**	**19.05**
0.30	**20.79**	**20.79**	**20.79**	**20.79**	**16.95**	**16.95**	**16.95**	**16.95**	**20.08**	**20.08**
0.31	**21.83**	**21.83**	**21.83**	**21.83**	**19.71**	**19.71**	**19.71**	**19.71**	**21.12**	**21.12**
0.32	**22.88**	**22.88**	**22.88**	**22.88**	**22.48**	**22.48**	**22.48**	**22.48**	**22.16**	**22.16**
0.33	**23.93**	**23.93**	**23.93**	**23.93**	**25.24**	**25.24**	**25.24**	**25.24**	**24.97**	**24.97**
0.34	**24.98**	**24.98**	**24.98**	**24.98**	**28.01**	**28.01**	**28.01**	**28.01**	**27.79**	**27.79**
0.35	**22.51**	**22.51**	**22.51**	**22.51**	**30.01**	**30.01**	**30.01**	**30.01**	**24.86**	**24.86**
0.36	**23.63**	**23.63**	**23.63**	**23.63**	**26.65**	**26.65**	**26.65**	**26.65**	**30.72**	**30.72**
0.37	**24.75**	**24.75**	**24.75**	**24.75**	**29.77**	**29.77**	**29.77**	**29.77**	**31.96**	**31.96**
0.38	**25.88**	**25.88**	**25.88**	**25.88**	**32.88**	**32.88**	**32.88**	**32.88**	**33.20**	**33.20**
0.39	**27.00**	**27.00**	**27.00**	**27.00**	**35.99**	**35.99**	**35.99**	**35.99**	**34.44**	**34.44**
0.40	**31.72**	**31.72**	**31.72**	**31.72**	**39.11**	**39.11**	**39.11**	**39.11**	**35.68**	**35.68**

Table S5: FEA Results of SPA60

P MP	Coordinates			Calculated Angles			
	**x points mm**	**y points mm**	**z points mm**	**y/x radian**	**y/x degree**	**z/x radian**	**z/x degree**
**0.1**	63.211	0.000	0.000	0.000000	0.000000	0.000000	0.000000
**0.11**	63.207	-3.038	-0.089	-0.048032	-2.752025	-0.001409	-0.080720
**0.12**	63.035	-5.865	-0.078	-0.092772	-5.315421	-0.001244	-0.071255
**0.13**	62.727	-8.496	0.019	-0.134621	-7.713202	0.000307	0.017610
**0.14**	62.310	-10.950	0.192	-0.173964	-9.967406	0.003083	0.176618
**0.15**	61.802	-13.245	0.429	-0.211113	-12.095866	0.006949	0.398170
**0.16**	61.219	-15.396	0.723	-0.246377	-14.116374	0.011813	0.676828
**0.17**	60.573	-17.410	1.065	-0.279882	-16.036036	0.017574	1.006891
**0.18**	59.874	-19.302	1.448	-0.311861	-17.868310	0.024174	1.385069
**0.19**	59.131	-21.078	1.866	-0.342420	-19.619200	0.031550	1.807663
**0.2**	58.351	-22.745	2.315	-0.371679	-21.295623	0.039653	2.271921
**0.21**	57.540	-24.311	2.790	-0.399761	-22.904606	0.048445	2.775716
**0.22**	56.703	-25.781	3.286	-0.426731	-24.449901	0.057890	3.316826
**0.23**	55.844	-27.160	3.801	-0.452671	-25.936137	0.067955	3.893511
**0.24**	54.968	-28.455	4.331	-0.477680	-27.369073	0.078628	4.505040
**0.25**	54.079	-29.668	4.873	-0.501776	-28.749627	0.089868	5.149059
**0.26**	53.177	-30.806	5.426	-0.525068	-30.084201	0.101684	5.826054
**0.27**	52.266	-31.871	5.987	-0.547587	-31.374439	0.114047	6.534413
**0.28**	51.350	-32.868	6.554	-0.569370	-32.622514	0.126942	7.273251
**0.29**	50.428	-33.799	7.125	-0.590470	-33.831462	0.140363	8.042189
**0.3**	49.504	-34.668	7.700	-0.610931	-35.003793	0.154302	8.840847
**0.31**	48.578	-35.477	8.276	-0.630788	-36.141487	0.168751	9.668713
**0.32**	47.652	-36.231	8.854	-0.650076	-37.246603	0.183704	10.525483
**0.33**	46.726	-36.930	9.431	-0.668831	-38.321174	0.199160	11.411002
**0.34**	45.803	-37.578	10.007	-0.687071	-39.366295	0.215105	12.324624
**0.35**	44.883	-38.177	10.582	-0.704838	-40.384242	0.231545	13.266526
**0.36**	43.966	-38.728	11.154	-0.722144	-41.375789	0.248465	14.235984
**0.37**	43.053	-39.235	11.724	-0.739024	-42.342981	0.265872	15.233353
**0.38**	42.146	-39.697	12.291	-0.755490	-43.286375	0.283751	16.257708
**0.39**	41.244	-40.118	12.853	-0.771564	-44.207356	0.302098	17.308930
**0.4**	40.349	-40.499	13.411	-0.787256	-45.106461	0.320899	18.386134

Table S6: Experimental Results SPA60 X Points

P (MPa)	T1	T2	T3	T4	T5	T6	T7	T8	T9	T10	T11	T12	T13	T14	T15	T16
0.10	**63.1**	**62.3**	**63.0**	**63.0**	**63.4**	**64.0**	**62.4**	**63.5**	**63.2**	**62.6**	**63.4**	**63.3**	**63.0**	**63.1**	**63.7**	**63.4**
0.11	**62.6**	**62.2**	**63.0**	**63.4**	**62.3**	**64.1**	**62.1**	**63.9**	**62.9**	**62.2**	**63.3**	**63.2**	**62.7**	**63.2**	**63.2**	**63.2**
0.12	**62.1**	**62.2**	**63.0**	**63.9**	**61.2**	**64.3**	**61.8**	**64.2**	**62.5**	**61.7**	**63.2**	**63.2**	**62.4**	**63.3**	**62.6**	**63.1**
0.13	**61.8**	**62.2**	**61.7**	**64.2**	**60.1**	**64.5**	**61.5**	**64.6**	**62.1**	**61.2**	**63.0**	**63.1**	**62.1**	**63.3**	**62.0**	**62.9**
0.14	**61.6**	**62.1**	**61.4**	**64.4**	**62.0**	**63.9**	**61.4**	**64.8**	**62.0**	**61.4**	**62.4**	**62.5**	**61.8**	**63.4**	**62.5**	**63.0**
0.15	**60.7**	**61.9**	**61.2**	**64.6**	**61.5**	**64.0**	**61.3**	**65.0**	**61.8**	**61.6**	**61.8**	**62.0**	**61.5**	**63.4**	**63.0**	**63.1**
0.16	**60.4**	**61.8**	**60.6**	**65.5**	**61.0**	**64.0**	**61.2**	**65.2**	**61.7**	**61.8**	**62.8**	**62.7**	**61.3**	**63.5**	**63.5**	**63.3**
0.17	**60.0**	**61.8**	**58.0**	**64.8**	**60.5**	**64.0**	**61.1**	**65.4**	**61.5**	**61.9**	**63.8**	**63.4**	**62.6**	**62.8**	**64.0**	**63.4**
0.18	**59.7**	**61.7**	**57.9**	**64.8**	**60.0**	**64.0**	**61.0**	**65.6**	**61.4**	**62.1**	**63.1**	**63.0**	**62.1**	**62.8**	**63.0**	**62.9**
0.19	**59.5**	**61.8**	**57.7**	**64.7**	**59.1**	**64.3**	**60.5**	**64.1**	**61.3**	**62.4**	**62.5**	**62.6**	**61.6**	**62.8**	**62.5**	**63.0**
0.20	**59.3**	**62.0**	**56.5**	**65.0**	**58.1**	**64.5**	**60.2**	**64.1**	**61.0**	**62.0**	**61.8**	**62.2**	**61.1**	**62.8**	**62.0**	**63.1**
0.21	**59.1**	**62.1**	**56.4**	**64.8**	**57.7**	**64.4**	**59.8**	**64.1**	**60.7**	**61.6**	**61.3**	**62.0**	**60.6**	**62.8**	**61.1**	**62.8**
0.22	**58.9**	**62.2**	**56.3**	**64.6**	**57.2**	**64.3**	**59.4**	**64.1**	**60.3**	**61.3**	**60.5**	**61.6**	**59.4**	**62.8**	**60.3**	**62.5**
0.23	**58.6**	**62.4**	**56.2**	**64.4**	**56.8**	**64.3**	**58.2**	**63.3**	**59.6**	**61.0**	**59.8**	**61.2**	**58.8**	**62.6**	**59.4**	**62.1**
0.24	**58.4**	**62.5**	**56.1**	**64.2**	**56.3**	**64.2**	**58.1**	**63.0**	**58.9**	**60.8**	**59.1**	**60.8**	**58.2**	**62.4**	**61.0**	**62.1**
0.25	**58.2**	**62.6**	**55.9**	**64.1**	**55.9**	**64.1**	**58.0**	**62.6**	**58.4**	**60.8**	**58.4**	**60.4**	**57.8**	**62.0**	**62.5**	**62.1**
0.26	**58.0**	**62.8**	**55.8**	**63.9**	**56.2**	**64.1**	**57.2**	**62.9**	**57.8**	**60.7**	**61.0**	**61.1**	**57.5**	**61.6**	**58.5**	**61.4**
0.27	**57.3**	**62.8**	**54.9**	**63.8**	**56.5**	**64.2**	**56.4**	**63.1**	**57.1**	**59.8**	**62.3**	**61.9**	**57.1**	**61.2**	**56.1**	**60.1**
0.28	**56.9**	**62.0**	**54.0**	**63.8**	**56.8**	**63.2**	**55.6**	**63.4**	**56.4**	**58.8**	**59.2**	**60.2**	**59.5**	**61.9**	**55.7**	**59.9**
0.29	**55.4**	**61.7**	**51.9**	**62.6**	**50.9**	**62.7**	**54.9**	**62.9**	**55.3**	**58.8**	**56.0**	**58.6**	**61.8**	**62.6**	**55.4**	**59.6**
0.30	**56.3**	**61.7**	**52.9**	**62.8**	**50.3**	**62.1**	**54.3**	**62.6**	**59.3**	**61.0**	**54.9**	**58.4**	**55.9**	**59.3**	**55.0**	**59.4**
0.31	**54.7**	**60.5**	**51.0**	**61.8**	**49.7**	**61.5**	**53.8**	**62.3**	**56.9**	**58.9**	**59.4**	**61.1**	**54.8**	**59.0**	**58.7**	**61.6**
0.32	**54.0**	**59.2**	**49.0**	**60.7**	**49.1**	**60.9**	**53.2**	**62.0**	**54.4**	**56.9**	**57.2**	**60.0**	**53.7**	**58.7**	**55.3**	**59.5**
0.33	**53.4**	**58.0**	**54.0**	**63.1**	**53.8**	**63.0**	**57.1**	**63.8**	**55.6**	**58.5**	**53.0**	**57.9**	**52.8**	**57.5**	**51.9**	**57.3**
0.34	**52.8**	**56.7**	**51.3**	**62.0**	**51.3**	**61.6**	**53.6**	**61.5**	**56.8**	**60.1**	**52.4**	**56.5**	**57.1**	**60.7**	**50.8**	**56.3**
0.35	**52.2**	**55.5**	**48.7**	**60.9**	**48.9**	**60.3**	**49.8**	**59.9**	**50.7**	**55.6**	**51.8**	**55.1**	**49.8**	**56.1**	**49.6**	**55.3**
0.36	**51.5**	**54.3**	**46.0**	**59.8**	**46.4**	**58.9**	**51.5**	**61.0**	**53.5**	**57.5**	**56.4**	**59.7**	**50.1**	**56.6**	**52.9**	**57.6**
0.37	**50.5**	**53.0**	**50.7**	**61.8**	**50.3**	**61.1**	**53.2**	**62.1**	**53.9**	**57.5**	**53.5**	**57.5**	**50.5**	**57.0**	**56.1**	**59.9**
0.38	**49.4**	**51.8**	**46.4**	**59.5**	**48.9**	**61.1**	**54.9**	**63.1**	**54.3**	**57.5**	**54.1**	**57.9**	**50.8**	**57.5**	**52.0**	**57.0**
0.39	**48.9**	**50.6**	**42.0**	**57.2**	**44.0**	**57.2**	**50.3**	**60.2**	**51.6**	**55.3**	**54.8**	**58.4**	**53.9**	**59.1**	**48.0**	**54.2**
0.40	**48.4**	**49.3**	**47.6**	**60.8**	**45.4**	**57.9**	**45.7**	**57.3**	**48.9**	**53.0**	**50.8**	**55.2**	**47.3**	**53.3**	**53.8**	**58.1**

Table S7: Experimental Results SPA60 Y Points

P	T1	T2	T3	T4	T5	T6	T7	T8	T9	T10	T11	T12	T13	T14	T15	T16
0.10	**-0.4**	**-0.4**	**-4.1**	**-4.1**	**0.5**	**0.5**	**0.2**	**0.2**	**0.4**	**0.4**	**0.4**	**0.4**	**0.4**	**0.4**	**-0.1**	**-0.1**
0.11	**-3.1**	**-3.1**	**-5.0**	**-5.0**	**-2.9**	**-2.9**	**-1.5**	**-1.5**	**-1.3**	**-1.3**	**-1.4**	**-1.4**	**-1.7**	**-1.7**	**-3.6**	**-3.6**
0.12	**-5.8**	**-5.8**	**-6.0**	**-6.0**	**-6.2**	**-6.2**	**-3.1**	**-3.1**	**-3.0**	**-3.0**	**-3.3**	**-3.3**	**-3.9**	**-3.9**	**-7.1**	**-7.1**
0.13	**-7.2**	**-7.2**	**-8.9**	**-8.9**	**-9.6**	**-9.6**	**-4.8**	**-4.8**	**-4.7**	**-4.7**	**-5.1**	**-5.1**	**-6.0**	**-6.0**	**-10.6**	**-10.6**
0.14	**-8.6**	**-8.6**	**-10.7**	**-10.7**	**-3.9**	**-3.9**	**-5.7**	**-5.7**	**-5.9**	**-5.9**	**-7.9**	**-7.9**	**-8.2**	**-8.2**	**-8.8**	**-8.8**
0.15	**-10.2**	**-10.2**	**-12.4**	**-12.4**	**-5.8**	**-5.8**	**-6.5**	**-6.5**	**-7.2**	**-7.2**	**-10.8**	**-10.8**	**-10.3**	**-10.3**	**-7.0**	**-7.0**
0.16	**-12.0**	**-12.0**	**-14.5**	**-14.5**	**-7.6**	**-7.6**	**-7.4**	**-7.4**	**-8.4**	**-8.4**	**-6.9**	**-6.9**	**-12.4**	**-12.4**	**-5.2**	**-5.2**
0.17	**-13.8**	**-13.8**	**-17.0**	**-17.0**	**-9.5**	**-9.5**	**-8.3**	**-8.3**	**-9.6**	**-9.6**	**-3.1**	**-3.1**	**-4.4**	**-4.4**	**-3.4**	**-3.4**
0.18	**-15.7**	**-15.7**	**-18.8**	**-18.8**	**-11.4**	**-11.4**	**-9.2**	**-9.2**	**-10.9**	**-10.9**	**-6.3**	**-6.3**	**-7.1**	**-7.1**	**-10.0**	**-10.0**
0.19	**-16.0**	**-16.0**	**-20.6**	**-20.6**	**-12.8**	**-12.8**	**-12.6**	**-12.6**	**-13.7**	**-13.7**	**-9.5**	**-9.5**	**-9.8**	**-9.8**	**-11.7**	**-11.7**
0.20	**-16.3**	**-16.3**	**-23.2**	**-23.2**	**-14.3**	**-14.3**	**-13.3**	**-13.3**	**-14.4**	**-14.4**	**-12.8**	**-12.8**	**-12.4**	**-12.4**	**-13.3**	**-13.3**
0.21	**-16.6**	**-16.6**	**-23.4**	**-23.4**	**-15.4**	**-15.4**	**-14.0**	**-14.0**	**-15.0**	**-15.0**	**-15.1**	**-15.1**	**-15.1**	**-15.1**	**-14.9**	**-14.9**
0.22	**-16.9**	**-16.9**	**-23.7**	**-23.7**	**-16.5**	**-16.5**	**-14.7**	**-14.7**	**-15.7**	**-15.7**	**-16.6**	**-16.6**	**-16.5**	**-16.5**	**-16.6**	**-16.6**
0.23	**-17.2**	**-17.2**	**-23.9**	**-23.9**	**-17.6**	**-17.6**	**-18.2**	**-18.2**	**-17.6**	**-17.6**	**-18.1**	**-18.1**	**-17.5**	**-17.5**	**-18.3**	**-18.3**
0.24	**-17.5**	**-17.5**	**-24.2**	**-24.2**	**-18.8**	**-18.8**	**-18.8**	**-18.8**	**-19.5**	**-19.5**	**-19.6**	**-19.6**	**-18.5**	**-18.5**	**-14.3**	**-14.3**
0.25	**-17.9**	**-17.9**	**-24.4**	**-24.4**	**-19.9**	**-19.9**	**-19.5**	**-19.5**	**-19.9**	**-19.9**	**-21.1**	**-21.1**	**-19.6**	**-19.6**	**-10.3**	**-10.3**
0.26	**-18.2**	**-18.2**	**-24.7**	**-24.7**	**-18.2**	**-18.2**	**-20.6**	**-20.6**	**-20.4**	**-20.4**	**-17.8**	**-17.8**	**-20.6**	**-20.6**	**-21.7**	**-21.7**
0.27	**-20.2**	**-20.2**	**-24.9**	**-24.9**	**-16.6**	**-16.6**	**-21.7**	**-21.7**	**-22.3**	**-22.3**	**-10.2**	**-10.2**	**-21.7**	**-21.7**	**-25.1**	**-25.1**
0.28	**-21.9**	**-21.9**	**-25.2**	**-25.2**	**-18.9**	**-18.9**	**-22.8**	**-22.8**	**-24.1**	**-24.1**	**-17.7**	**-17.7**	**-17.2**	**-17.2**	**-25.6**	**-25.6**
0.29	**-22.1**	**-22.1**	**-30.0**	**-30.0**	**-24.6**	**-24.6**	**-23.9**	**-23.9**	**-24.8**	**-24.8**	**-25.1**	**-25.1**	**-12.7**	**-12.7**	**-26.2**	**-26.2**
0.30	**-24.0**	**-24.0**	**-28.4**	**-28.4**	**-25.6**	**-25.6**	**-25.2**	**-25.2**	**-17.8**	**-17.8**	**-26.7**	**-26.7**	**-23.7**	**-23.7**	**-26.8**	**-26.8**
0.31	**-26.6**	**-26.6**	**-30.6**	**-30.6**	**-26.7**	**-26.7**	**-26.5**	**-26.5**	**-22.7**	**-22.7**	**-19.2**	**-19.2**	**-25.2**	**-25.2**	**-21.0**	**-21.0**
0.32	**-27.8**	**-27.8**	**-32.7**	**-32.7**	**-27.8**	**-27.8**	**-27.7**	**-27.7**	**-27.6**	**-27.6**	**-22.2**	**-22.2**	**-26.7**	**-26.7**	**-25.3**	**-25.3**
0.33	**-28.9**	**-28.9**	**-27.2**	**-27.2**	**-22.5**	**-22.5**	**-20.5**	**-20.5**	**-24.6**	**-24.6**	**-27.6**	**-27.6**	**-28.1**	**-28.1**	**-29.5**	**-29.5**
0.34	**-30.0**	**-30.0**	**-29.7**	**-29.7**	**-25.2**	**-25.2**	**-26.0**	**-26.0**	**-21.6**	**-21.6**	**-28.7**	**-28.7**	**-20.9**	**-20.9**	**-30.5**	**-30.5**
0.35	**-31.1**	**-31.1**	**-32.2**	**-32.2**	**-28.0**	**-28.0**	**-29.9**	**-29.9**	**-31.2**	**-31.2**	**-29.8**	**-29.8**	**-30.4**	**-30.4**	**-31.4**	**-31.4**
0.36	**-32.5**	**-32.5**	**-34.7**	**-34.7**	**-30.7**	**-30.7**	**-28.5**	**-28.5**	**-27.4**	**-27.4**	**-23.6**	**-23.6**	**-30.4**	**-30.4**	**-28.2**	**-28.2**
0.37	**-33.0**	**-33.0**	**-31.4**	**-31.4**	**-26.8**	**-26.8**	**-27.2**	**-27.2**	**-26.8**	**-26.8**	**-27.6**	**-27.6**	**-30.4**	**-30.4**	**-24.9**	**-24.9**
0.38	**-33.4**	**-33.4**	**-34.1**	**-34.1**	**-27.8**	**-27.8**	**-25.8**	**-25.8**	**-26.2**	**-26.2**	**-27.5**	**-27.5**	**-30.4**	**-30.4**	**-29.3**	**-29.3**
0.39	**-33.8**	**-33.8**	**-36.8**	**-36.8**	**-33.1**	**-33.1**	**-29.3**	**-29.3**	**-30.2**	**-30.2**	**-27.5**	**-27.5**	**-25.1**	**-25.1**	**-33.6**	**-33.6**
0.40	**-34.2**	**-34.2**	**-33.1**	**-33.1**	**-31.8**	**-31.8**	**-32.8**	**-32.8**	**-34.2**	**-34.2**	**-30.4**	**-30.4**	**-32.7**	**-32.7**	**-27.6**	**-27.6**

Table S8: Experimental Results SPA60 Z Points

P	T1	T2	T3	T4	T5	T6	T7	T8	T9	T10	T11	T12	T13	T14	T15	T16
0.10	**-0.2**	**-0.2**	**-0.3**	**-0.3**	**0.03**	**0.03**	**0.2**	**0.2**	**-0.5**	**-0.5**	**0.03**	**0.03**	**0.3**	**0.3**	**0.4**	**0.4**
0.11	**-0.1**	**-0.1**	**-0.4**	**-0.4**	**0.2**	**0.2**	**0.9**	**0.9**	**0.5**	**0.5**	**0.6**	**0.6**	**0.8**	**0.8**	**1.1**	**1.1**
0.12	**0.0**	**0.0**	**-0.5**	**-0.5**	**0.4**	**0.4**	**1.5**	**1.5**	**1.5**	**1.5**	**1.2**	**1.2**	**1.2**	**1.2**	**1.8**	**1.8**
0.13	**0.1**	**0.1**	**-0.8**	**-0.8**	**0.5**	**0.5**	**2.1**	**2.1**	**2.4**	**2.4**	**1.8**	**1.8**	**1.7**	**1.7**	**2.5**	**2.5**
0.14	**0.1**	**0.1**	**-1.0**	**-1.0**	**0.4**	**0.4**	**2.2**	**2.2**	**2.6**	**2.6**	**2.5**	**2.5**	**2.1**	**2.1**	**2.2**	**2.2**
0.15	**0.5**	**0.5**	**-1.2**	**-1.2**	**0.5**	**0.5**	**2.3**	**2.3**	**2.8**	**2.8**	**3.2**	**3.2**	**2.6**	**2.6**	**1.9**	**1.9**
0.16	**1.0**	**1.0**	**1.7**	**1.7**	**0.7**	**0.7**	**2.4**	**2.4**	**2.9**	**2.9**	**2.3**	**2.3**	**3.0**	**3.0**	**1.7**	**1.7**
0.17	**1.5**	**1.5**	**0.5**	**0.5**	**0.8**	**0.8**	**2.5**	**2.5**	**3.1**	**3.1**	**1.5**	**1.5**	**2.1**	**2.1**	**1.4**	**1.4**
0.18	**2.0**	**2.0**	**0.8**	**0.8**	**0.9**	**0.9**	**2.6**	**2.6**	**3.3**	**3.3**	**2.4**	**2.4**	**2.8**	**2.8**	**1.7**	**1.7**
0.19	**2.1**	**2.1**	**1.1**	**1.1**	**1.0**	**1.0**	**2.9**	**2.9**	**3.6**	**3.6**	**3.2**	**3.2**	**3.4**	**3.4**	**2.1**	**2.1**
0.20	**2.3**	**2.3**	**1.3**	**1.3**	**1.0**	**1.0**	**3.0**	**3.0**	**4.1**	**4.1**	**4.1**	**4.1**	**4.1**	**4.1**	**2.5**	**2.5**
0.21	**2.4**	**2.4**	**1.4**	**1.4**	**1.3**	**1.3**	**3.0**	**3.0**	**4.5**	**4.5**	**3.9**	**3.9**	**4.7**	**4.7**	**2.9**	**2.9**
0.22	**2.5**	**2.5**	**1.5**	**1.5**	**1.5**	**1.5**	**3.1**	**3.1**	**5.0**	**5.0**	**4.4**	**4.4**	**4.9**	**4.9**	**3.3**	**3.3**
0.23	**2.7**	**2.7**	**1.5**	**1.5**	**1.8**	**1.8**	**4.3**	**4.3**	**5.6**	**5.6**	**5.0**	**5.0**	**5.7**	**5.7**	**3.8**	**3.8**
0.24	**2.8**	**2.8**	**1.6**	**1.6**	**2.0**	**2.0**	**4.6**	**4.6**	**6.2**	**6.2**	**5.5**	**5.5**	**6.4**	**6.4**	**3.5**	**3.5**
0.25	**2.9**	**2.9**	**1.7**	**1.7**	**2.3**	**2.3**	**5.0**	**5.0**	**6.7**	**6.7**	**6.1**	**6.1**	**6.8**	**6.8**	**3.3**	**3.3**
0.26	**3.0**	**3.0**	**1.7**	**1.7**	**2.2**	**2.2**	**5.4**	**5.4**	**7.3**	**7.3**	**5.1**	**5.1**	**7.3**	**7.3**	**5.3**	**5.3**
0.27	**3.5**	**3.5**	**2.0**	**2.0**	**2.2**	**2.2**	**5.9**	**5.9**	**8.0**	**8.0**	**3.2**	**3.2**	**7.8**	**7.8**	**7.4**	**7.4**
0.28	**5.1**	**5.1**	**2.3**	**2.3**	**3.1**	**3.1**	**6.4**	**6.4**	**8.6**	**8.6**	**5.4**	**5.4**	**5.9**	**5.9**	**8.1**	**8.1**
0.29	**6.0**	**6.0**	**2.3**	**2.3**	**3.6**	**3.6**	**7.8**	**7.8**	**9.0**	**9.0**	**7.7**	**7.7**	**4.0**	**4.0**	**8.7**	**8.7**
0.30	**6.8**	**6.8**	**2.9**	**2.9**	**4.1**	**4.1**	**8.4**	**8.4**	**5.6**	**5.6**	**9.4**	**9.4**	**9.0**	**9.0**	**9.3**	**9.3**
0.31	**7.4**	**7.4**	**3.3**	**3.3**	**4.6**	**4.6**	**9.1**	**9.1**	**8.1**	**8.1**	**5.6**	**5.6**	**9.7**	**9.7**	**4.6**	**4.6**
0.32	**7.9**	**7.9**	**3.8**	**3.8**	**5.1**	**5.1**	**9.7**	**9.7**	**10.6**	**10.6**	**7.2**	**7.2**	**10.4**	**10.4**	**8.1**	**8.1**
0.33	**8.5**	**8.5**	**3.1**	**3.1**	**2.7**	**2.7**	**5.2**	**5.2**	**9.1**	**9.1**	**10.8**	**10.8**	**11.2**	**11.2**	**11.7**	**11.7**
0.34	**9.0**	**9.0**	**4.2**	**4.2**	**4.0**	**4.0**	**7.8**	**7.8**	**7.6**	**7.6**	**11.3**	**11.3**	**7.1**	**7.1**	**12.3**	**12.3**
0.35	**9.6**	**9.6**	**5.2**	**5.2**	**5.3**	**5.3**	**12.1**	**12.1**	**11.6**	**11.6**	**11.8**	**11.8**	**14.2**	**14.2**	**13.0**	**13.0**
0.36	**10.1**	**10.1**	**6.3**	**6.3**	**6.6**	**6.6**	**10.7**	**10.7**	**10.0**	**10.0**	**7.9**	**7.9**	**13.7**	**13.7**	**10.9**	**10.9**
0.37	**10.7**	**10.7**	**3.7**	**3.7**	**4.7**	**4.7**	**9.4**	**9.4**	**9.6**	**9.6**	**10.3**	**10.3**	**13.1**	**13.1**	**8.7**	**8.7**
0.38	**11.2**	**11.2**	**6.0**	**6.0**	**5.3**	**5.3**	**8.0**	**8.0**	**9.3**	**9.3**	**10.0**	**10.0**	**12.6**	**12.6**	**10.7**	**10.7**
0.39	**11.8**	**11.8**	**8.4**	**8.4**	**8.6**	**8.6**	**10.9**	**10.9**	**11.9**	**11.9**	**9.6**	**9.6**	**9.9**	**9.9**	**12.7**	**12.7**
0.40	**12.3**	**12.3**	**4.7**	**4.7**	**7.7**	**7.7**	**13.8**	**13.8**	**14.6**	**14.6**	**12.0**	**12.0**	**14.5**	**14.5**	**10.3**	**10.3**

Table S9: FEA Results of SPA120

P MPa	Coordinates			Calculated Angles			
	x points mm	y points mm	z points mm	y/x radian	y/x degree	z/x radian	z/x degree
**0.1**	63.211	0	0	0	0	0	0
**0.11**	63.20747981	-3.009601593	0.090667732	-0.047578709	-2.726059205	0.001434445	0.082187659
**0.12**	63.03906914	-5.800004005	0.081498064	-0.091748191	-5.25678414	0.001292818	0.074072993
**0.13**	62.73856554	-8.394256592	-0.012698289	-0.13300746	-7.620766082	-0.0002024	-0.011596669
**0.14**	62.33083478	-10.81354046	-0.179543942	-0.171776485	-9.842067593	-0.002880492	-0.165040011
**0.15**	61.8349295	-13.07546806	-0.408689618	-0.208387854	-11.93974455	-0.006609269	-0.378683205
**0.16**	61.26512126	-15.1965332	-0.69194001	-0.243138221	-13.93079392	-0.011293711	-0.647081991
**0.17**	60.63414243	-17.18563843	-1.021377921	-0.276188079	-15.8244113	-0.016843338	-0.965052158
**0.18**	59.95091561	-19.05514526	-1.391132474	-0.307747602	-17.63263875	-0.023200361	-1.329282754
**0.19**	59.22398761	-20.81214714	-1.795439363	-0.337934083	-19.36219673	-0.030306801	-1.736451804
**0.2**	58.46004823	-22.4642868	-2.229535103	-0.366870599	-21.02013693	-0.038119283	-2.184074012
**0.21**	57.66483739	-24.01811409	-2.689207792	-0.394659582	-22.61232837	-0.046601377	-2.670062198
**0.22**	56.84332851	-25.47936249	-3.170717239	-0.421388011	-24.14375456	-0.055722193	-3.192646469
**0.23**	55.99996523	-26.85298157	-3.670670986	-0.447128017	-25.61854829	-0.065454103	-3.750243839
**0.24**	55.13808588	-28.14434052	-4.186257839	-0.471959734	-27.04130088	-0.075777783	-4.341747132
**0.25**	54.26088384	-29.35770035	-4.715287209	-0.495943701	-28.41548095	-0.086682541	-4.966543749
**0.26**	53.37190679	-30.49615288	-5.254725456	-0.519116741	-29.74319833	-0.098138612	-5.62292829
**0.27**	52.47258047	-31.56473923	-5.803210258	-0.541556446	-31.02889872	-0.110147474	-6.31098536
**0.28**	51.56594137	-32.56580353	-6.358543873	-0.563286325	-32.27392907	-0.12268965	-7.029599121
**0.29**	50.65358213	-33.50293732	-6.919312477	-0.58435662	-33.48116804	-0.135760398	-7.778497827
**0.3**	49.73719076	-34.37913513	-7.483939171	-0.604806208	-34.65284317	-0.149349261	-8.557082303
**0.31**	48.81845716	-35.1969223	-8.051667213	-0.624665353	-35.79068835	-0.163459252	-9.36552529
**0.32**	47.89854578	-35.95910263	-8.620926857	-0.643971181	-36.89683079	-0.17807653	-10.20303361
**0.33**	46.97866777	-36.66804504	-9.190946579	-0.662752892	-37.97294355	-0.193200579	-11.06957778
**0.34**	46.06000093	-37.32591629	-9.760811806	-0.681035787	-39.02047629	-0.208825741	-11.96483361
**0.35**	45.14348749	-37.93484879	-10.32978725	-0.698845768	-40.04091306	-0.224948531	-12.88860146
**0.36**	44.23050645	-38.49649048	-10.89699078	-0.71619633	-41.03502701	-0.241557599	-13.84023095
**0.37**	43.32053712	-39.01353836	-11.4623642	-0.733134547	-42.00551535	-0.258666549	-14.82050153
**0.38**	42.41570428	-39.48685837	-12.02480793	-0.749653269	-42.95196844	-0.276250329	-15.82797792
**0.39**	41.51628831	-39.91835022	-12.5840044	-0.765778342	-43.87586705	-0.294307607	-16.86258378
**0.4**	40.6230388	-40.30950928	-13.13948345	-0.781524222	-44.7780395	-0.312828482	-17.92375174

Table S10: Experimental Results SPA120 X Points

P	T1	T2	T3	T4	T5	T6	T7	T8	T9	T10	T11	T12	T13	T14
0.10	**59.87**	**65.03**	**62.79**	**62.68**	**62.20**	**64.37**	**62.42**	**63.42**	**62.95**	**63.24**	**62.72**	**62.45**	**62.34**	**63.44**
0.11	**60.04**	**65.20**	**62.61**	**63.15**	**61.97**	**64.66**	**62.14**	**63.69**	**62.60**	**63.57**	**62.48**	**62.74**	**61.71**	**63.85**
0.12	**60.22**	**65.37**	**62.43**	**63.62**	**61.73**	**64.94**	**61.85**	**63.96**	**62.24**	**63.90**	**62.24**	**63.03**	**61.08**	**64.27**
0.13	**60.39**	**65.55**	**62.25**	**64.08**	**61.49**	**65.23**	**61.56**	**64.24**	**61.88**	**64.22**	**62.00**	**63.32**	**60.45**	**64.68**
0.14	**60.56**	**65.72**	**62.42**	**63.45**	**61.25**	**65.51**	**61.27**	**64.51**	**61.53**	**64.55**	**61.75**	**63.61**	**59.82**	**65.10**
0.15	**61.04**	**65.20**	**61.67**	**63.67**	**57.81**	**66.10**	**60.99**	**64.78**	**58.68**	**65.37**	**60.54**	**63.85**	**59.18**	**65.52**
0.16	**61.51**	**64.68**	**60.91**	**63.90**	**57.38**	**66.06**	**59.43**	**65.49**	**58.24**	**65.28**	**59.32**	**64.09**	**56.58**	**65.85**
0.17	**61.85**	**64.61**	**60.16**	**64.12**	**56.94**	**66.02**	**59.74**	**65.20**	**57.81**	**65.18**	**58.71**	**64.12**	**56.07**	**65.83**
0.18	**62.19**	**64.54**	**59.41**	**64.34**	**56.50**	**65.98**	**60.05**	**64.90**	**57.38**	**65.09**	**58.11**	**64.14**	**55.55**	**65.82**
0.19	**61.15**	**64.96**	**61.67**	**64.04**	**56.06**	**65.94**	**60.35**	**64.60**	**56.95**	**64.99**	**57.50**	**64.16**	**55.04**	**65.81**
0.20	**60.10**	**65.38**	**60.23**	**64.67**	**55.62**	**65.90**	**60.66**	**64.31**	**56.51**	**65.04**	**56.89**	**64.19**	**54.53**	**65.80**
0.21	**59.05**	**65.80**	**58.80**	**65.29**	**55.18**	**65.86**	**60.97**	**64.01**	**56.08**	**65.09**	**56.28**	**64.21**	**54.02**	**65.78**
0.22	**58.01**	**66.21**	**57.37**	**65.92**	**54.75**	**65.81**	**56.96**	**65.45**	**55.65**	**65.14**	**55.67**	**64.23**	**53.50**	**65.77**
0.23	**58.48**	**66.28**	**56.62**	**65.71**	**55.61**	**65.92**	**56.43**	**65.25**	**54.82**	**65.19**	**55.13**	**64.09**	**52.56**	**65.41**
0.24	**58.94**	**66.34**	**55.88**	**65.50**	**56.46**	**66.02**	**55.89**	**65.04**	**53.98**	**65.24**	**54.58**	**63.95**	**51.61**	**65.05**
0.25	**55.26**	**66.55**	**55.14**	**65.29**	**52.42**	**64.90**	**55.36**	**64.84**	**53.15**	**65.29**	**54.03**	**63.80**	**50.66**	**64.68**
0.26	**52.14**	**64.61**	**54.39**	**65.08**	**51.57**	**64.73**	**54.83**	**64.63**	**57.79**	**65.34**	**53.49**	**63.66**	**52.24**	**65.20**
0.27	**49.01**	**62.67**	**53.65**	**64.87**	**50.73**	**64.55**	**56.87**	**65.05**	**57.03**	**64.95**	**55.43**	**63.95**	**53.82**	**65.72**
0.28	**45.88**	**60.74**	**52.91**	**64.66**	**49.89**	**64.38**	**58.91**	**65.47**	**56.27**	**64.56**	**57.38**	**64.25**	**55.40**	**66.24**
0.29	**46.79**	**61.79**	**55.87**	**65.36**	**49.04**	**64.20**	**57.24**	**65.19**	**55.51**	**64.17**	**55.96**	**63.72**	**53.41**	**65.41**
0.30	**47.70**	**62.85**	**54.00**	**65.33**	**48.20**	**64.02**	**55.57**	**64.91**	**54.76**	**63.78**	**54.55**	**63.18**	**51.42**	**64.59**
0.31	**48.61**	**63.91**	**52.16**	**64.49**	**47.35**	**63.85**	**53.91**	**64.63**	**54.00**	**63.39**	**53.13**	**62.65**	**49.43**	**63.77**
0.32	**49.53**	**64.96**	**50.31**	**63.65**	**46.51**	**63.67**	**52.24**	**64.35**	**53.24**	**63.00**	**51.72**	**62.11**	**47.44**	**62.95**
0.33	**50.06**	**65.23**	**46.49**	**62.77**	**45.67**	**63.49**	**51.58**	**64.34**	**52.48**	**62.61**	**50.30**	**61.58**	**45.45**	**62.13**
0.34	**50.59**	**65.51**	**46.01**	**62.71**	**33.34**	**56.85**	**50.91**	**64.33**	**51.73**	**62.22**	**48.89**	**61.05**	**49.00**	**64.04**
0.35	**51.13**	**65.78**	**45.53**	**62.64**	**40.30**	**60.30**	**50.25**	**64.32**	**48.52**	**61.83**	**47.47**	**60.51**	**47.94**	**63.60**
0.36	**50.51**	**65.45**	**45.04**	**62.57**	**47.26**	**63.76**	**48.33**	**63.51**	**47.63**	**61.44**	**46.06**	**59.98**	**46.87**	**63.16**
0.37	**49.89**	**65.13**	**44.24**	**61.81**	**43.95**	**62.32**	**46.41**	**62.70**	**46.75**	**61.05**	**44.65**	**59.44**	**44.59**	**61.98**
0.38	**49.27**	**64.80**	**43.43**	**61.05**	**40.64**	**60.88**	**44.49**	**61.90**	**45.86**	**60.66**	**43.23**	**58.91**	**42.30**	**60.79**
0.39	**48.65**	**64.47**	**43.87**	**61.33**	**41.78**	**61.19**	**42.57**	**61.09**	**44.97**	**60.27**	**41.82**	**58.37**	**40.01**	**59.60**
0.40	**12.31**	**12.31**	**4.72**	**4.72**	**7.66**	**7.66**	**13.77**	**13.77**	**14.58**	**14.58**	**11.99**	**11.99**	**14.51**	**14.51**

Table S11: Experimental Results SPA120 Y Points

P	T1	T2	T3	T4	T5	T6	T7	T8	T9	T10	T11	T12	T13	T14
0.10	-8.87	-8.87	-1.26	-1.26	-1.24	-1.24	-1.34	-1.34	-6.86	-6.86	-1.51	-1.51	-1.89	-1.89
0.11	-8.36	-8.36	-3.05	-3.05	-3.02	-3.02	-2.81	-2.81	-7.48	-7.48	-3.50	-3.50	-3.79	-3.79
0.12	-7.85	-7.85	-4.84	-4.84	-4.80	-4.80	-4.28	-4.28	-8.11	-8.11	-5.49	-5.49	-5.68	-5.68
0.13	-7.34	-7.34	-6.63	-6.63	-6.58	-6.58	-5.76	-5.76	-8.73	-8.73	-7.49	-7.49	-7.58	-7.58
0.14	-6.83	-6.83	-4.60	-4.60	-8.36	-8.36	-7.23	-7.23	-9.35	-9.35	-9.48	-9.48	-9.47	-9.47
0.15	-4.46	-4.46	-7.46	-7.46	-16.94	-16.94	-8.70	-8.70	-15.95	-15.95	-13.37	-13.37	-11.36	-11.36
0.16	-2.08	-2.08	-10.33	-10.33	-17.73	-17.73	-15.56	-15.56	-16.74	-16.74	-17.26	-17.26	-17.05	-17.05
0.17	-1.86	-1.86	-13.20	-13.20	-18.52	-18.52	-13.79	-13.79	-17.52	-17.52	-18.07	-18.07	-17.91	-17.91
0.18	-1.64	-1.64	-16.06	-16.06	-19.31	-19.31	-12.02	-12.02	-18.31	-18.31	-18.88	-18.88	-18.78	-18.78
0.19	-4.91	-4.91	-11.01	-11.01	-20.10	-20.10	-10.26	-10.26	-19.10	-19.10	-19.69	-19.69	-19.65	-19.65
0.20	-8.19	-8.19	-13.77	-13.77	-20.89	-20.89	-8.49	-8.49	-19.89	-19.89	-20.50	-20.50	-20.52	-20.52
0.21	-11.47	-11.47	-16.54	-16.54	-21.68	-21.68	-6.72	-6.72	-20.68	-20.68	-21.31	-21.31	-21.39	-21.39
0.22	-14.74	-14.74	-19.31	-19.31	-22.47	-22.47	-21.35	-21.35	-21.47	-21.47	-22.12	-22.12	-22.25	-22.25
0.23	-12.82	-12.82	-20.72	-20.72	-20.69	-20.69	-22.07	-22.07	-22.78	-22.78	-22.79	-22.79	-23.67	-23.67
0.24	-10.90	-10.90	-22.14	-22.14	-18.91	-18.91	-22.80	-22.80	-24.08	-24.08	-23.46	-23.46	-25.09	-25.09
0.25	-17.44	-17.44	-23.55	-23.55	-25.04	-25.04	-23.52	-23.52	-25.39	-25.39	-24.13	-24.13	-26.51	-26.51
0.26	-21.26	-21.26	-24.97	-24.97	-25.90	-25.90	-24.25	-24.25	-19.15	-19.15	-24.80	-24.80	-24.31	-24.31
0.27	-25.09	-25.09	-26.38	-26.38	-26.76	-26.76	-20.99	-20.99	-20.18	-20.18	-21.76	-21.76	-22.10	-22.10
0.28	-28.91	-28.91	-27.80	-27.80	-27.62	-27.62	-17.73	-17.73	-21.20	-21.20	-18.72	-18.72	-19.89	-19.89
0.29	-28.30	-28.30	-22.38	-22.38	-28.48	-28.48	-20.07	-20.07	-22.23	-22.23	-20.18	-20.18	-22.54	-22.54
0.30	-27.68	-27.68	-25.62	-25.62	-29.34	-29.34	-22.41	-22.41	-23.25	-23.25	-21.64	-21.64	-25.19	-25.19
0.31	-27.07	-27.07	-27.69	-27.69	-30.19	-30.19	-24.75	-24.75	-24.28	-24.28	-23.10	-23.10	-27.84	-27.84
0.32	-26.45	-26.45	-29.75	-29.75	-31.05	-31.05	-27.09	-27.09	-25.30	-25.30	-24.56	-24.56	-30.49	-30.49
0.33	-25.77	-25.77	-33.50	-33.50	-31.91	-31.91	-27.72	-27.72	-26.33	-26.33	-26.01	-26.01	-33.14	-33.14
0.34	-25.10	-25.10	-33.68	-33.68	-38.86	-38.86	-28.35	-28.35	-27.35	-27.35	-27.47	-27.47	-28.88	-28.88
0.35	-24.42	-24.42	-33.87	-33.87	-34.53	-34.53	-28.98	-28.98	-29.67	-29.67	-28.93	-28.93	-30.07	-30.07
0.36	-24.97	-24.97	-34.05	-34.05	-30.19	-30.19	-30.53	-30.53	-29.98	-29.98	-30.39	-30.39	-31.25	-31.25
0.37	-25.51	-25.51	-34.84	-34.84	-33.02	-33.02	-32.08	-32.08	-30.29	-30.29	-31.85	-31.85	-32.99	-32.99
0.38	-26.06	-26.06	-35.62	-35.62	-35.84	-35.84	-33.63	-33.63	-30.60	-30.60	-33.31	-33.31	-34.72	-34.72
0.39	-26.60	-26.60	-35.53	-35.53	-34.98	-34.98	-35.18	-35.18	-30.92	-30.92	-34.77	-34.77	-36.46	-36.46
0.40	12.31	12.31	4.72	4.72	7.66	7.66	13.77	13.77	14.58	14.58	11.99	11.99	14.51	14.51

Table S12: Experimental Results SPA120 Z Points

P	T1	T2	T3	T4	T5	T6	T7	T8	T9	T10	T11	T12	T13	T14
0.10	**-4.86**	**-4.86**	**-0.28**	**-0.28**	**-0.91**	**-0.91**	**-0.44**	**-0.44**	**-0.58**	**-0.58**	**-0.45**	**-0.45**	**-0.45**	**-0.45**
0.11	**-4.26**	**-4.26**	**-0.70**	**-0.70**	**-1.61**	**-1.61**	**-0.95**	**-0.95**	**-1.20**	**-1.20**	**-1.04**	**-1.04**	**-0.83**	**-0.83**
0.12	**-3.65**	**-3.65**	**-1.12**	**-1.12**	**-2.31**	**-2.31**	**-1.46**	**-1.46**	**-1.83**	**-1.83**	**-1.62**	**-1.62**	**-1.22**	**-1.22**
0.13	**-3.05**	**-3.05**	**-1.54**	**-1.54**	**-3.01**	**-3.01**	**-1.97**	**-1.97**	**-2.46**	**-2.46**	**-2.21**	**-2.21**	**-1.60**	**-1.60**
0.14	**-2.44**	**-2.44**	**-1.20**	**-1.20**	**-3.71**	**-3.71**	**-2.48**	**-2.48**	**-3.09**	**-3.09**	**-2.80**	**-2.80**	**-1.98**	**-1.98**
0.15	**-1.56**	**-1.56**	**-1.65**	**-1.65**	**-3.94**	**-3.94**	**-2.99**	**-2.99**	**-4.41**	**-4.41**	**-3.31**	**-3.31**	**-2.37**	**-2.37**
0.16	**-0.68**	**-0.68**	**-2.10**	**-2.10**	**-4.17**	**-4.17**	**-4.01**	**-4.01**	**-4.03**	**-4.03**	**-3.83**	**-3.83**	**-3.98**	**-3.98**
0.17	**-0.06**	**-0.06**	**-2.55**	**-2.55**	**-4.40**	**-4.40**	**-3.90**	**-3.90**	**-3.65**	**-3.65**	**-4.00**	**-4.00**	**-4.27**	**-4.27**
0.18	**0.56**	**0.56**	**-3.00**	**-3.00**	**-4.62**	**-4.62**	**-3.79**	**-3.79**	**-3.27**	**-3.27**	**-4.17**	**-4.17**	**-4.57**	**-4.57**
0.19	**-0.50**	**-0.50**	**-2.51**	**-2.51**	**-4.85**	**-4.85**	**-3.68**	**-3.68**	**-2.89**	**-2.89**	**-4.34**	**-4.34**	**-4.87**	**-4.87**
0.20	**-1.57**	**-1.57**	**-2.96**	**-2.96**	**-5.08**	**-5.08**	**-3.57**	**-3.57**	**-3.23**	**-3.23**	**-4.51**	**-4.51**	**-5.17**	**-5.17**
0.21	**-2.63**	**-2.63**	**-3.42**	**-3.42**	**-5.31**	**-5.31**	**-3.46**	**-3.46**	**-3.57**	**-3.57**	**-4.68**	**-4.68**	**-5.47**	**-5.47**
0.22	**-3.70**	**-3.70**	**-3.87**	**-3.87**	**-5.54**	**-5.54**	**-5.01**	**-5.01**	**-3.91**	**-3.91**	**-4.85**	**-4.85**	**-5.77**	**-5.77**
0.23	**-3.39**	**-3.39**	**-4.40**	**-4.40**	**-5.54**	**-5.54**	**-5.50**	**-5.50**	**-4.25**	**-4.25**	**-5.44**	**-5.44**	**-6.48**	**-6.48**
0.24	**-3.07**	**-3.07**	**-4.93**	**-4.93**	**-5.55**	**-5.55**	**-6.00**	**-6.00**	**-4.59**	**-4.59**	**-6.03**	**-6.03**	**-7.20**	**-7.20**
0.25	**-4.61**	**-4.61**	**-5.45**	**-5.45**	**-7.50**	**-7.50**	**-6.49**	**-6.49**	**-4.93**	**-4.93**	**-6.63**	**-6.63**	**-7.92**	**-7.92**
0.26	**-7.29**	**-7.29**	**-5.98**	**-5.98**	**-7.97**	**-7.97**	**-6.98**	**-6.98**	**-5.27**	**-5.27**	**-7.22**	**-7.22**	**-6.67**	**-6.67**
0.27	**-9.98**	**-9.98**	**-6.51**	**-6.51**	**-8.44**	**-8.44**	**-5.75**	**-5.75**	**-5.81**	**-5.81**	**-5.70**	**-5.70**	**-5.43**	**-5.43**
0.28	**-12.66**	**-12.66**	**-7.04**	**-7.04**	**-8.91**	**-8.91**	**-4.51**	**-4.51**	**-6.35**	**-6.35**	**-4.17**	**-4.17**	**-4.19**	**-4.19**
0.29	**-11.32**	**-11.32**	**-5.62**	**-5.62**	**-9.38**	**-9.38**	**-5.38**	**-5.38**	**-6.89**	**-6.89**	**-5.06**	**-5.06**	**-5.71**	**-5.71**
0.30	**-9.98**	**-9.98**	**-6.42**	**-6.42**	**-9.84**	**-9.84**	**-6.24**	**-6.24**	**-7.42**	**-7.42**	**-5.94**	**-5.94**	**-7.23**	**-7.23**
0.31	**-8.64**	**-8.64**	**-7.27**	**-7.27**	**-10.31**	**-10.31**	**-7.10**	**-7.10**	**-7.96**	**-7.96**	**-6.82**	**-6.82**	**-8.75**	**-8.75**
0.32	**-7.30**	**-7.30**	**-8.12**	**-8.12**	**-10.78**	**-10.78**	**-7.97**	**-7.97**	**-8.50**	**-8.50**	**-7.70**	**-7.70**	**-10.28**	**-10.28**
0.33	**-7.24**	**-7.24**	**-10.02**	**-10.02**	**-11.25**	**-11.25**	**-8.22**	**-8.22**	**-9.04**	**-9.04**	**-8.58**	**-8.58**	**-11.80**	**-11.80**
0.34	**-7.18**	**-7.18**	**-10.34**	**-10.34**	**-18.75**	**-18.75**	**-8.47**	**-8.47**	**-9.57**	**-9.57**	**-9.46**	**-9.46**	**-9.08**	**-9.08**
0.35	**-7.12**	**-7.12**	**-10.66**	**-10.66**	**-14.40**	**-14.40**	**-8.72**	**-8.72**	**-10.11**	**-10.11**	**-10.34**	**-10.34**	**-9.96**	**-9.96**
0.36	**-7.37**	**-7.37**	**-10.97**	**-10.97**	**-10.06**	**-10.06**	**-10.25**	**-10.25**	**-10.65**	**-10.65**	**-11.22**	**-11.22**	**-10.84**	**-10.84**
0.37	**-7.62**	**-7.62**	**-11.91**	**-11.91**	**-12.50**	**-12.50**	**-11.78**	**-11.78**	**-11.19**	**-11.19**	**-12.10**	**-12.10**	**-12.19**	**-12.19**
0.38	**-7.87**	**-7.87**	**-12.84**	**-12.84**	**-14.94**	**-14.94**	**-13.31**	**-13.31**	**-11.72**	**-11.72**	**-12.99**	**-12.99**	**-13.53**	**-13.53**
0.39	**-8.13**	**-8.13**	**-12.43**	**-12.43**	**-14.08**	**-14.08**	**-14.84**	**-14.84**	**-12.26**	**-12.26**	**-13.87**	**-13.87**	**-14.88**	**-14.88**
0.40	**12.31**	**12.31**	**4.72**	**4.72**	**7.66**	**7.66**	**13.77**	**13.77**	**14.58**	**14.58**	**11.99**	**11.99**	**14.51**	**14.51**

Table S13: FEA Results of SPA145

P MPa	Coordinates			Calculated Angles			
	x points mm	y points mm	z points mm	y/x radian	y/x degree	z/x radian	z/x degree
**0.1**	65.611	0	0	0	0	0	0
**0.11**	65.6226505	-4.303837299	0.232534453	-0.065490839	-3.752348651	0.003543494	0.203027261
**0.12**	65.28612503	-8.400259972	-0.003484825	-0.127965293	-7.331871192	-5.33777E-05	-0.003058319
**0.13**	64.65639108	-12.19015312	-0.649886072	-0.186350007	-10.67706894	-0.010051043	-0.575882361
**0.14**	63.7930544	-15.61979008	-1.635281086	-0.24012666	-13.75824414	-0.025628543	-1.468407352
**0.15**	62.75250071	-18.66449738	-2.887821913	-0.28909764	-16.56407467	-0.045986788	-2.63484884
**0.16**	61.58380312	-21.32180595	-4.342394829	-0.333307169	-19.09709406	-0.07039545	-4.033362167
**0.17**	60.32991212	-23.59958839	-5.940520763	-0.372876048	-21.36422384	-0.098150853	-5.623629617
**0.18**	59.02569145	-25.51658058	-7.63363266	-0.408034306	-23.37864361	-0.128613416	-7.369005936
**0.19**	57.7002024	-27.09558868	-9.380672455	-0.439027155	-25.1544031	-0.161166022	-9.234132852
**0.2**	56.37642854	-28.3626442	-11.14880085	-0.466119762	-26.70669512	-0.195237346	-11.18627595
**0.21**	55.07254976	-29.34464836	-12.91165543	-0.489570208	-28.05030669	-0.230288893	-13.19458164
**0.22**	53.80279058	-30.06808662	-14.64848614	-0.50961806	-29.19896401	-0.265819469	-15.23033368
**0.23**	52.57822221	-30.55834389	-16.34296227	-0.526479571	-30.16505741	-0.301363996	-17.26688509
**0.24**	51.40691179	-30.83961678	-17.98291016	-0.540354699	-30.9600437	-0.336510022	-19.28060402
**0.25**	50.29764742	-30.93367958	-19.55549622	-0.551385001	-31.59203345	-0.370810118	-21.24585476
**0.26**	49.25259012	-30.86277962	-21.0564518	-0.559765184	-32.07218255	-0.404002906	-23.14766141
**0.27**	48.27643198	-30.64663696	-22.4783783	-0.565626679	-32.40802146	-0.435765727	-24.96753699
**0.28**	47.37346262	-30.30448914	-23.81434059	-0.569095619	-32.6067771	-0.465800236	-26.68838761
**0.29**	46.54574388	-29.85548782	-25.06015587	-0.570321697	-32.67702622	-0.493892451	-28.297953
**0.3**	45.79597086	-29.31889915	-26.21074677	-0.569459997	-32.62765444	-0.519830961	-29.78412012
**0.31**	45.12223619	-28.71279526	-27.26680756	-0.566707843	-32.46996759	-0.543566181	-31.14404803
**0.32**	44.52832979	-28.05790901	-28.22196579	-0.562268108	-32.21558955	-0.564900956	-32.36644064
**0.33**	44.01378625	-27.3744812	-29.07582855	-0.5564048	-31.87964675	-0.583795918	-33.44904222
**0.34**	43.57795709	-26.6830883	-29.82909775	-0.549419656	-31.47942747	-0.60024714	-34.39162777
**0.35**	43.21876329	-26.00317574	-30.48515892	-0.541642161	-31.03380984	-0.614319977	-35.19794196
**0.36**	42.93222803	-25.3513546	-31.05032539	-0.533402744	-30.56172604	-0.62615392	-35.87597692
**0.37**	42.71262735	-24.74020386	-31.53312302	-0.525003372	-30.08047743	-0.635946568	-36.43705436
**0.38**	42.55257982	-24.17748642	-31.94368935	-0.516693082	-29.60433293	-0.643940957	-36.89509911
**0.39**	42.44408029	-23.66671753	-32.29224014	-0.508657586	-29.14393288	-0.650389051	-37.26454764
**0.4**	42.37867731	-23.20759201	-32.5888176	-0.5010174	-28.70618251	-0.655544916	-37.55995697

Table S14: Experimental Results SPA145 X Points

P	T1	T2	T3	T4	T5	T6	T7	T8	T9	T10	T11	T12
0.10	**64.86**	**65.49**	**63.33**	**65.70**	**64.71**	**64.92**	**64.44**	**63.58**	**64.27**	**64.69**	**63.17**	**64.18**
0.11	**64.10**	**65.57**	**62.17**	**66.09**	**63.82**	**64.54**	**63.48**	**63.07**	**62.91**	**63.82**	**61.20**	**63.00**
0.12	**63.34**	**65.66**	**61.02**	**66.48**	**62.94**	**64.15**	**62.52**	**62.57**	**61.55**	**62.95**	**59.23**	**61.82**
0.13	**62.59**	**65.75**	**59.86**	**66.87**	**62.05**	**63.76**	**61.56**	**62.06**	**60.19**	**62.08**	**57.26**	**60.64**
0.14	**61.83**	**65.83**	**57.81**	**66.12**	**61.16**	**63.38**	**63.22**	**61.49**	**63.61**	**63.61**	**59.17**	**61.77**
0.15	**61.07**	**65.92**	**55.76**	**65.37**	**59.41**	**63.05**	**63.92**	**62.80**	**62.83**	**62.90**	**61.08**	**62.90**
0.16	**58.48**	**66.35**	**56.98**	**65.44**	**57.65**	**62.73**	**64.61**	**64.11**	**62.05**	**62.19**	**61.08**	**63.31**
0.17	**55.88**	**66.79**	**58.21**	**65.51**	**55.90**	**62.41**	**62.86**	**62.22**	**61.27**	**61.48**	**61.08**	**63.72**
0.18	**58.25**	**66.41**	**59.43**	**65.58**	**55.19**	**61.85**	**61.11**	**60.33**	**60.49**	**60.76**	**58.46**	**62.14**
0.19	**60.62**	**66.02**	**60.65**	**65.65**	**54.47**	**61.30**	**59.35**	**58.43**	**59.71**	**60.05**	**55.83**	**60.57**
0.20	**62.98**	**65.64**	**58.47**	**65.24**	**53.76**	**60.74**	**58.67**	**58.13**	**58.93**	**59.34**	**53.21**	**59.00**
0.21	**61.93**	**65.45**	**56.29**	**64.83**	**53.04**	**60.19**	**57.99**	**57.83**	**58.87**	**58.56**	**54.28**	**57.87**
0.22	**60.88**	**65.26**	**54.10**	**64.42**	**52.33**	**59.63**	**57.30**	**57.53**	**58.80**	**57.77**	**55.35**	**56.75**
0.23	**59.83**	**65.07**	**53.75**	**64.21**	**51.57**	**59.28**	**56.62**	**57.23**	**58.74**	**56.99**	**55.95**	**58.08**
0.24	**58.77**	**64.88**	**53.40**	**64.00**	**50.81**	**58.92**	**58.21**	**55.99**	**59.78**	**61.52**	**56.55**	**59.41**
0.25	**57.72**	**64.69**	**53.05**	**63.79**	**50.06**	**58.56**	**59.79**	**54.75**	**59.50**	**60.15**	**54.45**	**58.06**
0.26	**56.67**	**64.50**	**54.25**	**64.69**	**51.56**	**59.15**	**59.28**	**54.26**	**59.21**	**58.77**	**52.35**	**56.71**
0.27	**55.62**	**64.31**	**52.87**	**63.74**	**53.06**	**59.73**	**58.76**	**53.77**	**58.92**	**57.40**	**50.25**	**55.35**
0.28	**54.58**	**63.60**	**51.50**	**62.79**	**54.56**	**60.32**	**58.24**	**53.27**	**58.63**	**56.02**	**48.15**	**54.00**
0.29	**53.55**	**62.89**	**50.13**	**61.84**	**52.31**	**59.22**	**57.72**	**52.78**	**58.34**	**54.65**	**46.05**	**52.65**
0.30	**52.51**	**62.17**	**48.76**	**60.90**	**50.05**	**58.11**	**56.42**	**52.21**	**55.86**	**53.66**	**54.88**	**55.11**
0.31	**51.48**	**61.46**	**47.39**	**59.95**	**47.80**	**57.01**	**55.12**	**51.63**	**51.53**	**50.92**	**52.49**	**54.22**
0.32	**50.44**	**60.74**	**46.02**	**59.00**	**47.14**	**56.20**	**53.81**	**51.06**	**50.93**	**50.86**	**50.11**	**53.33**
0.33	**49.41**	**60.03**	**44.65**	**58.05**	**46.48**	**55.38**	**52.51**	**50.49**	**50.32**	**50.80**	**47.72**	**52.44**
0.34	**48.37**	**59.31**	**47.30**	**58.75**	**45.67**	**54.68**	**51.20**	**49.92**	**50.35**	**49.99**	**44.50**	**51.11**
0.35	**49.62**	**62.66**	**49.95**	**59.44**	**44.85**	**53.98**	**49.90**	**49.34**	**50.38**	**49.18**	**41.28**	**49.78**
0.36	**49.79**	**61.61**	**48.74**	**59.18**	**44.04**	**53.28**	**48.59**	**48.77**	**49.88**	**50.20**	**40.32**	**49.47**
0.37	**49.96**	**60.57**	**47.54**	**58.91**	**43.22**	**52.58**	**46.34**	**46.84**	**49.38**	**51.22**	**39.37**	**49.16**
0.38	**49.31**	**60.04**	**46.33**	**58.65**	**42.30**	**52.48**	**44.08**	**44.92**	**48.88**	**52.24**	**38.41**	**48.65**
0.39	**48.66**	**59.51**	**45.12**	**58.39**	**41.37**	**52.38**	**41.83**	**42.99**	**45.79**	**49.28**	**37.46**	**48.14**
0.40	**12.31**	**12.31**	**4.72**	**4.72**	**7.66**	**7.66**	**13.77**	**13.77**	**14.58**	**14.58**	**11.99**	**11.99**

Table S15: Experimental Results SPA145 Y Points

P	T1	T2	T3	T4	T5	T6	T7	T8	T9	T10	T11	T12
0.10	**-2.05**	**-2.05**	**-2.38**	**-2.38**	**-2.65**	**-2.65**	**-3.80**	**-3.80**	**-4.37**	**-4.37**	**-11.51**	**-11.51**
0.11	**-4.11**	**-4.11**	**-5.51**	**-5.51**	**-5.41**	**-5.41**	**-7.01**	**-7.01**	**-8.28**	**-8.28**	**-15.15**	**-15.15**
0.12	**-6.17**	**-6.17**	**-8.65**	**-8.65**	**-8.18**	**-8.18**	**-10.22**	**-10.22**	**-12.20**	**-12.20**	**-18.79**	**-18.79**
0.13	**-8.23**	**-8.23**	**-11.78**	**-11.78**	**-10.95**	**-10.95**	**-13.43**	**-13.43**	**-16.12**	**-16.12**	**-22.43**	**-22.43**
0.14	**-10.30**	**-10.30**	**-16.27**	**-16.27**	**-13.71**	**-13.71**	**-6.79**	**-6.79**	**-9.83**	**-9.83**	**-19.80**	**-19.80**
0.15	**-12.36**	**-12.36**	**-20.77**	**-20.77**	**-16.58**	**-16.58**	**-6.37**	**-6.37**	**-11.49**	**-11.49**	**-17.18**	**-17.18**
0.16	**-16.70**	**-16.70**	**-17.91**	**-17.91**	**-19.44**	**-19.44**	**-5.96**	**-5.96**	**-13.14**	**-13.14**	**-16.34**	**-16.34**
0.17	**-21.03**	**-21.03**	**-15.04**	**-15.04**	**-22.31**	**-22.31**	**-10.43**	**-10.43**	**-14.79**	**-14.79**	**-15.51**	**-15.51**
0.18	**-16.46**	**-16.46**	**-12.18**	**-12.18**	**-22.97**	**-22.97**	**-14.91**	**-14.91**	**-16.44**	**-16.44**	**-19.01**	**-19.01**
0.19	**-11.89**	**-11.89**	**-9.32**	**-9.32**	**-23.63**	**-23.63**	**-19.38**	**-19.38**	**-18.10**	**-18.10**	**-22.51**	**-22.51**
0.20	**-7.32**	**-7.32**	**-14.19**	**-14.19**	**-24.28**	**-24.28**	**-19.92**	**-19.92**	**-19.75**	**-19.75**	**-26.01**	**-26.01**
0.21	**-9.42**	**-9.42**	**-19.05**	**-19.05**	**-24.94**	**-24.94**	**-20.45**	**-20.45**	**-21.83**	**-21.83**	**-28.87**	**-28.87**
0.22	**-11.52**	**-11.52**	**-23.92**	**-23.92**	**-25.60**	**-25.60**	**-20.98**	**-20.98**	**-23.90**	**-23.90**	**-31.73**	**-31.73**
0.23	**-13.62**	**-13.62**	**-24.37**	**-24.37**	**-25.77**	**-25.77**	**-21.52**	**-21.52**	**-25.98**	**-25.98**	**-27.44**	**-27.44**
0.24	**-15.71**	**-15.71**	**-24.83**	**-24.83**	**-25.95**	**-25.95**	**-23.69**	**-23.69**	**-17.51**	**-17.51**	**-23.14**	**-23.14**
0.25	**-17.81**	**-17.81**	**-25.28**	**-25.28**	**-26.12**	**-26.12**	**-25.86**	**-25.86**	**-20.56**	**-20.56**	**-26.58**	**-26.58**
0.26	**-19.91**	**-19.91**	**-21.70**	**-21.70**	**-25.24**	**-25.24**	**-26.89**	**-26.89**	**-23.61**	**-23.61**	**-30.02**	**-30.02**
0.27	**-22.01**	**-22.01**	**-24.11**	**-24.11**	**-24.36**	**-24.36**	**-27.92**	**-27.92**	**-26.66**	**-26.66**	**-33.45**	**-33.45**
0.28	**-24.17**	**-24.17**	**-26.52**	**-26.52**	**-23.49**	**-23.49**	**-28.96**	**-28.96**	**-29.71**	**-29.71**	**-36.89**	**-36.89**
0.29	**-26.32**	**-26.32**	**-28.92**	**-28.92**	**-24.76**	**-24.76**	**-29.99**	**-29.99**	**-32.77**	**-32.77**	**-40.32**	**-40.32**
0.30	**-28.48**	**-28.48**	**-31.33**	**-31.33**	**-26.04**	**-26.04**	**-30.84**	**-30.84**	**-34.90**	**-34.90**	**-39.37**	**-39.37**
0.31	**-30.64**	**-30.64**	**-33.73**	**-33.73**	**-27.32**	**-27.32**	**-31.70**	**-31.70**	**-34.80**	**-34.80**	**-38.73**	**-38.73**
0.32	**-32.80**	**-32.80**	**-36.14**	**-36.14**	**-28.25**	**-28.25**	**-32.56**	**-32.56**	**-35.53**	**-35.53**	**-38.10**	**-38.10**
0.33	**-34.95**	**-34.95**	**-38.54**	**-38.54**	**-29.18**	**-29.18**	**-33.41**	**-33.41**	**-36.27**	**-36.27**	**-37.46**	**-37.46**
0.34	**-37.11**	**-37.11**	**-36.61**	**-36.61**	**-28.63**	**-28.63**	**-34.27**	**-34.27**	**-35.18**	**-35.18**	**-37.94**	**-37.94**
0.35	**-27.84**	**-27.84**	**-34.67**	**-34.67**	**-28.08**	**-28.08**	**-35.12**	**-35.12**	**-34.10**	**-34.10**	**-38.41**	**-38.41**
0.36	**-31.12**	**-31.12**	**-35.21**	**-35.21**	**-27.53**	**-27.53**	**-35.98**	**-35.98**	**-34.51**	**-34.51**	**-36.98**	**-36.98**
0.37	**-34.40**	**-34.40**	**-35.76**	**-35.76**	**-26.98**	**-26.98**	**-35.03**	**-35.03**	**-34.92**	**-34.92**	**-35.55**	**-35.55**
0.38	**-35.12**	**-35.12**	**-36.30**	**-36.30**	**-26.55**	**-26.55**	**-34.08**	**-34.08**	**-35.33**	**-35.33**	**-36.98**	**-36.98**
0.39	**-35.85**	**-35.85**	**-36.85**	**-36.85**	**-26.12**	**-26.12**	**-33.13**	**-33.13**	**-34.15**	**-34.15**	**-38.41**	**-38.41**
0.40	**12.31**	**12.31**	**4.72**	**4.72**	**7.66**	**7.66**	**13.77**	**13.77**	**14.58**	**14.58**	**11.99**	**11.99**

Table S16: Experimental Results SPA145 Z Points

P	T1	T2	T3	T4	T5	T6	T7	T8	T9	T10	T11	T12
0.10	**-0.40**	**-0.40**	**-0.95**	**-0.95**	**-1.88**	**-1.88**	**-1.19**	**-1.19**	**-1.63**	**-1.63**	**0.66**	**0.66**
0.11	**-0.77**	**-0.77**	**-1.74**	**-1.74**	**-2.66**	**-2.66**	**-2.31**	**-2.31**	**-3.15**	**-3.15**	**-0.57**	**-0.57**
0.12	**-1.15**	**-1.15**	**-2.52**	**-2.52**	**-3.44**	**-3.44**	**-3.44**	**-3.44**	**-4.67**	**-4.67**	**-1.80**	**-1.80**
0.13	**-1.52**	**-1.52**	**-3.31**	**-3.31**	**-4.22**	**-4.22**	**-4.56**	**-4.56**	**-6.20**	**-6.20**	**-3.03**	**-3.03**
0.14	**-1.90**	**-1.90**	**-6.23**	**-6.23**	**-5.00**	**-5.00**	**0.81**	**0.81**	**-3.03**	**-3.03**	**-1.19**	**-1.19**
0.15	**-2.27**	**-2.27**	**-9.15**	**-9.15**	**-7.03**	**-7.03**	**0.54**	**0.54**	**-4.36**	**-4.36**	**0.66**	**0.66**
0.16	**-4.44**	**-4.44**	**-7.48**	**-7.48**	**-9.05**	**-9.05**	**0.27**	**0.27**	**-5.69**	**-5.69**	**0.76**	**0.76**
0.17	**-6.60**	**-6.60**	**-5.80**	**-5.80**	**-11.08**	**-11.08**	**-2.58**	**-2.58**	**-7.03**	**-7.03**	**0.86**	**0.86**
0.18	**-4.86**	**-4.86**	**-4.12**	**-4.12**	**-12.21**	**-12.21**	**-5.42**	**-5.42**	**-8.36**	**-8.36**	**-2.08**	**-2.08**
0.19	**-3.13**	**-3.13**	**-2.44**	**-2.44**	**-13.34**	**-13.34**	**-8.26**	**-8.26**	**-9.69**	**-9.69**	**-5.01**	**-5.01**
0.20	**-1.40**	**-1.40**	**-6.02**	**-6.02**	**-14.47**	**-14.47**	**-8.90**	**-8.90**	**-11.03**	**-11.03**	**-7.95**	**-7.95**
0.21	**-2.97**	**-2.97**	**-9.60**	**-9.60**	**-15.60**	**-15.60**	**-9.55**	**-9.55**	**-11.85**	**-11.85**	**-8.87**	**-8.87**
0.22	**-4.54**	**-4.54**	**-13.18**	**-13.18**	**-16.73**	**-16.73**	**-10.19**	**-10.19**	**-12.66**	**-12.66**	**-9.80**	**-9.80**
0.23	**-6.10**	**-6.10**	**-14.03**	**-14.03**	**-17.93**	**-17.93**	**-10.83**	**-10.83**	**-13.48**	**-13.48**	**-7.34**	**-7.34**
0.24	**-7.67**	**-7.67**	**-14.89**	**-14.89**	**-19.13**	**-19.13**	**-11.73**	**-11.73**	**-7.90**	**-7.90**	**-4.88**	**-4.88**
0.25	**-9.24**	**-9.24**	**-15.74**	**-15.74**	**-20.32**	**-20.32**	**-12.63**	**-12.63**	**-9.47**	**-9.47**	**-7.75**	**-7.75**
0.26	**-10.81**	**-10.81**	**-10.40**	**-10.40**	**-18.40**	**-18.40**	**-13.59**	**-13.59**	**-11.03**	**-11.03**	**-10.62**	**-10.62**
0.27	**-12.37**	**-12.37**	**-12.44**	**-12.44**	**-16.47**	**-16.47**	**-14.54**	**-14.54**	**-12.59**	**-12.59**	**-13.49**	**-13.49**
0.28	**-13.80**	**-13.80**	**-14.49**	**-14.49**	**-14.54**	**-14.54**	**-15.50**	**-15.50**	**-14.16**	**-14.16**	**-16.36**	**-16.36**
0.29	**-15.24**	**-15.24**	**-16.54**	**-16.54**	**-17.85**	**-17.85**	**-16.46**	**-16.46**	**-15.72**	**-15.72**	**-19.22**	**-19.22**
0.30	**-16.67**	**-16.67**	**-18.59**	**-18.59**	**-21.16**	**-21.16**	**-17.08**	**-17.08**	**-20.46**	**-20.46**	**-14.31**	**-14.31**
0.31	**-18.10**	**-18.10**	**-20.63**	**-20.63**	**-24.47**	**-24.47**	**-17.70**	**-17.70**	**-22.23**	**-22.23**	**-16.70**	**-16.70**
0.32	**-19.53**	**-19.53**	**-22.68**	**-22.68**	**-26.03**	**-26.03**	**-18.32**	**-18.32**	**-23.10**	**-23.10**	**-19.09**	**-19.09**
0.33	**-20.96**	**-20.96**	**-24.73**	**-24.73**	**-27.58**	**-27.58**	**-18.94**	**-18.94**	**-23.97**	**-23.97**	**-21.48**	**-21.48**
0.34	**-22.39**	**-22.39**	**-23.39**	**-23.39**	**-28.22**	**-28.22**	**-19.56**	**-19.56**	**-27.73**	**-27.73**	**-25.17**	**-25.17**
0.35	**-16.34**	**-16.34**	**-22.05**	**-22.05**	**-28.85**	**-28.85**	**-20.18**	**-20.18**	**-31.50**	**-31.50**	**-28.86**	**-28.86**
0.36	**-17.79**	**-17.79**	**-23.10**	**-23.10**	**-29.49**	**-29.49**	**-20.80**	**-20.80**	**-30.48**	**-30.48**	**-29.98**	**-29.98**
0.37	**-19.25**	**-19.25**	**-24.14**	**-24.14**	**-30.13**	**-30.13**	**-25.42**	**-25.42**	**-29.46**	**-29.46**	**-31.11**	**-31.11**
0.38	**-20.59**	**-20.59**	**-25.19**	**-25.19**	**-35.91**	**-35.91**	**-30.03**	**-30.03**	**-28.44**	**-28.44**	**-33.98**	**-33.98**
0.39	**-21.93**	**-21.93**	**-26.23**	**-26.23**	**-41.69**	**-41.69**	**-34.65**	**-34.65**	**-33.25**	**-33.25**	**-36.85**	**-36.85**
0.40	**12.31**	**12.31**	**4.72**	**4.72**	**7.66**	**7.66**	**13.77**	**13.77**	**14.58**	**14.58**	**11.99**	**11.99**

Table S17: Turtle movement actuated by front drive SPA60 and SPA120.

Turtle		SPA120				SPA60			
**Y**	**X**	**Rot ang**	**Z**	**Y**	**X**	**Rot ang**	**Z**	**Y**	**X**
-178.45	24.19	0.39	-16.38	-39.56	37.26	-0.44	17.13	-36.12	41.61
-178.46	24.18	0.39	-16.38	-39.56	57.04	-0.44	17.13	-36.12	52.86
-178.46	24.18	0.47	-19.71	-39.03	34.26	-0.42	16.7	-37.12	43.93
-178.48	24.19	0.46	-18.76	-38.17	57.05	-0.42	16.7	-37.12	50.11
-178.47	24.2	0.39	-16.38	-39.56	37.26	-0.44	17.13	-36.12	41.61
-178.42	24.22	0.39	-16.38	-39.56	57.04	-0.44	17.13	-36.12	52.86
-178.43	24.21	0.47	-19.71	-39.03	34.26	-0.42	16.7	-37.12	43.93
-178.37	24.18	0.46	-18.76	-38.17	57.05	-0.42	16.7	-37.12	50.11
-178.4	24.18	0.39	-16.38	-39.56	37.26	-0.44	17.13	-36.12	41.61
-178.39	24.17	0.39	-16.38	-39.56	57.04	-0.44	17.13	-36.12	52.86
-178.41	24.18	0.47	-19.71	-39.03	34.26	-0.42	16.7	-37.12	43.93
-178.47	24.17	0.46	-18.76	-38.17	57.05	-0.42	16.7	-37.12	50.11
-178.5	24.18	0.39	-16.38	-39.56	37.26	-0.44	17.13	-36.12	41.61
-178.53	24.21	0.39	-16.38	-39.56	57.04	-0.44	17.13	-36.12	52.86
-178.47	24.21	0.47	-19.71	-39.03	34.26	-0.42	16.7	-37.12	43.93
-178.47	24.21	0.46	-18.76	-38.17	57.05	-0.42	16.7	-37.12	50.11
-178.49	24.22	0.39	-16.38	-39.56	37.26	-0.44	17.13	-36.12	41.61
-178.27	24.19	0.39	-16.38	-39.56	57.04	-0.44	17.13	-36.12	52.86
-178.34	24.18	0.47	-19.71	-39.03	34.26	-0.42	16.7	-37.12	43.93
-178.38	24.17	0.46	-18.76	-38.17	57.05	-0.42	16.7	-37.12	50.11
-178.4	24.18	0.39	-16.38	-39.56	37.26	-0.44	17.13	-36.12	41.61
-178.61	24.21	0.39	-16.38	-39.56	57.04	-0.44	17.13	-36.12	52.86
-179.13	24.09	0.47	-19.71	-39.03	34.26	-0.42	16.7	-37.12	43.93
-179	23.94	0.46	-18.76	-38.17	57.05	-0.42	16.7	-37.12	50.11
-179.39	24.19	0.39	-16.38	-39.56	37.26	-0.44	17.13	-36.12	41.61
-179.38	24.21	0.39	-16.38	-39.56	57.04	-0.44	17.13	-36.12	52.86
-179.38	24.31	0.47	-19.71	-39.03	34.26	-0.42	16.7	-37.12	43.93
-179.35	24.88	0.46	-18.76	-38.17	57.05	-0.42	16.7	-37.12	50.11
-179.6	25.91	0.39	-16.38	-39.56	37.26	-0.44	17.13	-36.12	41.61
-179.46	25.98	0.39	-16.38	-39.56	57.04	-0.44	17.13	-36.12	52.86
-179.69	26.19	0.47	-19.71	-39.03	34.26	-0.42	16.7	-37.12	43.93
-179.51	26.33	0.46	-18.76	-38.17	57.05	-0.42	16.7	-37.12	50.11
-179.31	26.73	0.39	-16.38	-39.56	37.26	-0.44	17.13	-36.12	41.61
-179.31	27.06	0.39	-16.38	-39.56	57.04	-0.44	17.13	-36.12	52.86
-179.27	27.53	0.47	-19.71	-39.03	34.26	-0.42	16.7	-37.12	43.93
-179.26	27.99	0.46	-18.76	-38.17	57.05	-0.42	16.7	-37.12	50.11
-179.24	28.46	0.39	-16.38	-39.56	37.26	-0.44	17.13	-36.12	41.61
-179.37	29.06	0.39	-16.38	-39.56	57.04	-0.44	17.13	-36.12	52.86
-179.37	29.29	0.47	-19.71	-39.03	34.26	-0.42	16.7	-37.12	43.93
-179.22	29.44	0.46	-18.76	-38.17	57.05	-0.42	16.7	-37.12	50.11
-179.33	29.79	0.39	-16.38	-39.56	37.26	-0.44	17.13	-36.12	41.61
-179.33	29.91	0.39	-16.38	-39.56	57.04	-0.44	17.13	-36.12	52.86
-179.16	30.02	0.47	-19.71	-39.03	34.26	-0.42	16.7	-37.12	43.93

Turtle		SPA120				SPA60			
Y	X	Rot ang	Z	Y	X	Rot ang	Z	Y	X
-179.07	30.83	0.46	-18.76	-38.17	57.05	-0.42	16.7	-37.12	50.11
-179.05	30.8	0.39	-16.38	-39.56	37.26	-0.44	17.13	-36.12	41.61
-178.96	31.39	0.39	-16.38	-39.56	57.04	-0.44	17.13	-36.12	52.86
-178.92	31.95	0.47	-19.71	-39.03	34.26	-0.42	16.7	-37.12	43.93
-178.84	32.62	0.46	-18.76	-38.17	57.05	-0.42	16.7	-37.12	50.11
-178.8	33.48	0.39	-16.38	-39.56	37.26	-0.44	17.13	-36.12	41.61
-178.78	33.98	0.39	-16.38	-39.56	57.04	-0.44	17.13	-36.12	52.86
-178.74	35.41	0.47	-19.71	-39.03	34.26	-0.42	16.7	-37.12	43.93
-178.73	36.05	0.46	-18.76	-38.17	57.05	-0.42	16.7	-37.12	50.11
-178.71	36.52	0.39	-16.38	-39.56	37.26	-0.44	17.13	-36.12	41.61
-178.66	37.26	0.39	-16.38	-39.56	57.04	-0.44	17.13	-36.12	52.86
-178.76	37.87	0.47	-19.71	-39.03	34.26	-0.42	16.7	-37.12	43.93
-178.76	38.12	0.46	-18.76	-38.17	57.05	-0.42	16.7	-37.12	50.11
-178.7	38.13	0.39	-16.38	-39.56	37.26	-0.44	17.13	-36.12	41.61
-178.91	38.36	0.39	-16.38	-39.56	57.04	-0.44	17.13	-36.12	52.86
-178.93	38.72	0.47	-19.71	-39.03	34.26	-0.42	16.7	-37.12	43.93
-178.8	39.07	0.46	-18.76	-38.17	57.05	-0.42	16.7	-37.12	50.11
-178.68	39.25	0.39	-16.38	-39.56	37.26	-0.44	17.13	-36.12	41.61
-178.62	39.87	0.39	-16.38	-39.56	57.04	-0.44	17.13	-36.12	52.86
-179.32	41.96	0.47	-19.71	-39.03	34.26	-0.42	16.7	-37.12	43.93
-179.38	43.54	0.46	-18.76	-38.17	57.05	-0.42	16.7	-37.12	50.11
-180	45.04	0.39	-16.38	-39.56	37.26	-0.44	17.13	-36.12	41.61
-180.8	45.98	0.39	-16.38	-39.56	57.04	-0.44	17.13	-36.12	52.86
-180.2	45.91	0.47	-19.71	-39.03	34.26	-0.42	16.7	-37.12	43.93
-180.39	45.89	0.46	-18.76	-38.17	57.05	-0.42	16.7	-37.12	50.11
-180.24	45.81	0.39	-16.38	-39.56	37.26	-0.44	17.13	-36.12	41.61
-180.48	45.95	0.39	-16.38	-39.56	57.04	-0.44	17.13	-36.12	52.86
-180.26	46.16	0.47	-19.71	-39.03	34.26	-0.42	16.7	-37.12	43.93
-180.42	46.65	0.46	-18.76	-38.17	57.05	-0.42	16.7	-37.12	50.11
-180.26	46.85	0.39	-16.38	-39.56	37.26	-0.44	17.13	-36.12	41.61
-180.08	47.37	0.39	-16.38	-39.56	57.04	-0.44	17.13	-36.12	52.86
-180.38	48.16	0.47	-19.71	-39.03	34.26	-0.42	16.7	-37.12	43.93
-180.64	48.44	0.46	-18.76	-38.17	57.05	-0.42	16.7	-37.12	50.11
-180.73	48.86	0.39	-16.38	-39.56	37.26	-0.44	17.13	-36.12	41.61
-180.76	48.71	0.39	-16.38	-39.56	57.04	-0.44	17.13	-36.12	52.86
-180.76	49.19	0.47	-19.71	-39.03	34.26	-0.42	16.7	-37.12	43.93
-180.82	49.52	0.46	-18.76	-38.17	57.05	-0.42	16.7	-37.12	50.11
-180.77	50.09	0.39	-16.38	-39.56	37.26	-0.44	17.13	-36.12	41.61
-180.75	50.31	0.39	-16.38	-39.56	57.04	-0.44	17.13	-36.12	52.86
-180.81	50.48	0.47	-19.71	-39.03	34.26	-0.42	16.7	-37.12	43.93
-180.81	50.65	0.46	-18.76	-38.17	57.05	-0.42	16.7	-37.12	50.11
-180.85	50.8	0.39	-16.38	-39.56	37.26	-0.44	17.13	-36.12	41.61
-180.76	51.06	0.39	-16.38	-39.56	57.04	-0.44	17.13	-36.12	52.86
-180.84	51.36	0.47	-19.71	-39.03	34.26	-0.42	16.7	-37.12	43.93

Turtle		SPA120				SPA60			
Y	X	Rot ang	Z	Y	X	Rot ang	Z	Y	X
-180.68	51.35	0.46	-18.76	-38.17	57.05	-0.42	16.7	-37.12	50.11
-180.61	52.17	0.39	-16.38	-39.56	37.26	-0.44	17.13	-36.12	41.61
-180.6	52.5	0.39	-16.38	-39.56	57.04	-0.44	17.13	-36.12	52.86
-180.53	53.15	0.47	-19.71	-39.03	34.26	-0.42	16.7	-37.12	43.93
-180.49	53.94	0.46	-18.76	-38.17	57.05	-0.42	16.7	-37.12	50.11
-180.5	53.99	0.39	-16.38	-39.56	37.26	-0.44	17.13	-36.12	41.61
-180.46	54.64	0.39	-16.38	-39.56	57.04	-0.44	17.13	-36.12	52.86
-180.44	55.23	0.47	-19.71	-39.03	34.26	-0.42	16.7	-37.12	43.93
-180.4	55.94	0.46	-18.76	-38.17	57.05	-0.42	16.7	-37.12	50.11
-180.35	56.75	0.39	-16.38	-39.56	37.26	-0.44	17.13	-36.12	41.61
-180.35	57.02	0.39	-16.38	-39.56	57.04	-0.44	17.13	-36.12	52.86
-180.34	58.11	0.47	-19.71	-39.03	34.26	-0.42	16.7	-37.12	43.93
-180.37	58.58	0.46	-18.76	-38.17	57.05	-0.42	16.7	-37.12	50.11
-180.47	58.72	0.39	-16.38	-39.56	37.26	-0.44	17.13	-36.12	41.61
-180.43	58.79	0.39	-16.38	-39.56	57.04	-0.44	17.13	-36.12	52.86
-180.56	58.93	0.47	-19.71	-39.03	34.26	-0.42	16.7	-37.12	43.93
-180.47	58.98	0.46	-18.76	-38.17	57.05	-0.42	16.7	-37.12	50.11
-180.38	58.88	0.39	-16.38	-39.56	37.26	-0.44	17.13	-36.12	41.61
-180.3	58.83	0.39	-16.38	-39.56	57.04	-0.44	17.13	-36.12	52.86
-180.28	58.78	0.47	-19.71	-39.03	34.26	-0.42	16.7	-37.12	43.93
-180.25	58.77	0.46	-18.76	-38.17	57.05	-0.42	16.7	-37.12	50.11
-180.23	58.73	0.39	-16.38	-39.56	37.26	-0.44	17.13	-36.12	41.61
-180.23	58.73	0.39	-16.38	-39.56	57.04	-0.44	17.13	-36.12	52.86
-180.24	58.73	0.47	-19.71	-39.03	34.26	-0.42	16.7	-37.12	43.93
-180.3	58.75	0.46	-18.76	-38.17	57.05	-0.42	16.7	-37.12	50.11
-180.19	58.55	0.39	-16.38	-39.56	37.26	-0.44	17.13	-36.12	41.61
-180.24	58.66	0.39	-16.38	-39.56	57.04	-0.44	17.13	-36.12	52.86
-180.25	58.74	0.47	-19.71	-39.03	34.26	-0.42	16.7	-37.12	43.93
-180.27	59.4	0.46	-18.76	-38.17	57.05	-0.42	16.7	-37.12	50.11
-180.25	59.67	0.39	-16.38	-39.56	37.26	-0.44	17.13	-36.12	41.61
-180.24	59.66	0.39	-16.38	-39.56	57.04	-0.44	17.13	-36.12	52.86
-180.21	60.16	0.47	-19.71	-39.03	34.26	-0.42	16.7	-37.12	43.93
-180.25	60.64	0.46	-18.76	-38.17	57.05	-0.42	16.7	-37.12	50.11
-180.19	60.81	0.39	-16.38	-39.56	37.26	-0.44	17.13	-36.12	41.61
-180.15	61.36	0.39	-16.38	-39.56	57.04	-0.44	17.13	-36.12	52.86
-180.14	61.69	0.47	-19.71	-39.03	34.26	-0.42	16.7	-37.12	43.93
-180.16	62.16	0.46	-18.76	-38.17	57.05	-0.42	16.7	-37.12	50.11
-180.16	62.44	0.39	-16.38	-39.56	37.26	-0.44	17.13	-36.12	41.61
-180.19	62.86	0.39	-16.38	-39.56	57.04	-0.44	17.13	-36.12	52.86
-180.1	63.06	0.47	-19.71	-39.03	34.26	-0.42	16.7	-37.12	43.93
-180.29	63.4	0.46	-18.76	-38.17	57.05	-0.42	16.7	-37.12	50.11
-180.17	63.53	0.39	-16.38	-39.56	37.26	-0.44	17.13	-36.12	41.61
-180.12	63.7	0.39	-16.38	-39.56	57.04	-0.44	17.13	-36.12	52.86
-180.02	63.94	0.47	-19.71	-39.03	34.26	-0.42	16.7	-37.12	43.93

Turtle		SPA120				SPA60			
Y	X	Rot ang	Z	Y	X	Rot ang	Z	Y	X
-180.01	64.22	0.46	-18.76	-38.17	57.05	-0.42	16.7	-37.12	50.11
-179.99	64.25	0.39	-16.38	-39.56	37.26	-0.44	17.13	-36.12	41.61
-179.96	64.33	0.39	-16.38	-39.56	57.04	-0.44	17.13	-36.12	52.86
-179.88	65.13	0.47	-19.71	-39.03	34.26	-0.42	16.7	-37.12	43.93
-179.87	65.38	0.46	-18.76	-38.17	57.05	-0.42	16.7	-37.12	50.11
-179.81	66.09	0.39	-16.38	-39.56	37.26	-0.44	17.13	-36.12	41.61
-179.76	66.83	0.39	-16.38	-39.56	57.04	-0.44	17.13	-36.12	52.86
-179.74	67.21	0.47	-19.71	-39.03	34.26	-0.42	16.7	-37.12	43.93
-179.7	67.95	0.46	-18.76	-38.17	57.05	-0.42	16.7	-37.12	50.11
-179.65	68.73	0.39	-16.38	-39.56	37.26	-0.44	17.13	-36.12	41.61
-179.64	69.21	0.39	-16.38	-39.56	57.04	-0.44	17.13	-36.12	52.86
-179.61	69.79	0.47	-19.71	-39.03	34.26	-0.42	16.7	-37.12	43.93
-179.57	70.53	0.46	-18.76	-38.17	57.05	-0.42	16.7	-37.12	50.11
-179.58	70.8	0.39	-16.38	-39.56	37.26	-0.44	17.13	-36.12	41.61
-179.57	71.12	0.39	-16.38	-39.56	57.04	-0.44	17.13	-36.12	52.86
-179.62	71.55	0.47	-19.71	-39.03	34.26	-0.42	16.7	-37.12	43.93
-179.59	71.63	0.46	-18.76	-38.17	57.05	-0.42	16.7	-37.12	50.11
-179.67	71.72	0.39	-16.38	-39.56	37.26	-0.44	17.13	-36.12	41.61
-179.94	71.99	0.39	-16.38	-39.56	57.04	-0.44	17.13	-36.12	52.86
-180.04	71.81	0.47	-19.71	-39.03	34.26	-0.42	16.7	-37.12	43.93
-180.29	71.8	0.46	-18.76	-38.17	57.05	-0.42	16.7	-37.12	50.11
-180.35	71.78	0.39	-16.38	-39.56	37.26	-0.44	17.13	-36.12	41.61
-180.32	71.75	0.39	-16.38	-39.56	57.04	-0.44	17.13	-36.12	52.86
-180.18	71.72	0.47	-19.71	-39.03	34.26	-0.42	16.7	-37.12	43.93
-179.93	71.78	0.46	-18.76	-38.17	57.05	-0.42	16.7	-37.12	50.11
-179.76	71.75	0.39	-16.38	-39.56	37.26	-0.44	17.13	-36.12	41.61
-179.54	71.61	0.39	-16.38	-39.56	57.04	-0.44	17.13	-36.12	52.86
-180.07	71.61	0.47	-19.71	-39.03	34.26	-0.42	16.7	-37.12	43.93
-180.43	71.76	0.46	-18.76	-38.17	57.05	-0.42	16.7	-37.12	50.11
-180.5	71.88	0.39	-16.38	-39.56	37.26	-0.44	17.13	-36.12	41.61
-180.47	72.67	0.39	-16.38	-39.56	57.04	-0.44	17.13	-36.12	52.86
-180.39	73.23	0.47	-19.71	-39.03	34.26	-0.42	16.7	-37.12	43.93
-180.34	73.6	0.46	-18.76	-38.17	57.05	-0.42	16.7	-37.12	50.11
-180.34	73.59	0.39	-16.38	-39.56	37.26	-0.44	17.13	-36.12	41.61
-180.38	73.83	0.39	-16.38	-39.56	57.04	-0.44	17.13	-36.12	52.86
-180.33	74.55	0.47	-19.71	-39.03	34.26	-0.42	16.7	-37.12	43.93
-180.48	74.87	0.46	-18.76	-38.17	57.05	-0.42	16.7	-37.12	50.11
-180.35	75.25	0.39	-16.38	-39.56	37.26	-0.44	17.13	-36.12	41.61
-180.38	75.55	0.39	-16.38	-39.56	57.04	-0.44	17.13	-36.12	52.86
-180.43	76.18	0.47	-19.71	-39.03	34.26	-0.42	16.7	-37.12	43.93
-180.34	76.25	0.46	-18.76	-38.17	57.05	-0.42	16.7	-37.12	50.11
-180.3	76.41	0.39	-16.38	-39.56	37.26	-0.44	17.13	-36.12	41.61
-180.3	76.57	0.39	-16.38	-39.56	57.04	-0.44	17.13	-36.12	52.86
-180.21	76.84	0.47	-19.71	-39.03	34.26	-0.42	16.7	-37.12	43.93

Turtle		SPA120				SPA60			
Y	X	Rot ang	Z	Y	X	Rot ang	Z	Y	X
-180.22	77.25	0.46	-18.76	-38.17	57.05	-0.42	16.7	-37.12	50.11
-180.27	77.33	0.39	-16.38	-39.56	37.26	-0.44	17.13	-36.12	41.61
-180.26	77.43	0.39	-16.38	-39.56	57.04	-0.44	17.13	-36.12	52.86
-180.24	77.46	0.47	-19.71	-39.03	34.26	-0.42	16.7	-37.12	43.93
-180.12	78.13	0.46	-18.76	-38.17	57.05	-0.42	16.7	-37.12	50.11
-180.12	78.49	0.39	-16.38	-39.56	37.26	-0.44	17.13	-36.12	41.61
-180.04	79.16	0.39	-16.38	-39.56	57.04	-0.44	17.13	-36.12	52.86
-179.92	79.91	0.47	-19.71	-39.03	34.26	-0.42	16.7	-37.12	43.93
-179.88	80.41	0.46	-18.76	-38.17	57.05	-0.42	16.7	-37.12	50.11
-179.86	81.04	0.39	-16.38	-39.56	37.26	-0.44	17.13	-36.12	41.61
-179.8	81.81	0.39	-16.38	-39.56	57.04	-0.44	17.13	-36.12	52.86
-179.85	82.95	0.47	-19.71	-39.03	34.26	-0.42	16.7	-37.12	43.93
-179.81	83.6	0.46	-18.76	-38.17	57.05	-0.42	16.7	-37.12	50.11
-179.8	83.67	0.39	-16.38	-39.56	37.26	-0.44	17.13	-36.12	41.61
-179.86	83.98	0.39	-16.38	-39.56	57.04	-0.44	17.13	-36.12	52.86
-179.76	84.57	0.47	-19.71	-39.03	34.26	-0.42	16.7	-37.12	43.93
-179.86	84.8	0.46	-18.76	-38.17	57.05	-0.42	16.7	-37.12	50.11
-179.81	84.79	0.39	-16.38	-39.56	37.26	-0.44	17.13	-36.12	41.61
-179.79	84.9	0.39	-16.38	-39.56	57.04	-0.44	17.13	-36.12	52.86
-179.83	84.95	0.47	-19.71	-39.03	34.26	-0.42	16.7	-37.12	43.93
-179.98	84.94	0.46	-18.76	-38.17	57.05	-0.42	16.7	-37.12	50.11
-180.42	84.96	0.39	-16.38	-39.56	37.26	-0.44	17.13	-36.12	41.61
-180.6	84.89	0.39	-16.38	-39.56	57.04	-0.44	17.13	-36.12	52.86
-180.61	84.84	0.47	-19.71	-39.03	34.26	-0.42	16.7	-37.12	43.93
-180.52	84.8	0.46	-18.76	-38.17	57.05	-0.42	16.7	-37.12	50.11
-180.57	84.79	0.39	-16.38	-39.56	37.26	-0.44	17.13	-36.12	41.61
-180.36	84.74	0.39	-16.38	-39.56	57.04	-0.44	17.13	-36.12	52.86
-179.79	84.59	0.47	-19.71	-39.03	34.26	-0.42	16.7	-37.12	43.93
-179.86	84.66	0.46	-18.76	-38.17	57.05	-0.42	16.7	-37.12	50.11
-179.84	84.75	0.39	-16.38	-39.56	37.26	-0.44	17.13	-36.12	41.61
-180.48	85.51	0.39	-16.38	-39.56	57.04	-0.44	17.13	-36.12	52.86
-180.63	85.85	0.47	-19.71	-39.03	34.26	-0.42	16.7	-37.12	43.93
-180.65	86.48	0.46	-18.76	-38.17	57.05	-0.42	16.7	-37.12	50.11
-180.86	86.83	0.39	-16.38	-39.56	37.26	-0.44	17.13	-36.12	41.61
-180.65	87.38	0.39	-16.38	-39.56	57.04	-0.44	17.13	-36.12	52.86
-180.55	87.61	0.47	-19.71	-39.03	34.26	-0.42	16.7	-37.12	43.93
-180.49	88.19	0.46	-18.76	-38.17	57.05	-0.42	16.7	-37.12	50.11
-180.48	88.31	0.39	-16.38	-39.56	37.26	-0.44	17.13	-36.12	41.61
-180.54	88.57	0.39	-16.38	-39.56	57.04	-0.44	17.13	-36.12	52.86
-180.55	88.78	0.47	-19.71	-39.03	34.26	-0.42	16.7	-37.12	43.93
-180.59	89.15	0.46	-18.76	-38.17	57.05	-0.42	16.7	-37.12	50.11
-181.28	89.71	0.39	-16.38	-39.56	37.26	-0.44	17.13	-36.12	41.61
-181.31	89.65	0.39	-16.38	-39.56	57.04	-0.44	17.13	-36.12	52.86
-181.26	90.02	0.47	-19.71	-39.03	34.26	-0.42	16.7	-37.12	43.93

Turtle		SPA120				SPA60			
Y	X	Rot ang	Z	Y	X	Rot ang	Z	Y	X
-181.27	90.35	0.46	-18.76	-38.17	57.05	-0.42	16.7	-37.12	50.11
-181.22	90.39	0.39	-16.38	-39.56	37.26	-0.44	17.13	-36.12	41.61
-181.22	90.49	0.39	-16.38	-39.56	57.04	-0.44	17.13	-36.12	52.86
-181.21	90.53	0.47	-19.71	-39.03	34.26	-0.42	16.7	-37.12	43.93
-181.17	91.22	0.46	-18.76	-38.17	57.05	-0.42	16.7	-37.12	50.11
-181.19	91.52	0.39	-16.38	-39.56	37.26	-0.44	17.13	-36.12	41.61
-181.2	91.43	0.39	-16.38	-39.56	57.04	-0.44	17.13	-36.12	52.86
-181.18	92.2	0.47	-19.71	-39.03	34.26	-0.42	16.7	-37.12	43.93
-181.18	92.57	0.46	-18.76	-38.17	57.05	-0.42	16.7	-37.12	50.11
-181.12	93.18	0.39	-16.38	-39.56	37.26	-0.44	17.13	-36.12	41.61
-181.04	94.01	0.39	-16.38	-39.56	57.04	-0.44	17.13	-36.12	52.86
-180.86	95.16	0.47	-19.71	-39.03	34.26	-0.42	16.7	-37.12	43.93
-180.84	95.85	0.46	-18.76	-38.17	57.05	-0.42	16.7	-37.12	50.11
-180.88	96.19	0.39	-16.38	-39.56	37.26	-0.44	17.13	-36.12	41.61
-180.88	96.76	0.39	-16.38	-39.56	57.04	-0.44	17.13	-36.12	52.86
-180.94	96.95	0.47	-19.71	-39.03	34.26	-0.42	16.7	-37.12	43.93
-181	97.61	0.46	-18.76	-38.17	57.05	-0.42	16.7	-37.12	50.11
-180.95	97.67	0.39	-16.38	-39.56	37.26	-0.44	17.13	-36.12	41.61
-181.08	97.77	0.39	-16.38	-39.56	57.04	-0.44	17.13	-36.12	52.86
-180.98	97.84	0.47	-19.71	-39.03	34.26	-0.42	16.7	-37.12	43.93
-181.05	97.91	0.46	-18.76	-38.17	57.05	-0.42	16.7	-37.12	50.11
-180.98	97.93	0.39	-16.38	-39.56	37.26	-0.44	17.13	-36.12	41.61
-180.93	97.96	0.39	-16.38	-39.56	57.04	-0.44	17.13	-36.12	52.86
-180.91	97.89	0.47	-19.71	-39.03	34.26	-0.42	16.7	-37.12	43.93
-180.9	97.86	0.46	-18.76	-38.17	57.05	-0.42	16.7	-37.12	50.11
-181	97.91	0.39	-16.38	-39.56	37.26	-0.44	17.13	-36.12	41.61
-180.91	97.82	0.39	-16.38	-39.56	57.04	-0.44	17.13	-36.12	52.86
-180.85	97.71	0.47	-19.71	-39.03	34.26	-0.42	16.7	-37.12	43.93
-180.88	97.75	0.46	-18.76	-38.17	57.05	-0.42	16.7	-37.12	50.11
-180.87	97.76	0.39	-16.38	-39.56	37.26	-0.44	17.13	-36.12	41.61
-180.88	97.83	0.39	-16.38	-39.56	57.04	-0.44	17.13	-36.12	52.86
-180.89	98.11	0.47	-19.71	-39.03	34.26	-0.42	16.7	-37.12	43.93
-180.96	98.68	0.46	-18.76	-38.17	57.05	-0.42	16.7	-37.12	50.11
-181.52	98.95	0.39	-16.38	-39.56	37.26	-0.44	17.13	-36.12	41.61
-181.59	99.52	0.39	-16.38	-39.56	57.04	-0.44	17.13	-36.12	52.86
-181.73	99.8	0.47	-19.71	-39.03	34.26	-0.42	16.7	-37.12	43.93
-181.69	100.44	0.46	-18.76	-38.17	57.05	-0.42	16.7	-37.12	50.11
-181.72	100.69	0.39	-16.38	-39.56	37.26	-0.44	17.13	-36.12	41.61
-181.74	101.3	0.39	-16.38	-39.56	57.04	-0.44	17.13	-36.12	52.86
-181.71	101.65	0.47	-19.71	-39.03	34.26	-0.42	16.7	-37.12	43.93
-181.71	101.91	0.46	-18.76	-38.17	57.05	-0.42	16.7	-37.12	50.11
-182.06	102.42	0.39	-16.38	-39.56	37.26	-0.44	17.13	-36.12	41.61
-182.25	102.45	0.39	-16.38	-39.56	57.04	-0.44	17.13	-36.12	52.86
-182.42	102.6	0.47	-19.71	-39.03	34.26	-0.42	16.7	-37.12	43.93

Turtle		SPA120				SPA60			
**Y**	**X**	**Rot ang**	**Z**	**Y**	**X**	**Rot ang**	**Z**	**Y**	**X**
-182.44	102.67	0.46	-18.76	-38.17	57.05	-0.42	16.7	-37.12	50.11
-182.46	102.86	0.39	-16.38	-39.56	37.26	-0.44	17.13	-36.12	41.61
-182.49	102.96	0.39	-16.38	-39.56	57.04	-0.44	17.13	-36.12	52.86
-182.44	103.12	0.47	-19.71	-39.03	34.26	-0.42	16.7	-37.12	43.93
-182.43	103.45	0.46	-18.76	-38.17	57.05	-0.42	16.7	-37.12	50.11
-182.41	103.46	0.39	-16.38	-39.56	37.26	-0.44	17.13	-36.12	41.61
-182.33	104.22	0.39	-16.38	-39.56	57.04	-0.44	17.13	-36.12	52.86
-182.32	104.55	0.47	-19.71	-39.03	34.26	-0.42	16.7	-37.12	43.93
-182.26	105.7	0.46	-18.76	-38.17	57.05	-0.42	16.7	-37.12	50.11
-182.22	106.26	0.39	-16.38	-39.56	37.26	-0.44	17.13	-36.12	41.61
-182.23	106.24	0.39	-16.38	-39.56	57.04	-0.44	17.13	-36.12	52.86
-182.16	107.05	0.47	-19.71	-39.03	34.26	-0.42	16.7	-37.12	43.93
-182.09	107.79	0.46	-18.76	-38.17	57.05	-0.42	16.7	-37.12	50.11
-182.08	108.26	0.39	-16.38	-39.56	37.26	-0.44	17.13	-36.12	41.61
-182.03	108.96	0.39	-16.38	-39.56	57.04	-0.44	17.13	-36.12	52.86
-182.01	109.42	0.47	-19.71	-39.03	34.26	-0.42	16.7	-37.12	43.93
-182	109.89	0.46	-18.76	-38.17	57.05	-0.42	16.7	-37.12	50.11
-182.03	110.12	0.39	-16.38	-39.56	37.26	-0.44	17.13	-36.12	41.61
-182.08	110.53	0.39	-16.38	-39.56	57.04	-0.44	17.13	-36.12	52.86
-182.08	110.81	0.47	-19.71	-39.03	34.26	-0.42	16.7	-37.12	43.93
-182.1	110.87	0.46	-18.76	-38.17	57.05	-0.42	16.7	-37.12	50.11
-182.2	111.02	0.39	-16.38	-39.56	37.26	-0.44	17.13	-36.12	41.61
-182.13	110.96	0.39	-16.38	-39.56	57.04	-0.44	17.13	-36.12	52.86
-182.08	111.02	0.47	-19.71	-39.03	34.26	-0.42	16.7	-37.12	43.93
-182.1	111.01	0.46	-18.76	-38.17	57.05	-0.42	16.7	-37.12	50.11
-182.07	110.96	0.39	-16.38	-39.56	37.26	-0.44	17.13	-36.12	41.61
-182.07	110.93	0.39	-16.38	-39.56	57.04	-0.44	17.13	-36.12	52.86
-182.06	110.91	0.47	-19.71	-39.03	34.26	-0.42	16.7	-37.12	43.93
-181.97	110.82	0.46	-18.76	-38.17	57.05	-0.42	16.7	-37.12	50.11
-181.95	110.78	0.39	-16.38	-39.56	37.26	-0.44	17.13	-36.12	41.61
-181.98	110.86	0.39	-16.38	-39.56	57.04	-0.44	17.13	-36.12	52.86
-182.01	111	0.47	-19.71	-39.03	34.26	-0.42	16.7	-37.12	43.93
-182.05	111.68	0.46	-18.76	-38.17	57.05	-0.42	16.7	-37.12	50.11
-182.22	112.05	0.39	-16.38	-39.56	37.26	-0.44	17.13	-36.12	41.61
-182.12	111.94	0.39	-16.38	-39.56	57.04	-0.44	17.13	-36.12	52.86
-182.02	112.51	0.47	-19.71	-39.03	34.26	-0.42	16.7	-37.12	43.93
-182.35	112.91	0.46	-18.76	-38.17	57.05	-0.42	16.7	-37.12	50.11
-182.34	113.41	0.39	-16.38	-39.56	37.26	-0.44	17.13	-36.12	41.61
-182.65	113.74	0.39	-16.38	-39.56	57.04	-0.44	17.13	-36.12	52.86
-182.72	114.39	0.47	-19.71	-39.03	34.26	-0.42	16.7	-37.12	43.93
-182.74	114.62	0.46	-18.76	-38.17	57.05	-0.42	16.7	-37.12	50.11
-182.79	114.98	0.39	-16.38	-39.56	37.26	-0.44	17.13	-36.12	41.61
-182.84	115.61	0.39	-16.38	-39.56	57.04	-0.44	17.13	-36.12	52.86
-182.78	115.62	0.47	-19.71	-39.03	34.26	-0.42	16.7	-37.12	43.93

Turtle		SPA120				SPA60			
Y	X	Rot ang	Z	Y	X	Rot ang	Z	Y	X
-182.82	115.89	0.46	-18.76	-38.17	57.05	-0.42	16.7	-37.12	50.11
-182.76	116.04	0.39	-16.38	-39.56	37.26	-0.44	17.13	-36.12	41.61
-182.7	116.24	0.39	-16.38	-39.56	57.04	-0.44	17.13	-36.12	52.86
-182.7	116.43	0.47	-19.71	-39.03	34.26	-0.42	16.7	-37.12	43.93
-182.71	116.5	0.46	-18.76	-38.17	57.05	-0.42	16.7	-37.12	50.11
-182.69	116.53	0.39	-16.38	-39.56	37.26	-0.44	17.13	-36.12	41.61
-182.65	116.61	0.39	-16.38	-39.56	57.04	-0.44	17.13	-36.12	52.86
-182.5	117.58	0.47	-19.71	-39.03	34.26	-0.42	16.7	-37.12	43.93
-182.39	118.23	0.46	-18.76	-38.17	57.05	-0.42	16.7	-37.12	50.11
-182.4	118.51	0.39	-16.38	-39.56	37.26	-0.44	17.13	-36.12	41.61
-182.37	119.23	0.39	-16.38	-39.56	57.04	-0.44	17.13	-36.12	52.86
-182.29	120.03	0.47	-19.71	-39.03	34.26	-0.42	16.7	-37.12	43.93
-182.26	120.48	0.46	-18.76	-38.17	57.05	-0.42	16.7	-37.12	50.11
-182.24	121.12	0.39	-16.38	-39.56	37.26	-0.44	17.13	-36.12	41.61
-182.28	121.11	0.39	-16.38	-39.56	57.04	-0.44	17.13	-36.12	52.86
-182.25	121.74	0.47	-19.71	-39.03	34.26	-0.42	16.7	-37.12	43.93
-182.25	122.16	0.46	-18.76	-38.17	57.05	-0.42	16.7	-37.12	50.11
-182.21	123.05	0.39	-16.38	-39.56	37.26	-0.44	17.13	-36.12	41.61
-182.31	123.33	0.39	-16.38	-39.56	57.04	-0.44	17.13	-36.12	52.86
-182.43	123.52	0.47	-19.71	-39.03	34.26	-0.42	16.7	-37.12	43.93
-182.36	123.68	0.46	-18.76	-38.17	57.05	-0.42	16.7	-37.12	50.11
-182.53	123.81	0.39	-16.38	-39.56	37.26	-0.44	17.13	-36.12	41.61
-182.37	123.83	0.39	-16.38	-39.56	57.04	-0.44	17.13	-36.12	52.86
-182.34	123.88	0.47	-19.71	-39.03	34.26	-0.42	16.7	-37.12	43.93
-182.29	123.88	0.46	-18.76	-38.17	57.05	-0.42	16.7	-37.12	50.11
-182.26	123.94	0.39	-16.38	-39.56	37.26	-0.44	17.13	-36.12	41.61
-182.23	123.92	0.39	-16.38	-39.56	57.04	-0.44	17.13	-36.12	52.86
-182.22	123.9	0.47	-19.71	-39.03	34.26	-0.42	16.7	-37.12	43.93
-182.21	123.9	0.46	-18.76	-38.17	57.05	-0.42	16.7	-37.12	50.11
-182.21	123.87	0.39	-16.38	-39.56	37.26	-0.44	17.13	-36.12	41.61
-182.08	123.69	0.39	-16.38	-39.56	57.04	-0.44	17.13	-36.12	52.86
-182.19	123.76	0.47	-19.71	-39.03	34.26	-0.42	16.7	-37.12	43.93
-182.2	123.87	0.46	-18.76	-38.17	57.05	-0.42	16.7	-37.12	50.11
-182.19	123.97	0.39	-16.38	-39.56	37.26	-0.44	17.13	-36.12	41.61
-182.15	124.56	0.39	-16.38	-39.56	57.04	-0.44	17.13	-36.12	52.86
-182.17	124.84	0.47	-19.71	-39.03	34.26	-0.42	16.7	-37.12	43.93
-182.11	125.44	0.46	-18.76	-38.17	57.05	-0.42	16.7	-37.12	50.11
-182.09	126.27	0.39	-16.38	-39.56	37.26	-0.44	17.13	-36.12	41.61
-182.07	126.46	0.39	-16.38	-39.56	57.04	-0.44	17.13	-36.12	52.86
-182.05	126.69	0.47	-19.71	-39.03	34.26	-0.42	16.7	-37.12	43.93
-182	127.27	0.46	-18.76	-38.17	57.05	-0.42	16.7	-37.12	50.11
-182.02	127.62	0.39	-16.38	-39.56	37.26	-0.44	17.13	-36.12	41.61
-182.04	127.76	0.39	-16.38	-39.56	57.04	-0.44	17.13	-36.12	52.86
-182.03	128.36	0.47	-19.71	-39.03	34.26	-0.42	16.7	-37.12	43.93

Turtle		SPA120				SPA60			
Y	X	Rot ang	Z	Y	X	Rot ang	Z	Y	X
-182.07	128.64	0.46	-18.76	-38.17	57.05	-0.42	16.7	-37.12	50.11
-182.03	128.83	0.39	-16.38	-39.56	37.26	-0.44	17.13	-36.12	41.61
-181.94	129.2	0.39	-16.38	-39.56	57.04	-0.44	17.13	-36.12	52.86
-181.92	129.31	0.47	-19.71	-39.03	34.26	-0.42	16.7	-37.12	43.93
-181.92	129.39	0.46	-18.76	-38.17	57.05	-0.42	16.7	-37.12	50.11
-181.92	129.49	0.39	-16.38	-39.56	37.26	-0.44	17.13	-36.12	41.61
-182.05	129.64	0.39	-16.38	-39.56	57.04	-0.44	17.13	-36.12	52.86
-181.96	129.58	0.47	-19.71	-39.03	34.26	-0.42	16.7	-37.12	43.93
-181.84	130.23	0.46	-18.76	-38.17	57.05	-0.42	16.7	-37.12	50.11
-181.74	130.6	0.39	-16.38	-39.56	37.26	-0.44	17.13	-36.12	41.61
-181.73	131.23	0.39	-16.38	-39.56	57.04	-0.44	17.13	-36.12	52.86
-181.58	131.72	0.47	-19.71	-39.03	34.26	-0.42	16.7	-37.12	43.93
-181.56	132.24	0.46	-18.76	-38.17	57.05	-0.42	16.7	-37.12	50.11
-181.5	132.87	0.39	-16.38	-39.56	37.26	-0.44	17.13	-36.12	41.61
-181.53	133.33	0.39	-16.38	-39.56	57.04	-0.44	17.13	-36.12	52.86
-181.53	134.05	0.47	-19.71	-39.03	34.26	-0.42	16.7	-37.12	43.93
-181.52	135.01	0.46	-18.76	-38.17	57.05	-0.42	16.7	-37.12	50.11
-181.47	135.51	0.39	-16.38	-39.56	37.26	-0.44	17.13	-36.12	41.61
-181.48	135.72	0.39	-16.38	-39.56	57.04	-0.44	17.13	-36.12	52.86
-181.48	135.99	0.47	-19.71	-39.03	34.26	-0.42	16.7	-37.12	43.93
-181.55	136.34	0.46	-18.76	-38.17	57.05	-0.42	16.7	-37.12	50.11
-181.54	136.59	0.39	-16.38	-39.56	37.26	-0.44	17.13	-36.12	41.61
-181.52	136.7	0.39	-16.38	-39.56	57.04	-0.44	17.13	-36.12	52.86
-181.62	136.81	0.47	-19.71	-39.03	34.26	-0.42	16.7	-37.12	43.93
-181.59	136.82	0.46	-18.76	-38.17	57.05	-0.42	16.7	-37.12	50.11
-181.59	136.9	0.39	-16.38	-39.56	37.26	-0.44	17.13	-36.12	41.61
-181.57	136.89	0.39	-16.38	-39.56	57.04	-0.44	17.13	-36.12	52.86
-181.56	136.9	0.47	-19.71	-39.03	34.26	-0.42	16.7	-37.12	43.93
-181.54	136.91	0.46	-18.76	-38.17	57.05	-0.42	16.7	-37.12	50.11
-181.49	136.87	0.39	-16.38	-39.56	37.26	-0.44	17.13	-36.12	41.61
-181.49	136.86	0.39	-16.38	-39.56	57.04	-0.44	17.13	-36.12	52.86
-181.36	136.69	0.47	-19.71	-39.03	34.26	-0.42	16.7	-37.12	43.93
-181.48	136.77	0.46	-18.76	-38.17	57.05	-0.42	16.7	-37.12	50.11
-181.55	136.87	0.39	-16.38	-39.56	37.26	-0.44	17.13	-36.12	41.61
-181.55	136.97	0.39	-16.38	-39.56	57.04	-0.44	17.13	-36.12	52.86
-181.53	137.63	0.47	-19.71	-39.03	34.26	-0.42	16.7	-37.12	43.93
-181.61	137.98	0.46	-18.76	-38.17	57.05	-0.42	16.7	-37.12	50.11
-181.52	138.48	0.39	-16.38	-39.56	37.26	-0.44	17.13	-36.12	41.61
-181.5	139.43	0.39	-16.38	-39.56	57.04	-0.44	17.13	-36.12	52.86
-181.46	139.71	0.47	-19.71	-39.03	34.26	-0.42	16.7	-37.12	43.93
-181.62	140.34	0.46	-18.76	-38.17	57.05	-0.42	16.7	-37.12	50.11
-181.59	140.57	0.39	-16.38	-39.56	37.26	-0.44	17.13	-36.12	41.61
-181.46	140.57	0.39	-16.38	-39.56	57.04	-0.44	17.13	-36.12	52.86
-181.4	140.69	0.47	-19.71	-39.03	34.26	-0.42	16.7	-37.12	43.93

Turtle		SPA120				SPA60			
Y	X	Rot ang	Z	Y	X	Rot ang	Z	Y	X
-181.36	141.21	0.46	-18.76	-38.17	57.05	-0.42	16.7	-37.12	50.11
-181.45	141.55	0.39	-16.38	-39.56	37.26	-0.44	17.13	-36.12	41.61
-181.51	141.77	0.39	-16.38	-39.56	57.04	-0.44	17.13	-36.12	52.86
-181.37	142.08	0.47	-19.71	-39.03	34.26	-0.42	16.7	-37.12	43.93
-181.39	142.38	0.46	-18.76	-38.17	57.05	-0.42	16.7	-37.12	50.11
-181.51	142.6	0.39	-16.38	-39.56	37.26	-0.44	17.13	-36.12	41.61
-181.48	142.72	0.39	-16.38	-39.56	57.04	-0.44	17.13	-36.12	52.86
-181.3	142.7	0.47	-19.71	-39.03	34.26	-0.42	16.7	-37.12	43.93
-181.19	143.3	0.46	-18.76	-38.17	57.05	-0.42	16.7	-37.12	50.11
-181.1	143.7	0.39	-16.38	-39.56	37.26	-0.44	17.13	-36.12	41.61
-181.11	143.53	0.39	-16.38	-39.56	57.04	-0.44	17.13	-36.12	52.86
-180.99	144.2	0.47	-19.71	-39.03	34.26	-0.42	16.7	-37.12	43.93
-180.93	144.56	0.46	-18.76	-38.17	57.05	-0.42	16.7	-37.12	50.11
-180.88	145.2	0.39	-16.38	-39.56	37.26	-0.44	17.13	-36.12	41.61
-180.81	145.95	0.39	-16.38	-39.56	57.04	-0.44	17.13	-36.12	52.86
-180.76	146.41	0.47	-19.71	-39.03	34.26	-0.42	16.7	-37.12	43.93
-180.7	147	0.46	-18.76	-38.17	57.05	-0.42	16.7	-37.12	50.11
-180.71	147.55	0.39	-16.38	-39.56	37.26	-0.44	17.13	-36.12	41.61
-180.69	148.04	0.39	-16.38	-39.56	57.04	-0.44	17.13	-36.12	52.86
-180.72	148.71	0.47	-19.71	-39.03	34.26	-0.42	16.7	-37.12	43.93
-180.82	149.42	0.46	-18.76	-38.17	57.05	-0.42	16.7	-37.12	50.11
-180.8	149.62	0.39	-16.38	-39.56	37.26	-0.44	17.13	-36.12	41.61
-180.83	149.82	0.39	-16.38	-39.56	57.04	-0.44	17.13	-36.12	52.86
-180.81	149.82	0.47	-19.71	-39.03	34.26	-0.42	16.7	-37.12	43.93
-180.83	149.85	0.46	-18.76	-38.17	57.05	-0.42	16.7	-37.12	50.11
-180.87	149.87	0.39	-16.38	-39.56	37.26	-0.44	17.13	-36.12	41.61
-180.84	149.88	0.39	-16.38	-39.56	57.04	-0.44	17.13	-36.12	52.86
-180.82	149.89	0.47	-19.71	-39.03	34.26	-0.42	16.7	-37.12	43.93
-180.79	149.9	0.46	-18.76	-38.17	57.05	-0.42	16.7	-37.12	50.11
-180.8	149.88	0.39	-16.38	-39.56	37.26	-0.44	17.13	-36.12	41.61
-180.78	149.88	0.39	-16.38	-39.56	57.04	-0.44	17.13	-36.12	52.86
-180.76	149.85	0.47	-19.71	-39.03	34.26	-0.42	16.7	-37.12	43.93
-180.75	149.81	0.46	-18.76	-38.17	57.05	-0.42	16.7	-37.12	50.11
-180.76	149.82	0.39	-16.38	-39.56	37.26	-0.44	17.13	-36.12	41.61
-180.77	149.87	0.39	-16.38	-39.56	57.04	-0.44	17.13	-36.12	52.86
-180.75	150.08	0.47	-19.71	-39.03	34.26	-0.42	16.7	-37.12	43.93
-180.74	150.68	0.46	-18.76	-38.17	57.05	-0.42	16.7	-37.12	50.11
-180.73	150.86	0.39	-16.38	-39.56	37.26	-0.44	17.13	-36.12	41.61
-180.72	151.59	0.39	-16.38	-39.56	57.04	-0.44	17.13	-36.12	52.86
-180.79	151.85	0.47	-19.71	-39.03	34.26	-0.42	16.7	-37.12	43.93
-180.91	152.32	0.46	-18.76	-38.17	57.05	-0.42	16.7	-37.12	50.11
-181.04	152.77	0.39	-16.38	-39.56	37.26	-0.44	17.13	-36.12	41.61
-181.1	153.32	0.39	-16.38	-39.56	57.04	-0.44	17.13	-36.12	52.86
-181.23	153.58	0.47	-19.71	-39.03	34.26	-0.42	16.7	-37.12	43.93

Turtle		SPA120				SPA60			
Y	X	Rot ang	Z	Y	X	Rot ang	Z	Y	X
-181.24	153.94	0.46	-18.76	-38.17	57.05	-0.42	16.7	-37.12	50.11
-181.36	154.4	0.39	-16.38	-39.56	37.26	-0.44	17.13	-36.12	41.61
-181.38	154.54	0.39	-16.38	-39.56	57.04	-0.44	17.13	-36.12	52.86
-181.37	154.65	0.47	-19.71	-39.03	34.26	-0.42	16.7	-37.12	43.93
-181.46	154.91	0.46	-18.76	-38.17	57.05	-0.42	16.7	-37.12	50.11
-181.47	155.21	0.39	-16.38	-39.56	37.26	-0.44	17.13	-36.12	41.61
-181.52	155.35	0.39	-16.38	-39.56	57.04	-0.44	17.13	-36.12	52.86
-181.52	155.48	0.47	-19.71	-39.03	34.26	-0.42	16.7	-37.12	43.93
-181.52	155.52	0.46	-18.76	-38.17	57.05	-0.42	16.7	-37.12	50.11
-181.42	155.51	0.39	-16.38	-39.56	37.26	-0.44	17.13	-36.12	41.61
-181.31	156.23	0.39	-16.38	-39.56	57.04	-0.44	17.13	-36.12	52.86
-181.2	156.56	0.47	-19.71	-39.03	34.26	-0.42	16.7	-37.12	43.93
-181.19	157.24	0.46	-18.76	-38.17	57.05	-0.42	16.7	-37.12	50.11
-181.15	157.59	0.39	-16.38	-39.56	37.26	-0.44	17.13	-36.12	41.61
-181.12	158.2	0.39	-16.38	-39.56	57.04	-0.44	17.13	-36.12	52.86
-181.09	159.04	0.47	-19.71	-39.03	34.26	-0.42	16.7	-37.12	43.93
-180.97	159.33	0.46	-18.76	-38.17	57.05	-0.42	16.7	-37.12	50.11
-181	160.06	0.39	-16.38	-39.56	37.26	-0.44	17.13	-36.12	41.61
-180.98	160.74	0.39	-16.38	-39.56	57.04	-0.44	17.13	-36.12	52.86
-180.79	161.1	0.47	-19.71	-39.03	34.26	-0.42	16.7	-37.12	43.93
-180.78	161.95	0.46	-18.76	-38.17	57.05	-0.42	16.7	-37.12	50.11
-180.73	162.19	0.39	-16.38	-39.56	37.26	-0.44	17.13	-36.12	41.61
-180.83	162.08	0.39	-16.38	-39.56	57.04	-0.44	17.13	-36.12	52.86
-180.99	162.12	0.47	-19.71	-39.03	34.26	-0.42	16.7	-37.12	43.93
-180.98	162.04	0.46	-18.76	-38.17	57.05	-0.42	16.7	-37.12	50.11
-181.06	162.18	0.39	-16.38	-39.56	37.26	-0.44	17.13	-36.12	41.61
-181.04	162.07	0.39	-16.38	-39.56	57.04	-0.44	17.13	-36.12	52.86
-181.02	162.11	0.47	-19.71	-39.03	34.26	-0.42	16.7	-37.12	43.93
-180.96	162.1	0.46	-18.76	-38.17	57.05	-0.42	16.7	-37.12	50.11
-180.97	162.08	0.39	-16.38	-39.56	37.26	-0.44	17.13	-36.12	41.61
-180.97	162.02	0.39	-16.38	-39.56	57.04	-0.44	17.13	-36.12	52.86
-180.97	162.04	0.47	-19.71	-39.03	34.26	-0.42	16.7	-37.12	43.93
-180.99	161.97	0.46	-18.76	-38.17	57.05	-0.42	16.7	-37.12	50.11
-180.94	161.87	0.39	-16.38	-39.56	37.26	-0.44	17.13	-36.12	41.61
-181.06	161.94	0.39	-16.38	-39.56	57.04	-0.44	17.13	-36.12	52.86
-181.06	162.04	0.47	-19.71	-39.03	34.26	-0.42	16.7	-37.12	43.93
-181.06	162.9	0.46	-18.76	-38.17	57.05	-0.42	16.7	-37.12	50.11
-181.03	162.89	0.39	-16.38	-39.56	37.26	-0.44	17.13	-36.12	41.61
-181.09	163.42	0.39	-16.38	-39.56	57.04	-0.44	17.13	-36.12	52.86
-180.99	163.82	0.47	-19.71	-39.03	34.26	-0.42	16.7	-37.12	43.93
-181.01	164.41	0.46	-18.76	-38.17	57.05	-0.42	16.7	-37.12	50.11
-181.1	164.77	0.39	-16.38	-39.56	37.26	-0.44	17.13	-36.12	41.61
-180.97	165.02	0.39	-16.38	-39.56	57.04	-0.44	17.13	-36.12	52.86
-181.23	165.61	0.47	-19.71	-39.03	34.26	-0.42	16.7	-37.12	43.93

Turtle		SPA120				SPA60			
**Y**	**X**	**Rot ang**	**Z**	**Y**	**X**	**Rot ang**	**Z**	**Y**	**X**
-181.12	165.81	0.46	-18.76	-38.17	57.05	-0.42	16.7	-37.12	50.11
-181.1	166.32	0.39	-16.38	-39.56	37.26	-0.44	17.13	-36.12	41.61
-180.96	166.59	0.39	-16.38	-39.56	57.04	-0.44	17.13	-36.12	52.86
-181.03	166.79	0.47	-19.71	-39.03	34.26	-0.42	16.7	-37.12	43.93
-180.97	167.25	0.46	-18.76	-38.17	57.05	-0.42	16.7	-37.12	50.11
-180.92	167.44	0.39	-16.38	-39.56	37.26	-0.44	17.13	-36.12	41.61
-180.91	167.52	0.39	-16.38	-39.56	57.04	-0.44	17.13	-36.12	52.86
-180.82	167.59	0.47	-19.71	-39.03	34.26	-0.42	16.7	-37.12	43.93
-180.8	167.74	0.46	-18.76	-38.17	57.05	-0.42	16.7	-37.12	50.11
-180.8	167.72	0.39	-16.38	-39.56	37.26	-0.44	17.13	-36.12	41.61
-180.75	167.66	0.39	-16.38	-39.56	57.04	-0.44	17.13	-36.12	52.86
-180.59	168.35	0.47	-19.71	-39.03	34.26	-0.42	16.7	-37.12	43.93
-180.56	168.63	0.46	-18.76	-38.17	57.05	-0.42	16.7	-37.12	50.11
-180.51	169.33	0.39	-16.38	-39.56	37.26	-0.44	17.13	-36.12	41.61
-180.19	170.38	0.39	-16.38	-39.56	57.04	-0.44	17.13	-36.12	52.86
-180.22	171.15	0.47	-19.71	-39.03	34.26	-0.42	16.7	-37.12	43.93
-180.17	171.76	0.46	-18.76	-38.17	57.05	-0.42	16.7	-37.12	50.11
-180.27	171.92	0.39	-16.38	-39.56	37.26	-0.44	17.13	-36.12	41.61
-180.08	172.33	0.39	-16.38	-39.56	57.04	-0.44	17.13	-36.12	52.86
-180.18	172.98	0.47	-19.71	-39.03	34.26	-0.42	16.7	-37.12	43.93
-180.07	173.27	0.46	-18.76	-38.17	57.05	-0.42	16.7	-37.12	50.11
-180.21	173.85	0.39	-16.38	-39.56	37.26	-0.44	17.13	-36.12	41.61
-180.17	174.03	0.39	-16.38	-39.56	57.04	-0.44	17.13	-36.12	52.86
-180.25	174.57	0.47	-19.71	-39.03	34.26	-0.42	16.7	-37.12	43.93
-180.33	174.86	0.46	-18.76	-38.17	57.05	-0.42	16.7	-37.12	50.11
-180.32	174.88	0.39	-16.38	-39.56	37.26	-0.44	17.13	-36.12	41.61
-180.33	174.91	0.39	-16.38	-39.56	57.04	-0.44	17.13	-36.12	52.86
-180.35	174.92	0.47	-19.71	-39.03	34.26	-0.42	16.7	-37.12	43.93
-180.29	175.01	0.46	-18.76	-38.17	57.05	-0.42	16.7	-37.12	50.11
-180.29	174.98	0.39	-16.38	-39.56	37.26	-0.44	17.13	-36.12	41.61
-180.29	174.95	0.39	-16.38	-39.56	57.04	-0.44	17.13	-36.12	52.86
-180.29	174.94	0.47	-19.71	-39.03	34.26	-0.42	16.7	-37.12	43.93
-180.29	174.94	0.46	-18.76	-38.17	57.05	-0.42	16.7	-37.12	50.11
-180.26	174.93	0.39	-16.38	-39.56	37.26	-0.44	17.13	-36.12	41.61
-180.3	174.86	0.39	-16.38	-39.56	57.04	-0.44	17.13	-36.12	52.86
-180.31	174.93	0.47	-19.71	-39.03	34.26	-0.42	16.7	-37.12	43.93
-180.38	174.99	0.46	-18.76	-38.17	57.05	-0.42	16.7	-37.12	50.11
-180.54	175.59	0.39	-16.38	-39.56	37.26	-0.44	17.13	-36.12	41.61
-180.53	175.97	0.39	-16.38	-39.56	57.04	-0.44	17.13	-36.12	52.86
-180.91	176.7	0.47	-19.71	-39.03	34.26	-0.42	16.7	-37.12	43.93
-181.1	177.66	0.46	-18.76	-38.17	57.05	-0.42	16.7	-37.12	50.11
-181.08	177.69	0.39	-16.38	-39.56	37.26	-0.44	17.13	-36.12	41.61
-181.06	177.95	0.39	-16.38	-39.56	57.04	-0.44	17.13	-36.12	52.86
-181.05	178.58	0.47	-19.71	-39.03	34.26	-0.42	16.7	-37.12	43.93

Turtle		SPA120				SPA60			
Y	X	Rot ang	Z	Y	X	Rot ang	Z	Y	X
-181.07	178.76	0.46	-18.76	-38.17	57.05	-0.42	16.7	-37.12	50.11
-181.1	179.37	0.39	-16.38	-39.56	37.26	-0.44	17.13	-36.12	41.61
-181.03	179.6	0.39	-16.38	-39.56	57.04	-0.44	17.13	-36.12	52.86
-181.01	179.69	0.47	-19.71	-39.03	34.26	-0.42	16.7	-37.12	43.93
-181.03	180.45	0.46	-18.76	-38.17	57.05	-0.42	16.7	-37.12	50.11
-181.03	180.57	0.39	-16.38	-39.56	37.26	-0.44	17.13	-36.12	41.61
-180.93	180.67	0.39	-16.38	-39.56	57.04	-0.44	17.13	-36.12	52.86
-180.92	180.61	0.47	-19.71	-39.03	34.26	-0.42	16.7	-37.12	43.93
-180.99	180.77	0.46	-18.76	-38.17	57.05	-0.42	16.7	-37.12	50.11
-180.9	180.79	0.39	-16.38	-39.56	37.26	-0.44	17.13	-36.12	41.61
-180.9	181.14	0.39	-16.38	-39.56	57.04	-0.44	17.13	-36.12	52.86
-180.88	181.3	0.47	-19.71	-39.03	34.26	-0.42	16.7	-37.12	43.93
-180.75	181.59	0.46	-18.76	-38.17	57.05	-0.42	16.7	-37.12	50.11
-180.66	182.27	0.39	-16.38	-39.56	37.26	-0.44	17.13	-36.12	41.61
-180.59	182.92	0.39	-16.38	-39.56	57.04	-0.44	17.13	-36.12	52.86
-180.5	184.16	0.47	-19.71	-39.03	34.26	-0.42	16.7	-37.12	43.93
-180.29	184.79	0.46	-18.76	-38.17	57.05	-0.42	16.7	-37.12	50.11
-179.96	185.2	0.39	-16.38	-39.56	37.26	-0.44	17.13	-36.12	41.61
-179.93	185.9	0.39	-16.38	-39.56	57.04	-0.44	17.13	-36.12	52.86
-179.51	186.33	0.47	-19.71	-39.03	34.26	-0.42	16.7	-37.12	43.93
-179.56	186.19	0.46	-18.76	-38.17	57.05	-0.42	16.7	-37.12	50.11
-179.67	186.8	0.39	-16.38	-39.56	37.26	-0.44	17.13	-36.12	41.61
-179.46	187.1	0.39	-16.38	-39.56	57.04	-0.44	17.13	-36.12	52.86
-179.76	187.67	0.47	-19.71	-39.03	34.26	-0.42	16.7	-37.12	43.93
-179.56	187.88	0.46	-18.76	-38.17	57.05	-0.42	16.7	-37.12	50.11
-179.63	187.96	0.39	-16.38	-39.56	37.26	-0.44	17.13	-36.12	41.61
-179.61	187.95	0.39	-16.38	-39.56	57.04	-0.44	17.13	-36.12	52.86
-179.56	187.94	0.47	-19.71	-39.03	34.26	-0.42	16.7	-37.12	43.93
-179.35	187.99	0.46	-18.76	-38.17	57.05	-0.42	16.7	-37.12	50.11
-179.42	187.95	0.39	-16.38	-39.56	37.26	-0.44	17.13	-36.12	41.61
-179.47	187.95	0.39	-16.38	-39.56	57.04	-0.44	17.13	-36.12	52.86
-179.38	188.02	0.47	-19.71	-39.03	34.26	-0.42	16.7	-37.12	43.93
-179.36	187.98	0.46	-18.76	-38.17	57.05	-0.42	16.7	-37.12	50.11
-179.46	187.94	0.39	-16.38	-39.56	37.26	-0.44	17.13	-36.12	41.61
-179.6	187.9	0.39	-16.38	-39.56	57.04	-0.44	17.13	-36.12	52.86
-179.59	187.87	0.47	-19.71	-39.03	34.26	-0.42	16.7	-37.12	43.93
-179.6	187.93	0.46	-18.76	-38.17	57.05	-0.42	16.7	-37.12	50.11
-179.4	188.03	0.39	-16.38	-39.56	37.26	-0.44	17.13	-36.12	41.61
-179.73	188.5	0.39	-16.38	-39.56	57.04	-0.44	17.13	-36.12	52.86
-179.62	189.01	0.47	-19.71	-39.03	34.26	-0.42	16.7	-37.12	43.93
-179.8	189.66	0.46	-18.76	-38.17	57.05	-0.42	16.7	-37.12	50.11
-179.67	189.86	0.39	-16.38	-39.56	37.26	-0.44	17.13	-36.12	41.61
-179.68	190.41	0.39	-16.38	-39.56	57.04	-0.44	17.13	-36.12	52.86
-179.76	190.8	0.47	-19.71	-39.03	34.26	-0.42	16.7	-37.12	43.93

Turtle		SPA120				SPA60			
Y	X	Rot ang	Z	Y	X	Rot ang	Z	Y	X
-179.93	191.46	0.46	-18.76	-38.17	57.05	-0.42	16.7	-37.12	50.11
-180.06	191.76	0.39	-16.38	-39.56	37.26	-0.44	17.13	-36.12	41.61
-180.28	192.35	0.39	-16.38	-39.56	57.04	-0.44	17.13	-36.12	52.86
-180.31	192.61	0.47	-19.71	-39.03	34.26	-0.42	16.7	-37.12	43.93
-180.22	192.8	0.46	-18.76	-38.17	57.05	-0.42	16.7	-37.12	50.11
-180.22	193.24	0.39	-16.38	-39.56	37.26	-0.44	17.13	-36.12	41.61
-180.27	193.47	0.39	-16.38	-39.56	57.04	-0.44	17.13	-36.12	52.86
-180.47	193.57	0.47	-19.71	-39.03	34.26	-0.42	16.7	-37.12	43.93
-180.38	193.64	0.46	-18.76	-38.17	57.05	-0.42	16.7	-37.12	50.11
-180.35	193.87	0.39	-16.38	-39.56	37.26	-0.44	17.13	-36.12	41.61
-180.25	194.22	0.39	-16.38	-39.56	57.04	-0.44	17.13	-36.12	52.86
-180.35	194.4	0.47	-19.71	-39.03	34.26	-0.42	16.7	-37.12	43.93
-180.14	194.47	0.46	-18.76	-38.17	57.05	-0.42	16.7	-37.12	50.11
-179.93	195.13	0.39	-16.38	-39.56	37.26	-0.44	17.13	-36.12	41.61
-180	195.37	0.39	-16.38	-39.56	57.04	-0.44	17.13	-36.12	52.86
-179.86	196.07	0.47	-19.71	-39.03	34.26	-0.42	16.7	-37.12	43.93
-179.65	196.45	0.46	-18.76	-38.17	57.05	-0.42	16.7	-37.12	50.11
-179.55	197.14	0.39	-16.38	-39.56	37.26	-0.44	17.13	-36.12	41.61
-179.68	197.77	0.39	-16.38	-39.56	57.04	-0.44	17.13	-36.12	52.86
-179.25	198.17	0.47	-19.71	-39.03	34.26	-0.42	16.7	-37.12	43.93
-179.26	198.81	0.46	-18.76	-38.17	57.05	-0.42	16.7	-37.12	50.11
-178.87	199.13	0.39	-16.38	-39.56	37.26	-0.44	17.13	-36.12	41.61
-179.09	199.74	0.39	-16.38	-39.56	57.04	-0.44	17.13	-36.12	52.86
-178.61	200.06	0.47	-19.71	-39.03	34.26	-0.42	16.7	-37.12	43.93
-178.36	200.05	0.46	-18.76	-38.17	57.05	-0.42	16.7	-37.12	50.11
-178.64	200.87	0.39	-16.38	-39.56	37.26	-0.44	17.13	-36.12	41.61
-178.7	200.86	0.39	-16.38	-39.56	57.04	-0.44	17.13	-36.12	52.86
-178.72	200.87	0.47	-19.71	-39.03	34.26	-0.42	16.7	-37.12	43.93
-178.68	200.89	0.46	-18.76	-38.17	57.05	-0.42	16.7	-37.12	50.11
-178.61	200.99	0.39	-16.38	-39.56	37.26	-0.44	17.13	-36.12	41.61
-178.69	200.94	0.39	-16.38	-39.56	57.04	-0.44	17.13	-36.12	52.86
-178.62	200.99	0.47	-19.71	-39.03	34.26	-0.42	16.7	-37.12	43.93
-178.71	200.93	0.46	-18.76	-38.17	57.05	-0.42	16.7	-37.12	50.11
-178.73	200.91	0.39	-16.38	-39.56	37.26	-0.44	17.13	-36.12	41.61
-178.77	200.89	0.39	-16.38	-39.56	57.04	-0.44	17.13	-36.12	52.86
-178.83	200.83	0.47	-19.71	-39.03	34.26	-0.42	16.7	-37.12	43.93
-178.67	200.91	0.46	-18.76	-38.17	57.05	-0.42	16.7	-37.12	50.11
-178.49	201.01	0.39	-16.38	-39.56	37.26	-0.44	17.13	-36.12	41.61
-178.53	200.95	0.39	-16.38	-39.56	57.04	-0.44	17.13	-36.12	52.86
-179.41	201.71	0.47	-19.71	-39.03	34.26	-0.42	16.7	-37.12	43.93
-179.28	202.03	0.46	-18.76	-38.17	57.05	-0.42	16.7	-37.12	50.11
-179.89	202.64	0.39	-16.38	-39.56	37.26	-0.44	17.13	-36.12	41.61
-179.96	203.61	0.39	-16.38	-39.56	57.04	-0.44	17.13	-36.12	52.86
-179.56	203.86	0.47	-19.71	-39.03	34.26	-0.42	16.7	-37.12	43.93

Turtle		SPA120				SPA60			
Y	X	Rot ang	Z	Y	X	Rot ang	Z	Y	X
-179.75	204.47	0.46	-18.76	-38.17	57.05	-0.42	16.7	-37.12	50.11
-179.65	204.7	0.39	-16.38	-39.56	37.26	-0.44	17.13	-36.12	41.61
-179.7	205.22	0.39	-16.38	-39.56	57.04	-0.44	17.13	-36.12	52.86
-179.64	205.53	0.47	-19.71	-39.03	34.26	-0.42	16.7	-37.12	43.93
-179.79	205.65	0.46	-18.76	-38.17	57.05	-0.42	16.7	-37.12	50.11
-179.68	205.87	0.39	-16.38	-39.56	37.26	-0.44	17.13	-36.12	41.61
-179.63	205.72	0.39	-16.38	-39.56	57.04	-0.44	17.13	-36.12	52.86
-179.69	206.28	0.47	-19.71	-39.03	34.26	-0.42	16.7	-37.12	43.93
-179.56	206.46	0.46	-18.76	-38.17	57.05	-0.42	16.7	-37.12	50.11
-179.54	206.65	0.39	-16.38	-39.56	37.26	-0.44	17.13	-36.12	41.61
-179.65	206.68	0.39	-16.38	-39.56	57.04	-0.44	17.13	-36.12	52.86
-179.6	207.16	0.47	-19.71	-39.03	34.26	-0.42	16.7	-37.12	43.93
-179.36	207.34	0.46	-18.76	-38.17	57.05	-0.42	16.7	-37.12	50.11
-179.16	207.52	0.39	-16.38	-39.56	37.26	-0.44	17.13	-36.12	41.61
-179.08	208.18	0.39	-16.38	-39.56	57.04	-0.44	17.13	-36.12	52.86
-178.79	208.41	0.47	-19.71	-39.03	34.26	-0.42	16.7	-37.12	43.93
-178.81	209.13	0.46	-18.76	-38.17	57.05	-0.42	16.7	-37.12	50.11
-177.86	209.47	0.39	-16.38	-39.56	37.26	-0.44	17.13	-36.12	41.61
-177.74	209.33	0.39	-16.38	-39.56	57.04	-0.44	17.13	-36.12	52.86
-177.75	210.01	0.47	-19.71	-39.03	34.26	-0.42	16.7	-37.12	43.93
-178.16	210.72	0.46	-18.76	-38.17	57.05	-0.42	16.7	-37.12	50.11
-177.95	211.78	0.39	-16.38	-39.56	37.26	-0.44	17.13	-36.12	41.61
-177.37	211.95	0.39	-16.38	-39.56	57.04	-0.44	17.13	-36.12	52.86
-176.65	211.97	0.47	-19.71	-39.03	34.26	-0.42	16.7	-37.12	43.93
-176.57	211.94	0.46	-18.76	-38.17	57.05	-0.42	16.7	-37.12	50.11
-177.42	212.66	0.39	-16.38	-39.56	37.26	-0.44	17.13	-36.12	41.61
-176.92	212.95	0.39	-16.38	-39.56	57.04	-0.44	17.13	-36.12	52.86
-178.22	213.6	0.47	-19.71	-39.03	34.26	-0.42	16.7	-37.12	43.93
-178.21	213.67	0.46	-18.76	-38.17	57.05	-0.42	16.7	-37.12	50.11
-178.05	213.79	0.39	-16.38	-39.56	37.26	-0.44	17.13	-36.12	41.61
-178.08	213.81	0.39	-16.38	-39.56	57.04	-0.44	17.13	-36.12	52.86
-178.09	213.82	0.47	-19.71	-39.03	34.26	-0.42	16.7	-37.12	43.93
-178.07	213.83	0.46	-18.76	-38.17	57.05	-0.42	16.7	-37.12	50.11
-177.96	213.83	0.39	-16.38	-39.56	37.26	-0.44	17.13	-36.12	41.61
-177.87	213.84	0.39	-16.38	-39.56	57.04	-0.44	17.13	-36.12	52.86
-177.85	213.86	0.47	-19.71	-39.03	34.26	-0.42	16.7	-37.12	43.93
-177.89	213.86	0.46	-18.76	-38.17	57.05	-0.42	16.7	-37.12	50.11
-178.07	213.82	0.39	-16.38	-39.56	37.26	-0.44	17.13	-36.12	41.61
-177.9	213.89	0.39	-16.38	-39.56	57.04	-0.44	17.13	-36.12	52.86
-178.03	213.91	0.47	-19.71	-39.03	34.26	-0.42	16.7	-37.12	43.93
-177.49	213.96	0.46	-18.76	-38.17	57.05	-0.42	16.7	-37.12	50.11
-179.27	215.49	0.39	-16.38	-39.56	37.26	-0.44	17.13	-36.12	41.61
-179.12	215.62	0.39	-16.38	-39.56	57.04	-0.44	17.13	-36.12	52.86
-178.52	215.86	0.47	-19.71	-39.03	34.26	-0.42	16.7	-37.12	43.93

Turtle		SPA120				SPA60			
**Y**	**X**	**Rot ang**	**Z**	**Y**	**X**	**Rot ang**	**Z**	**Y**	**X**
-179.05	216.56	0.46	-18.76	-38.17	57.05	-0.42	16.7	-37.12	50.11
-178.7	216.9	0.39	-16.38	-39.56	37.26	-0.44	17.13	-36.12	41.61
-179	217.49	0.39	-16.38	-39.56	57.04	-0.44	17.13	-36.12	52.86
-178.87	217.68	0.47	-19.71	-39.03	34.26	-0.42	16.7	-37.12	43.93
-179.18	218.23	0.46	-18.76	-38.17	57.05	-0.42	16.7	-37.12	50.11
-178.88	218.51	0.39	-16.38	-39.56	37.26	-0.44	17.13	-36.12	41.61
-178.76	218.66	0.39	-16.38	-39.56	57.04	-0.44	17.13	-36.12	52.86
-179.48	219.26	0.47	-19.71	-39.03	34.26	-0.42	16.7	-37.12	43.93
-179.14	219.52	0.46	-18.76	-38.17	57.05	-0.42	16.7	-37.12	50.11
-178.95	219.71	0.39	-16.38	-39.56	37.26	-0.44	17.13	-36.12	41.61
-178.9	219.6	0.39	-16.38	-39.56	57.04	-0.44	17.13	-36.12	52.86
-178.73	219.67	0.47	-19.71	-39.03	34.26	-0.42	16.7	-37.12	43.93
-178.82	219.74	0.46	-18.76	-38.17	57.05	-0.42	16.7	-37.12	50.11
-179.14	220	0.39	-16.38	-39.56	37.26	-0.44	17.13	-36.12	41.61
-178.83	220.29	0.39	-16.38	-39.56	57.04	-0.44	17.13	-36.12	52.86
-178.64	220.55	0.47	-19.71	-39.03	34.26	-0.42	16.7	-37.12	43.93
-178.64	221.17	0.46	-18.76	-38.17	57.05	-0.42	16.7	-37.12	50.11
-177.97	221.37	0.39	-16.38	-39.56	37.26	-0.44	17.13	-36.12	41.61
-177.26	222.33	0.39	-16.38	-39.56	57.04	-0.44	17.13	-36.12	52.86
-177.69	223.04	0.47	-19.71	-39.03	34.26	-0.42	16.7	-37.12	43.93
-178.24	223.84	0.46	-18.76	-38.17	57.05	-0.42	16.7	-37.12	50.11
-177.37	224.03	0.39	-16.38	-39.56	37.26	-0.44	17.13	-36.12	41.61
-177.42	224.03	0.39	-16.38	-39.56	57.04	-0.44	17.13	-36.12	52.86
-176.45	224.15	0.47	-19.71	-39.03	34.26	-0.42	16.7	-37.12	43.93
-177.5	224.9	0.46	-18.76	-38.17	57.05	-0.42	16.7	-37.12	50.11
-178.23	225.73	0.39	-16.38	-39.56	37.26	-0.44	17.13	-36.12	41.61
-177.64	225.83	0.39	-16.38	-39.56	57.04	-0.44	17.13	-36.12	52.86
-178.13	226.76	0.47	-19.71	-39.03	34.26	-0.42	16.7	-37.12	43.93
-178.19	226.81	0.46	-18.76	-38.17	57.05	-0.42	16.7	-37.12	50.11
-178.22	226.83	0.39	-16.38	-39.56	37.26	-0.44	17.13	-36.12	41.61
-178.24	226.86	0.39	-16.38	-39.56	57.04	-0.44	17.13	-36.12	52.86
-178.19	226.94	0.47	-19.71	-39.03	34.26	-0.42	16.7	-37.12	43.93
-178.21	226.91	0.46	-18.76	-38.17	57.05	-0.42	16.7	-37.12	50.11
-178.17	226.9	0.39	-16.38	-39.56	37.26	-0.44	17.13	-36.12	41.61
-178.2	226.89	0.39	-16.38	-39.56	57.04	-0.44	17.13	-36.12	52.86
-178.25	226.89	0.47	-19.71	-39.03	34.26	-0.42	16.7	-37.12	43.93
-177.98	226.9	0.46	-18.76	-38.17	57.05	-0.42	16.7	-37.12	50.11
-178.27	226.87	0.39	-16.38	-39.56	37.26	-0.44	17.13	-36.12	41.61
-178.31	226.85	0.39	-16.38	-39.56	57.04	-0.44	17.13	-36.12	52.86
-177.99	226.91	0.47	-19.71	-39.03	34.26	-0.42	16.7	-37.12	43.93
-177.22	226.98	0.46	-18.76	-38.17	57.05	-0.42	16.7	-37.12	50.11
-178.29	227.72	0.39	-16.38	-39.56	37.26	-0.44	17.13	-36.12	41.61
-179.2	228.58	0.39	-16.38	-39.56	57.04	-0.44	17.13	-36.12	52.86
-178.28	228.77	0.47	-19.71	-39.03	34.26	-0.42	16.7	-37.12	43.93

Turtle		SPA120				SPA60			
Y	X	Rot ang	Z	Y	X	Rot ang	Z	Y	X
-179.16	229.54	0.46	-18.76	-38.17	57.05	-0.42	16.7	-37.12	50.11
-178.44	229.72	0.39	-16.38	-39.56	37.26	-0.44	17.13	-36.12	41.61
-178.34	229.69	0.39	-16.38	-39.56	57.04	-0.44	17.13	-36.12	52.86
-178.99	230.51	0.47	-19.71	-39.03	34.26	-0.42	16.7	-37.12	43.93
-178.64	230.75	0.46	-18.76	-38.17	57.05	-0.42	16.7	-37.12	50.11
-178.31	230.83	0.39	-16.38	-39.56	37.26	-0.44	17.13	-36.12	41.61
-178.7	231.58	0.39	-16.38	-39.56	57.04	-0.44	17.13	-36.12	52.86
-178.55	231.79	0.47	-19.71	-39.03	34.26	-0.42	16.7	-37.12	43.93
-179.36	232.31	0.46	-18.76	-38.17	57.05	-0.42	16.7	-37.12	50.11
-179.01	232.46	0.39	-16.38	-39.56	37.26	-0.44	17.13	-36.12	41.61
-178.85	232.51	0.39	-16.38	-39.56	57.04	-0.44	17.13	-36.12	52.86
-178.82	232.64	0.47	-19.71	-39.03	34.26	-0.42	16.7	-37.12	43.93
-178.74	232.71	0.46	-18.76	-38.17	57.05	-0.42	16.7	-37.12	50.11
-178.78	232.64	0.39	-16.38	-39.56	37.26	-0.44	17.13	-36.12	41.61
-178.83	232.58	0.39	-16.38	-39.56	57.04	-0.44	17.13	-36.12	52.86
-178.79	232.8	0.47	-19.71	-39.03	34.26	-0.42	16.7	-37.12	43.93
-178.74	233.33	0.46	-18.76	-38.17	57.05	-0.42	16.7	-37.12	50.11
-178.6	234.3	0.39	-16.38	-39.56	37.26	-0.44	17.13	-36.12	41.61
-177.25	234.31	0.39	-16.38	-39.56	57.04	-0.44	17.13	-36.12	52.86
-177.98	235.14	0.47	-19.71	-39.03	34.26	-0.42	16.7	-37.12	43.93
-178.61	236.04	0.46	-18.76	-38.17	57.05	-0.42	16.7	-37.12	50.11
-177.68	236.16	0.39	-16.38	-39.56	37.26	-0.44	17.13	-36.12	41.61
-178.15	236.88	0.39	-16.38	-39.56	57.04	-0.44	17.13	-36.12	52.86
-177.23	236.95	0.47	-19.71	-39.03	34.26	-0.42	16.7	-37.12	43.93
-177.9	237.88	0.46	-18.76	-38.17	57.05	-0.42	16.7	-37.12	50.11
-178.86	238.73	0.39	-16.38	-39.56	37.26	-0.44	17.13	-36.12	41.61
-178.76	238.82	0.39	-16.38	-39.56	57.04	-0.44	17.13	-36.12	52.86
-177.82	238.91	0.47	-19.71	-39.03	34.26	-0.42	16.7	-37.12	43.93
-177.33	239.07	0.46	-18.76	-38.17	57.05	-0.42	16.7	-37.12	50.11
-178.72	239.74	0.39	-16.38	-39.56	37.26	-0.44	17.13	-36.12	41.61
-178.71	239.8	0.39	-16.38	-39.56	57.04	-0.44	17.13	-36.12	52.86
-178.51	239.89	0.47	-19.71	-39.03	34.26	-0.42	16.7	-37.12	43.93
-178.46	239.91	0.46	-18.76	-38.17	57.05	-0.42	16.7	-37.12	50.11
-178.4	239.91	0.39	-16.38	-39.56	37.26	-0.44	17.13	-36.12	41.61
-178.37	239.91	0.39	-16.38	-39.56	57.04	-0.44	17.13	-36.12	52.86
-178.29	239.92	0.47	-19.71	-39.03	34.26	-0.42	16.7	-37.12	43.93
-178.19	239.92	0.46	-18.76	-38.17	57.05	-0.42	16.7	-37.12	50.11
-178.41	239.91	0.39	-16.38	-39.56	37.26	-0.44	17.13	-36.12	41.61
-178.45	239.9	0.39	-16.38	-39.56	57.04	-0.44	17.13	-36.12	52.86
-178.73	239.87	0.47	-19.71	-39.03	34.26	-0.42	16.7	-37.12	43.93
-178.69	239.9	0.46	-18.76	-38.17	57.05	-0.42	16.7	-37.12	50.11
-177.76	240	0.39	-16.38	-39.56	37.26	-0.44	17.13	-36.12	41.61
-178.75	240.74	0.39	-16.38	-39.56	57.04	-0.44	17.13	-36.12	52.86
-177.83	240.87	0.47	-19.71	-39.03	34.26	-0.42	16.7	-37.12	43.93

Turtle		SPA120				SPA60			
Y	X	Rot ang	Z	Y	X	Rot ang	Z	Y	X
-178.44	241.75	0.46	-18.76	-38.17	57.05	-0.42	16.7	-37.12	50.11
-177.81	241.82	0.39	-16.38	-39.56	37.26	-0.44	17.13	-36.12	41.61
-178.7	242.54	0.39	-16.38	-39.56	57.04	-0.44	17.13	-36.12	52.86
-177.97	242.75	0.47	-19.71	-39.03	34.26	-0.42	16.7	-37.12	43.93
-179.08	243.56	0.46	-18.76	-38.17	57.05	-0.42	16.7	-37.12	50.11
-178.68	243.62	0.39	-16.38	-39.56	37.26	-0.44	17.13	-36.12	41.61
-178.54	243.61	0.39	-16.38	-39.56	57.04	-0.44	17.13	-36.12	52.86
-178.05	243.81	0.47	-19.71	-39.03	34.26	-0.42	16.7	-37.12	43.93
-179	244.4	0.46	-18.76	-38.17	57.05	-0.42	16.7	-37.12	50.11
-178.14	244.65	0.39	-16.38	-39.56	37.26	-0.44	17.13	-36.12	41.61
-178.04	244.67	0.39	-16.38	-39.56	57.04	-0.44	17.13	-36.12	52.86
-179.01	245.4	0.47	-19.71	-39.03	34.26	-0.42	16.7	-37.12	43.93
-178.77	245.49	0.46	-18.76	-38.17	57.05	-0.42	16.7	-37.12	50.11
-178.76	245.52	0.39	-16.38	-39.56	37.26	-0.44	17.13	-36.12	41.61
-178.38	245.52	0.39	-16.38	-39.56	57.04	-0.44	17.13	-36.12	52.86
-178.19	245.52	0.47	-19.71	-39.03	34.26	-0.42	16.7	-37.12	43.93
-179.14	246.29	0.46	-18.76	-38.17	57.05	-0.42	16.7	-37.12	50.11
-178.03	246.39	0.39	-16.38	-39.56	37.26	-0.44	17.13	-36.12	41.61
-178.07	246.38	0.39	-16.38	-39.56	57.04	-0.44	17.13	-36.12	52.86
-178.88	247.24	0.47	-19.71	-39.03	34.26	-0.42	16.7	-37.12	43.93
-178.01	247.32	0.46	-18.76	-38.17	57.05	-0.42	16.7	-37.12	50.11
-178.63	248.24	0.39	-16.38	-39.56	37.26	-0.44	17.13	-36.12	41.61
-178.1	249.16	0.39	-16.38	-39.56	57.04	-0.44	17.13	-36.12	52.86
-178.49	250.13	0.47	-19.71	-39.03	34.26	-0.42	16.7	-37.12	43.93
-179.06	250.94	0.46	-18.76	-38.17	57.05	-0.42	16.7	-37.12	50.11
-178.98	250.99	0.39	-16.38	-39.56	37.26	-0.44	17.13	-36.12	41.61
-178.19	251.05	0.39	-16.38	-39.56	57.04	-0.44	17.13	-36.12	52.86
-178.84	251.86	0.47	-19.71	-39.03	34.26	-0.42	16.7	-37.12	43.93
-179.02	252.69	0.46	-18.76	-38.17	57.05	-0.42	16.7	-37.12	50.11
-178.95	252.86	0.39	-16.38	-39.56	37.26	-0.44	17.13	-36.12	41.61
-178.88	252.89	0.39	-16.38	-39.56	57.04	-0.44	17.13	-36.12	52.86
-178.68	252.92	0.47	-19.71	-39.03	34.26	-0.42	16.7	-37.12	43.93
-178.65	252.93	0.46	-18.76	-38.17	57.05	-0.42	16.7	-37.12	50.11
-178.64	252.96	0.39	-16.38	-39.56	37.26	-0.44	17.13	-36.12	41.61
-178.68	252.95	0.39	-16.38	-39.56	57.04	-0.44	17.13	-36.12	52.86
-178.43	252.99	0.47	-19.71	-39.03	34.26	-0.42	16.7	-37.12	43.93
-178.53	252.97	0.46	-18.76	-38.17	57.05	-0.42	16.7	-37.12	50.11
-178.6	252.95	0.39	-16.38	-39.56	37.26	-0.44	17.13	-36.12	41.61
-178.71	252.93	0.39	-16.38	-39.56	57.04	-0.44	17.13	-36.12	52.86
-178.79	252.9	0.47	-19.71	-39.03	34.26	-0.42	16.7	-37.12	43.93
-178.86	252.92	0.46	-18.76	-38.17	57.05	-0.42	16.7	-37.12	50.11
-178.82	252.92	0.39	-16.38	-39.56	37.26	-0.44	17.13	-36.12	41.61
-178.48	252.98	0.39	-16.38	-39.56	57.04	-0.44	17.13	-36.12	52.86
-177.73	253	0.47	-19.71	-39.03	34.26	-0.42	16.7	-37.12	43.93

Turtle		SPA120				SPA60			
Y	X	Rot ang	Z	Y	X	Rot ang	Z	Y	X
-178.85	253.76	0.46	-18.76	-38.17	57.05	-0.42	16.7	-37.12	50.11
-178.01	253.84	0.39	-16.38	-39.56	37.26	-0.44	17.13	-36.12	41.61
-178.03	254.77	0.39	-16.38	-39.56	57.04	-0.44	17.13	-36.12	52.86
-178.76	255.54	0.47	-19.71	-39.03	34.26	-0.42	16.7	-37.12	43.93
-178.1	255.66	0.46	-18.76	-38.17	57.05	-0.42	16.7	-37.12	50.11
-179.06	256.57	0.39	-16.38	-39.56	37.26	-0.44	17.13	-36.12	41.61
-178.35	256.67	0.39	-16.38	-39.56	57.04	-0.44	17.13	-36.12	52.86
-177.7	256.66	0.47	-19.71	-39.03	34.26	-0.42	16.7	-37.12	43.93
-177.13	256.68	0.46	-18.76	-38.17	57.05	-0.42	16.7	-37.12	50.11
-178.51	257.45	0.39	-16.38	-39.56	37.26	-0.44	17.13	-36.12	41.61
-178.49	257.49	0.39	-16.38	-39.56	57.04	-0.44	17.13	-36.12	52.86
-178.05	257.58	0.47	-19.71	-39.03	34.26	-0.42	16.7	-37.12	43.93
-177.63	257.57	0.46	-18.76	-38.17	57.05	-0.42	16.7	-37.12	50.11
-177.31	257.64	0.39	-16.38	-39.56	37.26	-0.44	17.13	-36.12	41.61
-178.38	258.43	0.39	-16.38	-39.56	57.04	-0.44	17.13	-36.12	52.86
-178.42	258.46	0.47	-19.71	-39.03	34.26	-0.42	16.7	-37.12	43.93
-178.35	258.48	0.46	-18.76	-38.17	57.05	-0.42	16.7	-37.12	50.11
-177.82	258.47	0.39	-16.38	-39.56	37.26	-0.44	17.13	-36.12	41.61
-178.36	259.36	0.39	-16.38	-39.56	57.04	-0.44	17.13	-36.12	52.86
-177.13	259.49	0.47	-19.71	-39.03	34.26	-0.42	16.7	-37.12	43.93
-178.3	260.28	0.46	-18.76	-38.17	57.05	-0.42	16.7	-37.12	50.11
-178.31	260.3	0.39	-16.38	-39.56	37.26	-0.44	17.13	-36.12	41.61
-178.62	261.02	0.39	-16.38	-39.56	57.04	-0.44	17.13	-36.12	52.86
-177.7	261.21	0.47	-19.71	-39.03	34.26	-0.42	16.7	-37.12	43.93
-178.17	262.13	0.46	-18.76	-38.17	57.05	-0.42	16.7	-37.12	50.11
-177.48	262.22	0.39	-16.38	-39.56	37.26	-0.44	17.13	-36.12	41.61
-177.78	263.14	0.39	-16.38	-39.56	57.04	-0.44	17.13	-36.12	52.86
-177.84	264.03	0.47	-19.71	-39.03	34.26	-0.42	16.7	-37.12	43.93
-177.43	264.15	0.46	-18.76	-38.17	57.05	-0.42	16.7	-37.12	50.11
-178.22	264.84	0.39	-16.38	-39.56	37.26	-0.44	17.13	-36.12	41.61
-178.07	264.97	0.39	-16.38	-39.56	57.04	-0.44	17.13	-36.12	52.86
-177.99	264.97	0.47	-19.71	-39.03	34.26	-0.42	16.7	-37.12	43.93
-177.98	264.97	0.46	-18.76	-38.17	57.05	-0.42	16.7	-37.12	50.11
-178	264.97	0.39	-16.38	-39.56	37.26	-0.44	17.13	-36.12	41.61
-177.83	265.03	0.39	-16.38	-39.56	57.04	-0.44	17.13	-36.12	52.86
-177.92	265.02	0.47	-19.71	-39.03	34.26	-0.42	16.7	-37.12	43.93
-177.99	265.01	0.46	-18.76	-38.17	57.05	-0.42	16.7	-37.12	50.11
-177.98	265.02	0.39	-16.38	-39.56	37.26	-0.44	17.13	-36.12	41.61
-177.84	265.05	0.39	-16.38	-39.56	57.04	-0.44	17.13	-36.12	52.86
-178.03	264.99	0.47	-19.71	-39.03	34.26	-0.42	16.7	-37.12	43.93
-178.11	264.95	0.46	-18.76	-38.17	57.05	-0.42	16.7	-37.12	50.11
-178.13	264.97	0.39	-16.38	-39.56	37.26	-0.44	17.13	-36.12	41.61
-178.08	265.05	0.39	-16.38	-39.56	57.04	-0.44	17.13	-36.12	52.86
-177.47	265.08	0.47	-19.71	-39.03	34.26	-0.42	16.7	-37.12	43.93

Turtle		SPA120				SPA60			
Y	X	Rot ang	Z	Y	X	Rot ang	Z	Y	X
-178.12	265.86	0.46	-18.76	-38.17	57.05	-0.42	16.7	-37.12	50.11
-177.33	265.93	0.39	-16.38	-39.56	37.26	-0.44	17.13	-36.12	41.61
-177.22	265.9	0.39	-16.38	-39.56	57.04	-0.44	17.13	-36.12	52.86
-178.07	266.69	0.47	-19.71	-39.03	34.26	-0.42	16.7	-37.12	43.93
-177.27	266.82	0.46	-18.76	-38.17	57.05	-0.42	16.7	-37.12	50.11
-178.05	267.61	0.39	-16.38	-39.56	37.26	-0.44	17.13	-36.12	41.61
-178.28	268.53	0.39	-16.38	-39.56	57.04	-0.44	17.13	-36.12	52.86
-177.54	268.7	0.47	-19.71	-39.03	34.26	-0.42	16.7	-37.12	43.93
-176.89	268.69	0.46	-18.76	-38.17	57.05	-0.42	16.7	-37.12	50.11
-178.14	269.44	0.39	-16.38	-39.56	37.26	-0.44	17.13	-36.12	41.61
-177.62	269.62	0.39	-16.38	-39.56	57.04	-0.44	17.13	-36.12	52.86
-177.06	269.6	0.47	-19.71	-39.03	34.26	-0.42	16.7	-37.12	43.93
-176.64	269.67	0.46	-18.76	-38.17	57.05	-0.42	16.7	-37.12	50.11
-178.28	270.49	0.39	-16.38	-39.56	37.26	-0.44	17.13	-36.12	41.61
-178.41	270.53	0.39	-16.38	-39.56	57.04	-0.44	17.13	-36.12	52.86
-178.28	270.57	0.47	-19.71	-39.03	34.26	-0.42	16.7	-37.12	43.93
-177.75	270.55	0.46	-18.76	-38.17	57.05	-0.42	16.7	-37.12	50.11
-177.45	270.59	0.39	-16.38	-39.56	37.26	-0.44	17.13	-36.12	41.61
-176.88	270.56	0.39	-16.38	-39.56	57.04	-0.44	17.13	-36.12	52.86
-177.76	271.42	0.47	-19.71	-39.03	34.26	-0.42	16.7	-37.12	43.93
-177.74	271.44	0.46	-18.76	-38.17	57.05	-0.42	16.7	-37.12	50.11
-176.65	271.54	0.39	-16.38	-39.56	37.26	-0.44	17.13	-36.12	41.61
-177.86	273.19	0.39	-16.38	-39.56	57.04	-0.44	17.13	-36.12	52.86
-176.67	273.35	0.47	-19.71	-39.03	34.26	-0.42	16.7	-37.12	43.93
-177.17	274.27	0.46	-18.76	-38.17	57.05	-0.42	16.7	-37.12	50.11
-177.22	274.25	0.39	-16.38	-39.56	37.26	-0.44	17.13	-36.12	41.61
-177.82	274.98	0.39	-16.38	-39.56	57.04	-0.44	17.13	-36.12	52.86
-176.92	275.15	0.47	-19.71	-39.03	34.26	-0.42	16.7	-37.12	43.93
-177.54	275.97	0.46	-18.76	-38.17	57.05	-0.42	16.7	-37.12	50.11
-176.61	276.1	0.39	-16.38	-39.56	37.26	-0.44	17.13	-36.12	41.61
-176.13	276.24	0.39	-16.38	-39.56	57.04	-0.44	17.13	-36.12	52.86
-177.01	276.96	0.47	-19.71	-39.03	34.26	-0.42	16.7	-37.12	43.93
-176.73	276.98	0.46	-18.76	-38.17	57.05	-0.42	16.7	-37.12	50.11
-176.59	277.01	0.39	-16.38	-39.56	37.26	-0.44	17.13	-36.12	41.61
-176.69	277.01	0.39	-16.38	-39.56	57.04	-0.44	17.13	-36.12	52.86
-176.52	277.06	0.47	-19.71	-39.03	34.26	-0.42	16.7	-37.12	43.93
-176.51	277.02	0.46	-18.76	-38.17	57.05	-0.42	16.7	-37.12	50.11
-176.47	276.99	0.39	-16.38	-39.56	37.26	-0.44	17.13	-36.12	41.61
-176.48	276.99	0.39	-16.38	-39.56	57.04	-0.44	17.13	-36.12	52.86
-176.48	276.98	0.47	-19.71	-39.03	34.26	-0.42	16.7	-37.12	43.93
-176.4	276.97	0.46	-18.76	-38.17	57.05	-0.42	16.7	-37.12	50.11
-176.38	276.97	0.39	-16.38	-39.56	37.26	-0.44	17.13	-36.12	41.61
-176.49	276.97	0.39	-16.38	-39.56	57.04	-0.44	17.13	-36.12	52.86
-176.36	277.09	0.47	-19.71	-39.03	34.26	-0.42	16.7	-37.12	43.93

Turtle		SPA120				SPA60			
Y	X	Rot ang	Z	Y	X	Rot ang	Z	Y	X
-176.1	277.17	0.46	-18.76	-38.17	57.05	-0.42	16.7	-37.12	50.11
-176.22	277.09	0.39	-16.38	-39.56	37.26	-0.44	17.13	-36.12	41.61
-177.2	277.88	0.39	-16.38	-39.56	57.04	-0.44	17.13	-36.12	52.86
-176.52	277.95	0.47	-19.71	-39.03	34.26	-0.42	16.7	-37.12	43.93
-176.22	278.86	0.46	-18.76	-38.17	57.05	-0.42	16.7	-37.12	50.11
-175.59	278.93	0.39	-16.38	-39.56	37.26	-0.44	17.13	-36.12	41.61
-175.58	278.87	0.39	-16.38	-39.56	57.04	-0.44	17.13	-36.12	52.86
-176.51	279.7	0.47	-19.71	-39.03	34.26	-0.42	16.7	-37.12	43.93
-175.73	279.78	0.46	-18.76	-38.17	57.05	-0.42	16.7	-37.12	50.11
-177.17	280.58	0.39	-16.38	-39.56	37.26	-0.44	17.13	-36.12	41.61
-176.41	280.67	0.39	-16.38	-39.56	57.04	-0.44	17.13	-36.12	52.86
-175.76	280.7	0.47	-19.71	-39.03	34.26	-0.42	16.7	-37.12	43.93
-176.8	281.54	0.46	-18.76	-38.17	57.05	-0.42	16.7	-37.12	50.11
-176.04	281.61	0.39	-16.38	-39.56	37.26	-0.44	17.13	-36.12	41.61
-176.05	281.63	0.39	-16.38	-39.56	57.04	-0.44	17.13	-36.12	52.86
-175.76	281.61	0.47	-19.71	-39.03	34.26	-0.42	16.7	-37.12	43.93
-174.98	281.69	0.46	-18.76	-38.17	57.05	-0.42	16.7	-37.12	50.11
-175.08	281.63	0.39	-16.38	-39.56	37.26	-0.44	17.13	-36.12	41.61
-174.78	281.61	0.39	-16.38	-39.56	57.04	-0.44	17.13	-36.12	52.86
-174.57	281.58	0.47	-19.71	-39.03	34.26	-0.42	16.7	-37.12	43.93
-175.56	282.28	0.46	-18.76	-38.17	57.05	-0.42	16.7	-37.12	50.11
-174.81	282.57	0.39	-16.38	-39.56	37.26	-0.44	17.13	-36.12	41.61
-175.94	283.36	0.39	-16.38	-39.56	57.04	-0.44	17.13	-36.12	52.86
-175.5	284.14	0.47	-19.71	-39.03	34.26	-0.42	16.7	-37.12	43.93
-174.8	284.46	0.46	-18.76	-38.17	57.05	-0.42	16.7	-37.12	50.11
-174.99	285.21	0.39	-16.38	-39.56	37.26	-0.44	17.13	-36.12	41.61
-175.47	285.79	0.39	-16.38	-39.56	57.04	-0.44	17.13	-36.12	52.86
-174.97	286.18	0.47	-19.71	-39.03	34.26	-0.42	16.7	-37.12	43.93
-175.69	286.88	0.46	-18.76	-38.17	57.05	-0.42	16.7	-37.12	50.11
-175.55	287.14	0.39	-16.38	-39.56	37.26	-0.44	17.13	-36.12	41.61
-175.69	287.14	0.39	-16.38	-39.56	57.04	-0.44	17.13	-36.12	52.86
-175.26	287.2	0.47	-19.71	-39.03	34.26	-0.42	16.7	-37.12	43.93
-175.83	287.89	0.46	-18.76	-38.17	57.05	-0.42	16.7	-37.12	50.11
-175.54	288	0.39	-16.38	-39.56	37.26	-0.44	17.13	-36.12	41.61
-175.64	288.03	0.39	-16.38	-39.56	57.04	-0.44	17.13	-36.12	52.86
-175.44	288.11	0.47	-19.71	-39.03	34.26	-0.42	16.7	-37.12	43.93
-175.47	288.1	0.46	-18.76	-38.17	57.05	-0.42	16.7	-37.12	50.11
-175.63	288.09	0.39	-16.38	-39.56	37.26	-0.44	17.13	-36.12	41.61
-175.72	288.09	0.39	-16.38	-39.56	57.04	-0.44	17.13	-36.12	52.86
-175.71	288.09	0.47	-19.71	-39.03	34.26	-0.42	16.7	-37.12	43.93
-175.81	288.08	0.46	-18.76	-38.17	57.05	-0.42	16.7	-37.12	50.11
-175.82	288.07	0.39	-16.38	-39.56	37.26	-0.44	17.13	-36.12	41.61
-175.84	288.07	0.39	-16.38	-39.56	57.04	-0.44	17.13	-36.12	52.86
-175.83	288.08	0.47	-19.71	-39.03	34.26	-0.42	16.7	-37.12	43.93

Turtle		SPA120				SPA60			
Y	X	Rot ang	Z	Y	X	Rot ang	Z	Y	X
-175.89	288.22	0.46	-18.76	-38.17	57.05	-0.42	16.7	-37.12	50.11
-175.6	288.3	0.39	-16.38	-39.56	37.26	-0.44	17.13	-36.12	41.61
-176.3	288.82	0.39	-16.38	-39.56	57.04	-0.44	17.13	-36.12	52.86
-175.94	289.17	0.47	-19.71	-39.03	34.26	-0.42	16.7	-37.12	43.93
-176.01	289.55	0.46	-18.76	-38.17	57.05	-0.42	16.7	-37.12	50.11
-175.6	289.96	0.39	-16.38	-39.56	37.26	-0.44	17.13	-36.12	41.61
-175.57	290.9	0.39	-16.38	-39.56	57.04	-0.44	17.13	-36.12	52.86
-175.34	291.12	0.47	-19.71	-39.03	34.26	-0.42	16.7	-37.12	43.93
-176.14	291.68	0.46	-18.76	-38.17	57.05	-0.42	16.7	-37.12	50.11
-176.21	291.76	0.39	-16.38	-39.56	37.26	-0.44	17.13	-36.12	41.61
-175.66	291.8	0.39	-16.38	-39.56	57.04	-0.44	17.13	-36.12	52.86
-175.42	291.9	0.47	-19.71	-39.03	34.26	-0.42	16.7	-37.12	43.93
-176.15	292.66	0.46	-18.76	-38.17	57.05	-0.42	16.7	-37.12	50.11
-176.05	292.7	0.39	-16.38	-39.56	37.26	-0.44	17.13	-36.12	41.61
-175.68	292.78	0.39	-16.38	-39.56	57.04	-0.44	17.13	-36.12	52.86
-175.18	293.03	0.47	-19.71	-39.03	34.26	-0.42	16.7	-37.12	43.93
-175.81	293.42	0.46	-18.76	-38.17	57.05	-0.42	16.7	-37.12	50.11
-175.6	293.57	0.39	-16.38	-39.56	37.26	-0.44	17.13	-36.12	41.61
-175.47	293.63	0.39	-16.38	-39.56	57.04	-0.44	17.13	-36.12	52.86
-175.47	293.64	0.47	-19.71	-39.03	34.26	-0.42	16.7	-37.12	43.93
-175.55	293.66	0.46	-18.76	-38.17	57.05	-0.42	16.7	-37.12	50.11
-176.2	294.54	0.39	-16.38	-39.56	37.26	-0.44	17.13	-36.12	41.61
-175.49	294.59	0.39	-16.38	-39.56	57.04	-0.44	17.13	-36.12	52.86
-176.02	295.5	0.47	-19.71	-39.03	34.26	-0.42	16.7	-37.12	43.93
-175.16	295.62	0.46	-18.76	-38.17	57.05	-0.42	16.7	-37.12	50.11
-175.48	296.38	0.39	-16.38	-39.56	37.26	-0.44	17.13	-36.12	41.61
-176.4	297.15	0.39	-16.38	-39.56	57.04	-0.44	17.13	-36.12	52.86
-175.42	297.34	0.47	-19.71	-39.03	34.26	-0.42	16.7	-37.12	43.93
-176.03	298.09	0.46	-18.76	-38.17	57.05	-0.42	16.7	-37.12	50.11
-175.58	298.23	0.39	-16.38	-39.56	37.26	-0.44	17.13	-36.12	41.61
-175.96	299.06	0.39	-16.38	-39.56	57.04	-0.44	17.13	-36.12	52.86
-175.52	299.12	0.47	-19.71	-39.03	34.26	-0.42	16.7	-37.12	43.93
-175.41	299.21	0.46	-18.76	-38.17	57.05	-0.42	16.7	-37.12	50.11
-175.22	299.42	0.39	-16.38	-39.56	37.26	-0.44	17.13	-36.12	41.61
-175.21	299.27	0.39	-16.38	-39.56	57.04	-0.44	17.13	-36.12	52.86
-175.37	299.25	0.47	-19.71	-39.03	34.26	-0.42	16.7	-37.12	43.93
-175.37	299.2	0.46	-18.76	-38.17	57.05	-0.42	16.7	-37.12	50.11
-175.43	299.1	0.39	-16.38	-39.56	37.26	-0.44	17.13	-36.12	41.61
-175.38	299.13	0.39	-16.38	-39.56	57.04	-0.44	17.13	-36.12	52.86

Table S18: Turtle movement actuated by front drive SPA35 and SPA145.

SPA35				SPA145				Turtle	
X	Y	Z	Rot ang	X	Y	Z	Rot ang	X	Y
35.83	-37.33	52.15	-0.95	49.15	-17.14	-45.35	1.21	21.34	-156.05
38.43	-37.33	52.15	-0.95	54.79	-17.14	-45.35	1.21	23.87	-156.24
43.63	-31.29	49.83	-1.01	35	-27.43	-47.92	1.05	26.49	-156.78
37.18	-31.29	49.83	-1.01	54.29	-27.43	-47.92	1.05	29.25	-156.96
32.22	-34.46	48.29	-0.95	40.56	-35	-39.9	0.85	31.66	-156.75
38.35	-34.46	48.29	-0.95	37.91	-35	-39.9	0.85	33.01	-156.59
35.83	-37.33	52.15	-0.95	41.99	-33.86	-49.31	0.97	34.62	-157.22
38.43	-37.33	52.15	-0.95	44.67	-33.86	-49.31	0.97	34.62	-157.22
43.63	-31.29	49.83	-1.01	38.18	-36.74	-44.23	0.88	36.49	-157.69
37.18	-31.29	49.83	-1.01	47.52	-36.74	-44.23	0.88	38.34	-158.09
32.22	-34.46	48.29	-0.95	49.15	-17.14	-45.35	1.21	39.97	-158.57
38.35	-34.46	48.29	-0.95	54.79	-17.14	-45.35	1.21	41.27	-158.82
35.83	-37.33	52.15	-0.95	35	-27.43	-47.92	1.05	43.54	-159.37
38.43	-37.33	52.15	-0.95	54.29	-27.43	-47.92	1.05	44.09	-159.46
43.63	-31.29	49.83	-1.01	40.56	-35	-39.9	0.85	44.52	-159.6
37.18	-31.29	49.83	-1.01	37.91	-35	-39.9	0.85	44.68	-160.01
32.22	-34.46	48.29	-0.95	41.99	-33.86	-49.31	0.97	44.88	-160.2
38.35	-34.46	48.29	-0.95	44.67	-33.86	-49.31	0.97	45.29	-160.23
35.83	-37.33	52.15	-0.95	38.18	-36.74	-44.23	0.88	45.46	-160.33
38.43	-37.33	52.15	-0.95	47.52	-36.74	-44.23	0.88	45.44	-160.26
43.63	-31.29	49.83	-1.01	49.15	-17.14	-45.35	1.21	45.61	-160.39
37.18	-31.29	49.83	-1.01	54.79	-17.14	-45.35	1.21	45.55	-160.27
32.22	-34.46	48.29	-0.95	35	-27.43	-47.92	1.05	45.48	-160.19
38.35	-34.46	48.29	-0.95	54.29	-27.43	-47.92	1.05	45.35	-159.46
35.83	-37.33	52.15	-0.95	40.56	-35	-39.9	0.85	44.44	-158.44
38.43	-37.33	52.15	-0.95	37.91	-35	-39.9	0.85	43.91	-157.68
43.63	-31.29	49.83	-1.01	41.99	-33.86	-49.31	0.97	43.54	-157.32
37.18	-31.29	49.83	-1.01	44.67	-33.86	-49.31	0.97	43.43	-156.76
32.22	-34.46	48.29	-0.95	38.18	-36.74	-44.23	0.88	43.23	-156.39
38.35	-34.46	48.29	-0.95	47.52	-36.74	-44.23	0.88	43.26	-155.91
35.83	-37.33	52.15	-0.95	49.15	-17.14	-45.35	1.21	43.2	-155.81
38.43	-37.33	52.15	-0.95	54.79	-17.14	-45.35	1.21	43.28	-155.81
43.63	-31.29	49.83	-1.01	35	-27.43	-47.92	1.05	43.13	-155.93
37.18	-31.29	49.83	-1.01	54.29	-27.43	-47.92	1.05	42.78	-155.81
32.22	-34.46	48.29	-0.95	40.56	-35	-39.9	0.85	42.6	-155.32
38.35	-34.46	48.29	-0.95	37.91	-35	-39.9	0.85	42.44	-155.02
35.83	-37.33	52.15	-0.95	41.99	-33.86	-49.31	0.97	42.26	-154.76
38.43	-37.33	52.15	-0.95	44.67	-33.86	-49.31	0.97	42.32	-154.46
43.63	-31.29	49.83	-1.01	38.18	-36.74	-44.23	0.88	42.3	-154.17
37.18	-31.29	49.83	-1.01	47.52	-36.74	-44.23	0.88	42.34	-154.06
32.22	-34.46	48.29	-0.95	49.15	-17.14	-45.35	1.21	42.3	-153.8
38.35	-34.46	48.29	-0.95	54.79	-17.14	-45.35	1.21	42.34	-153.88
35.83	-37.33	52.15	-0.95	35	-27.43	-47.92	1.05	42.33	-153.93
38.43	-37.33	52.15	-0.95	54.29	-27.43	-47.92	1.05	42.36	-154
43.63	-31.29	49.83	-1.01	40.56	-35	-39.9	0.85	42.36	-154
37.18	-31.29	49.83	-1.01	37.91	-35	-39.9	0.85	42.66	-154.13

SPA35				SPA145				Turtle	
X	Y	Z	Rot ang	X	Y	Z	Rot ang	X	Y
32.22	-34.46	48.29	-0.95	41.99	-33.86	-49.31	0.97	44.92	-154.88
38.35	-34.46	48.29	-0.95	44.67	-33.86	-49.31	0.97	47.01	-155.64
35.83	-37.33	52.15	-0.95	38.18	-36.74	-44.23	0.88	49.52	-155.84
38.43	-37.33	52.15	-0.95	47.52	-36.74	-44.23	0.88	52.64	-156.64
43.63	-31.29	49.83	-1.01	49.15	-17.14	-45.35	1.21	56.7	-156.4
37.18	-31.29	49.83	-1.01	54.79	-17.14	-45.35	1.21	58.27	-156.65
32.22	-34.46	48.29	-0.95	35	-27.43	-47.92	1.05	59.93	-156.85
38.35	-34.46	48.29	-0.95	54.29	-27.43	-47.92	1.05	61.82	-157.39
35.83	-37.33	52.15	-0.95	40.56	-35	-39.9	0.85	63.63	-157.75
38.43	-37.33	52.15	-0.95	37.91	-35	-39.9	0.85	63.76	-157.55
43.63	-31.29	49.83	-1.01	41.99	-33.86	-49.31	0.97	64.91	-158
37.18	-31.29	49.83	-1.01	44.67	-33.86	-49.31	0.97	66.01	-158.23
32.22	-34.46	48.29	-0.95	38.18	-36.74	-44.23	0.88	67.3	-158.4
38.35	-34.46	48.29	-0.95	47.52	-36.74	-44.23	0.88	68.19	-158.77
35.83	-37.33	52.15	-0.95	49.15	-17.14	-45.35	1.21	68.52	-158.8
38.43	-37.33	52.15	-0.95	54.79	-17.14	-45.35	1.21	68.73	-158.91
43.63	-31.29	49.83	-1.01	35	-27.43	-47.92	1.05	69.11	-159.23
37.18	-31.29	49.83	-1.01	54.29	-27.43	-47.92	1.05	69.39	-159.52
32.22	-34.46	48.29	-0.95	40.56	-35	-39.9	0.85	69.55	-159.7
38.35	-34.46	48.29	-0.95	37.91	-35	-39.9	0.85	69.56	-159.71
35.83	-37.33	52.15	-0.95	41.99	-33.86	-49.31	0.97	69.47	-159.67
38.43	-37.33	52.15	-0.95	44.67	-33.86	-49.31	0.97	69.44	-159.67
43.63	-31.29	49.83	-1.01	38.18	-36.74	-44.23	0.88	69.37	-158.89
37.18	-31.29	49.83	-1.01	47.52	-36.74	-44.23	0.88	69.13	-158.69
32.22	-34.46	48.29	-0.95	49.15	-17.14	-45.35	1.21	68.5	-157.97
38.35	-34.46	48.29	-0.95	54.79	-17.14	-45.35	1.21	68.14	-157.22
35.83	-37.33	52.15	-0.95	35	-27.43	-47.92	1.05	67.95	-156.9
38.43	-37.33	52.15	-0.95	54.29	-27.43	-47.92	1.05	67.72	-156.35
43.63	-31.29	49.83	-1.01	40.56	-35	-39.9	0.85	67.43	-156.1
37.18	-31.29	49.83	-1.01	37.91	-35	-39.9	0.85	67.24	-155.83
32.22	-34.46	48.29	-0.95	41.99	-33.86	-49.31	0.97	67.19	-155.69
38.35	-34.46	48.29	-0.95	44.67	-33.86	-49.31	0.97	67.16	-155.34
35.83	-37.33	52.15	-0.95	38.18	-36.74	-44.23	0.88	67.04	-154.8
38.43	-37.33	52.15	-0.95	47.52	-36.74	-44.23	0.88	67.14	-154.68
43.63	-31.29	49.83	-1.01	49.15	-17.14	-45.35	1.21	67.08	-154.47
37.18	-31.29	49.83	-1.01	54.79	-17.14	-45.35	1.21	67.08	-154.14
32.22	-34.46	48.29	-0.95	35	-27.43	-47.92	1.05	67.21	-153.84
38.35	-34.46	48.29	-0.95	54.29	-27.43	-47.92	1.05	67.2	-153.72
35.83	-37.33	52.15	-0.95	40.56	-35	-39.9	0.85	67.22	-153.6
38.43	-37.33	52.15	-0.95	37.91	-35	-39.9	0.85	67.21	-153.51
43.63	-31.29	49.83	-1.01	41.99	-33.86	-49.31	0.97	67.23	-153.09
37.18	-31.29	49.83	-1.01	44.67	-33.86	-49.31	0.97	67.26	-152.81
32.22	-34.46	48.29	-0.95	38.18	-36.74	-44.23	0.88	67.25	-152.76
38.35	-34.46	48.29	-0.95	47.52	-36.74	-44.23	0.88	67.46	-153.33
35.83	-37.33	52.15	-0.95	49.15	-17.14	-45.35	1.21	69.68	-153.77
38.43	-37.33	52.15	-0.95	54.79	-17.14	-45.35	1.21	69.52	-153.64
43.63	-31.29	49.83	-1.01	35	-27.43	-47.92	1.05	71.68	-154.28
37.18	-31.29	49.83	-1.01	54.29	-27.43	-47.92	1.05	74.49	-154.93

SPA35				SPA145				Turtle	
X	Y	Z	Rot ang	X	Y	Z	Rot ang	X	Y
32.22	-34.46	48.29	-0.95	40.56	-35	-39.9	0.85	77.3	-155.6
38.35	-34.46	48.29	-0.95	37.91	-35	-39.9	0.85	79.91	-155.91
35.83	-37.33	52.15	-0.95	41.99	-33.86	-49.31	0.97	81.7	-155.48
38.43	-37.33	52.15	-0.95	44.67	-33.86	-49.31	0.97	83.27	-155.37
43.63	-31.29	49.83	-1.01	38.18	-36.74	-44.23	0.88	84.91	-155.94
37.18	-31.29	49.83	-1.01	47.52	-36.74	-44.23	0.88	88.37	-156.73
32.22	-34.46	48.29	-0.95	49.15	-17.14	-45.35	1.21	89.89	-156.96
38.35	-34.46	48.29	-0.95	54.79	-17.14	-45.35	1.21	91.15	-157.16
35.83	-37.33	52.15	-0.95	35	-27.43	-47.92	1.05	91.31	-157.37
38.43	-37.33	52.15	-0.95	54.29	-27.43	-47.92	1.05	92.44	-157.6
43.63	-31.29	49.83	-1.01	40.56	-35	-39.9	0.85	93.28	-158
37.18	-31.29	49.83	-1.01	37.91	-35	-39.9	0.85	94.14	-158.27
32.22	-34.46	48.29	-0.95	41.99	-33.86	-49.31	0.97	94.38	-158.35
38.35	-34.46	48.29	-0.95	44.67	-33.86	-49.31	0.97	94.71	-158.43
35.83	-37.33	52.15	-0.95	38.18	-36.74	-44.23	0.88	94.83	-158.78
38.43	-37.33	52.15	-0.95	47.52	-36.74	-44.23	0.88	95.27	-159.12
43.63	-31.29	49.83	-1.01	49.15	-17.14	-45.35	1.21	95.43	-159.13
37.18	-31.29	49.83	-1.01	54.79	-17.14	-45.35	1.21	95.33	-159.12
32.22	-34.46	48.29	-0.95	35	-27.43	-47.92	1.05	95.25	-159.13
38.35	-34.46	48.29	-0.95	54.29	-27.43	-47.92	1.05	95.18	-158.31
35.83	-37.33	52.15	-0.95	40.56	-35	-39.9	0.85	94.93	-157.8
38.43	-37.33	52.15	-0.95	37.91	-35	-39.9	0.85	94.32	-157.25
43.63	-31.29	49.83	-1.01	41.99	-33.86	-49.31	0.97	94	-156.48
37.18	-31.29	49.83	-1.01	44.67	-33.86	-49.31	0.97	93.9	-155.78
32.22	-34.46	48.29	-0.95	38.18	-36.74	-44.23	0.88	93.66	-155.59
38.35	-34.46	48.29	-0.95	47.52	-36.74	-44.23	0.88	93.72	-154.91
35.83	-37.33	52.15	-0.95	49.15	-17.14	-45.35	1.21	93.73	-154.72
38.43	-37.33	52.15	-0.95	54.79	-17.14	-45.35	1.21	93.64	-154.73
43.63	-31.29	49.83	-1.01	35	-27.43	-47.92	1.05	93.46	-154.26
37.18	-31.29	49.83	-1.01	54.29	-27.43	-47.92	1.05	93.58	-153.8
32.22	-34.46	48.29	-0.95	40.56	-35	-39.9	0.85	93.63	-153.64
38.35	-34.46	48.29	-0.95	37.91	-35	-39.9	0.85	93.71	-153.09
35.83	-37.33	52.15	-0.95	41.99	-33.86	-49.31	0.97	93.74	-152.81
38.43	-37.33	52.15	-0.95	44.67	-33.86	-49.31	0.97	93.78	-152.88
43.63	-31.29	49.83	-1.01	38.18	-36.74	-44.23	0.88	93.88	-152.27
37.18	-31.29	49.83	-1.01	47.52	-36.74	-44.23	0.88	93.89	-152.18
32.22	-34.46	48.29	-0.95	49.15	-17.14	-45.35	1.21	93.85	-152.11
38.35	-34.46	48.29	-0.95	54.79	-17.14	-45.35	1.21	93.82	-152.05
35.83	-37.33	52.15	-0.95	35	-27.43	-47.92	1.05	93.84	-152.04
38.43	-37.33	52.15	-0.95	54.29	-27.43	-47.92	1.05	93.9	-151.92
43.63	-31.29	49.83	-1.01	40.56	-35	-39.9	0.85	93.98	-151.5
37.18	-31.29	49.83	-1.01	37.91	-35	-39.9	0.85	96.54	-152.79
32.22	-34.46	48.29	-0.95	41.99	-33.86	-49.31	0.97	99.07	-153.08
38.35	-34.46	48.29	-0.95	44.67	-33.86	-49.31	0.97	101.61	-153.48
35.83	-37.33	52.15	-0.95	38.18	-36.74	-44.23	0.88	104.42	-154.1
38.43	-37.33	52.15	-0.95	47.52	-36.74	-44.23	0.88	104.5	-154.12
43.63	-31.29	49.83	-1.01	49.15	-17.14	-45.35	1.21	107.28	-154.76
37.18	-31.29	49.83	-1.01	54.79	-17.14	-45.35	1.21	109.1	-154.51

SPA35				SPA145				Turtle	
X	Y	Z	Rot ang	X	Y	Z	Rot ang	X	Y
32.22	-34.46	48.29	-0.95	35	-27.43	-47.92	1.05	110.66	-154.3
38.35	-34.46	48.29	-0.95	54.29	-27.43	-47.92	1.05	112.17	-154.68
35.83	-37.33	52.15	-0.95	40.56	-35	-39.9	0.85	113.88	-155.3
38.43	-37.33	52.15	-0.95	37.91	-35	-39.9	0.85	115.47	-155.49
43.63	-31.29	49.83	-1.01	41.99	-33.86	-49.31	0.97	117.08	-155.66
37.18	-31.29	49.83	-1.01	44.67	-33.86	-49.31	0.97	119.18	-156.13
32.22	-34.46	48.29	-0.95	38.18	-36.74	-44.23	0.88	120.06	-156.52
38.35	-34.46	48.29	-0.95	47.52	-36.74	-44.23	0.88	120.76	-156.76
35.83	-37.33	52.15	-0.95	49.15	-17.14	-45.35	1.21	121.36	-156.8
38.43	-37.33	52.15	-0.95	54.79	-17.14	-45.35	1.21	121.49	-156.78
43.63	-31.29	49.83	-1.01	35	-27.43	-47.92	1.05	121.69	-156.94
37.18	-31.29	49.83	-1.01	54.29	-27.43	-47.92	1.05	121.94	-157.16
32.22	-34.46	48.29	-0.95	40.56	-35	-39.9	0.85	122.25	-156.97
38.35	-34.46	48.29	-0.95	37.91	-35	-39.9	0.85	122.49	-156.89
35.83	-37.33	52.15	-0.95	41.99	-33.86	-49.31	0.97	122.79	-156.92
38.43	-37.33	52.15	-0.95	44.67	-33.86	-49.31	0.97	122.71	-156.8
43.63	-31.29	49.83	-1.01	38.18	-36.74	-44.23	0.88	122.28	-156.35
37.18	-31.29	49.83	-1.01	47.52	-36.74	-44.23	0.88	122.22	-155.81
32.22	-34.46	48.29	-0.95	49.15	-17.14	-45.35	1.21	121.78	-155.03
38.35	-34.46	48.29	-0.95	54.79	-17.14	-45.35	1.21	121.5	-154.26
35.83	-37.33	52.15	-0.95	35	-27.43	-47.92	1.05	121.1	-153.35
38.43	-37.33	52.15	-0.95	54.29	-27.43	-47.92	1.05	121.23	-153.31
43.63	-31.29	49.83	-1.01	40.56	-35	-39.9	0.85	120.88	-153.19
37.18	-31.29	49.83	-1.01	37.91	-35	-39.9	0.85	120.79	-152.59
32.22	-34.46	48.29	-0.95	41.99	-33.86	-49.31	0.97	120.49	-152.88
38.35	-34.46	48.29	-0.95	44.67	-33.86	-49.31	0.97	120.54	-152.42
35.83	-37.33	52.15	-0.95	38.18	-36.74	-44.23	0.88	120.64	-151.83
38.43	-37.33	52.15	-0.95	47.52	-36.74	-44.23	0.88	120.47	-151.45
43.63	-31.29	49.83	-1.01	49.15	-17.14	-45.35	1.21	120.59	-151.25
37.18	-31.29	49.83	-1.01	54.79	-17.14	-45.35	1.21	120.6	-150.66
32.22	-34.46	48.29	-0.95	35	-27.43	-47.92	1.05	120.54	-150.54
38.35	-34.46	48.29	-0.95	54.29	-27.43	-47.92	1.05	120.49	-150.49
35.83	-37.33	52.15	-0.95	40.56	-35	-39.9	0.85	120.5	-150.41
38.43	-37.33	52.15	-0.95	37.91	-35	-39.9	0.85	120.47	-150.51
43.63	-31.29	49.83	-1.01	41.99	-33.86	-49.31	0.97	120.54	-150.43
37.18	-31.29	49.83	-1.01	44.67	-33.86	-49.31	0.97	120.49	-150.31
32.22	-34.46	48.29	-0.95	38.18	-36.74	-44.23	0.88	120.46	-150.4
38.35	-34.46	48.29	-0.95	47.52	-36.74	-44.23	0.88	120.55	-150.44
35.83	-37.33	52.15	-0.95	49.15	-17.14	-45.35	1.21	121.11	-150.54
38.43	-37.33	52.15	-0.95	54.79	-17.14	-45.35	1.21	122.99	-151.34
43.63	-31.29	49.83	-1.01	35	-27.43	-47.92	1.05	124.97	-152.04
37.18	-31.29	49.83	-1.01	54.29	-27.43	-47.92	1.05	127.14	-152.08
32.22	-34.46	48.29	-0.95	40.56	-35	-39.9	0.85	130.11	-152.79
38.35	-34.46	48.29	-0.95	37.91	-35	-39.9	0.85	132.51	-153.38
35.83	-37.33	52.15	-0.95	41.99	-33.86	-49.31	0.97	134.55	-153.3
38.43	-37.33	52.15	-0.95	44.67	-33.86	-49.31	0.97	135.96	-153.18
43.63	-31.29	49.83	-1.01	38.18	-36.74	-44.23	0.88	137.36	-153.29
37.18	-31.29	49.83	-1.01	47.52	-36.74	-44.23	0.88	138.84	-153.66

SPA35				SPA145				Turtle	
X	Y	Z	Rot ang	X	Y	Z	Rot ang	X	Y
32.22	-34.46	48.29	-0.95	49.15	-17.14	-45.35	1.21	140.99	-154.01
38.35	-34.46	48.29	-0.95	54.79	-17.14	-45.35	1.21	142.18	-154.53
35.83	-37.33	52.15	-0.95	35	-27.43	-47.92	1.05	143.39	-154.62
38.43	-37.33	52.15	-0.95	54.29	-27.43	-47.92	1.05	144.67	-154.76
43.63	-31.29	49.83	-1.01	40.56	-35	-39.9	0.85	145.41	-155.1
37.18	-31.29	49.83	-1.01	37.91	-35	-39.9	0.85	146.34	-155.31
32.22	-34.46	48.29	-0.95	41.99	-33.86	-49.31	0.97	146.99	-155.38
38.35	-34.46	48.29	-0.95	44.67	-33.86	-49.31	0.97	147.37	-155.42
35.83	-37.33	52.15	-0.95	38.18	-36.74	-44.23	0.88	147.27	-155.8
38.43	-37.33	52.15	-0.95	47.52	-36.74	-44.23	0.88	147.49	-156.15
43.63	-31.29	49.83	-1.01	49.15	-17.14	-45.35	1.21	147.5	-156.18
37.18	-31.29	49.83	-1.01	54.79	-17.14	-45.35	1.21	147.57	-156.22
32.22	-34.46	48.29	-0.95	35	-27.43	-47.92	1.05	147.47	-156.19
38.35	-34.46	48.29	-0.95	54.29	-27.43	-47.92	1.05	147.5	-155.41
35.83	-37.33	52.15	-0.95	40.56	-35	-39.9	0.85	146.9	-155.14
38.43	-37.33	52.15	-0.95	37.91	-35	-39.9	0.85	146.65	-154.43
43.63	-31.29	49.83	-1.01	41.99	-33.86	-49.31	0.97	146.43	-153.71
37.18	-31.29	49.83	-1.01	44.67	-33.86	-49.31	0.97	146.22	-153.4
32.22	-34.46	48.29	-0.95	38.18	-36.74	-44.23	0.88	146.17	-152.78
38.35	-34.46	48.29	-0.95	47.52	-36.74	-44.23	0.88	146.06	-152.46
35.83	-37.33	52.15	-0.95	49.15	-17.14	-45.35	1.21	146.11	-152.4
38.43	-37.33	52.15	-0.95	54.79	-17.14	-45.35	1.21	146.15	-152.46
43.63	-31.29	49.83	-1.01	35	-27.43	-47.92	1.05	146.21	-151.9
37.18	-31.29	49.83	-1.01	54.29	-27.43	-47.92	1.05	146.19	-151.54
32.22	-34.46	48.29	-0.95	40.56	-35	-39.9	0.85	146.34	-151.24
38.35	-34.46	48.29	-0.95	37.91	-35	-39.9	0.85	146.24	-150.97
35.83	-37.33	52.15	-0.95	41.99	-33.86	-49.31	0.97	146.18	-150.56
38.43	-37.33	52.15	-0.95	44.67	-33.86	-49.31	0.97	146.5	-150.43
43.63	-31.29	49.83	-1.01	38.18	-36.74	-44.23	0.88	146.35	-150.24
37.18	-31.29	49.83	-1.01	47.52	-36.74	-44.23	0.88	146.31	-150.07
32.22	-34.46	48.29	-0.95	49.15	-17.14	-45.35	1.21	146.37	-149.99
38.35	-34.46	48.29	-0.95	54.79	-17.14	-45.35	1.21	146.3	-149.8
35.83	-37.33	52.15	-0.95	35	-27.43	-47.92	1.05	146.27	-149.9
38.43	-37.33	52.15	-0.95	54.29	-27.43	-47.92	1.05	146.21	-149.97
43.63	-31.29	49.83	-1.01	40.56	-35	-39.9	0.85	146.25	-149.93
37.18	-31.29	49.83	-1.01	37.91	-35	-39.9	0.85	146.89	-150
32.22	-34.46	48.29	-0.95	41.99	-33.86	-49.31	0.97	148.6	-150.77
38.35	-34.46	48.29	-0.95	44.67	-33.86	-49.31	0.97	150.45	-151.47
35.83	-37.33	52.15	-0.95	38.18	-36.74	-44.23	0.88	153.14	-151.61
38.43	-37.33	52.15	-0.95	47.52	-36.74	-44.23	0.88	155.78	-152.22
43.63	-31.29	49.83	-1.01	49.15	-17.14	-45.35	1.21	157.93	-152.87
37.18	-31.29	49.83	-1.01	54.79	-17.14	-45.35	1.21	159.97	-152.84
32.22	-34.46	48.29	-0.95	35	-27.43	-47.92	1.05	161.32	-152.65
38.35	-34.46	48.29	-0.95	54.29	-27.43	-47.92	1.05	162.85	-152.76
35.83	-37.33	52.15	-0.95	40.56	-35	-39.9	0.85	165.99	-153.76
38.43	-37.33	52.15	-0.95	37.91	-35	-39.9	0.85	165.92	-153.58
43.63	-31.29	49.83	-1.01	41.99	-33.86	-49.31	0.97	167.47	-153.69
37.18	-31.29	49.83	-1.01	44.67	-33.86	-49.31	0.97	168.58	-153.98

SPA35				SPA145				Turtle	
X	Y	Z	Rot ang	X	Y	Z	Rot ang	X	Y
32.22	-34.46	48.29	-0.95	38.18	-36.74	-44.23	0.88	169.83	-154.14
38.35	-34.46	48.29	-0.95	47.52	-36.74	-44.23	0.88	170.93	-154.03
35.83	-37.33	52.15	-0.95	49.15	-17.14	-45.35	1.21	171.91	-154.68
38.43	-37.33	52.15	-0.95	54.79	-17.14	-45.35	1.21	172.18	-154.76
43.63	-31.29	49.83	-1.01	35	-27.43	-47.92	1.05	172.44	-154.85
37.18	-31.29	49.83	-1.01	54.29	-27.43	-47.92	1.05	172.59	-154.83
32.22	-34.46	48.29	-0.95	40.56	-35	-39.9	0.85	173.03	-154.96
38.35	-34.46	48.29	-0.95	37.91	-35	-39.9	0.85	172.56	-155.46
35.83	-37.33	52.15	-0.95	41.99	-33.86	-49.31	0.97	172.43	-155.54
38.43	-37.33	52.15	-0.95	44.67	-33.86	-49.31	0.97	172.33	-155.6
43.63	-31.29	49.83	-1.01	38.18	-36.74	-44.23	0.88	172.31	-154.81
37.18	-31.29	49.83	-1.01	47.52	-36.74	-44.23	0.88	172.12	-154.06
32.22	-34.46	48.29	-0.95	49.15	-17.14	-45.35	1.21	171.99	-153.3
38.35	-34.46	48.29	-0.95	54.79	-17.14	-45.35	1.21	171.68	-152.94
35.83	-37.33	52.15	-0.95	35	-27.43	-47.92	1.05	171.75	-151.85
38.43	-37.33	52.15	-0.95	54.29	-27.43	-47.92	1.05	171.87	-151.41
43.63	-31.29	49.83	-1.01	40.56	-35	-39.9	0.85	171.88	-151.29
37.18	-31.29	49.83	-1.01	37.91	-35	-39.9	0.85	171.89	-151.28
32.22	-34.46	48.29	-0.95	41.99	-33.86	-49.31	0.97	171.84	-151.03
38.35	-34.46	48.29	-0.95	44.67	-33.86	-49.31	0.97	171.93	-150.45
35.83	-37.33	52.15	-0.95	38.18	-36.74	-44.23	0.88	171.91	-150.31
38.43	-37.33	52.15	-0.95	47.52	-36.74	-44.23	0.88	171.9	-150.33
43.63	-31.29	49.83	-1.01	49.15	-17.14	-45.35	1.21	171.96	-150.16
37.18	-31.29	49.83	-1.01	54.79	-17.14	-45.35	1.21	172.13	-149.68
32.22	-34.46	48.29	-0.95	35	-27.43	-47.92	1.05	172.06	-149.46
38.35	-34.46	48.29	-0.95	54.29	-27.43	-47.92	1.05	171.94	-149.28
35.83	-37.33	52.15	-0.95	40.56	-35	-39.9	0.85	171.9	-149.37
38.43	-37.33	52.15	-0.95	37.91	-35	-39.9	0.85	171.97	-149.39
43.63	-31.29	49.83	-1.01	41.99	-33.86	-49.31	0.97	171.92	-149.26
37.18	-31.29	49.83	-1.01	44.67	-33.86	-49.31	0.97	171.94	-149.34
32.22	-34.46	48.29	-0.95	38.18	-36.74	-44.23	0.88	171.91	-149.36
38.35	-34.46	48.29	-0.95	47.52	-36.74	-44.23	0.88	172	-149.26
35.83	-37.33	52.15	-0.95	49.15	-17.14	-45.35	1.21	173.8	-150.22
38.43	-37.33	52.15	-0.95	54.79	-17.14	-45.35	1.21	173.74	-150.24
43.63	-31.29	49.83	-1.01	35	-27.43	-47.92	1.05	175.74	-150.69
37.18	-31.29	49.83	-1.01	54.29	-27.43	-47.92	1.05	178.71	-151.01
32.22	-34.46	48.29	-0.95	40.56	-35	-39.9	0.85	180.93	-151.65
38.35	-34.46	48.29	-0.95	37.91	-35	-39.9	0.85	183.82	-151.97
35.83	-37.33	52.15	-0.95	41.99	-33.86	-49.31	0.97	185.75	-151.73
38.43	-37.33	52.15	-0.95	44.67	-33.86	-49.31	0.97	187.2	-151.52
43.63	-31.29	49.83	-1.01	38.18	-36.74	-44.23	0.88	188.57	-151.42
37.18	-31.29	49.83	-1.01	47.52	-36.74	-44.23	0.88	190.11	-151.92
32.22	-34.46	48.29	-0.95	49.15	-17.14	-45.35	1.21	191.52	-152.1
38.35	-34.46	48.29	-0.95	54.79	-17.14	-45.35	1.21	193.25	-152.16
35.83	-37.33	52.15	-0.95	35	-27.43	-47.92	1.05	195.31	-152.54
38.43	-37.33	52.15	-0.95	54.29	-27.43	-47.92	1.05	195.23	-152.57
43.63	-31.29	49.83	-1.01	40.56	-35	-39.9	0.85	196.27	-152.74
37.18	-31.29	49.83	-1.01	37.91	-35	-39.9	0.85	197.15	-152.93

SPA35				SPA145				Turtle	
X	Y	Z	Rot ang	X	Y	Z	Rot ang	X	Y
32.22	-34.46	48.29	-0.95	41.99	-33.86	-49.31	0.97	197.78	-152.72
38.35	-34.46	48.29	-0.95	44.67	-33.86	-49.31	0.97	197.93	-152.68
35.83	-37.33	52.15	-0.95	38.18	-36.74	-44.23	0.88	198.17	-152.88
38.43	-37.33	52.15	-0.95	47.52	-36.74	-44.23	0.88	198.31	-152.52
43.63	-31.29	49.83	-1.01	49.15	-17.14	-45.35	1.21	198.68	-152.63
37.18	-31.29	49.83	-1.01	54.79	-17.14	-45.35	1.21	198.82	-152.81
32.22	-34.46	48.29	-0.95	35	-27.43	-47.92	1.05	198.59	-152.28
38.35	-34.46	48.29	-0.95	54.29	-27.43	-47.92	1.05	198.6	-152.34
35.83	-37.33	52.15	-0.95	40.56	-35	-39.9	0.85	198.44	-151.57
38.43	-37.33	52.15	-0.95	37.91	-35	-39.9	0.85	198.28	-150.76
43.63	-31.29	49.83	-1.01	41.99	-33.86	-49.31	0.97	198.06	-150.69
37.18	-31.29	49.83	-1.01	44.67	-33.86	-49.31	0.97	198.04	-149.83
32.22	-34.46	48.29	-0.95	38.18	-36.74	-44.23	0.88	198.02	-149.5
38.35	-34.46	48.29	-0.95	47.52	-36.74	-44.23	0.88	198.29	-149.09
35.83	-37.33	52.15	-0.95	49.15	-17.14	-45.35	1.21	198.29	-148.89
38.43	-37.33	52.15	-0.95	54.79	-17.14	-45.35	1.21	198.38	-149.13
43.63	-31.29	49.83	-1.01	35	-27.43	-47.92	1.05	198.31	-148.87
37.18	-31.29	49.83	-1.01	54.29	-27.43	-47.92	1.05	198.44	-148.8
32.22	-34.46	48.29	-0.95	40.56	-35	-39.9	0.85	198.45	-148.53
38.35	-34.46	48.29	-0.95	37.91	-35	-39.9	0.85	198.45	-148.47
35.83	-37.33	52.15	-0.95	41.99	-33.86	-49.31	0.97	198.5	-148.44
38.43	-37.33	52.15	-0.95	44.67	-33.86	-49.31	0.97	198.48	-148.59
43.63	-31.29	49.83	-1.01	38.18	-36.74	-44.23	0.88	198.51	-148.67
37.18	-31.29	49.83	-1.01	47.52	-36.74	-44.23	0.88	198.54	-148.74
32.22	-34.46	48.29	-0.95	49.15	-17.14	-45.35	1.21	198.51	-148.73
38.35	-34.46	48.29	-0.95	54.79	-17.14	-45.35	1.21	198.55	-148.64
35.83	-37.33	52.15	-0.95	35	-27.43	-47.92	1.05	198.5	-148.72
38.43	-37.33	52.15	-0.95	54.29	-27.43	-47.92	1.05	198.6	-148.65
43.63	-31.29	49.83	-1.01	40.56	-35	-39.9	0.85	199.61	-148.71
37.18	-31.29	49.83	-1.01	37.91	-35	-39.9	0.85	201.12	-149.14
32.22	-34.46	48.29	-0.95	41.99	-33.86	-49.31	0.97	203.74	-149.95
38.35	-34.46	48.29	-0.95	44.67	-33.86	-49.31	0.97	206.26	-150.13
35.83	-37.33	52.15	-0.95	38.18	-36.74	-44.23	0.88	209.19	-150.17
38.43	-37.33	52.15	-0.95	47.52	-36.74	-44.23	0.88	209.17	-150.16
43.63	-31.29	49.83	-1.01	49.15	-17.14	-45.35	1.21	212.11	-149.98
37.18	-31.29	49.83	-1.01	54.79	-17.14	-45.35	1.21	214.28	-149.82
32.22	-34.46	48.29	-0.95	35	-27.43	-47.92	1.05	216.09	-149.61
38.35	-34.46	48.29	-0.95	54.29	-27.43	-47.92	1.05	217.49	-149.62
35.83	-37.33	52.15	-0.95	40.56	-35	-39.9	0.85	218.75	-149.65
38.43	-37.33	52.15	-0.95	37.91	-35	-39.9	0.85	220.5	-149.77
43.63	-31.29	49.83	-1.01	41.99	-33.86	-49.31	0.97	221.89	-150.16
37.18	-31.29	49.83	-1.01	44.67	-33.86	-49.31	0.97	222.83	-150.38
32.22	-34.46	48.29	-0.95	38.18	-36.74	-44.23	0.88	223.88	-150.11
38.35	-34.46	48.29	-0.95	47.52	-36.74	-44.23	0.88	225.54	-150.88
35.83	-37.33	52.15	-0.95	49.15	-17.14	-45.35	1.21	226.07	-150.89
38.43	-37.33	52.15	-0.95	54.79	-17.14	-45.35	1.21	226.06	-150.87
43.63	-31.29	49.83	-1.01	35	-27.43	-47.92	1.05	226.31	-151.01
37.18	-31.29	49.83	-1.01	54.29	-27.43	-47.92	1.05	226.33	-151

SPA35				SPA145				Turtle	
X	Y	Z	Rot ang	X	Y	Z	Rot ang	X	Y
32.22	-34.46	48.29	-0.95	40.56	-35	-39.9	0.85	226.56	-151
38.35	-34.46	48.29	-0.95	37.91	-35	-39.9	0.85	226.92	-150.95
35.83	-37.33	52.15	-0.95	41.99	-33.86	-49.31	0.97	227.08	-150.92
38.43	-37.33	52.15	-0.95	44.67	-33.86	-49.31	0.97	227.03	-150.87
43.63	-31.29	49.83	-1.01	38.18	-36.74	-44.23	0.88	227.15	-150.18
37.18	-31.29	49.83	-1.01	47.52	-36.74	-44.23	0.88	226.83	-149.93
32.22	-34.46	48.29	-0.95	49.15	-17.14	-45.35	1.21	226.8	-149.14
38.35	-34.46	48.29	-0.95	54.79	-17.14	-45.35	1.21	226.77	-148.49
35.83	-37.33	52.15	-0.95	35	-27.43	-47.92	1.05	226.76	-148.21
38.43	-37.33	52.15	-0.95	54.29	-27.43	-47.92	1.05	226.77	-148.2
43.63	-31.29	49.83	-1.01	40.56	-35	-39.9	0.85	226.74	-147.82
37.18	-31.29	49.83	-1.01	37.91	-35	-39.9	0.85	226.84	-148.1
32.22	-34.46	48.29	-0.95	41.99	-33.86	-49.31	0.97	226.8	-147.76
38.35	-34.46	48.29	-0.95	44.67	-33.86	-49.31	0.97	226.91	-147.39
35.83	-37.33	52.15	-0.95	38.18	-36.74	-44.23	0.88	227.01	-147.2
38.43	-37.33	52.15	-0.95	47.52	-36.74	-44.23	0.88	227.02	-147.01
43.63	-31.29	49.83	-1.01	49.15	-17.14	-45.35	1.21	227.04	-146.67
37.18	-31.29	49.83	-1.01	54.79	-17.14	-45.35	1.21	226.99	-146.38
32.22	-34.46	48.29	-0.95	35	-27.43	-47.92	1.05	227.09	-146.31
38.35	-34.46	48.29	-0.95	54.29	-27.43	-47.92	1.05	226.93	-146.14
35.83	-37.33	52.15	-0.95	40.56	-35	-39.9	0.85	226.9	-146.21
38.43	-37.33	52.15	-0.95	37.91	-35	-39.9	0.85	226.82	-146.29
43.63	-31.29	49.83	-1.01	41.99	-33.86	-49.31	0.97	226.88	-146.17
37.18	-31.29	49.83	-1.01	44.67	-33.86	-49.31	0.97	226.82	-146.22
32.22	-34.46	48.29	-0.95	38.18	-36.74	-44.23	0.88	226.83	-146.24
38.35	-34.46	48.29	-0.95	47.52	-36.74	-44.23	0.88	227.17	-146.17
35.83	-37.33	52.15	-0.95	49.15	-17.14	-45.35	1.21	227.96	-146.32
38.43	-37.33	52.15	-0.95	54.79	-17.14	-45.35	1.21	229.17	-147.09
43.63	-31.29	49.83	-1.01	35	-27.43	-47.92	1.05	231.25	-147.77
37.18	-31.29	49.83	-1.01	54.29	-27.43	-47.92	1.05	233.68	-147.86
32.22	-34.46	48.29	-0.95	40.56	-35	-39.9	0.85	239.12	-148.44
38.35	-34.46	48.29	-0.95	37.91	-35	-39.9	0.85	241.16	-148.15
35.83	-37.33	52.15	-0.95	41.99	-33.86	-49.31	0.97	242.76	-148.1
38.43	-37.33	52.15	-0.95	44.67	-33.86	-49.31	0.97	242.78	-148.11
43.63	-31.29	49.83	-1.01	38.18	-36.74	-44.23	0.88	244.1	-148.07
37.18	-31.29	49.83	-1.01	47.52	-36.74	-44.23	0.88	245.42	-148.2
32.22	-34.46	48.29	-0.95	49.15	-17.14	-45.35	1.21	247	-148.08
38.35	-34.46	48.29	-0.95	54.79	-17.14	-45.35	1.21	248.33	-148.18
35.83	-37.33	52.15	-0.95	35	-27.43	-47.92	1.05	249.44	-147.87
38.43	-37.33	52.15	-0.95	54.29	-27.43	-47.92	1.05	250.74	-147.98
43.63	-31.29	49.83	-1.01	40.56	-35	-39.9	0.85	251.74	-147.88
37.18	-31.29	49.83	-1.01	37.91	-35	-39.9	0.85	252.64	-147.75
32.22	-34.46	48.29	-0.95	41.99	-33.86	-49.31	0.97	253.35	-147.75
38.35	-34.46	48.29	-0.95	44.67	-33.86	-49.31	0.97	253.61	-147.76
35.83	-37.33	52.15	-0.95	38.18	-36.74	-44.23	0.88	253.75	-147.8
38.43	-37.33	52.15	-0.95	47.52	-36.74	-44.23	0.88	253.86	-147.68
43.63	-31.29	49.83	-1.01	49.15	-17.14	-45.35	1.21	254.19	-147.63
37.18	-31.29	49.83	-1.01	54.79	-17.14	-45.35	1.21	254.31	-147.68

SPA35				SPA145				Turtle	
X	Y	Z	Rot ang	X	Y	Z	Rot ang	X	Y
32.22	-34.46	48.29	-0.95	35	-27.43	-47.92	1.05	254.31	-147.58
38.35	-34.46	48.29	-0.95	54.29	-27.43	-47.92	1.05	254.3	-147.25
35.83	-37.33	52.15	-0.95	40.56	-35	-39.9	0.85	254.09	-146.68
38.43	-37.33	52.15	-0.95	37.91	-35	-39.9	0.85	253.66	-146.57
43.63	-31.29	49.83	-1.01	41.99	-33.86	-49.31	0.97	253.48	-146.34
37.18	-31.29	49.83	-1.01	44.67	-33.86	-49.31	0.97	253.45	-146.46
32.22	-34.46	48.29	-0.95	38.18	-36.74	-44.23	0.88	253.52	-146.31
38.35	-34.46	48.29	-0.95	47.52	-36.74	-44.23	0.88	253.72	-145.86
35.83	-37.33	52.15	-0.95	49.15	-17.14	-45.35	1.21	253.82	-145.15
38.43	-37.33	52.15	-0.95	54.79	-17.14	-45.35	1.21	254	-145.04
43.63	-31.29	49.83	-1.01	35	-27.43	-47.92	1.05	254.24	-144.77
37.18	-31.29	49.83	-1.01	54.29	-27.43	-47.92	1.05	254.32	-144.59
32.22	-34.46	48.29	-0.95	40.56	-35	-39.9	0.85	254.35	-144.64
38.35	-34.46	48.29	-0.95	37.91	-35	-39.9	0.85	254.31	-144.62
35.83	-37.33	52.15	-0.95	41.99	-33.86	-49.31	0.97	254.25	-144.62
38.43	-37.33	52.15	-0.95	44.67	-33.86	-49.31	0.97	254.4	-144.62
43.63	-31.29	49.83	-1.01	38.18	-36.74	-44.23	0.88	254.37	-144.64
37.18	-31.29	49.83	-1.01	47.52	-36.74	-44.23	0.88	254.54	-144.53
32.22	-34.46	48.29	-0.95	49.15	-17.14	-45.35	1.21	254.41	-144.64
38.35	-34.46	48.29	-0.95	54.79	-17.14	-45.35	1.21	254.42	-144.54
35.83	-37.33	52.15	-0.95	35	-27.43	-47.92	1.05	254.46	-144.51
38.43	-37.33	52.15	-0.95	54.29	-27.43	-47.92	1.05	254.33	-144.62
43.63	-31.29	49.83	-1.01	40.56	-35	-39.9	0.85	254.35	-144.58
37.18	-31.29	49.83	-1.01	37.91	-35	-39.9	0.85	254.33	-144.7
32.22	-34.46	48.29	-0.95	41.99	-33.86	-49.31	0.97	256.03	-146.09
38.35	-34.46	48.29	-0.95	44.67	-33.86	-49.31	0.97	257.78	-146.3
35.83	-37.33	52.15	-0.95	38.18	-36.74	-44.23	0.88	259.72	-146.19
38.43	-37.33	52.15	-0.95	47.52	-36.74	-44.23	0.88	262.96	-146.29
43.63	-31.29	49.83	-1.01	49.15	-17.14	-45.35	1.21	264.89	-146.43
37.18	-31.29	49.83	-1.01	54.79	-17.14	-45.35	1.21	268.56	-145.85
32.22	-34.46	48.29	-0.95	35	-27.43	-47.92	1.05	270.09	-145.98
38.35	-34.46	48.29	-0.95	54.29	-27.43	-47.92	1.05	271.67	-146.26
35.83	-37.33	52.15	-0.95	40.56	-35	-39.9	0.85	273.39	-146.42
38.43	-37.33	52.15	-0.95	37.91	-35	-39.9	0.85	273.52	-146.41
43.63	-31.29	49.83	-1.01	41.99	-33.86	-49.31	0.97	274.92	-146.46
37.18	-31.29	49.83	-1.01	44.67	-33.86	-49.31	0.97	275.78	-146.15
32.22	-34.46	48.29	-0.95	38.18	-36.74	-44.23	0.88	276.72	-146.18
38.35	-34.46	48.29	-0.95	47.52	-36.74	-44.23	0.88	277.53	-146.18
35.83	-37.33	52.15	-0.95	49.15	-17.14	-45.35	1.21	278.44	-146.09
38.43	-37.33	52.15	-0.95	54.79	-17.14	-45.35	1.21	279.01	-146.3
43.63	-31.29	49.83	-1.01	35	-27.43	-47.92	1.05	279.31	-146.24
37.18	-31.29	49.83	-1.01	54.29	-27.43	-47.92	1.05	279.33	-146.27
32.22	-34.46	48.29	-0.95	40.56	-35	-39.9	0.85	279.23	-146.19
38.35	-34.46	48.29	-0.95	37.91	-35	-39.9	0.85	279.22	-146.16
35.83	-37.33	52.15	-0.95	41.99	-33.86	-49.31	0.97	279.22	-146.16
38.43	-37.33	52.15	-0.95	44.67	-33.86	-49.31	0.97	279.16	-146.14
43.63	-31.29	49.83	-1.01	38.18	-36.74	-44.23	0.88	279.16	-145.9
37.18	-31.29	49.83	-1.01	47.52	-36.74	-44.23	0.88	278.97	-145.53

SPA35				SPA145				Turtle	
X	Y	Z	Rot ang	X	Y	Z	Rot ang	X	Y
32.22	-34.46	48.29	-0.95	49.15	-17.14	-45.35	1.21	278.92	-145.44
38.35	-34.46	48.29	-0.95	54.79	-17.14	-45.35	1.21	278.94	-145.33
35.83	-37.33	52.15	-0.95	35	-27.43	-47.92	1.05	279.06	-145.27
38.43	-37.33	52.15	-0.95	54.29	-27.43	-47.92	1.05	279.17	-145.36
43.63	-31.29	49.83	-1.01	40.56	-35	-39.9	0.85	279.12	-144.36
37.18	-31.29	49.83	-1.01	37.91	-35	-39.9	0.85	279.2	-144.05
32.22	-34.46	48.29	-0.95	41.99	-33.86	-49.31	0.97	279.22	-143.72
38.35	-34.46	48.29	-0.95	44.67	-33.86	-49.31	0.97	280.29	-142.67
35.83	-37.33	52.15	-0.95	38.18	-36.74	-44.23	0.88	280.51	-142.64
38.43	-37.33	52.15	-0.95	47.52	-36.74	-44.23	0.88	280.66	-142.56
43.63	-31.29	49.83	-1.01	49.15	-17.14	-45.35	1.21	280.67	-142.49
37.18	-31.29	49.83	-1.01	54.79	-17.14	-45.35	1.21	280.78	-142.29
32.22	-34.46	48.29	-0.95	35	-27.43	-47.92	1.05	280.89	-142.25
38.35	-34.46	48.29	-0.95	54.29	-27.43	-47.92	1.05	280.79	-142.32
35.83	-37.33	52.15	-0.95	40.56	-35	-39.9	0.85	280.96	-142.34
38.43	-37.33	52.15	-0.95	37.91	-35	-39.9	0.85	282.02	-142.4
43.63	-31.29	49.83	-1.01	41.99	-33.86	-49.31	0.97	282.19	-142.33
37.18	-31.29	49.83	-1.01	44.67	-33.86	-49.31	0.97	282.27	-142.34
32.22	-34.46	48.29	-0.95	38.18	-36.74	-44.23	0.88	282.34	-142.31
38.35	-34.46	48.29	-0.95	47.52	-36.74	-44.23	0.88	282.42	-142.29
35.83	-37.33	52.15	-0.95	49.15	-17.14	-45.35	1.21	284.02	-142.65
38.43	-37.33	52.15	-0.95	54.79	-17.14	-45.35	1.21	284.07	-142.53
43.63	-31.29	49.83	-1.01	35	-27.43	-47.92	1.05	286.07	-142.5
37.18	-31.29	49.83	-1.01	54.29	-27.43	-47.92	1.05	289.5	-143.6
32.22	-34.46	48.29	-0.95	40.56	-35	-39.9	0.85	291.48	-143.51
38.35	-34.46	48.29	-0.95	37.91	-35	-39.9	0.85	293.31	-143.45
35.83	-37.33	52.15	-0.95	41.99	-33.86	-49.31	0.97	296.88	-143.26
38.43	-37.33	52.15	-0.95	44.67	-33.86	-49.31	0.97	298.37	-143.45
43.63	-31.29	49.83	-1.01	38.18	-36.74	-44.23	0.88	299.13	-143.39
37.18	-31.29	49.83	-1.01	47.52	-36.74	-44.23	0.88	300.42	-143.25
32.22	-34.46	48.29	-0.95	49.15	-17.14	-45.35	1.21	302.06	-143.73
38.35	-34.46	48.29	-0.95	54.79	-17.14	-45.35	1.21	303.72	-144.47
35.83	-37.33	52.15	-0.95	35	-27.43	-47.92	1.05	304.04	-143.41
38.43	-37.33	52.15	-0.95	54.29	-27.43	-47.92	1.05	303.95	-143.67
43.63	-31.29	49.83	-1.01	40.56	-35	-39.9	0.85	305.5	-144.48
37.18	-31.29	49.83	-1.01	37.91	-35	-39.9	0.85	305.68	-143.83
32.22	-34.46	48.29	-0.95	41.99	-33.86	-49.31	0.97	307.07	-144.73
38.35	-34.46	48.29	-0.95	44.67	-33.86	-49.31	0.97	307.33	-144.61
35.83	-37.33	52.15	-0.95	38.18	-36.74	-44.23	0.88	307.46	-144.45
38.43	-37.33	52.15	-0.95	47.52	-36.74	-44.23	0.88	307.54	-144.47
43.63	-31.29	49.83	-1.01	49.15	-17.14	-45.35	1.21	307.52	-144.49
37.18	-31.29	49.83	-1.01	54.79	-17.14	-45.35	1.21	307.52	-144.45
32.22	-34.46	48.29	-0.95	35	-27.43	-47.92	1.05	307.49	-144.53
38.35	-34.46	48.29	-0.95	54.29	-27.43	-47.92	1.05	307.42	-144.4
35.83	-37.33	52.15	-0.95	40.56	-35	-39.9	0.85	307.34	-144.19
38.43	-37.33	52.15	-0.95	37.91	-35	-39.9	0.85	307.36	-144.32
43.63	-31.29	49.83	-1.01	41.99	-33.86	-49.31	0.97	307.31	-144.39
37.18	-31.29	49.83	-1.01	44.67	-33.86	-49.31	0.97	307.34	-144.23

SPA35				SPA145				Turtle	
X	Y	Z	Rot ang	X	Y	Z	Rot ang	X	Y
32.22	-34.46	48.29	-0.95	38.18	-36.74	-44.23	0.88	307.47	-144.16
38.35	-34.46	48.29	-0.95	47.52	-36.74	-44.23	0.88	307.56	-142.73
35.83	-37.33	52.15	-0.95	49.15	-17.14	-45.35	1.21	307.64	-142.61
38.43	-37.33	52.15	-0.95	54.79	-17.14	-45.35	1.21	308.71	-142.63
43.63	-31.29	49.83	-1.01	35	-27.43	-47.92	1.05	308.9	-141.28
37.18	-31.29	49.83	-1.01	54.29	-27.43	-47.92	1.05	309	-141.12
32.22	-34.46	48.29	-0.95	40.56	-35	-39.9	0.85	309.03	-140.99
38.35	-34.46	48.29	-0.95	37.91	-35	-39.9	0.85	309.26	-140.64
35.83	-37.33	52.15	-0.95	41.99	-33.86	-49.31	0.97	309.23	-140.52
38.43	-37.33	52.15	-0.95	44.67	-33.86	-49.31	0.97	309.13	-140.63
43.63	-31.29	49.83	-1.01	38.18	-36.74	-44.23	0.88	309.27	-140.65
37.18	-31.29	49.83	-1.01	47.52	-36.74	-44.23	0.88	309.25	-140.75
32.22	-34.46	48.29	-0.95	49.15	-17.14	-45.35	1.21	310.34	-140.79
38.35	-34.46	48.29	-0.95	54.79	-17.14	-45.35	1.21	310.5	-140.76
35.83	-37.33	52.15	-0.95	35	-27.43	-47.92	1.05	310.65	-140.68
38.43	-37.33	52.15	-0.95	54.29	-27.43	-47.92	1.05	310.74	-140.75
43.63	-31.29	49.83	-1.01	40.56	-35	-39.9	0.85	310.75	-140.79
37.18	-31.29	49.83	-1.01	37.91	-35	-39.9	0.85	312.36	-140.97
32.22	-34.46	48.29	-0.95	41.99	-33.86	-49.31	0.97	314.41	-142.18
38.35	-34.46	48.29	-0.95	44.67	-33.86	-49.31	0.97	317.55	-142.26
35.83	-37.33	52.15	-0.95	38.18	-36.74	-44.23	0.88	319.69	-142.45
38.43	-37.33	52.15	-0.95	47.52	-36.74	-44.23	0.88	319.68	-142.29
43.63	-31.29	49.83	-1.01	49.15	-17.14	-45.35	1.21	321.81	-141.9
37.18	-31.29	49.83	-1.01	54.79	-17.14	-45.35	1.21	323.43	-141.93
32.22	-34.46	48.29	-0.95	35	-27.43	-47.92	1.05	324.87	-141.91
38.35	-34.46	48.29	-0.95	54.29	-27.43	-47.92	1.05	325.35	-141.69
35.83	-37.33	52.15	-0.95	40.56	-35	-39.9	0.85	326.81	-143.07
38.43	-37.33	52.15	-0.95	37.91	-35	-39.9	0.85	329.97	-143.43
43.63	-31.29	49.83	-1.01	41.99	-33.86	-49.31	0.97	330.52	-143.18
37.18	-31.29	49.83	-1.01	44.67	-33.86	-49.31	0.97	331.95	-143.32
32.22	-34.46	48.29	-0.95	38.18	-36.74	-44.23	0.88	332.03	-143.25
38.35	-34.46	48.29	-0.95	47.52	-36.74	-44.23	0.88	332.38	-143.08
35.83	-37.33	52.15	-0.95	49.15	-17.14	-45.35	1.21	333.65	-143.18
38.43	-37.33	52.15	-0.95	54.79	-17.14	-45.35	1.21	333.93	-143.2
43.63	-31.29	49.83	-1.01	35	-27.43	-47.92	1.05	334.12	-143.03
37.18	-31.29	49.83	-1.01	54.29	-27.43	-47.92	1.05	334.14	-143
32.22	-34.46	48.29	-0.95	40.56	-35	-39.9	0.85	334.07	-142.98
38.35	-34.46	48.29	-0.95	37.91	-35	-39.9	0.85	334.17	-142.88
35.83	-37.33	52.15	-0.95	41.99	-33.86	-49.31	0.97	334.2	-142.88
38.43	-37.33	52.15	-0.95	44.67	-33.86	-49.31	0.97	334.09	-142.94
43.63	-31.29	49.83	-1.01	38.18	-36.74	-44.23	0.88	334.02	-142.96
37.18	-31.29	49.83	-1.01	47.52	-36.74	-44.23	0.88	333.95	-143.02
32.22	-34.46	48.29	-0.95	49.15	-17.14	-45.35	1.21	333.9	-141.61
38.35	-34.46	48.29	-0.95	54.79	-17.14	-45.35	1.21	334.15	-141.21
35.83	-37.33	52.15	-0.95	35	-27.43	-47.92	1.05	335.29	-140.79
38.43	-37.33	52.15	-0.95	54.29	-27.43	-47.92	1.05	335.41	-140.94
43.63	-31.29	49.83	-1.01	40.56	-35	-39.9	0.85	335.55	-140.9
37.18	-31.29	49.83	-1.01	37.91	-35	-39.9	0.85	335.63	-140.96

SPA35				SPA145				Turtle	
X	Y	Z	Rot ang	X	Y	Z	Rot ang	X	Y
32.22	-34.46	48.29	-0.95	41.99	-33.86	-49.31	0.97	335.68	-140.88
38.35	-34.46	48.29	-0.95	44.67	-33.86	-49.31	0.97	335.89	-139.4
35.83	-37.33	52.15	-0.95	38.18	-36.74	-44.23	0.88	336.92	-139.52
38.43	-37.33	52.15	-0.95	47.52	-36.74	-44.23	0.88	337.15	-139.33
43.63	-31.29	49.83	-1.01	49.15	-17.14	-45.35	1.21	337.28	-139.22
37.18	-31.29	49.83	-1.01	54.79	-17.14	-45.35	1.21	337.36	-139.19
32.22	-34.46	48.29	-0.95	35	-27.43	-47.92	1.05	337.51	-139.14
38.35	-34.46	48.29	-0.95	54.29	-27.43	-47.92	1.05	337.51	-139.25
35.83	-37.33	52.15	-0.95	40.56	-35	-39.9	0.85	337.64	-139.43
38.43	-37.33	52.15	-0.95	37.91	-35	-39.9	0.85	337.5	-139.38
43.63	-31.29	49.83	-1.01	41.99	-33.86	-49.31	0.97	337.65	-139.61
37.18	-31.29	49.83	-1.01	44.67	-33.86	-49.31	0.97	337.64	-139.48
32.22	-34.46	48.29	-0.95	38.18	-36.74	-44.23	0.88	337.65	-139.49
38.35	-34.46	48.29	-0.95	47.52	-36.74	-44.23	0.88	337.67	-139.43
35.83	-37.33	52.15	-0.95	49.15	-17.14	-45.35	1.21	337.67	-139.3
38.43	-37.33	52.15	-0.95	54.79	-17.14	-45.35	1.21	339.04	-139.16
43.63	-31.29	49.83	-1.01	35	-27.43	-47.92	1.05	340.91	-139.64
37.18	-31.29	49.83	-1.01	54.29	-27.43	-47.92	1.05	342.79	-140.05
32.22	-34.46	48.29	-0.95	40.56	-35	-39.9	0.85	344.67	-139.9
38.35	-34.46	48.29	-0.95	37.91	-35	-39.9	0.85	347.73	-141.23
35.83	-37.33	52.15	-0.95	41.99	-33.86	-49.31	0.97	350.05	-140.74
38.43	-37.33	52.15	-0.95	44.67	-33.86	-49.31	0.97	349.95	-140.51
43.63	-31.29	49.83	-1.01	38.18	-36.74	-44.23	0.88	351.49	-141.03
37.18	-31.29	49.83	-1.01	47.52	-36.74	-44.23	0.88	351.79	-140.79
32.22	-34.46	48.29	-0.95	49.15	-17.14	-45.35	1.21	353.34	-141.12
38.35	-34.46	48.29	-0.95	54.79	-17.14	-45.35	1.21	355	-142.13
35.83	-37.33	52.15	-0.95	35	-27.43	-47.92	1.05	355.24	-141.35
38.43	-37.33	52.15	-0.95	54.29	-27.43	-47.92	1.05	356.71	-142.5
43.63	-31.29	49.83	-1.01	40.56	-35	-39.9	0.85	357.07	-142.25
37.18	-31.29	49.83	-1.01	37.91	-35	-39.9	0.85	358.71	-142.81
32.22	-34.46	48.29	-0.95	41.99	-33.86	-49.31	0.97	358.83	-142.4
38.35	-34.46	48.29	-0.95	44.67	-33.86	-49.31	0.97	358.92	-142.32
35.83	-37.33	52.15	-0.95	38.18	-36.74	-44.23	0.88	358.9	-142.38
38.43	-37.33	52.15	-0.95	47.52	-36.74	-44.23	0.88	358.86	-142.42
43.63	-31.29	49.83	-1.01	49.15	-17.14	-45.35	1.21	358.92	-142.29
37.18	-31.29	49.83	-1.01	54.79	-17.14	-45.35	1.21	358.91	-142.24
32.22	-34.46	48.29	-0.95	35	-27.43	-47.92	1.05	358.95	-142.26
38.35	-34.46	48.29	-0.95	54.29	-27.43	-47.92	1.05	358.79	-142.48
35.83	-37.33	52.15	-0.95	40.56	-35	-39.9	0.85	358.72	-142.41
38.43	-37.33	52.15	-0.95	37.91	-35	-39.9	0.85	358.78	-140.92
43.63	-31.29	49.83	-1.01	41.99	-33.86	-49.31	0.97	358.79	-140.66
37.18	-31.29	49.83	-1.01	44.67	-33.86	-49.31	0.97	360.25	-140.27
32.22	-34.46	48.29	-0.95	38.18	-36.74	-44.23	0.88	360.27	-139.13
38.35	-34.46	48.29	-0.95	47.52	-36.74	-44.23	0.88	360.34	-139
35.83	-37.33	52.15	-0.95	49.15	-17.14	-45.35	1.21	360.51	-138.79
38.43	-37.33	52.15	-0.95	54.79	-17.14	-45.35	1.21	360.45	-138.82
43.63	-31.29	49.83	-1.01	35	-27.43	-47.92	1.05	360.63	-138.59
37.18	-31.29	49.83	-1.01	54.29	-27.43	-47.92	1.05	361.7	-138.79

SPA35				SPA145				Turtle	
X	Y	Z	Rot ang	X	Y	Z	Rot ang	X	Y
32.22	-34.46	48.29	-0.95	40.56	-35	-39.9	0.85	361.96	-138.79
38.35	-34.46	48.29	-0.95	37.91	-35	-39.9	0.85	362.13	-138.73
35.83	-37.33	52.15	-0.95	41.99	-33.86	-49.31	0.97	362.2	-138.64
38.43	-37.33	52.15	-0.95	44.67	-33.86	-49.31	0.97	362.43	-138.61
43.63	-31.29	49.83	-1.01	38.18	-36.74	-44.23	0.88	362.3	-138.66
37.18	-31.29	49.83	-1.01	47.52	-36.74	-44.23	0.88	362.44	-138.77
32.22	-34.46	48.29	-0.95	49.15	-17.14	-45.35	1.21	363.04	-138.75
38.35	-34.46	48.29	-0.95	54.79	-17.14	-45.35	1.21	363.41	-138.76
35.83	-37.33	52.15	-0.95	35	-27.43	-47.92	1.05	363.65	-138.7
38.43	-37.33	52.15	-0.95	54.29	-27.43	-47.92	1.05	363.72	-138.73
43.63	-31.29	49.83	-1.01	40.56	-35	-39.9	0.85	363.89	-138.83
37.18	-31.29	49.83	-1.01	37.91	-35	-39.9	0.85	363.91	-138.72
32.22	-34.46	48.29	-0.95	41.99	-33.86	-49.31	0.97	364.91	-138.47
38.35	-34.46	48.29	-0.95	44.67	-33.86	-49.31	0.97	366.97	-138.57
35.83	-37.33	52.15	-0.95	38.18	-36.74	-44.23	0.88	369.26	-138.75
38.43	-37.33	52.15	-0.95	47.52	-36.74	-44.23	0.88	374.59	-139.72
43.63	-31.29	49.83	-1.01	49.15	-17.14	-45.35	1.21	376.22	-140.1
37.18	-31.29	49.83	-1.01	54.79	-17.14	-45.35	1.21	377.79	-139.94
32.22	-34.46	48.29	-0.95	35	-27.43	-47.92	1.05	378.26	-139.71
38.35	-34.46	48.29	-0.95	54.29	-27.43	-47.92	1.05	379.53	-139.75
35.83	-37.33	52.15	-0.95	40.56	-35	-39.9	0.85	379.99	-139.63
38.43	-37.33	52.15	-0.95	37.91	-35	-39.9	0.85	379.98	-139.62
43.63	-31.29	49.83	-1.01	41.99	-33.86	-49.31	0.97	381.59	-140.81
37.18	-31.29	49.83	-1.01	44.67	-33.86	-49.31	0.97	381.85	-140.39
32.22	-34.46	48.29	-0.95	38.18	-36.74	-44.23	0.88	383.26	-140.96
38.35	-34.46	48.29	-0.95	47.52	-36.74	-44.23	0.88	383.55	-141.15
35.83	-37.33	52.15	-0.95	49.15	-17.14	-45.35	1.21	383.66	-141.12
38.43	-37.33	52.15	-0.95	54.79	-17.14	-45.35	1.21	383.9	-140.94
43.63	-31.29	49.83	-1.01	35	-27.43	-47.92	1.05	385.06	-142.71
37.18	-31.29	49.83	-1.01	54.29	-27.43	-47.92	1.05	385.29	-142.78
32.22	-34.46	48.29	-0.95	40.56	-35	-39.9	0.85	385.35	-142.78
38.35	-34.46	48.29	-0.95	37.91	-35	-39.9	0.85	385.42	-142.69
35.83	-37.33	52.15	-0.95	41.99	-33.86	-49.31	0.97	385.47	-142.66
38.43	-37.33	52.15	-0.95	44.67	-33.86	-49.31	0.97	385.5	-142.6
43.63	-31.29	49.83	-1.01	38.18	-36.74	-44.23	0.88	385.42	-142.73
37.18	-31.29	49.83	-1.01	47.52	-36.74	-44.23	0.88	385.45	-141.9
32.22	-34.46	48.29	-0.95	49.15	-17.14	-45.35	1.21	385.67	-140.9
38.35	-34.46	48.29	-0.95	54.79	-17.14	-45.35	1.21	386.82	-140.66
35.83	-37.33	52.15	-0.95	35	-27.43	-47.92	1.05	387.02	-140.57
38.43	-37.33	52.15	-0.95	54.29	-27.43	-47.92	1.05	387.04	-139.48
43.63	-31.29	49.83	-1.01	40.56	-35	-39.9	0.85	387.13	-139.04
37.18	-31.29	49.83	-1.01	37.91	-35	-39.9	0.85	388.47	-139.15
32.22	-34.46	48.29	-0.95	41.99	-33.86	-49.31	0.97	388.68	-138.92
38.35	-34.46	48.29	-0.95	44.67	-33.86	-49.31	0.97	388.74	-137.54
35.83	-37.33	52.15	-0.95	38.18	-36.74	-44.23	0.88	388.83	-137.83
38.43	-37.33	52.15	-0.95	47.52	-36.74	-44.23	0.88	388.94	-137.41
43.63	-31.29	49.83	-1.01	49.15	-17.14	-45.35	1.21	388.84	-137.36
37.18	-31.29	49.83	-1.01	54.79	-17.14	-45.35	1.21	388.93	-137.54

SPA35				SPA145				Turtle	
X	Y	Z	Rot ang	X	Y	Z	Rot ang	X	Y
32.22	-34.46	48.29	-0.95	35	-27.43	-47.92	1.05	390.28	-137.37
38.35	-34.46	48.29	-0.95	54.29	-27.43	-47.92	1.05	390.34	-137.33
35.83	-37.33	52.15	-0.95	40.56	-35	-39.9	0.85	390.39	-137.44
38.43	-37.33	52.15	-0.95	37.91	-35	-39.9	0.85	390.45	-137.41
43.63	-31.29	49.83	-1.01	41.99	-33.86	-49.31	0.97	390.5	-137.32
37.18	-31.29	49.83	-1.01	44.67	-33.86	-49.31	0.97	390.72	-137.29
32.22	-34.46	48.29	-0.95	38.18	-36.74	-44.23	0.88	390.69	-137.25
38.35	-34.46	48.29	-0.95	47.52	-36.74	-44.23	0.88	390.76	-137.16
35.83	-37.33	52.15	-0.95	49.15	-17.14	-45.35	1.21	392.03	-137.24
38.43	-37.33	52.15	-0.95	54.79	-17.14	-45.35	1.21	393.95	-137.07
43.63	-31.29	49.83	-1.01	35	-27.43	-47.92	1.05	393.95	-137.04
37.18	-31.29	49.83	-1.01	54.29	-27.43	-47.92	1.05	395.89	-136.97
32.22	-34.46	48.29	-0.95	40.56	-35	-39.9	0.85	397.7	-137.06
38.35	-34.46	48.29	-0.95	37.91	-35	-39.9	0.85	399.48	-136.83
35.83	-37.33	52.15	-0.95	41.99	-33.86	-49.31	0.97	401.31	-137.52
38.43	-37.33	52.15	-0.95	44.67	-33.86	-49.31	0.97	402.87	-138.18
43.63	-31.29	49.83	-1.01	38.18	-36.74	-44.23	0.88	403.17	-138.12
37.18	-31.29	49.83	-1.01	47.52	-36.74	-44.23	0.88	404.86	-138.56
32.22	-34.46	48.29	-0.95	49.15	-17.14	-45.35	1.21	406.5	-139.48
38.35	-34.46	48.29	-0.95	54.79	-17.14	-45.35	1.21	406.95	-139.67
35.83	-37.33	52.15	-0.95	35	-27.43	-47.92	1.05	408.09	-140.97
38.43	-37.33	52.15	-0.95	54.29	-27.43	-47.92	1.05	408.4	-140.55
43.63	-31.29	49.83	-1.01	40.56	-35	-39.9	0.85	408.39	-140.58
37.18	-31.29	49.83	-1.01	37.91	-35	-39.9	0.85	408.55	-140.72
32.22	-34.46	48.29	-0.95	41.99	-33.86	-49.31	0.97	409.1	-141.21
38.35	-34.46	48.29	-0.95	44.67	-33.86	-49.31	0.97	409.44	-141.25
35.83	-37.33	52.15	-0.95	38.18	-36.74	-44.23	0.88	409.39	-141.41
38.43	-37.33	52.15	-0.95	47.52	-36.74	-44.23	0.88	409.4	-141.41
43.63	-31.29	49.83	-1.01	49.15	-17.14	-45.35	1.21	409.39	-141.45
37.18	-31.29	49.83	-1.01	54.79	-17.14	-45.35	1.21	409.45	-141.35
32.22	-34.46	48.29	-0.95	35	-27.43	-47.92	1.05	409.38	-141.33
38.35	-34.46	48.29	-0.95	54.29	-27.43	-47.92	1.05	409.84	-141.23
35.83	-37.33	52.15	-0.95	40.56	-35	-39.9	0.85	410.74	-140.29
38.43	-37.33	52.15	-0.95	37.91	-35	-39.9	0.85	410.96	-139.74
43.63	-31.29	49.83	-1.01	41.99	-33.86	-49.31	0.97	410.97	-139.67
37.18	-31.29	49.83	-1.01	44.67	-33.86	-49.31	0.97	411.05	-139.46
32.22	-34.46	48.29	-0.95	38.18	-36.74	-44.23	0.88	411.06	-139.1
38.35	-34.46	48.29	-0.95	47.52	-36.74	-44.23	0.88	411.82	-138.74
35.83	-37.33	52.15	-0.95	49.15	-17.14	-45.35	1.21	412.18	-138.45
38.43	-37.33	52.15	-0.95	54.79	-17.14	-45.35	1.21	412.33	-138.63
43.63	-31.29	49.83	-1.01	35	-27.43	-47.92	1.05	412.72	-138
37.18	-31.29	49.83	-1.01	54.29	-27.43	-47.92	1.05	413.63	-137.87
32.22	-34.46	48.29	-0.95	40.56	-35	-39.9	0.85	414.05	-137.75
38.35	-34.46	48.29	-0.95	37.91	-35	-39.9	0.85	414.41	-137.59
35.83	-37.33	52.15	-0.95	41.99	-33.86	-49.31	0.97	414.52	-137.65
38.43	-37.33	52.15	-0.95	44.67	-33.86	-49.31	0.97	414.75	-137.54
43.63	-31.29	49.83	-1.01	38.18	-36.74	-44.23	0.88	414.61	-137.56
37.18	-31.29	49.83	-1.01	47.52	-36.74	-44.23	0.88	414.69	-137.6

SPA35				SPA145				Turtle	
X	Y	Z	Rot ang	X	Y	Z	Rot ang	X	Y
32.22	-34.46	48.29	-0.95	49.15	-17.14	-45.35	1.21	414.8	-137.58
38.35	-34.46	48.29	-0.95	54.79	-17.14	-45.35	1.21	415.01	-137.55
35.83	-37.33	52.15	-0.95	35	-27.43	-47.92	1.05	415.52	-137.6
38.43	-37.33	52.15	-0.95	54.29	-27.43	-47.92	1.05	415.87	-137.59
43.63	-31.29	49.83	-1.01	40.56	-35	-39.9	0.85	416.14	-137.58
37.18	-31.29	49.83	-1.01	37.91	-35	-39.9	0.85	416.59	-137.47
32.22	-34.46	48.29	-0.95	41.99	-33.86	-49.31	0.97	418.75	-137.81
38.35	-34.46	48.29	-0.95	44.67	-33.86	-49.31	0.97	421.54	-138.1
35.83	-37.33	52.15	-0.95	38.18	-36.74	-44.23	0.88	423.49	-138.75
38.43	-37.33	52.15	-0.95	47.52	-36.74	-44.23	0.88	425.28	-139.07
43.63	-31.29	49.83	-1.01	49.15	-17.14	-45.35	1.21	425.23	-138.94
37.18	-31.29	49.83	-1.01	54.79	-17.14	-45.35	1.21	426.89	-139.45
32.22	-34.46	48.29	-0.95	35	-27.43	-47.92	1.05	427.79	-139.87
38.35	-34.46	48.29	-0.95	54.29	-27.43	-47.92	1.05	428.73	-140.03
35.83	-37.33	52.15	-0.95	40.56	-35	-39.9	0.85	429.4	-140.52
38.43	-37.33	52.15	-0.95	37.91	-35	-39.9	0.85	430.58	-140.84
43.63	-31.29	49.83	-1.01	41.99	-33.86	-49.31	0.97	431.56	-141.19
37.18	-31.29	49.83	-1.01	44.67	-33.86	-49.31	0.97	433.03	-141.89
32.22	-34.46	48.29	-0.95	38.18	-36.74	-44.23	0.88	433.43	-142.1
38.35	-34.46	48.29	-0.95	47.52	-36.74	-44.23	0.88	434.13	-142.5
35.83	-37.33	52.15	-0.95	49.15	-17.14	-45.35	1.21	434.39	-142.54
38.43	-37.33	52.15	-0.95	54.79	-17.14	-45.35	1.21	434.51	-142.66
43.63	-31.29	49.83	-1.01	35	-27.43	-47.92	1.05	434.4	-142.68
37.18	-31.29	49.83	-1.01	54.29	-27.43	-47.92	1.05	434.36	-142.66
32.22	-34.46	48.29	-0.95	40.56	-35	-39.9	0.85	434.35	-142.67
38.35	-34.46	48.29	-0.95	37.91	-35	-39.9	0.85	434.31	-142.76
35.83	-37.33	52.15	-0.95	41.99	-33.86	-49.31	0.97	434.3	-142.71
38.43	-37.33	52.15	-0.95	44.67	-33.86	-49.31	0.97	434.31	-142.74
43.63	-31.29	49.83	-1.01	38.18	-36.74	-44.23	0.88	434.49	-141.88
37.18	-31.29	49.83	-1.01	47.52	-36.74	-44.23	0.88	435.18	-141.2
32.22	-34.46	48.29	-0.95	49.15	-17.14	-45.35	1.21	435.82	-140.9
38.35	-34.46	48.29	-0.95	54.79	-17.14	-45.35	1.21	435.98	-140.49
35.83	-37.33	52.15	-0.95	35	-27.43	-47.92	1.05	436.04	-139.91
38.43	-37.33	52.15	-0.95	54.29	-27.43	-47.92	1.05	436.74	-139.81
43.63	-31.29	49.83	-1.01	40.56	-35	-39.9	0.85	436.75	-139.85
37.18	-31.29	49.83	-1.01	37.91	-35	-39.9	0.85	436.91	-139.57
32.22	-34.46	48.29	-0.95	41.99	-33.86	-49.31	0.97	437.49	-139.78
38.35	-34.46	48.29	-0.95	44.67	-33.86	-49.31	0.97	437.78	-139.06
35.83	-37.33	52.15	-0.95	38.18	-36.74	-44.23	0.88	438.2	-139.33
38.43	-37.33	52.15	-0.95	47.52	-36.74	-44.23	0.88	438.61	-138.89
43.63	-31.29	49.83	-1.01	49.15	-17.14	-45.35	1.21	439.01	-139.15
37.18	-31.29	49.83	-1.01	54.79	-17.14	-45.35	1.21	439.4	-138.88
32.22	-34.46	48.29	-0.95	35	-27.43	-47.92	1.05	439.52	-138.75
38.35	-34.46	48.29	-0.95	54.29	-27.43	-47.92	1.05	440.04	-138.77
35.83	-37.33	52.15	-0.95	40.56	-35	-39.9	0.85	440.51	-138.58
38.43	-37.33	52.15	-0.95	37.91	-35	-39.9	0.85	440.79	-138.68
43.63	-31.29	49.83	-1.01	41.99	-33.86	-49.31	0.97	440.95	-138.7
37.18	-31.29	49.83	-1.01	44.67	-33.86	-49.31	0.97	441.14	-138.68

SPA35				SPA145				Turtle	
X	Y	Z	Rot ang	X	Y	Z	Rot ang	X	Y
32.22	-34.46	48.29	-0.95	38.18	-36.74	-44.23	0.88	441.21	-138.52
38.35	-34.46	48.29	-0.95	47.52	-36.74	-44.23	0.88	441.33	-138.6
35.83	-37.33	52.15	-0.95	49.15	-17.14	-45.35	1.21	441.31	-138.64
38.43	-37.33	52.15	-0.95	54.79	-17.14	-45.35	1.21	442.1	-138.88
43.63	-31.29	49.83	-1.01	35	-27.43	-47.92	1.05	443.94	-139.27
37.18	-31.29	49.83	-1.01	54.29	-27.43	-47.92	1.05	448.22	-140.44
32.22	-34.46	48.29	-0.95	40.56	-35	-39.9	0.85	450.23	-140.54
38.35	-34.46	48.29	-0.95	37.91	-35	-39.9	0.85	451.69	-141.26
35.83	-37.33	52.15	-0.95	41.99	-33.86	-49.31	0.97	452.7	-141.65
38.43	-37.33	52.15	-0.95	44.67	-33.86	-49.31	0.97	453.56	-141.77
43.63	-31.29	49.83	-1.01	38.18	-36.74	-44.23	0.88	453.64	-141.7
37.18	-31.29	49.83	-1.01	47.52	-36.74	-44.23	0.88	454.03	-141.67
32.22	-34.46	48.29	-0.95	49.15	-17.14	-45.35	1.21	455.42	-142.73
38.35	-34.46	48.29	-0.95	54.79	-17.14	-45.35	1.21	456.29	-143.07
35.83	-37.33	52.15	-0.95	35	-27.43	-47.92	1.05	457.06	-143.32
38.43	-37.33	52.15	-0.95	54.29	-27.43	-47.92	1.05	457.44	-143.41
43.63	-31.29	49.83	-1.01	40.56	-35	-39.9	0.85	458.11	-143.96
37.18	-31.29	49.83	-1.01	37.91	-35	-39.9	0.85	459.02	-144.76
32.22	-34.46	48.29	-0.95	41.99	-33.86	-49.31	0.97	459.21	-144.79
38.35	-34.46	48.29	-0.95	44.67	-33.86	-49.31	0.97	459.28	-144.79
35.83	-37.33	52.15	-0.95	38.18	-36.74	-44.23	0.88	459.26	-144.83
38.43	-37.33	52.15	-0.95	47.52	-36.74	-44.23	0.88	459.24	-144.84
43.63	-31.29	49.83	-1.01	49.15	-17.14	-45.35	1.21	459.24	-144.84
37.18	-31.29	49.83	-1.01	54.79	-17.14	-45.35	1.21	459.25	-144.86
32.22	-34.46	48.29	-0.95	35	-27.43	-47.92	1.05	459.25	-144.85
38.35	-34.46	48.29	-0.95	54.29	-27.43	-47.92	1.05	459.4	-144.59
35.83	-37.33	52.15	-0.95	40.56	-35	-39.9	0.85	460.79	-143.47
38.43	-37.33	52.15	-0.95	37.91	-35	-39.9	0.85	460.99	-142.85
43.63	-31.29	49.83	-1.01	41.99	-33.86	-49.31	0.97	461.02	-142.5
37.18	-31.29	49.83	-1.01	44.67	-33.86	-49.31	0.97	461.52	-142.3
32.22	-34.46	48.29	-0.95	38.18	-36.74	-44.23	0.88	462.34	-141.98
38.35	-34.46	48.29	-0.95	47.52	-36.74	-44.23	0.88	462.61	-141.87
35.83	-37.33	52.15	-0.95	49.15	-17.14	-45.35	1.21	462.79	-141.53
38.43	-37.33	52.15	-0.95	54.79	-17.14	-45.35	1.21	463.2	-141.22
43.63	-31.29	49.83	-1.01	35	-27.43	-47.92	1.05	463.36	-141.15
37.18	-31.29	49.83	-1.01	54.29	-27.43	-47.92	1.05	464.18	-141.03
32.22	-34.46	48.29	-0.95	40.56	-35	-39.9	0.85	464.5	-140.57
38.35	-34.46	48.29	-0.95	37.91	-35	-39.9	0.85	464.56	-140.39
35.83	-37.33	52.15	-0.95	41.99	-33.86	-49.31	0.97	465.3	-140.22
38.43	-37.33	52.15	-0.95	44.67	-33.86	-49.31	0.97	465.38	-140.09
43.63	-31.29	49.83	-1.01	38.18	-36.74	-44.23	0.88	465.77	-140.08
37.18	-31.29	49.83	-1.01	47.52	-36.74	-44.23	0.88	466.17	-139.91
32.22	-34.46	48.29	-0.95	49.15	-17.14	-45.35	1.21	466.26	-139.9
38.35	-34.46	48.29	-0.95	54.79	-17.14	-45.35	1.21	466.68	-139.96
35.83	-37.33	52.15	-0.95	35	-27.43	-47.92	1.05	466.99	-139.91
38.43	-37.33	52.15	-0.95	54.29	-27.43	-47.92	1.05	467.06	-139.73
43.63	-31.29	49.83	-1.01	40.56	-35	-39.9	0.85	467.01	-139.83
37.18	-31.29	49.83	-1.01	37.91	-35	-39.9	0.85	467.02	-139.9

SPA35				SPA145				Turtle	
X	Y	Z	Rot ang	X	Y	Z	Rot ang	X	Y
32.22	-34.46	48.29	-0.95	41.99	-33.86	-49.31	0.97	467.67	-140.05
38.35	-34.46	48.29	-0.95	44.67	-33.86	-49.31	0.97	469.49	-140.72
35.83	-37.33	52.15	-0.95	38.18	-36.74	-44.23	0.88	473.65	-141.38
38.43	-37.33	52.15	-0.95	47.52	-36.74	-44.23	0.88	475.28	-141.9
43.63	-31.29	49.83	-1.01	49.15	-17.14	-45.35	1.21	476.9	-142.55
37.18	-31.29	49.83	-1.01	54.79	-17.14	-45.35	1.21	477.81	-142.94
32.22	-34.46	48.29	-0.95	35	-27.43	-47.92	1.05	478.4	-143.23
38.35	-34.46	48.29	-0.95	54.29	-27.43	-47.92	1.05	479.41	-143.63
35.83	-37.33	52.15	-0.95	40.56	-35	-39.9	0.85	480.35	-143.91
38.43	-37.33	52.15	-0.95	37.91	-35	-39.9	0.85	481.18	-144.57
43.63	-31.29	49.83	-1.01	41.99	-33.86	-49.31	0.97	481.31	-144.54
37.18	-31.29	49.83	-1.01	44.67	-33.86	-49.31	0.97	482.25	-144.98
32.22	-34.46	48.29	-0.95	38.18	-36.74	-44.23	0.88	482.47	-145.31
38.35	-34.46	48.29	-0.95	47.52	-36.74	-44.23	0.88	482.61	-145.4
35.83	-37.33	52.15	-0.95	49.15	-17.14	-45.35	1.21	483.38	-146.14
38.43	-37.33	52.15	-0.95	54.79	-17.14	-45.35	1.21	483.41	-146.35
43.63	-31.29	49.83	-1.01	35	-27.43	-47.92	1.05	483.35	-146.29
37.18	-31.29	49.83	-1.01	54.29	-27.43	-47.92	1.05	483.3	-146.36
32.22	-34.46	48.29	-0.95	40.56	-35	-39.9	0.85	483.29	-146.28
38.35	-34.46	48.29	-0.95	37.91	-35	-39.9	0.85	483.28	-146.44
35.83	-37.33	52.15	-0.95	41.99	-33.86	-49.31	0.97	483.28	-146.32
38.43	-37.33	52.15	-0.95	44.67	-33.86	-49.31	0.97	483.65	-145.88
43.63	-31.29	49.83	-1.01	38.18	-36.74	-44.23	0.88	483.48	-146
37.18	-31.29	49.83	-1.01	47.52	-36.74	-44.23	0.88	483.83	-145.43
32.22	-34.46	48.29	-0.95	49.15	-17.14	-45.35	1.21	484.84	-144.86
38.35	-34.46	48.29	-0.95	54.79	-17.14	-45.35	1.21	485.16	-144.21
35.83	-37.33	52.15	-0.95	35	-27.43	-47.92	1.05	485.78	-143.97
38.43	-37.33	52.15	-0.95	54.29	-27.43	-47.92	1.05	486.06	-143.33
43.63	-31.29	49.83	-1.01	40.56	-35	-39.9	0.85	486.67	-143.11
37.18	-31.29	49.83	-1.01	37.91	-35	-39.9	0.85	486.8	-142.67
32.22	-34.46	48.29	-0.95	41.99	-33.86	-49.31	0.97	487.15	-142.68
38.35	-34.46	48.29	-0.95	44.67	-33.86	-49.31	0.97	487.88	-142.28
35.83	-37.33	52.15	-0.95	38.18	-36.74	-44.23	0.88	489.14	-142.25
38.43	-37.33	52.15	-0.95	47.52	-36.74	-44.23	0.88	489.38	-142.12
43.63	-31.29	49.83	-1.01	49.15	-17.14	-45.35	1.21	489.34	-142.19
37.18	-31.29	49.83	-1.01	54.79	-17.14	-45.35	1.21	489.97	-142.26
32.22	-34.46	48.29	-0.95	35	-27.43	-47.92	1.05	490.32	-141.99
38.35	-34.46	48.29	-0.95	54.29	-27.43	-47.92	1.05	490.27	-141.69
35.83	-37.33	52.15	-0.95	40.56	-35	-39.9	0.85	490.93	-142.14
38.43	-37.33	52.15	-0.95	37.91	-35	-39.9	0.85	491.12	-141.37
43.63	-31.29	49.83	-1.01	41.99	-33.86	-49.31	0.97	491.18	-141.22
37.18	-31.29	49.83	-1.01	44.67	-33.86	-49.31	0.97	491.23	-141.21
32.22	-34.46	48.29	-0.95	38.18	-36.74	-44.23	0.88	491.76	-141.39
38.35	-34.46	48.29	-0.95	47.52	-36.74	-44.23	0.88	491.86	-141.27
35.83	-37.33	52.15	-0.95	49.15	-17.14	-45.35	1.21	492.13	-141.26
38.43	-37.33	52.15	-0.95	54.79	-17.14	-45.35	1.21	493.74	-141.99
43.63	-31.29	49.83	-1.01	35	-27.43	-47.92	1.05	493.73	-142.01
37.18	-31.29	49.83	-1.01	54.29	-27.43	-47.92	1.05	495.6	-142.78

SPA35				SPA145				Turtle	
X	Y	Z	Rot ang	X	Y	Z	Rot ang	X	Y
32.22	-34.46	48.29	-0.95	40.56	-35	-39.9	0.85	497.41	-143.21
38.35	-34.46	48.29	-0.95	37.91	-35	-39.9	0.85	499.14	-143.95
35.83	-37.33	52.15	-0.95	41.99	-33.86	-49.31	0.97	500.02	-144.73
38.43	-37.33	52.15	-0.95	44.67	-33.86	-49.31	0.97	500.95	-145.16
43.63	-31.29	49.83	-1.01	38.18	-36.74	-44.23	0.88	501.88	-145.43
37.18	-31.29	49.83	-1.01	47.52	-36.74	-44.23	0.88	502.83	-145.97
32.22	-34.46	48.29	-0.95	49.15	-17.14	-45.35	1.21	503.74	-146.6
38.35	-34.46	48.29	-0.95	54.79	-17.14	-45.35	1.21	504.08	-146.68
35.83	-37.33	52.15	-0.95	35	-27.43	-47.92	1.05	504.57	-147.09
38.43	-37.33	52.15	-0.95	54.29	-27.43	-47.92	1.05	504.98	-147.3
43.63	-31.29	49.83	-1.01	40.56	-35	-39.9	0.85	504.83	-147.43
37.18	-31.29	49.83	-1.01	37.91	-35	-39.9	0.85	505.4	-147.84
32.22	-34.46	48.29	-0.95	41.99	-33.86	-49.31	0.97	505.64	-148.2
38.35	-34.46	48.29	-0.95	44.67	-33.86	-49.31	0.97	505.7	-148.35
35.83	-37.33	52.15	-0.95	38.18	-36.74	-44.23	0.88	505.68	-148.42
38.43	-37.33	52.15	-0.95	47.52	-36.74	-44.23	0.88	505.63	-148.54
43.63	-31.29	49.83	-1.01	49.15	-17.14	-45.35	1.21	505.63	-148.57
37.18	-31.29	49.83	-1.01	54.79	-17.14	-45.35	1.21	505.62	-148.53
32.22	-34.46	48.29	-0.95	35	-27.43	-47.92	1.05	505.67	-147.91
38.35	-34.46	48.29	-0.95	54.29	-27.43	-47.92	1.05	506.02	-147.66
35.83	-37.33	52.15	-0.95	40.56	-35	-39.9	0.85	506.36	-146.68
38.43	-37.33	52.15	-0.95	37.91	-35	-39.9	0.85	506.36	-146.14
43.63	-31.29	49.83	-1.01	41.99	-33.86	-49.31	0.97	506.35	-145.96
37.18	-31.29	49.83	-1.01	44.67	-33.86	-49.31	0.97	506.53	-145.55
32.22	-34.46	48.29	-0.95	38.18	-36.74	-44.23	0.88	506.44	-144.95
38.35	-34.46	48.29	-0.95	47.52	-36.74	-44.23	0.88	507.04	-144.9
35.83	-37.33	52.15	-0.95	49.15	-17.14	-45.35	1.21	507.27	-144.65
38.43	-37.33	52.15	-0.95	54.79	-17.14	-45.35	1.21	507.27	-144.13
43.63	-31.29	49.83	-1.01	35	-27.43	-47.92	1.05	507.8	-143.95
37.18	-31.29	49.83	-1.01	54.29	-27.43	-47.92	1.05	508.08	-143.78
32.22	-34.46	48.29	-0.95	40.56	-35	-39.9	0.85	508.36	-143.63
38.35	-34.46	48.29	-0.95	37.91	-35	-39.9	0.85	508.8	-143.82
35.83	-37.33	52.15	-0.95	41.99	-33.86	-49.31	0.97	509.67	-143.61
38.43	-37.33	52.15	-0.95	44.67	-33.86	-49.31	0.97	509.83	-143.49
43.63	-31.29	49.83	-1.01	38.18	-36.74	-44.23	0.88	509.81	-143.62
37.18	-31.29	49.83	-1.01	47.52	-36.74	-44.23	0.88	509.91	-143.26
32.22	-34.46	48.29	-0.95	49.15	-17.14	-45.35	1.21	509.93	-143.01
38.35	-34.46	48.29	-0.95	54.79	-17.14	-45.35	1.21	509.87	-142.89
35.83	-37.33	52.15	-0.95	35	-27.43	-47.92	1.05	509.83	-142.86
38.43	-37.33	52.15	-0.95	54.29	-27.43	-47.92	1.05	509.77	-142.8
43.63	-31.29	49.83	-1.01	40.56	-35	-39.9	0.85	509.7	-142.84
37.18	-31.29	49.83	-1.01	37.91	-35	-39.9	0.85	510.61	-143.41
32.22	-34.46	48.29	-0.95	41.99	-33.86	-49.31	0.97	512.38	-143.92
38.35	-34.46	48.29	-0.95	44.67	-33.86	-49.31	0.97	514.98	-144.43
35.83	-37.33	52.15	-0.95	38.18	-36.74	-44.23	0.88	516.95	-144.38
38.43	-37.33	52.15	-0.95	47.52	-36.74	-44.23	0.88	518.69	-144.92
43.63	-31.29	49.83	-1.01	49.15	-17.14	-45.35	1.21	518.71	-144.98
37.18	-31.29	49.83	-1.01	54.79	-17.14	-45.35	1.21	520.34	-145.42

SPA35				SPA145				Turtle	
X	Y	Z	Rot ang	X	Y	Z	Rot ang	X	Y
32.22	-34.46	48.29	-0.95	35	-27.43	-47.92	1.05	521.16	-145.85
38.35	-34.46	48.29	-0.95	54.29	-27.43	-47.92	1.05	521.43	-145.88
35.83	-37.33	52.15	-0.95	40.56	-35	-39.9	0.85	523.14	-146.65
38.43	-37.33	52.15	-0.95	37.91	-35	-39.9	0.85	523.97	-147.3
43.63	-31.29	49.83	-1.01	41.99	-33.86	-49.31	0.97	524.28	-147.45
37.18	-31.29	49.83	-1.01	44.67	-33.86	-49.31	0.97	525.02	-148.14
32.22	-34.46	48.29	-0.95	38.18	-36.74	-44.23	0.88	525.22	-148.21
38.35	-34.46	48.29	-0.95	47.52	-36.74	-44.23	0.88	525.37	-148.56
35.83	-37.33	52.15	-0.95	49.15	-17.14	-45.35	1.21	525.85	-148.96
38.43	-37.33	52.15	-0.95	54.79	-17.14	-45.35	1.21	526.04	-149.06
43.63	-31.29	49.83	-1.01	35	-27.43	-47.92	1.05	526.05	-149.06
37.18	-31.29	49.83	-1.01	54.29	-27.43	-47.92	1.05	526.11	-149.19
32.22	-34.46	48.29	-0.95	40.56	-35	-39.9	0.85	526.11	-149.11
38.35	-34.46	48.29	-0.95	37.91	-35	-39.9	0.85	526.03	-149.13
35.83	-37.33	52.15	-0.95	41.99	-33.86	-49.31	0.97	526.08	-149.15
38.43	-37.33	52.15	-0.95	44.67	-33.86	-49.31	0.97	526.18	-148.93
43.63	-31.29	49.83	-1.01	38.18	-36.74	-44.23	0.88	526.71	-148.37
37.18	-31.29	49.83	-1.01	47.52	-36.74	-44.23	0.88	526.94	-147.45
32.22	-34.46	48.29	-0.95	49.15	-17.14	-45.35	1.21	527.64	-147.23
38.35	-34.46	48.29	-0.95	54.79	-17.14	-45.35	1.21	527.79	-146.83
35.83	-37.33	52.15	-0.95	35	-27.43	-47.92	1.05	527.91	-146.41
38.43	-37.33	52.15	-0.95	54.29	-27.43	-47.92	1.05	528.5	-146.26
43.63	-31.29	49.83	-1.01	40.56	-35	-39.9	0.85	528.52	-146.26
37.18	-31.29	49.83	-1.01	37.91	-35	-39.9	0.85	528.66	-145.68
32.22	-34.46	48.29	-0.95	41.99	-33.86	-49.31	0.97	529.3	-145.58
38.35	-34.46	48.29	-0.95	44.67	-33.86	-49.31	0.97	529.9	-145.38
35.83	-37.33	52.15	-0.95	38.18	-36.74	-44.23	0.88	531.03	-145.14
38.43	-37.33	52.15	-0.95	47.52	-36.74	-44.23	0.88	531.18	-144.51
43.63	-31.29	49.83	-1.01	49.15	-17.14	-45.35	1.21	531.77	-144.62
37.18	-31.29	49.83	-1.01	54.79	-17.14	-45.35	1.21	532.04	-144.47
32.22	-34.46	48.29	-0.95	35	-27.43	-47.92	1.05	532.18	-144.2
38.35	-34.46	48.29	-0.95	54.29	-27.43	-47.92	1.05	532.49	-144.29
35.83	-37.33	52.15	-0.95	40.56	-35	-39.9	0.85	532.89	-144.23
38.43	-37.33	52.15	-0.95	37.91	-35	-39.9	0.85	533.05	-144.11
43.63	-31.29	49.83	-1.01	41.99	-33.86	-49.31	0.97	532.98	-144.17
37.18	-31.29	49.83	-1.01	44.67	-33.86	-49.31	0.97	533.09	-144.17
32.22	-34.46	48.29	-0.95	38.18	-36.74	-44.23	0.88	533	-144.2
38.35	-34.46	48.29	-0.95	47.52	-36.74	-44.23	0.88	532.96	-144.21
35.83	-37.33	52.15	-0.95	49.15	-17.14	-45.35	1.21	533.56	-144.47
38.43	-37.33	52.15	-0.95	54.79	-17.14	-45.35	1.21	535.01	-144.97
43.63	-31.29	49.83	-1.01	35	-27.43	-47.92	1.05	537.01	-145.66
37.18	-31.29	49.83	-1.01	54.29	-27.43	-47.92	1.05	538.81	-145.91
32.22	-34.46	48.29	-0.95	40.56	-35	-39.9	0.85	540.39	-146.26
38.35	-34.46	48.29	-0.95	37.91	-35	-39.9	0.85	540.27	-146.37
35.83	-37.33	52.15	-0.95	41.99	-33.86	-49.31	0.97	541.79	-146.89
38.43	-37.33	52.15	-0.95	44.67	-33.86	-49.31	0.97	542.88	-147.38
43.63	-31.29	49.83	-1.01	38.18	-36.74	-44.23	0.88	542.86	-147.32
37.18	-31.29	49.83	-1.01	47.52	-36.74	-44.23	0.88	543.43	-147.56

SPA35				SPA145				Turtle	
X	Y	Z	Rot ang	X	Y	Z	Rot ang	X	Y
32.22	-34.46	48.29	-0.95	49.15	-17.14	-45.35	1.21	544.11	-147.9
38.35	-34.46	48.29	-0.95	54.79	-17.14	-45.35	1.21	544.83	-148.54
35.83	-37.33	52.15	-0.95	35	-27.43	-47.92	1.05	545.48	-148.91
38.43	-37.33	52.15	-0.95	54.29	-27.43	-47.92	1.05	545.72	-149.15
43.63	-31.29	49.83	-1.01	40.56	-35	-39.9	0.85	546.27	-149.51
37.18	-31.29	49.83	-1.01	37.91	-35	-39.9	0.85	546.57	-149.82
32.22	-34.46	48.29	-0.95	41.99	-33.86	-49.31	0.97	546.67	-150
38.35	-34.46	48.29	-0.95	44.67	-33.86	-49.31	0.97	546.89	-150.32
35.83	-37.33	52.15	-0.95	38.18	-36.74	-44.23	0.88	546.74	-150.58
38.43	-37.33	52.15	-0.95	47.52	-36.74	-44.23	0.88	546.66	-150.68
43.63	-31.29	49.83	-1.01	49.15	-17.14	-45.35	1.21	546.63	-150.64
37.18	-31.29	49.83	-1.01	54.79	-17.14	-45.35	1.21	546.62	-150.62
32.22	-34.46	48.29	-0.95	35	-27.43	-47.92	1.05	546.6	-150.6
38.35	-34.46	48.29	-0.95	54.29	-27.43	-47.92	1.05	546.74	-149.96
35.83	-37.33	52.15	-0.95	40.56	-35	-39.9	0.85	547.05	-149.68
38.43	-37.33	52.15	-0.95	37.91	-35	-39.9	0.85	547.37	-148.8
43.63	-31.29	49.83	-1.01	41.99	-33.86	-49.31	0.97	547.56	-148.19
37.18	-31.29	49.83	-1.01	44.67	-33.86	-49.31	0.97	548.14	-147.86
32.22	-34.46	48.29	-0.95	38.18	-36.74	-44.23	0.88	548.79	-147.66
38.35	-34.46	48.29	-0.95	47.52	-36.74	-44.23	0.88	549.68	-147.2
35.83	-37.33	52.15	-0.95	49.15	-17.14	-45.35	1.21	550.3	-146.7
38.43	-37.33	52.15	-0.95	54.79	-17.14	-45.35	1.21	550.89	-146.4
43.63	-31.29	49.83	-1.01	35	-27.43	-47.92	1.05	550.84	-146.61
37.18	-31.29	49.83	-1.01	54.29	-27.43	-47.92	1.05	551.46	-146.64
32.22	-34.46	48.29	-0.95	40.56	-35	-39.9	0.85	551.74	-146.25
38.35	-34.46	48.29	-0.95	37.91	-35	-39.9	0.85	552.28	-146.15
35.83	-37.33	52.15	-0.95	41.99	-33.86	-49.31	0.97	552.59	-145.93
38.43	-37.33	52.15	-0.95	44.67	-33.86	-49.31	0.97	552.85	-145.91
43.63	-31.29	49.83	-1.01	38.18	-36.74	-44.23	0.88	553.22	-145.88
37.18	-31.29	49.83	-1.01	47.52	-36.74	-44.23	0.88	553.58	-145.68
32.22	-34.46	48.29	-0.95	49.15	-17.14	-45.35	1.21	553.78	-145.77
38.35	-34.46	48.29	-0.95	54.79	-17.14	-45.35	1.21	553.97	-145.74
35.83	-37.33	52.15	-0.95	35	-27.43	-47.92	1.05	554.26	-145.67
38.43	-37.33	52.15	-0.95	54.29	-27.43	-47.92	1.05	554.34	-145.67
43.63	-31.29	49.83	-1.01	40.56	-35	-39.9	0.85	554.32	-145.68
37.18	-31.29	49.83	-1.01	37.91	-35	-39.9	0.85	554.31	-145.71
32.22	-34.46	48.29	-0.95	41.99	-33.86	-49.31	0.97	555.05	-145.85
38.35	-34.46	48.29	-0.95	44.67	-33.86	-49.31	0.97	556.86	-146.44
35.83	-37.33	52.15	-0.95	38.18	-36.74	-44.23	0.88	560.81	-147.23
38.43	-37.33	52.15	-0.95	47.52	-36.74	-44.23	0.88	562.45	-147.64
43.63	-31.29	49.83	-1.01	49.15	-17.14	-45.35	1.21	564.05	-147.89
37.18	-31.29	49.83	-1.01	54.79	-17.14	-45.35	1.21	565.04	-148.53
32.22	-34.46	48.29	-0.95	35	-27.43	-47.92	1.05	565.65	-148.6
38.35	-34.46	48.29	-0.95	54.29	-27.43	-47.92	1.05	566.2	-148.79
35.83	-37.33	52.15	-0.95	40.56	-35	-39.9	0.85	566.91	-149.28
38.43	-37.33	52.15	-0.95	37.91	-35	-39.9	0.85	567.67	-149.66
43.63	-31.29	49.83	-1.01	41.99	-33.86	-49.31	0.97	567.73	-149.56
37.18	-31.29	49.83	-1.01	44.67	-33.86	-49.31	0.97	567.99	-150.06

SPA35				SPA145				Turtle	
X	Y	Z	Rot ang	X	Y	Z	Rot ang	X	Y
32.22	-34.46	48.29	-0.95	38.18	-36.74	-44.23	0.88	568.7	-150.26
38.35	-34.46	48.29	-0.95	47.52	-36.74	-44.23	0.88	568.83	-150.64
35.83	-37.33	52.15	-0.95	49.15	-17.14	-45.35	1.21	569.03	-150.93
38.43	-37.33	52.15	-0.95	54.79	-17.14	-45.35	1.21	569.39	-151.26
43.63	-31.29	49.83	-1.01	35	-27.43	-47.92	1.05	569.62	-151.56
37.18	-31.29	49.83	-1.01	54.29	-27.43	-47.92	1.05	569.75	-151.87
32.22	-34.46	48.29	-0.95	40.56	-35	-39.9	0.85	569.75	-151.94
38.35	-34.46	48.29	-0.95	37.91	-35	-39.9	0.85	569.75	-152
35.83	-37.33	52.15	-0.95	41.99	-33.86	-49.31	0.97	569.74	-152
38.43	-37.33	52.15	-0.95	44.67	-33.86	-49.31	0.97	569.86	-151.77
43.63	-31.29	49.83	-1.01	38.18	-36.74	-44.23	0.88	569.81	-151.86
37.18	-31.29	49.83	-1.01	47.52	-36.74	-44.23	0.88	570.48	-151.22
32.22	-34.46	48.29	-0.95	49.15	-17.14	-45.35	1.21	571.17	-150.54
38.35	-34.46	48.29	-0.95	54.79	-17.14	-45.35	1.21	571.65	-150.08
35.83	-37.33	52.15	-0.95	35	-27.43	-47.92	1.05	572.41	-149.27
38.43	-37.33	52.15	-0.95	54.29	-27.43	-47.92	1.05	572.98	-148.91
43.63	-31.29	49.83	-1.01	40.56	-35	-39.9	0.85	573.16	-148.89
37.18	-31.29	49.83	-1.01	37.91	-35	-39.9	0.85	573.86	-148.49
32.22	-34.46	48.29	-0.95	41.99	-33.86	-49.31	0.97	574.2	-148.14
38.35	-34.46	48.29	-0.95	44.67	-33.86	-49.31	0.97	574.84	-147.88
35.83	-37.33	52.15	-0.95	38.18	-36.74	-44.23	0.88	575.05	-147.99
38.43	-37.33	52.15	-0.95	47.52	-36.74	-44.23	0.88	575.59	-147.73
43.63	-31.29	49.83	-1.01	49.15	-17.14	-45.35	1.21	575.64	-147.88
37.18	-31.29	49.83	-1.01	54.79	-17.14	-45.35	1.21	575.88	-147.96
32.22	-34.46	48.29	-0.95	35	-27.43	-47.92	1.05	576.26	-147.77
38.35	-34.46	48.29	-0.95	54.29	-27.43	-47.92	1.05	576.58	-147.9
35.83	-37.33	52.15	-0.95	40.56	-35	-39.9	0.85	577.19	-147.62
38.43	-37.33	52.15	-0.95	37.91	-35	-39.9	0.85	577.31	-147.74
43.63	-31.29	49.83	-1.01	41.99	-33.86	-49.31	0.97	577.49	-147.85
37.18	-31.29	49.83	-1.01	44.67	-33.86	-49.31	0.97	577.57	-147.96
32.22	-34.46	48.29	-0.95	38.18	-36.74	-44.23	0.88	577.59	-147.9
38.35	-34.46	48.29	-0.95	47.52	-36.74	-44.23	0.88	577.61	-147.94
35.83	-37.33	52.15	-0.95	49.15	-17.14	-45.35	1.21	578.37	-147.92
38.43	-37.33	52.15	-0.95	54.79	-17.14	-45.35	1.21	579.86	-148.54
43.63	-31.29	49.83	-1.01	35	-27.43	-47.92	1.05	580	-148.59
37.18	-31.29	49.83	-1.01	54.29	-27.43	-47.92	1.05	581.92	-149.03
32.22	-34.46	48.29	-0.95	40.56	-35	-39.9	0.85	583.84	-149.41
38.35	-34.46	48.29	-0.95	37.91	-35	-39.9	0.85	585.6	-149.6
35.83	-37.33	52.15	-0.95	41.99	-33.86	-49.31	0.97	587.19	-150.03
38.43	-37.33	52.15	-0.95	44.67	-33.86	-49.31	0.97	588.17	-150.21
43.63	-31.29	49.83	-1.01	38.18	-36.74	-44.23	0.88	588.62	-150.99
37.18	-31.29	49.83	-1.01	47.52	-36.74	-44.23	0.88	589.15	-150.85
32.22	-34.46	48.29	-0.95	49.15	-17.14	-45.35	1.21	589.94	-151.04
38.35	-34.46	48.29	-0.95	54.79	-17.14	-45.35	1.21	590.37	-151.74
35.83	-37.33	52.15	-0.95	35	-27.43	-47.92	1.05	591.53	-152.04
38.43	-37.33	52.15	-0.95	54.29	-27.43	-47.92	1.05	592	-152.6
43.63	-31.29	49.83	-1.01	40.56	-35	-39.9	0.85	591.98	-152.54
37.18	-31.29	49.83	-1.01	37.91	-35	-39.9	0.85	592.19	-153.27

SPA35				SPA145				Turtle	
X	Y	Z	Rot ang	X	Y	Z	Rot ang	X	Y
32.22	-34.46	48.29	-0.95	41.99	-33.86	-49.31	0.97	592.4	-153.54
38.35	-34.46	48.29	-0.95	44.67	-33.86	-49.31	0.97	592.36	-153.71
35.83	-37.33	52.15	-0.95	38.18	-36.74	-44.23	0.88	592.19	-154.11
38.43	-37.33	52.15	-0.95	47.52	-36.74	-44.23	0.88	592.12	-154.19
43.63	-31.29	49.83	-1.01	49.15	-17.14	-45.35	1.21	592.08	-154.19
37.18	-31.29	49.83	-1.01	54.79	-17.14	-45.35	1.21	592.09	-154.29
32.22	-34.46	48.29	-0.95	35	-27.43	-47.92	1.05	592.07	-154.26
38.35	-34.46	48.29	-0.95	54.29	-27.43	-47.92	1.05	592.15	-154.11
35.83	-37.33	52.15	-0.95	40.56	-35	-39.9	0.85	593.54	-152.42
38.43	-37.33	52.15	-0.95	37.91	-35	-39.9	0.85	593.89	-152.32
43.63	-31.29	49.83	-1.01	41.99	-33.86	-49.31	0.97	593.82	-152.33
37.18	-31.29	49.83	-1.01	44.67	-33.86	-49.31	0.97	594.53	-151.64
32.22	-34.46	48.29	-0.95	38.18	-36.74	-44.23	0.88	594.99	-151.49
38.35	-34.46	48.29	-0.95	47.52	-36.74	-44.23	0.88	595.43	-151.1
35.83	-37.33	52.15	-0.95	49.15	-17.14	-45.35	1.21	595.66	-151.2
38.43	-37.33	52.15	-0.95	54.79	-17.14	-45.35	1.21	596.34	-150.74
43.63	-31.29	49.83	-1.01	35	-27.43	-47.92	1.05	597.12	-150.23
37.18	-31.29	49.83	-1.01	54.29	-27.43	-47.92	1.05	597.71	-150.05
32.22	-34.46	48.29	-0.95	40.56	-35	-39.9	0.85	598.06	-149.99
38.35	-34.46	48.29	-0.95	37.91	-35	-39.9	0.85	598.81	-149.64
35.83	-37.33	52.15	-0.95	41.99	-33.86	-49.31	0.97	599.6	-149.37
38.43	-37.33	52.15	-0.95	44.67	-33.86	-49.31	0.97	599.79	-149.33
43.63	-31.29	49.83	-1.01	38.18	-36.74	-44.23	0.88	599.78	-149.34
37.18	-31.29	49.83	-1.01	47.52	-36.74	-44.23	0.88	599.87	-149.37
32.22	-34.46	48.29	-0.95	49.15	-17.14	-45.35	1.21	600.07	-149.41
38.35	-34.46	48.29	-0.95	54.79	-17.14	-45.35	1.21	600.41	-149.27
35.83	-37.33	52.15	-0.95	35	-27.43	-47.92	1.05	600.65	-149.34
38.43	-37.33	52.15	-0.95	54.29	-27.43	-47.92	1.05	600.75	-149.32
43.63	-31.29	49.83	-1.01	40.56	-35	-39.9	0.85	600.84	-149.35
37.18	-31.29	49.83	-1.01	37.91	-35	-39.9	0.85	601.55	-149.31
32.22	-34.46	48.29	-0.95	41.99	-33.86	-49.31	0.97	603.38	-149.96
38.35	-34.46	48.29	-0.95	44.67	-33.86	-49.31	0.97	605.9	-150.05
35.83	-37.33	52.15	-0.95	38.18	-36.74	-44.23	0.88	607.65	-150.65
38.43	-37.33	52.15	-0.95	47.52	-36.74	-44.23	0.88	608.89	-151.39
43.63	-31.29	49.83	-1.01	49.15	-17.14	-45.35	1.21	608.88	-151.44
37.18	-31.29	49.83	-1.01	54.79	-17.14	-45.35	1.21	610.36	-151.53
32.22	-34.46	48.29	-0.95	35	-27.43	-47.92	1.05	610.77	-152.22
38.35	-34.46	48.29	-0.95	54.29	-27.43	-47.92	1.05	611.39	-152.28
35.83	-37.33	52.15	-0.95	40.56	-35	-39.9	0.85	611.68	-152.88
38.43	-37.33	52.15	-0.95	37.91	-35	-39.9	0.85	612.46	-153.12
43.63	-31.29	49.83	-1.01	41.99	-33.86	-49.31	0.97	613.32	-153.33
37.18	-31.29	49.83	-1.01	44.67	-33.86	-49.31	0.97	614.13	-154
32.22	-34.46	48.29	-0.95	38.18	-36.74	-44.23	0.88	614.18	-153.95
38.35	-34.46	48.29	-0.95	47.52	-36.74	-44.23	0.88	614.3	-154.65
35.83	-37.33	52.15	-0.95	49.15	-17.14	-45.35	1.21	615.06	-154.88
38.43	-37.33	52.15	-0.95	54.79	-17.14	-45.35	1.21	615.18	-155.11
43.63	-31.29	49.83	-1.01	35	-27.43	-47.92	1.05	615.18	-154.97
37.18	-31.29	49.83	-1.01	54.29	-27.43	-47.92	1.05	615.19	-155.53

SPA35				SPA145				Turtle	
X	Y	Z	Rot ang	X	Y	Z	Rot ang	X	Y
32.22	-34.46	48.29	-0.95	40.56	-35	-39.9	0.85	615.22	-155.57
38.35	-34.46	48.29	-0.95	37.91	-35	-39.9	0.85	615.24	-155.67
35.83	-37.33	52.15	-0.95	41.99	-33.86	-49.31	0.97	615.23	-155.64
38.43	-37.33	52.15	-0.95	44.67	-33.86	-49.31	0.97	615.23	-155.62
43.63	-31.29	49.83	-1.01	38.18	-36.74	-44.23	0.88	615.29	-155.4
37.18	-31.29	49.83	-1.01	47.52	-36.74	-44.23	0.88	616.7	-153.8
32.22	-34.46	48.29	-0.95	49.15	-17.14	-45.35	1.21	617.01	-153.61
38.35	-34.46	48.29	-0.95	54.79	-17.14	-45.35	1.21	617.75	-152.99
35.83	-37.33	52.15	-0.95	35	-27.43	-47.92	1.05	618.36	-152.57
38.43	-37.33	52.15	-0.95	54.29	-27.43	-47.92	1.05	618.78	-152.54
43.63	-31.29	49.83	-1.01	40.56	-35	-39.9	0.85	618.72	-152.61
37.18	-31.29	49.83	-1.01	37.91	-35	-39.9	0.85	619.45	-152.08
32.22	-34.46	48.29	-0.95	41.99	-33.86	-49.31	0.97	619.85	-152
38.35	-34.46	48.29	-0.95	44.67	-33.86	-49.31	0.97	620.43	-151.66
35.83	-37.33	52.15	-0.95	38.18	-36.74	-44.23	0.88	621.27	-151.15
38.43	-37.33	52.15	-0.95	47.52	-36.74	-44.23	0.88	621.62	-151.09
43.63	-31.29	49.83	-1.01	49.15	-17.14	-45.35	1.21	622.02	-150.73
37.18	-31.29	49.83	-1.01	54.79	-17.14	-45.35	1.21	622.86	-150.56
32.22	-34.46	48.29	-0.95	35	-27.43	-47.92	1.05	623.04	-150.64
38.35	-34.46	48.29	-0.95	54.29	-27.43	-47.92	1.05	623.28	-150.62
35.83	-37.33	52.15	-0.95	40.56	-35	-39.9	0.85	623.72	-150.32
38.43	-37.33	52.15	-0.95	37.91	-35	-39.9	0.85	623.88	-150.52
43.63	-31.29	49.83	-1.01	41.99	-33.86	-49.31	0.97	623.87	-150.58
37.18	-31.29	49.83	-1.01	44.67	-33.86	-49.31	0.97	624.01	-150.55
32.22	-34.46	48.29	-0.95	38.18	-36.74	-44.23	0.88	624.14	-150.57
38.35	-34.46	48.29	-0.95	47.52	-36.74	-44.23	0.88	624.46	-150.3
35.83	-37.33	52.15	-0.95	49.15	-17.14	-45.35	1.21	624.63	-150.45
38.43	-37.33	52.15	-0.95	54.79	-17.14	-45.35	1.21	625.04	-150.67
43.63	-31.29	49.83	-1.01	35	-27.43	-47.92	1.05	626.52	-151.24
37.18	-31.29	49.83	-1.01	54.29	-27.43	-47.92	1.05	628.31	-152
32.22	-34.46	48.29	-0.95	40.56	-35	-39.9	0.85	630.21	-152.24
38.35	-34.46	48.29	-0.95	37.91	-35	-39.9	0.85	631.25	-152.88
35.83	-37.33	52.15	-0.95	41.99	-33.86	-49.31	0.97	633.46	-153.6
38.43	-37.33	52.15	-0.95	44.67	-33.86	-49.31	0.97	633.81	-153.81
43.63	-31.29	49.83	-1.01	38.18	-36.74	-44.23	0.88	633.79	-153.75
37.18	-31.29	49.83	-1.01	47.52	-36.74	-44.23	0.88	633.92	-154.4
32.22	-34.46	48.29	-0.95	49.15	-17.14	-45.35	1.21	634.79	-154.6
38.35	-34.46	48.29	-0.95	54.79	-17.14	-45.35	1.21	635.37	-155.01
35.83	-37.33	52.15	-0.95	35	-27.43	-47.92	1.05	635.92	-155.48
38.43	-37.33	52.15	-0.95	54.29	-27.43	-47.92	1.05	636.49	-155.4
43.63	-31.29	49.83	-1.01	40.56	-35	-39.9	0.85	636.56	-156.05
37.18	-31.29	49.83	-1.01	37.91	-35	-39.9	0.85	636.81	-156.54
32.22	-34.46	48.29	-0.95	41.99	-33.86	-49.31	0.97	636.74	-156.98
38.35	-34.46	48.29	-0.95	44.67	-33.86	-49.31	0.97	636.86	-157.1
35.83	-37.33	52.15	-0.95	38.18	-36.74	-44.23	0.88	637.35	-157
38.43	-37.33	52.15	-0.95	47.52	-36.74	-44.23	0.88	637.4	-157.02
43.63	-31.29	49.83	-1.01	49.15	-17.14	-45.35	1.21	637.44	-157.03
37.18	-31.29	49.83	-1.01	54.79	-17.14	-45.35	1.21	637.47	-157.04

SPA35				SPA145				Turtle	
X	Y	Z	Rot ang	X	Y	Z	Rot ang	X	Y
32.22	-34.46	48.29	-0.95	35	-27.43	-47.92	1.05	637.54	-157.14
38.35	-34.46	48.29	-0.95	54.29	-27.43	-47.92	1.05	637.56	-157
35.83	-37.33	52.15	-0.95	40.56	-35	-39.9	0.85	638.27	-156.11
38.43	-37.33	52.15	-0.95	37.91	-35	-39.9	0.85	639.04	-155.18
43.63	-31.29	49.83	-1.01	41.99	-33.86	-49.31	0.97	639.19	-155.05
37.18	-31.29	49.83	-1.01	44.67	-33.86	-49.31	0.97	639.81	-154.28
32.22	-34.46	48.29	-0.95	38.18	-36.74	-44.23	0.88	640.08	-154.2
38.35	-34.46	48.29	-0.95	47.52	-36.74	-44.23	0.88	640.84	-153.39
35.83	-37.33	52.15	-0.95	49.15	-17.14	-45.35	1.21	641.13	-153.39
38.43	-37.33	52.15	-0.95	54.79	-17.14	-45.35	1.21	642.44	-152.36
43.63	-31.29	49.83	-1.01	35	-27.43	-47.92	1.05	642.49	-152.33
37.18	-31.29	49.83	-1.01	54.29	-27.43	-47.92	1.05	643.18	-152.12
32.22	-34.46	48.29	-0.95	40.56	-35	-39.9	0.85	643.53	-152.19
38.35	-34.46	48.29	-0.95	37.91	-35	-39.9	0.85	644.17	-152.06
35.83	-37.33	52.15	-0.95	41.99	-33.86	-49.31	0.97	644.53	-152.11
38.43	-37.33	52.15	-0.95	44.67	-33.86	-49.31	0.97	645.25	-151.66
43.63	-31.29	49.83	-1.01	38.18	-36.74	-44.23	0.88	645.22	-151.95
37.18	-31.29	49.83	-1.01	47.52	-36.74	-44.23	0.88	646.15	-151.38
32.22	-34.46	48.29	-0.95	49.15	-17.14	-45.35	1.21	646.35	-151.46
38.35	-34.46	48.29	-0.95	54.79	-17.14	-45.35	1.21	646.74	-151.14
35.83	-37.33	52.15	-0.95	35	-27.43	-47.92	1.05	646.94	-151.15
38.43	-37.33	52.15	-0.95	54.29	-27.43	-47.92	1.05	647.08	-151.21
43.63	-31.29	49.83	-1.01	40.56	-35	-39.9	0.85	647.03	-151.18
37.18	-31.29	49.83	-1.01	37.91	-35	-39.9	0.85	647.01	-151.18
32.22	-34.46	48.29	-0.95	41.99	-33.86	-49.31	0.97	647.74	-151.15
38.35	-34.46	48.29	-0.95	44.67	-33.86	-49.31	0.97	648.83	-151.94
35.83	-37.33	52.15	-0.95	38.18	-36.74	-44.23	0.88	650.71	-152.7
38.43	-37.33	52.15	-0.95	47.52	-36.74	-44.23	0.88	652.54	-152.83
43.63	-31.29	49.83	-1.01	49.15	-17.14	-45.35	1.21	654.03	-153.34
37.18	-31.29	49.83	-1.01	54.79	-17.14	-45.35	1.21	655	-154.04
32.22	-34.46	48.29	-0.95	35	-27.43	-47.92	1.05	656	-154.93
38.35	-34.46	48.29	-0.95	54.29	-27.43	-47.92	1.05	656.67	-154.92
35.83	-37.33	52.15	-0.95	40.56	-35	-39.9	0.85	656.96	-155.7
38.43	-37.33	52.15	-0.95	37.91	-35	-39.9	0.85	657.88	-155.91
43.63	-31.29	49.83	-1.01	41.99	-33.86	-49.31	0.97	657.87	-155.89
37.18	-31.29	49.83	-1.01	44.67	-33.86	-49.31	0.97	657.99	-156.61
32.22	-34.46	48.29	-0.95	38.18	-36.74	-44.23	0.88	658.68	-156.67
38.35	-34.46	48.29	-0.95	47.52	-36.74	-44.23	0.88	658.74	-157.21
35.83	-37.33	52.15	-0.95	49.15	-17.14	-45.35	1.21	658.95	-157.61
38.43	-37.33	52.15	-0.95	54.79	-17.14	-45.35	1.21	659.26	-158.5
43.63	-31.29	49.83	-1.01	35	-27.43	-47.92	1.05	659.3	-158.67
37.18	-31.29	49.83	-1.01	54.29	-27.43	-47.92	1.05	659.35	-158.72
32.22	-34.46	48.29	-0.95	40.56	-35	-39.9	0.85	659.13	-158.61
38.35	-34.46	48.29	-0.95	37.91	-35	-39.9	0.85	659.11	-158.8
35.83	-37.33	52.15	-0.95	41.99	-33.86	-49.31	0.97	659.04	-158.66
38.43	-37.33	52.15	-0.95	44.67	-33.86	-49.31	0.97	659.1	-158.45
43.63	-31.29	49.83	-1.01	38.18	-36.74	-44.23	0.88	659.04	-158.45
37.18	-31.29	49.83	-1.01	47.52	-36.74	-44.23	0.88	659.77	-157.53

SPA35				SPA145				Turtle	
X	Y	Z	Rot ang	X	Y	Z	Rot ang	X	Y
32.22	-34.46	48.29	-0.95	49.15	-17.14	-45.35	1.21	660.54	-156.65
38.35	-34.46	48.29	-0.95	54.79	-17.14	-45.35	1.21	660.65	-156.48
35.83	-37.33	52.15	-0.95	35	-27.43	-47.92	1.05	661.38	-155.74
38.43	-37.33	52.15	-0.95	54.29	-27.43	-47.92	1.05	662.28	-154.93
43.63	-31.29	49.83	-1.01	40.56	-35	-39.9	0.85	662.71	-154.63
37.18	-31.29	49.83	-1.01	37.91	-35	-39.9	0.85	663.2	-154.65
32.22	-34.46	48.29	-0.95	41.99	-33.86	-49.31	0.97	663.18	-154.65
38.35	-34.46	48.29	-0.95	44.67	-33.86	-49.31	0.97	663.85	-154.37
35.83	-37.33	52.15	-0.95	38.18	-36.74	-44.23	0.88	664.82	-153.83
38.43	-37.33	52.15	-0.95	47.52	-36.74	-44.23	0.88	665.14	-153.86
43.63	-31.29	49.83	-1.01	49.15	-17.14	-45.35	1.21	665.7	-153.59
37.18	-31.29	49.83	-1.01	54.79	-17.14	-45.35	1.21	666.1	-153.73
32.22	-34.46	48.29	-0.95	35	-27.43	-47.92	1.05	666.51	-153.52
38.35	-34.46	48.29	-0.95	54.29	-27.43	-47.92	1.05	666.74	-153.54
35.83	-37.33	52.15	-0.95	40.56	-35	-39.9	0.85	667.23	-153.3
38.43	-37.33	52.15	-0.95	37.91	-35	-39.9	0.85	667.52	-153.42
43.63	-31.29	49.83	-1.01	41.99	-33.86	-49.31	0.97	667.67	-153.5
37.18	-31.29	49.83	-1.01	44.67	-33.86	-49.31	0.97	667.92	-153.15
32.22	-34.46	48.29	-0.95	38.18	-36.74	-44.23	0.88	668.32	-153.37
38.35	-34.46	48.29	-0.95	47.52	-36.74	-44.23	0.88	668.41	-153.41
35.83	-37.33	52.15	-0.95	49.15	-17.14	-45.35	1.21	669.07	-153.34
38.43	-37.33	52.15	-0.95	54.79	-17.14	-45.35	1.21	670.28	-154.22
43.63	-31.29	49.83	-1.01	35	-27.43	-47.92	1.05	670.25	-154.24
37.18	-31.29	49.83	-1.01	54.29	-27.43	-47.92	1.05	672.02	-154.85
32.22	-34.46	48.29	-0.95	40.56	-35	-39.9	0.85	673.69	-155
38.35	-34.46	48.29	-0.95	37.91	-35	-39.9	0.85	674.81	-155.83
35.83	-37.33	52.15	-0.95	41.99	-33.86	-49.31	0.97	675.62	-156.43
38.43	-37.33	52.15	-0.95	44.67	-33.86	-49.31	0.97	676.56	-157.14
43.63	-31.29	49.83	-1.01	38.18	-36.74	-44.23	0.88	676.58	-157.3
37.18	-31.29	49.83	-1.01	47.52	-36.74	-44.23	0.88	677.06	-157.35
32.22	-34.46	48.29	-0.95	49.15	-17.14	-45.35	1.21	677.54	-157.99
38.35	-34.46	48.29	-0.95	54.79	-17.14	-45.35	1.21	678.51	-159.06
35.83	-37.33	52.15	-0.95	35	-27.43	-47.92	1.05	678.7	-159.23
38.43	-37.33	52.15	-0.95	54.29	-27.43	-47.92	1.05	679.25	-159.27
43.63	-31.29	49.83	-1.01	40.56	-35	-39.9	0.85	679.29	-159.21
37.18	-31.29	49.83	-1.01	37.91	-35	-39.9	0.85	679.33	-159.86
32.22	-34.46	48.29	-0.95	41.99	-33.86	-49.31	0.97	679.4	-160.03
38.35	-34.46	48.29	-0.95	44.67	-33.86	-49.31	0.97	679.54	-160.2
35.83	-37.33	52.15	-0.95	38.18	-36.74	-44.23	0.88	679.48	-160.72
38.43	-37.33	52.15	-0.95	47.52	-36.74	-44.23	0.88	679.49	-160.87
43.63	-31.29	49.83	-1.01	49.15	-17.14	-45.35	1.21	679.44	-160.88
37.18	-31.29	49.83	-1.01	54.79	-17.14	-45.35	1.21	679.47	-160.9
32.22	-34.46	48.29	-0.95	35	-27.43	-47.92	1.05	679.64	-160.75
38.35	-34.46	48.29	-0.95	54.29	-27.43	-47.92	1.05	680.29	-159.97
35.83	-37.33	52.15	-0.95	40.56	-35	-39.9	0.85	680.92	-159.05
38.43	-37.33	52.15	-0.95	37.91	-35	-39.9	0.85	681.02	-158.73
43.63	-31.29	49.83	-1.01	41.99	-33.86	-49.31	0.97	681.07	-158.86
37.18	-31.29	49.83	-1.01	44.67	-33.86	-49.31	0.97	681.06	-158.36

SPA35				SPA145				Turtle	
X	Y	Z	Rot ang	X	Y	Z	Rot ang	X	Y
32.22	-34.46	48.29	-0.95	38.18	-36.74	-44.23	0.88	680.67	-157.86
38.35	-34.46	48.29	-0.95	47.52	-36.74	-44.23	0.88	680.93	-157.39
35.83	-37.33	52.15	-0.95	49.15	-17.14	-45.35	1.21	681.01	-157.34
38.43	-37.33	52.15	-0.95	54.79	-17.14	-45.35	1.21	681.88	-156.35
43.63	-31.29	49.83	-1.01	35	-27.43	-47.92	1.05	682.2	-156.4
37.18	-31.29	49.83	-1.01	54.29	-27.43	-47.92	1.05	682.82	-156.13
32.22	-34.46	48.29	-0.95	40.56	-35	-39.9	0.85	683.59	-155.59
38.35	-34.46	48.29	-0.95	37.91	-35	-39.9	0.85	683.78	-155.9
35.83	-37.33	52.15	-0.95	41.99	-33.86	-49.31	0.97	684.45	-155.44
38.43	-37.33	52.15	-0.95	44.67	-33.86	-49.31	0.97	684.6	-155.52
43.63	-31.29	49.83	-1.01	38.18	-36.74	-44.23	0.88	684.55	-155.41
37.18	-31.29	49.83	-1.01	47.52	-36.74	-44.23	0.88	684.7	-155.43
32.22	-34.46	48.29	-0.95	49.15	-17.14	-45.35	1.21	684.65	-155.29
38.35	-34.46	48.29	-0.95	54.79	-17.14	-45.35	1.21	684.95	-155.13
35.83	-37.33	52.15	-0.95	35	-27.43	-47.92	1.05	685.14	-155.16
38.43	-37.33	52.15	-0.95	54.29	-27.43	-47.92	1.05	685.1	-155.02
43.63	-31.29	49.83	-1.01	40.56	-35	-39.9	0.85	684.61	-155.25
37.18	-31.29	49.83	-1.01	37.91	-35	-39.9	0.85	685.16	-155.13
32.22	-34.46	48.29	-0.95	41.99	-33.86	-49.31	0.97	686.31	-155.89
38.35	-34.46	48.29	-0.95	44.67	-33.86	-49.31	0.97	688.11	-156.19
35.83	-37.33	52.15	-0.95	38.18	-36.74	-44.23	0.88	689.75	-156.66
38.43	-37.33	52.15	-0.95	47.52	-36.74	-44.23	0.88	690.84	-157.51
43.63	-31.29	49.83	-1.01	49.15	-17.14	-45.35	1.21	690.83	-157.53
37.18	-31.29	49.83	-1.01	54.79	-17.14	-45.35	1.21	691.68	-157.99
32.22	-34.46	48.29	-0.95	35	-27.43	-47.92	1.05	692.48	-158.36
38.35	-34.46	48.29	-0.95	54.29	-27.43	-47.92	1.05	692.72	-158.55
35.83	-37.33	52.15	-0.95	40.56	-35	-39.9	0.85	693.54	-159.19
38.43	-37.33	52.15	-0.95	37.91	-35	-39.9	0.85	694.37	-159.28
43.63	-31.29	49.83	-1.01	41.99	-33.86	-49.31	0.97	694.56	-160.04
37.18	-31.29	49.83	-1.01	44.67	-33.86	-49.31	0.97	694.67	-160.71
32.22	-34.46	48.29	-0.95	38.18	-36.74	-44.23	0.88	694.98	-160.49
38.35	-34.46	48.29	-0.95	47.52	-36.74	-44.23	0.88	695.44	-160.99
35.83	-37.33	52.15	-0.95	49.15	-17.14	-45.35	1.21	695.44	-160.95
38.43	-37.33	52.15	-0.95	54.79	-17.14	-45.35	1.21	695.43	-161.27
43.63	-31.29	49.83	-1.01	35	-27.43	-47.92	1.05	695.54	-161.78
37.18	-31.29	49.83	-1.01	54.29	-27.43	-47.92	1.05	695.6	-161.9
32.22	-34.46	48.29	-0.95	40.56	-35	-39.9	0.85	695.62	-161.96
38.35	-34.46	48.29	-0.95	37.91	-35	-39.9	0.85	695.64	-162.02
35.83	-37.33	52.15	-0.95	41.99	-33.86	-49.31	0.97	695.64	-162.03
38.43	-37.33	52.15	-0.95	44.67	-33.86	-49.31	0.97	695.58	-161.91
43.63	-31.29	49.83	-1.01	38.18	-36.74	-44.23	0.88	695.69	-161.76
37.18	-31.29	49.83	-1.01	47.52	-36.74	-44.23	0.88	696.82	-159.95
32.22	-34.46	48.29	-0.95	49.15	-17.14	-45.35	1.21	697.12	-159.29
38.35	-34.46	48.29	-0.95	54.79	-17.14	-45.35	1.21	696.52	-159.17
35.83	-37.33	52.15	-0.95	35	-27.43	-47.92	1.05	696.3	-158.94
38.43	-37.33	52.15	-0.95	54.29	-27.43	-47.92	1.05	696.27	-158.34
43.63	-31.29	49.83	-1.01	40.56	-35	-39.9	0.85	696.24	-158.27
37.18	-31.29	49.83	-1.01	37.91	-35	-39.9	0.85	696.39	-158.24

SPA35				SPA145				Turtle	
X	Y	Z	Rot ang	X	Y	Z	Rot ang	X	Y
32.22	-34.46	48.29	-0.95	41.99	-33.86	-49.31	0.97	696.95	-157.75
38.35	-34.46	48.29	-0.95	44.67	-33.86	-49.31	0.97	697.59	-157.28
35.83	-37.33	52.15	-0.95	38.18	-36.74	-44.23	0.88	697.89	-157.19
38.43	-37.33	52.15	-0.95	47.52	-36.74	-44.23	0.88	698.04	-157.23
43.63	-31.29	49.83	-1.01	49.15	-17.14	-45.35	1.21	698.63	-156.56
37.18	-31.29	49.83	-1.01	54.79	-17.14	-45.35	1.21	698.41	-156.25
32.22	-34.46	48.29	-0.95	35	-27.43	-47.92	1.05	698.15	-156.46
38.35	-34.46	48.29	-0.95	54.29	-27.43	-47.92	1.05	697.75	-156.2
35.83	-37.33	52.15	-0.95	40.56	-35	-39.9	0.85	697.11	-156.28
38.43	-37.33	52.15	-0.95	37.91	-35	-39.9	0.85	696.94	-155.65
43.63	-31.29	49.83	-1.01	41.99	-33.86	-49.31	0.97	696.93	-155.57
37.18	-31.29	49.83	-1.01	44.67	-33.86	-49.31	0.97	696.41	-155.6
32.22	-34.46	48.29	-0.95	38.18	-36.74	-44.23	0.88	695.98	-155.48
38.35	-34.46	48.29	-0.95	47.52	-36.74	-44.23	0.88	695.8	-155.43
35.83	-37.33	52.15	-0.95	49.15	-17.14	-45.35	1.21	695.49	-155.59
38.43	-37.33	52.15	-0.95	54.79	-17.14	-45.35	1.21	696.05	-155.59
43.63	-31.29	49.83	-1.01	35	-27.43	-47.92	1.05	697.25	-156.36
37.18	-31.29	49.83	-1.01	54.29	-27.43	-47.92	1.05	700.92	-157.35
32.22	-34.46	48.29	-0.95	40.56	-35	-39.9	0.85	701.92	-157.91
38.35	-34.46	48.29	-0.95	37.91	-35	-39.9	0.85	703.2	-157.95
35.83	-37.33	52.15	-0.95	41.99	-33.86	-49.31	0.97	703.61	-158.73
38.43	-37.33	52.15	-0.95	44.67	-33.86	-49.31	0.97	704.28	-158.73
43.63	-31.29	49.83	-1.01	38.18	-36.74	-44.23	0.88	704.27	-158.71
37.18	-31.29	49.83	-1.01	47.52	-36.74	-44.23	0.88	704.5	-159.48
32.22	-34.46	48.29	-0.95	49.15	-17.14	-45.35	1.21	705.11	-159.44
38.35	-34.46	48.29	-0.95	54.79	-17.14	-45.35	1.21	705.33	-160.2
35.83	-37.33	52.15	-0.95	35	-27.43	-47.92	1.05	706.01	-160.3
38.43	-37.33	52.15	-0.95	54.29	-27.43	-47.92	1.05	706.2	-160.79
43.63	-31.29	49.83	-1.01	40.56	-35	-39.9	0.85	706.99	-161.28
37.18	-31.29	49.83	-1.01	37.91	-35	-39.9	0.85	707	-161.77
32.22	-34.46	48.29	-0.95	41.99	-33.86	-49.31	0.97	707.12	-161.99
38.35	-34.46	48.29	-0.95	44.67	-33.86	-49.31	0.97	707.13	-162.04
35.83	-37.33	52.15	-0.95	38.18	-36.74	-44.23	0.88	707.27	-162.21
38.43	-37.33	52.15	-0.95	47.52	-36.74	-44.23	0.88	707.17	-162.18
43.63	-31.29	49.83	-1.01	49.15	-17.14	-45.35	1.21	707.13	-162.11
37.18	-31.29	49.83	-1.01	54.79	-17.14	-45.35	1.21	707.16	-162.24
32.22	-34.46	48.29	-0.95	35	-27.43	-47.92	1.05	707.13	-162.13
38.35	-34.46	48.29	-0.95	54.29	-27.43	-47.92	1.05	707.31	-162
35.83	-37.33	52.15	-0.95	40.56	-35	-39.9	0.85	707.95	-161.07
38.43	-37.33	52.15	-0.95	37.91	-35	-39.9	0.85	708.7	-160.17
43.63	-31.29	49.83	-1.01	41.99	-33.86	-49.31	0.97	709.5	-158.68
37.18	-31.29	49.83	-1.01	44.67	-33.86	-49.31	0.97	710.71	-160.46
32.22	-34.46	48.29	-0.95	38.18	-36.74	-44.23	0.88	711.15	-159.51
38.35	-34.46	48.29	-0.95	47.52	-36.74	-44.23	0.88	711.41	-159.68
35.83	-37.33	52.15	-0.95	49.15	-17.14	-45.35	1.21	712.09	-158.33
38.43	-37.33	52.15	-0.95	54.79	-17.14	-45.35	1.21	712.24	-158.1
43.63	-31.29	49.83	-1.01	35	-27.43	-47.92	1.05	712.37	-157.97
37.18	-31.29	49.83	-1.01	54.29	-27.43	-47.92	1.05	712.79	-156.69

SPA35				SPA145				Turtle	
X	Y	Z	Rot ang	X	Y	Z	Rot ang	X	Y
32.22	-34.46	48.29	-0.95	40.56	-35	-39.9	0.85	712.86	-156.55
38.35	-34.46	48.29	-0.95	37.91	-35	-39.9	0.85	712.98	-156.49
35.83	-37.33	52.15	-0.95	41.99	-33.86	-49.31	0.97	713.28	-156.58
38.43	-37.33	52.15	-0.95	44.67	-33.86	-49.31	0.97	713.57	-156.17
43.63	-31.29	49.83	-1.01	38.18	-36.74	-44.23	0.88	714.27	-155.88
37.18	-31.29	49.83	-1.01	47.52	-36.74	-44.23	0.88	714.61	-157.79
32.22	-34.46	48.29	-0.95	49.15	-17.14	-45.35	1.21	714.81	-158.04
38.35	-34.46	48.29	-0.95	54.79	-17.14	-45.35	1.21	714.99	-158.2
35.83	-37.33	52.15	-0.95	35	-27.43	-47.92	1.05	715.34	-157.84
38.43	-37.33	52.15	-0.95	54.29	-27.43	-47.92	1.05	715.58	-157.92
43.63	-31.29	49.83	-1.01	40.56	-35	-39.9	0.85	715.6	-157.82
37.18	-31.29	49.83	-1.01	37.91	-35	-39.9	0.85	715.71	-157.97
32.22	-34.46	48.29	-0.95	41.99	-33.86	-49.31	0.97	716.29	-157.94
38.35	-34.46	48.29	-0.95	44.67	-33.86	-49.31	0.97	717.78	-158.83
35.83	-37.33	52.15	-0.95	38.18	-36.74	-44.23	0.88	721.32	-159.73
38.43	-37.33	52.15	-0.95	47.52	-36.74	-44.23	0.88	722.32	-160.23
43.63	-31.29	49.83	-1.01	49.15	-17.14	-45.35	1.21	723.06	-160.7
37.18	-31.29	49.83	-1.01	54.79	-17.14	-45.35	1.21	723.9	-160.98

Figure S1: Difference between SPA 35 and SPA145 rotation motion angle distribution for different paths (extension for figure 7 of reliability of SPA during path)

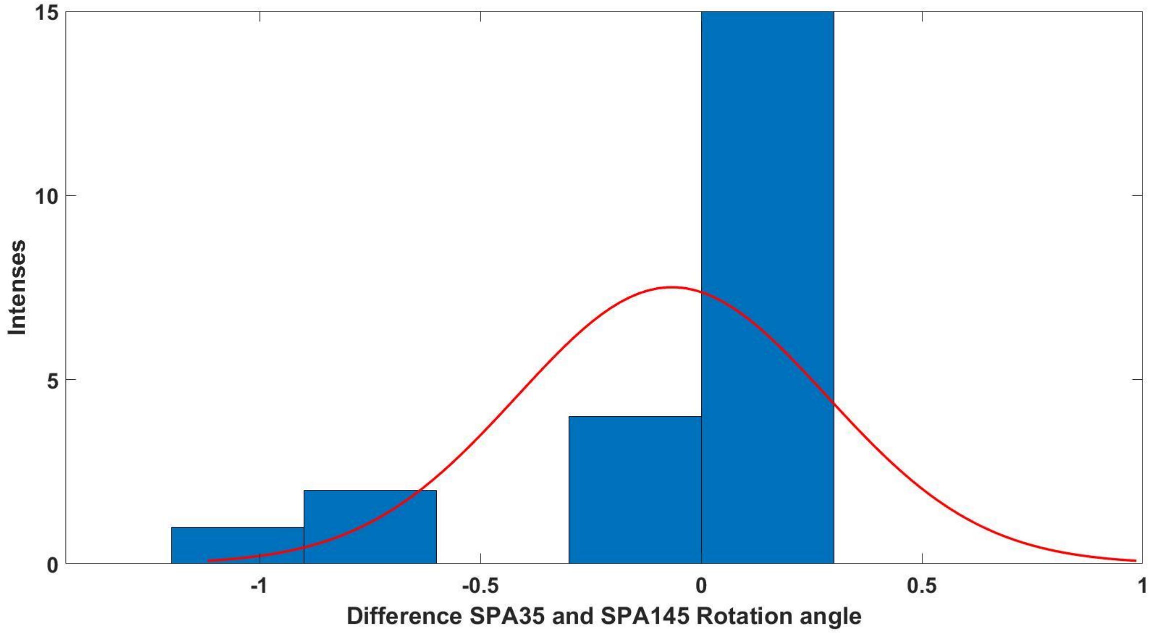

Figure S1 Difference between SPA 35 and SPA145 rotation motion angle distribution for different paths (extension for figure 7 of reliability of SPA during path)

Figure S2: Difference between SPA60 and SPA120 Rotation motion angle distribution for different paths (extension for figure 7 of reliability of SPA during path)

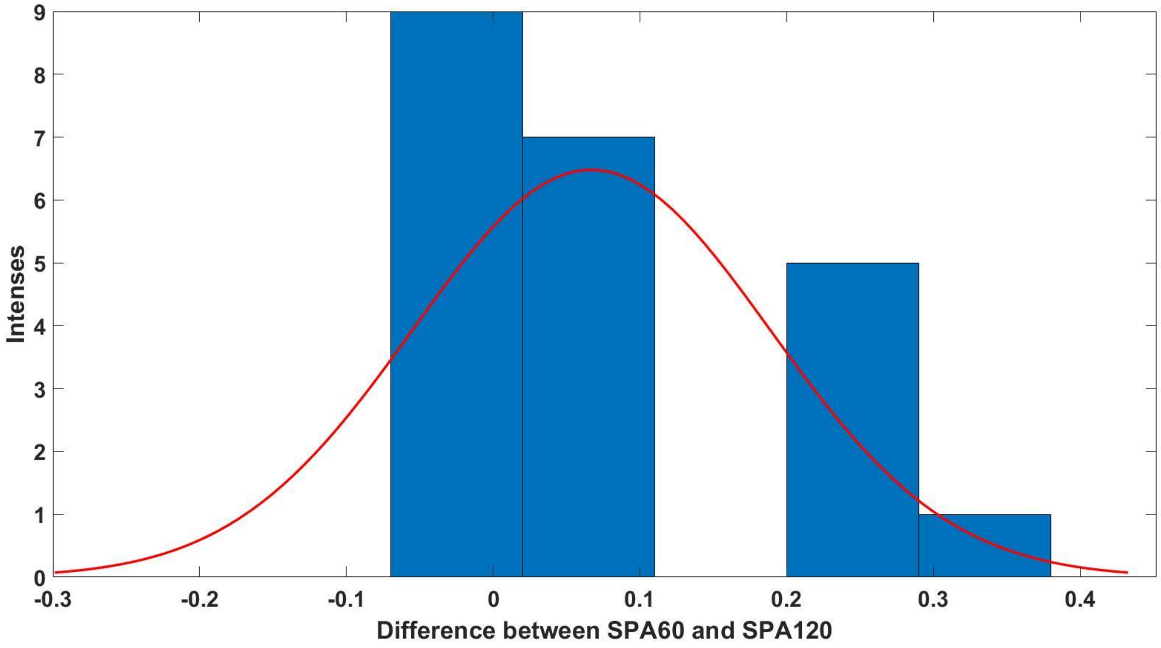

Figure S2 Difference between SPA60 and SPA120 Rotation motion angle distribution for different paths (extension for figure 7 of reliability of SPA during path)

Figure S3, S4, S5, and S6 Details of robot design and fabrication Figures: -

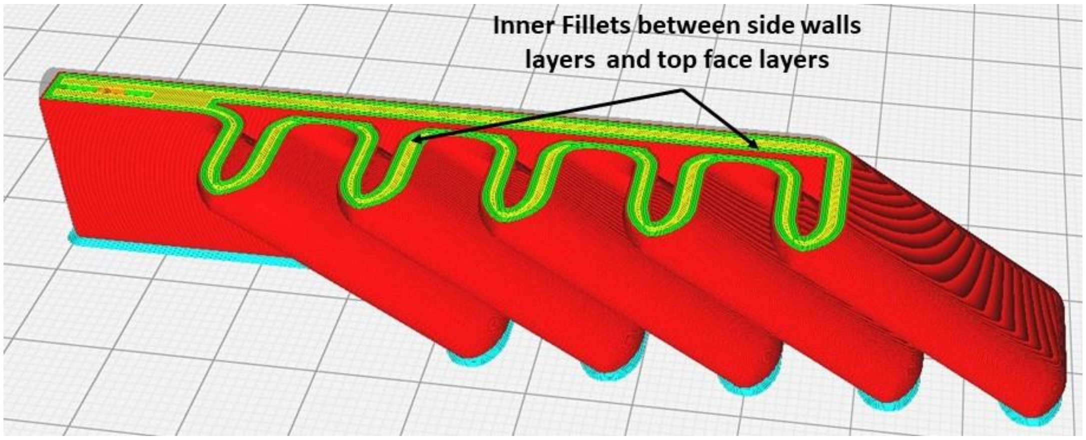

Figure S3 Screenshot from 3D Printer software shows the added fillets in the actuator design for facilitating 3D printing.

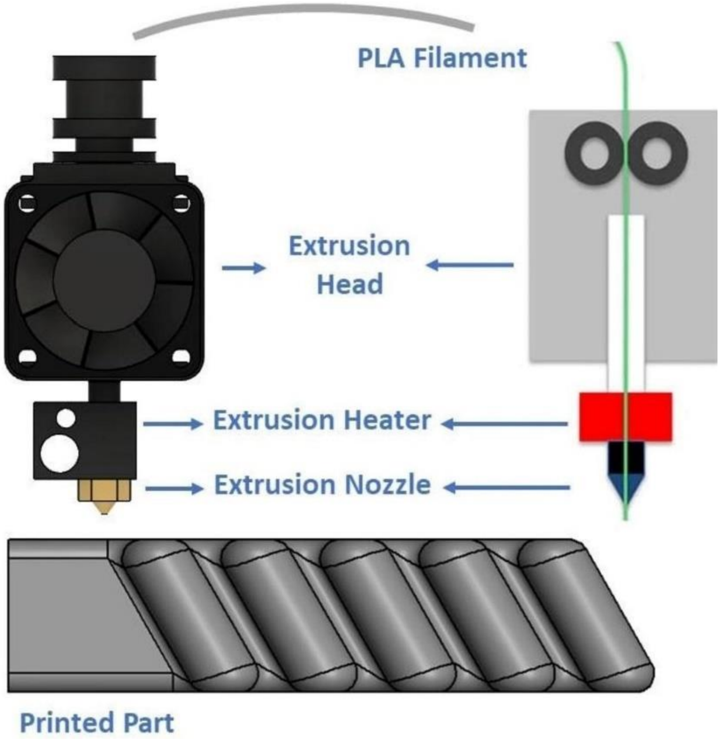

Figure S4 3D Printing details

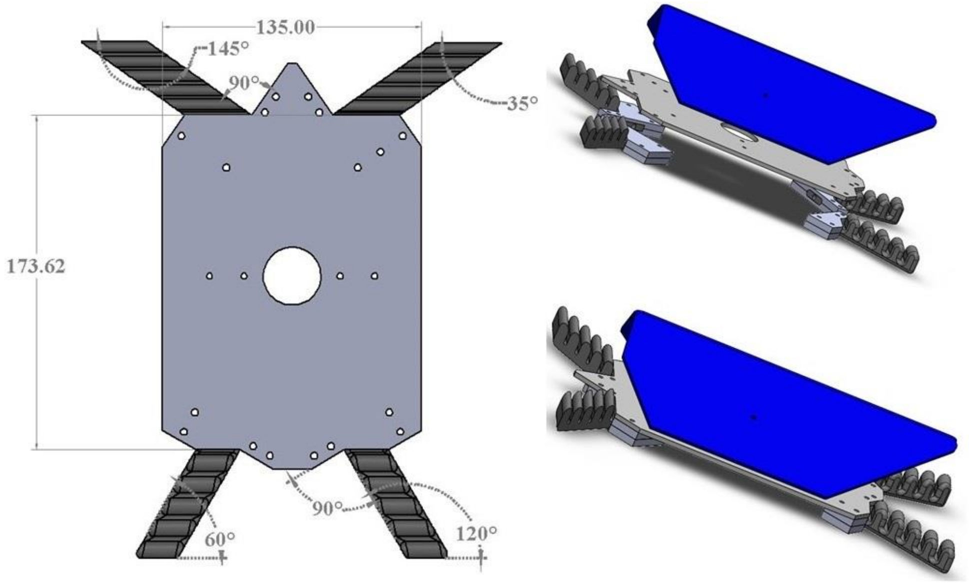

Figure S5 CAD Model details for Turtle robot

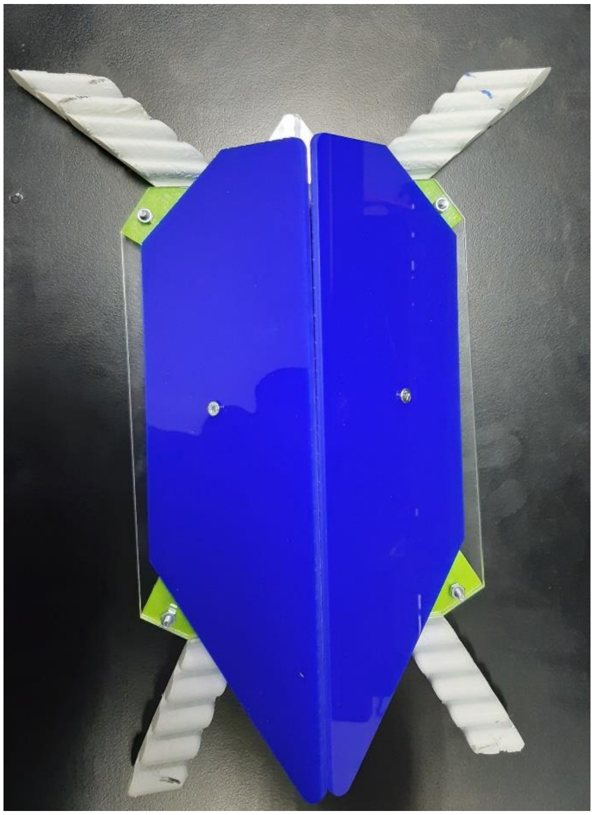

Figure S6 Turtle Robot

Figure S7: Experimental vs FEA for (a) SPA35, (b) SPA145, (c) SPA60, and (d) SPA120 for Rotation angle.

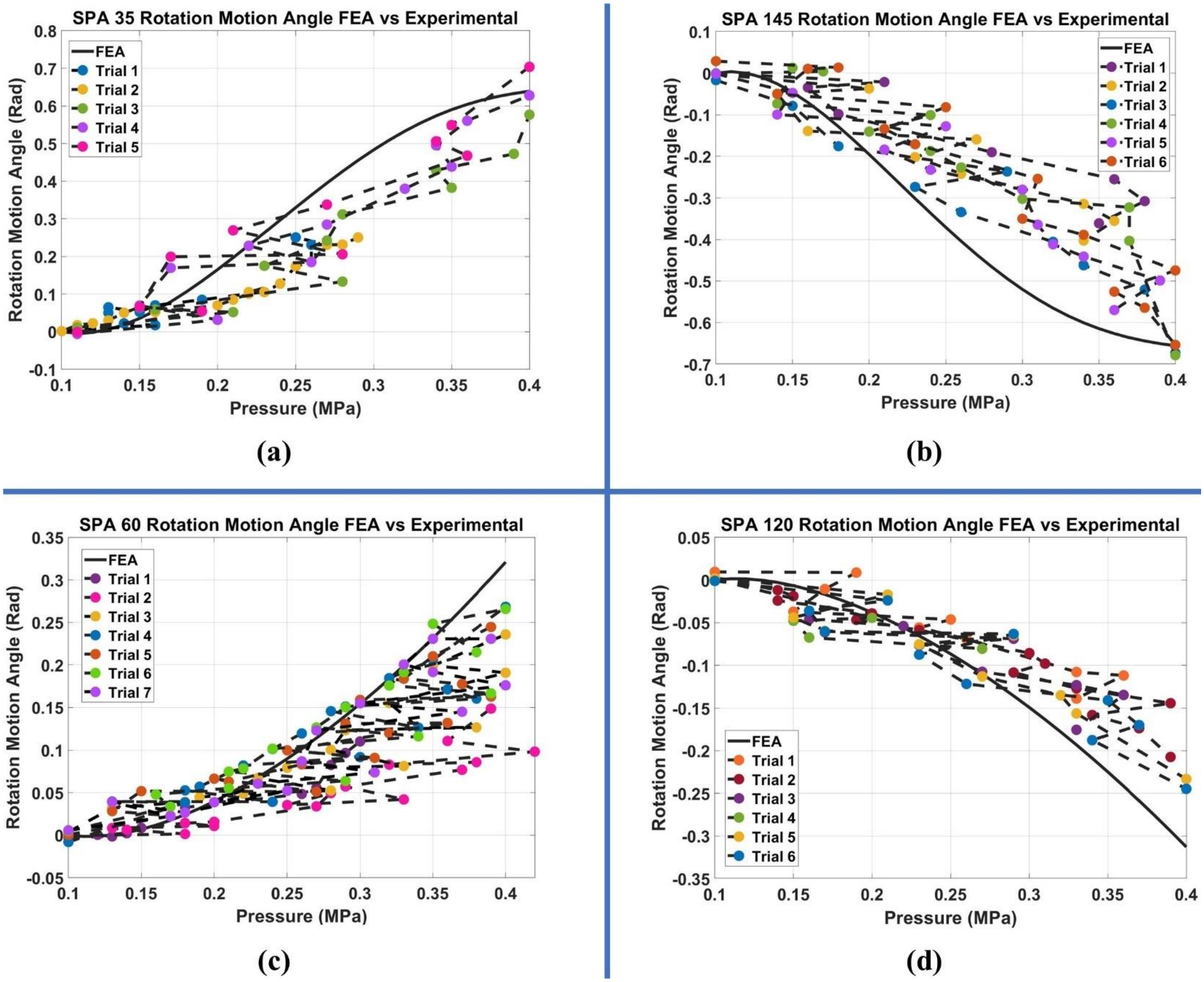

Figure S7:- Experimental vs FEA for (a) SPA35, (b) SPA145, (c) SPA60, and (d) SPA120 for Rotation angle.

Figure S8: FEA for (a) FEA (X, Y, Z) work envelope in 3D, (b) FEA (X, Y) work envelope vs applied pressure, and (c) FEA Z points (rotation in 3D) work envelope vs applied pressure.

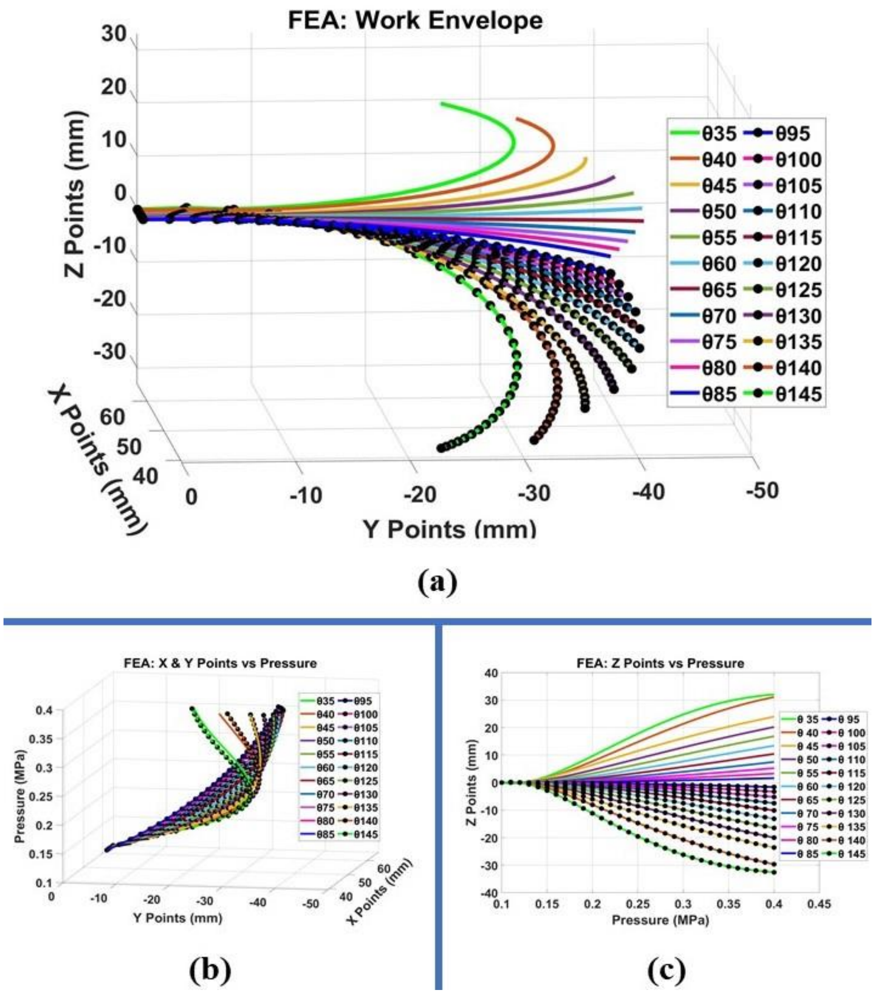

Figure S8 (a) FEA (X, Y, Z) work envelope in 3D, (b) FEA (X, Y) work envelope vs applied pressure, and (c) FEA Z points (rotation in 3D) work envelope vs applied pressure.
